# Scope of tetrazolo[1,5-*a*]quinoxalines in CuAAC reactions for the synthesis of triazoloquinoxalines, imidazoloquinoxalines, and rhenium complexes thereof

**DOI:** 10.3762/bjoc.18.111

**Published:** 2022-08-24

**Authors:** Laura Holzhauer, Chloé Liagre, Olaf Fuhr, Nicole Jung, Stefan Bräse

**Affiliations:** 1 Institute of Biological and Chemical Systems, Karlsruhe Institute of Technology, Hermann-von-Helmholtz-Platz 1, 76344 Eggenstein-Leopoldshafen, Germany; 2 Institute of Nanotechnology, Karlsruhe Institute of Technology, Hermann-von-Helmholtz-Platz 1, 76344 Eggenstein-Leopoldshafen, Germany; 3 Karlsruhe Nano Micro Facility (KNMF), Karlsruhe Institute of Technology, Hermann-von-Helmholtz-Platz 1, 76344 Eggenstein-Leopoldshafen, Germany; 4 Institute of Organic Chemistry, Karlsruhe Institute of Technology, Fritz-Haber-Weg 6, 76131 Karlsruhe, Germany

**Keywords:** click reaction, CuAAC, denitrogenative annulation, imidazole, metal complexes, quinoxaline, tetrazole, triazole

## Abstract

The conversion of tetrazolo[1,5-*a*]quinoxalines to 1,2,3-triazoloquinoxalines and triazoloimidazoquinoxalines under typical conditions of a CuAAC reaction has been investigated. Derivatives of the novel compound class of triazoloimidazoquinoxalines (TIQ) and rhenium(I) triazoloquinoxaline complexes as well as a new TIQ rhenium complex were synthesized. As a result, a small 1,2,3-triazoloquinoxaline library was obtained and the method could be expanded towards 4-substituted tetrazoloquinoxalines. The compatibility of various aliphatic and aromatic alkynes towards the reaction was investigated and the denitrogenative annulation towards imidazoloquinoxalines could be observed as a competing reaction depending on the alkyne concentration and the substitutions at the quinoxaline.

## Introduction

**Scheme 1:**
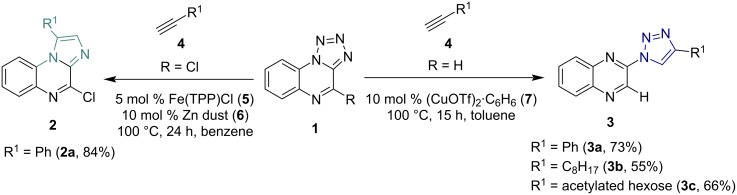
Reactions of tetrazoloquinoxalines **1** to 1,2,3-triazoloquinoxalines **3** via CuAAC and denitrogenative annulation to imidazo[1,2-*a*]quinoxalines **2** catalyzed by an iron porphyrin catalyst **5** in combination with Zn. The scheme includes all quinoxaline-based derivatives that were obtained by these procedures so far [10–12].

Quinoxalines are amongst the most versatile N-heterocyclic compounds, combining a straightforward synthesis with a diverse set of possible functionalizations and a wide range of applications in drug development and materials sciences [1]. Different quinoxaline derivatives possess antibacterial [2], antifungal [3], and antiviral properties [4] and form the core structure of commercially available drugs like brimonidine, varenicline, and quinacillin [5]. Quinoxalines can also be used in organic solar cell polymers [1,6] and have been described as donor moieties in many TADF and OLED compounds [7–9]. Amongst many other possible ways to modify and extend the core structure of quinoxalines, the conversion of tetrazolo[1,5-*a*]quinoxalines offers several advantages, as tetrazolo[1,5-*a*]quinoxalines can be used as quinoxaline-azide precursor, serving as a precursor for new nitrogen-enriched quinoxaline-based structures. Literature-known procedures for such a quinoxaline modification starting from tetrazolo[1,5-*a*]quinoxalines **1** are the synthesis of 1,2,3-triazoloquinoxalines **3** via copper-catalyzed azide–alkyne cycloaddition (CuAAC) [10] and the synthesis of imidazo[1,2-*a*]quinoxalines **2**, which was recently reported for the first time using tetraphenylporphyrin iron(III) chloride as a catalyst (Scheme 1) [11].

While the target compounds, 1,2,3-triazoloquinoxalines **3** and imidazo[1,2-*a*]quinoxalines **2**, offer a wide range of possible applications, the current knowledge on their formation from tetrazolo[1,5-*a*]quinoxalines **1** is still limited. Triazole-linked N-heterocycles like pyridotriazoles and quinolinotriazoles exert a variety of favorable biological properties like anticancer and antimicrobial activities as well as protein kinase inhibition [10,13–15]. Moreover, a vast diversity of metal complexes incorporating 1,2,3-triazoles as ligands have been reported [16–18]. Triazole ligands with N-heterocycles such as Pyta (4-(2-pyridyl)-1,2,3-triazole) and related structures were employed to obtain novel metal complexes as catalysts [19–20] and imaging probes [21], as well as metallosupramolecular assemblies [22]. The so-called inverse constellation of the triazole bound to the heterocycle via the nitrogen has been shown to possess interesting properties compared to the “regular” form [23–24], underlining the importance of accessing the desired triazole-heterocycle products from ring-fused 1,2,3,4-tetrazoles. Although some triazoloquinoxalines with a spacer moiety have been reported in the past [25–26], only three successfully synthesized derivatives of 1,2,3-triazoloquinoxalines **3** without a spacer are known [10,12]. To date, only one study describes the formation of a metal complex with an inverse triazoloquinoxaline ligand [12].

Imidazo[1,2-*a*]quinoxalines have been reported to possess anticancer and antitumor properties [27–28] and show activity as adenosine receptor antagonists [29] as well as PDE4 inhibitors [30]. The reaction of ring-fused tetrazoles to imidazole-fused products via denitrogenative annulation leading to **2** is, compared to the ever-present CuAAC, less known and was only shown with one example so far [11].

The study described herein intends to investigate the reactivity of tetrazolo[1,5-*a*]quinoxalines **1** concerning the competing formation of 1,2,3-triazoloquinoxalines **3** and imidazo[1,2-*a*]quinoxalines **2** under conditions known for copper-catalyzed azide–alkyne cycloaddition (CuAAC) [10]. The currently published porphyrin-catalyzed process requires glovebox conditions and the use of an expensive catalyst [11]. We intend to elucidate the conditions that favor the triazole formation or the imidazole, giving indications for alternative strategies to access imidazo[1,2-*a*]quinoxalines.

## Results

**Scheme 2:**
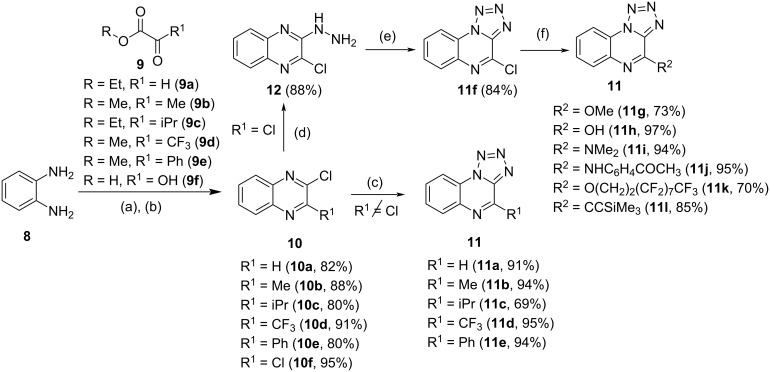
Synthesis of tetrazolo[1,5-*a*]quinoxalines. Reaction conditions: (a) **9**, THF or 4 M HCl, 70*–*110 °C, 2–3 h; (b) POCl_3_, 100 °C, 2*–*4 h, yields over two steps are given above; (c) NaN_3_, DMF, 60*–*80 °C, 2*–*26 h; (d) H_2_NNH_2·_H_2_O, EtOH, 25 °C, 21 h; (e) NaNO_2_, AcOH/H_2_O, 0 °C, 3 h; (f) diverse conditions, see Supporting Information File 1 for details.

All tetrazolo[1,5-*a*]quinoxaline precursors were synthesized in three to five steps from commercially available *o*-1,2-phenylenediamine (**8**, Scheme 2). Condensation to the corresponding quinoxalinone and subsequent chlorination was followed by introduction of the tetrazole moiety into the molecule via sodium azide to yield **11a**–**e**. Alternatively, 4-chlorotetrazolo[1,5-*a*]quinoxaline (**11f**) was obtained after reaction of 2,3-dichloroquinoxaline (**10f**) with hydrazine and sodium nitrite. Further derivation of **11f** led to compounds **11g**–**l** which include different substitution patterns for R^2^. The tetrazolo[1,5-*a*]quinoxaline products **11a**–**l** were obtained in yields of 36% to 81% for all steps (see Supporting Information File 1 for the entire scheme).

**Scheme 3:**
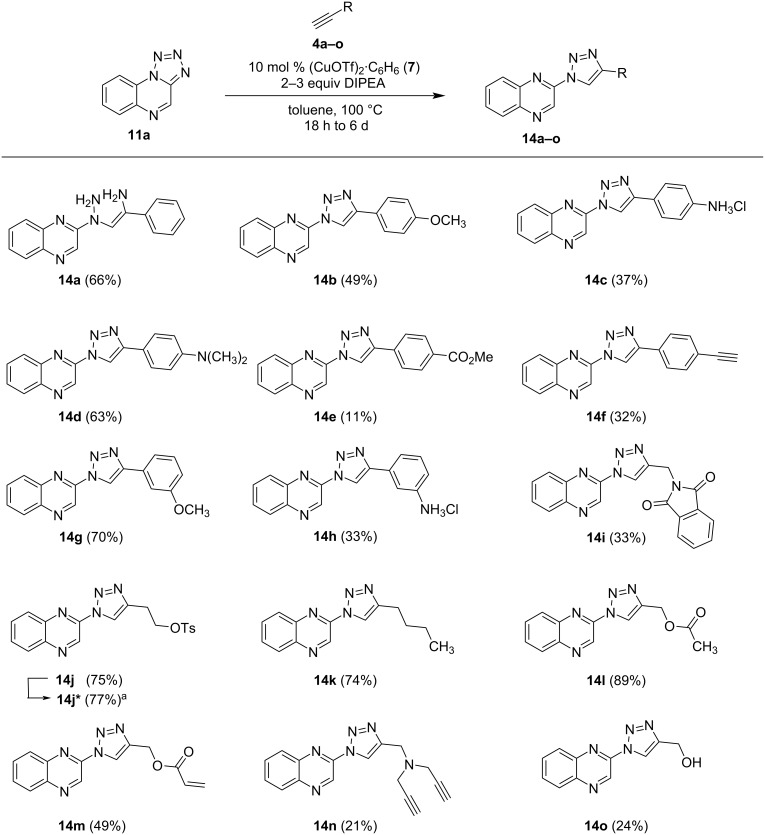
Synthesis of 1,2,3-triazole-substituted quinoxalines via CuAAC from tetrazolo[1,5-*a*]quinoxaline (**11a**). ^a^Synthesis of 14j* from **14j** = Et_2_NH, K_2_CO_3_, DMF, 70 °C, 1 d.

Starting from **11a**, a small library of 1,2,3-triazole-substituted quinoxalines was synthesized applying the method of Chattopadhyay et al. [10] with minor adjustments. Altogether, a series of 21 different aliphatic and aromatic terminal alkynes were reacted with tetrazolo[1,5-*a*]quinoxaline and Cu(I) triflate as a catalyst at 100 °C in dry toluene, using DIPEA as an additional base. The use of DIPEA resulted in faster conversions and slightly higher yields (see Table S1, Supporting Information File 1). In total, 14 novel triazoloquinoxalines could be obtained successfully with yields ranging from 11% to 89%, showing the compatibility of the conversion with a diverse set of alkynes. Reduction of the starting material **11a** to quinoxalin-2-amine as a side product was observed in some cases (see Supporting Information File 1 for details). The wide range of tolerated alkynes allows the installation of functional groups for further modification of the triazoloquinoxalines. For example, the alkyne-bearing compound **14f** can be used for further CuAAC reactions and compounds including leaving groups, such as in **14j**, can be easily converted by nucleophilic substitutions. In addition, compounds with alkene- (**14m**) or hydroxy- (**14o**) functionality can also be applied for various other reactions. Possible modifications of compounds **14** were exemplarily shown for **14j**, which was converted to the amine-substituted product 14j^*^ via nucleophilic substitution with a yield of 77% (see Scheme 3). However, alkynes **4** with reactive and electron-withdrawing functional groups, such as carboxylic acids, were not tolerated in the reaction of **11** to **14**, or led to lower yields (for not successful reactions, please see Supporting Information File 1). The highest yields could be observed for the compounds **14j–l** (Scheme 3).

To extend the scope of the reaction of tetrazolo[1,5-*a*]quinoxalines with alkynes under CuAAC conditions, different substituted quinoxalines **11** were reacted with hexyne (**4k**) as a model system (Table 1). A variation of the experimental setting for the substituted derivates found that the reaction gives better yields in the absence of DIPEA (see Table S2, Supporting Information File 1). Therefore, no base was used in the following experiments to convert substituted tetrazolo[1,5-*a*]quinoxalines with alkynes. Under these conditions, in addition to the reaction to the expected 1,2,3-triazoloquinoxalines, denitrogenative annulation was observed as a competing reaction, leading to imidazole product **16**. This competing reaction was also observed for an aromatic alkyne (see Supporting Information File 1), but did not occur in any of the previous experiments with unsubstituted tetrazolo[1,5-*a*]quinoxalines. Moreover, the denitrogenative reduction to quinoxaline-2-amines **17** was noticed as a side reaction. Depending on the residue in 4-position (R, Table 1) on the pyrazine ring of the tetrazolo[1,5-*a*]quinoxaline, the formation of either the triazole or the imidazole product or both products occurred. For groups with electron-donating properties or a positive mesomeric effect combined with a low steric demand, such as methyl and methoxy groups, the triazole product was preferably formed. Increased steric demand of the groups such as for isopropyl residues led to the formation of the imidazole product instead. When using starting materials that incorporate functional groups with strong electron-withdrawing effects such as trifluoromethyl or chlorine, the imidazole product **16** was formed without any detectable amount of the triazole compound **15**.

**Table 1:** Results of the reaction of different tetrazolo[1,5-*a*]quinoxalines **11** with hexyne (**4k**) under CuAAC conditions.^a^

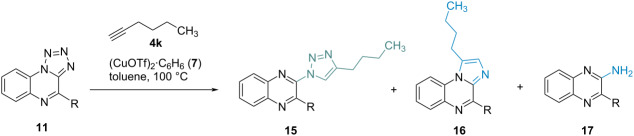						

Entry	Starting material	R	Equiv of hexyne (**4k**)	Yield [%]		

				**15a**	**16a**	**17a**

1	**11b**	Me		31	0	18
2	**11b**	Me	2	17	0	nd
3	**11b**	Me	1.1	15	0	33^b^

				**15b**	**16b**	**17b**

4	**11c**	iPr	5	8	17	11
5	**11c**	iPr	2.5	0	13	34
6	**11c**	iPr	1.1	0	22	41

				**15c**	**16c**	**17c**

7	**11d**	CF_3_	8	0	0	41

8	**11d**	CF_3_	2	0	17	66

				**15d**	**16d**	**17d**

9	**11e**	Ph	5	11	0	11
10	**11e**	Ph	2	11	0	24
11	**11e**	Ph	1.1	9	0	31

				**15e**	**16e**	**17e**

12	**11f**	Cl	5	0	4	23

				**15f**	**16f**	**17f**

13	**11g**	OMe	2	49	0	0

				**15g**	**16g**	**17g**

14	**11j**	NHC_6_H_4_COCH_3_	2.5	8	0	9

				**15h**	**16h**	**17h**

15	**11k**	O(CH_2_)_2_(CF_2_)_7_CF_3_	15	62	13	0
16	**11k**	O(CH_2_)_2_(CF_2_)_7_CF_3_	5	50	15	21
17	**11k**	O(CH_2_)_2_(CF_2_)_7_CF_3_	2	10	19	55
18	**11k**	O(CH_2_)_2_(CF_2_)_7_CF_3_	1.1	0	22	29

^a^1.1–5 equiv hexyne, 10 mol % (CuOTf)_2_·C_6_H_6_ (**7**), toluene, 100 °C, 3 d. Full results including also not successful conversions are available in Supporting Information File 1; ^b^obtained with impurities, nd = not determined.

In the cases when both products were observed, the ratio of the gained products depended strongly on the amount of alkyne used in the reaction. To investigate this effect, the perfluoro-substituted compound **11k** was used as a model substrate as it showed the formation of both products under standard conditions with two equivalents of hexyne. When the amount of alkyne was reduced to 1.1 equivalents, no more triazole product could be isolated; the yield of the imidazole product was only slightly affected. In contrast, an increase in the alkyne amount led to a noticeable improvement of the yield from 10% up to 62%. In parallel, the imidazole formation decreased from 22% to 13% under the same conditions. The experiments were thus repeated with the methyl-, isopropyl- and phenyl-substituted compounds **11b**, **11c**, and **11e**; again, increasing the amount of alkyne led to increased formation of the triazole product, especially for **11b** and **11c**.

**Scheme 4:**
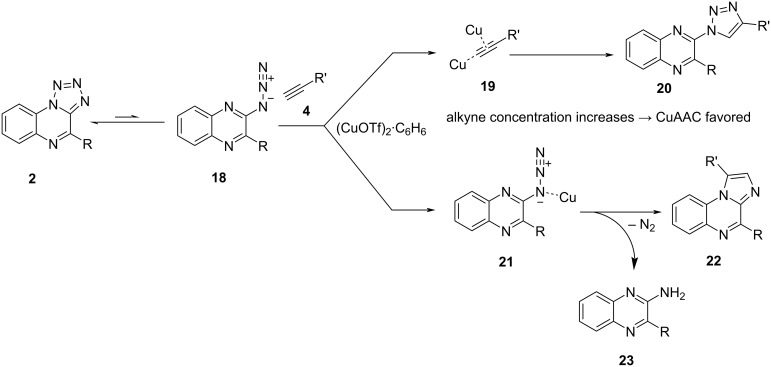
Mechanism of CuAAC vs denitrogenative annulation.

These observations match with the general mechanism of CuAAC reactions and denitrogenative annulation according to Roy et al. [11]. Copper-catalyzed azide–alkyne cycloadditions are initiated via the (dual) complexation of the alkyne, whereas denitrogenative annulation on 1,2,3,4-tetrazoles is assumed to start via complexation of the open-form azide **18** (see Scheme 4). Increasing the amount of alkyne **4** increases the probability of the alkyne being coordinated in contrast to the tetrazole, which leads to launching of the CuAAC cycle. The probability of coordination on the tetrazole should also be indirectly impacted by this. However, the imidazole formation is only slightly decreased when the alkyne concentration is raised for compounds **11c** and **11k**. In contrast to that, no imidazole formation could be observed for compound **11d** when 8 equivalents of alkyne were used. Therefore, further investigations will be necessary to determine why the imidazole formation is not completely suppressed in some cases when increasing the alkyne concentration drastically.

The denitrogenative annulation reaction was then further explored using derivate **11d** regarding the influences of different catalysts and additives (for details and results see Supporting Information File 1, Tables S3 and S4). Improving this route provides an alternative to the literature-known method [11] that requires both a special porphyrin complex and glovebox conditions. Using neither silver(I) triflate nor copper(I) iodide yielded the imidazole product, indicating that the use of copper(I) triflate is crucial for the reaction to take place. The increase of the amount of catalyst did not significantly improve the yield, while the addition of a base (DIPEA) or Lewis acid (AlCl_3_) resulted in suppression of imidazole formation and almost complete conversion to the amine **17**. Addition of Zn(OTf)_2_ reduced the yield of the desired product **16** whereas addition of zinc powder seems to have different effects depending on the derivative (see Supporting Information File 1).

**Scheme 5:**
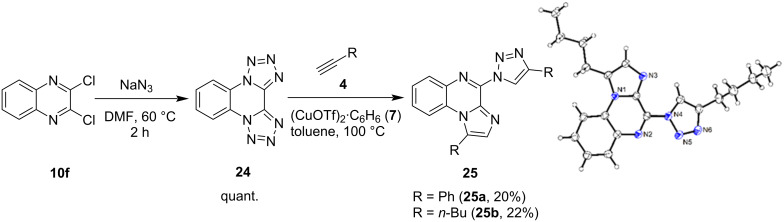
Synthesis of bis(tetrazolo)[1,5-*a*:5',1'-*c*]quinoxaline (**24)** and conversion to triazoloimidazoquinoxalines (TIQs): 2.5 equiv hexyne, 10 mol % (CuOTf)_2_·C_6_H_6_ (**7**), toluene, 100 °C, 4 h to 3 d. ORTEP diagram of triazoloimidazoquinoxaline **25b** with the thermal ellipsoids shown at 50% probability.

We could then show that the conversion of tetrazoles to both triazoles and imidazoles can occur together in the same molecule. When bis(tetrazolo)[1,5-*a*:5',1'-*c*]quinoxaline (**24**) was reacted with alkynes under Cu(I) triflate catalysis (see Scheme 5), CuAAC and denitrogenative annulation were observed in parallel to form triazoloimidazoquinoxalines (TIQs) as the main product, which have not been described in the literature yet. It remains unclear if one of the reactions takes place first and is required for the second reaction or whether both reactions occur independently of each other. Single crystals for **25b** were obtained from slow evaporation of methanol under ambient pressure and the assumed structure of the TIQ product could unambiguously be confirmed via single crystal X-ray crystallography. Several other byproducts, such as the bistriazolo product were isolated (see Supporting Information File 1).

The obtained triazoloquinoxaline and TIQ products are promising ligands for complexation with different metals. The formation of organometallic complexes is a well-established method to obtain interesting materials for catalysis [31–33] and optoelectronics [34–35], as well as for biological applications [36–37]. Therefore, the obtained triazole and TIQ products were employed to act as ligands in rhenium tricarbonyl complexes. These are especially used as CO_2_ reduction catalysts [38–40] and noninvasive imaging probes [12,41]; examples for the application in organic light-emitting diodes [35] and as photoactive CO-releasing molecule [42–43] have been reported as well.

**Scheme 6:**
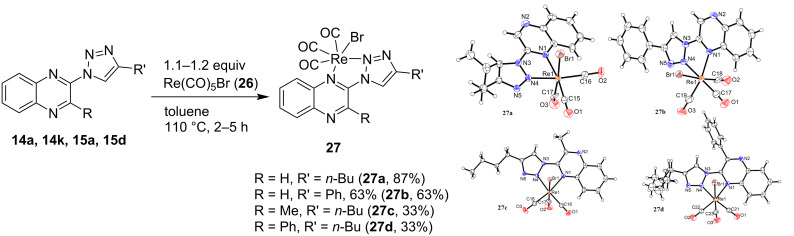
Synthesis of rhenium tricarbonyl complexes **27a–d** and ORTEP diagrams of the resulting molecular structures with the thermal ellipsoids shown at 50% probability.

**Scheme 7:**
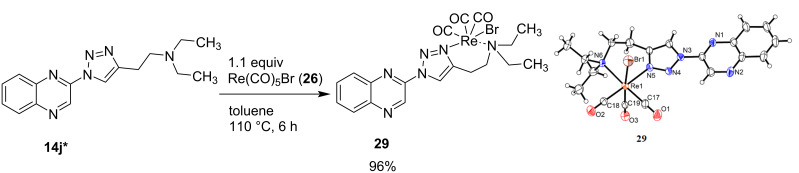
Synthesis of rhenium tricarbonyl complex **29** and ORTEP diagram of the resulting molecular structure with the thermal ellipsoids shown at 50% probability.

For the complexation experiments, compounds with three different residues on the triazole moiety (**14a**, **14k** and 14j*) were selected. Moreover, the two substituted ligands **15a** and **15d** were employed to obtain novel substituted rhenium triazoloquinoxaline complexes and the TIQ compound **25b** was tested for use as a ligand in rhenium tricarbonyl complexes. The complexes were prepared by reaction of the ligands with rhenium pentacarbonyl bromide (**26**) in toluene at 110 °C (see Scheme 6 and Scheme 7) as reported in the literature [12]. The structures of all obtained complexes could be confirmed via single crystal X-ray crystallography, verifying unambiguously the formation of the obtained products. Single crystals for complexes **27a**–**d** were obtained via slow evaporation of a solution in either methylene chloride, ethyl acetate, or deuterated chloroform under ambient conditions. The rhenium atom is coordinated to three carbonyl groups, the bromine atom and two nitrogens of the 1,2,3-triazoloquinoxaline ligand in a distorted octahedral coordination geometry in all cases. The obtained data for the alkyl-chain complex **27a** corresponds to similar published results [12].

For complex **29**, single crystals were formed from slow evaporation of a methylene chloride solution under ambient conditions. The crystal structure confirmed that rhenium is coordinated to three carbonyl groups, the bromine atom and two nitrogens of the 1,2,3-triazoloquinoxaline ligand. However, in this case, instead of coordination via the quinoxaline nitrogen and the 2-nitrogen of the triazole ring, the complex is formed via complexation of the 3-nitrogen of the triazole ring and the nitrogen of the amine side chain. The complex has a yellow color in contrast to the red complexes **27a**–**d**.

**Scheme 8:**
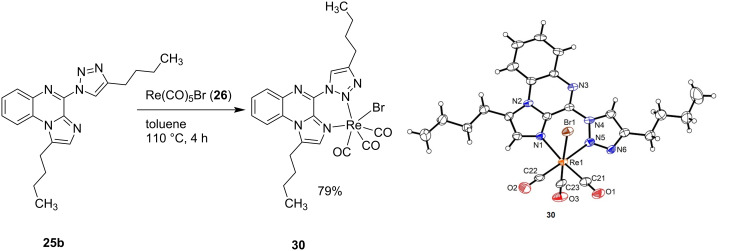
Synthesis of a TIQ rhenium complex and ORTEP diagram of the obtained product **30** with the thermal ellipsoids shown at 50% probability.

Using TIQ ligand **25b** for a complexation attempt with Re(CO)_5_Br, an orange complex (**30**) was successfully isolated in 79% yield. Single crystals were obtained from slow evaporation of a solution of **25b** in acetonitrile under ambient conditions. Crystal structure analysis of compound **30** confirmed that the rhenium complexation happens via the nitrogen of the imidazole and the 2-nitrogen of the triazole group in addition to three carbonyl groups and one bromine atom (see Scheme 8).

**Figure 1:**
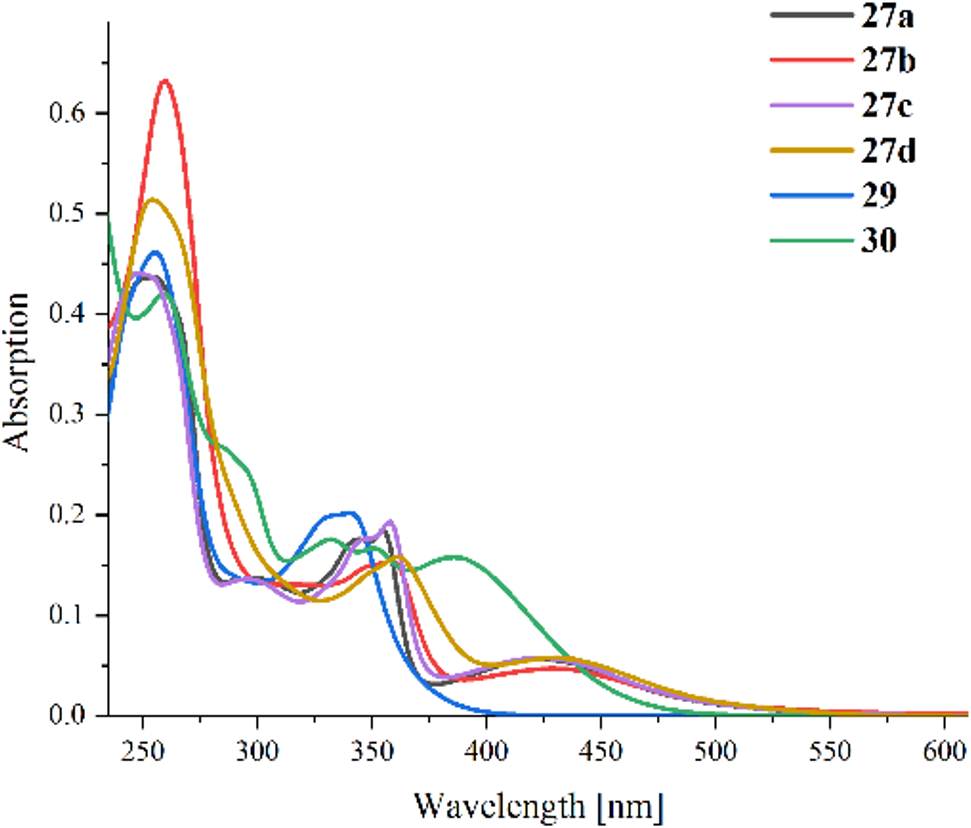
UV–vis absorption spectra of the obtained metal complexes (18 µM solutions) in acetonitrile at 20 °C.

UV–vis absorption spectra of all obtained rhenium complexes (Figure 1) and those of the free ligands (Figure S4, Supporting Information File 1) were measured in acetonitrile. The molar extinction coefficients ε of the complexes were calculated from the obtained quantitative data (see Table 2). Complexes **27a**–**d** show similar properties to the literature [12] containing a low-energy broad absorption band with a maximum at 424–432 nm (see Table 2) and an absorption maximum at around 356 nm with a shoulder peak at around 344 nm for **27a**, **27b**, and **27c**. Complex **29** displays different absorption properties due to the different complexation; it possesses a peak with a center at around 340 nm but no noticeable absorption in the range of 420–430 nm. The TIQ complex **30** shows two minor peaks at 332 nm and 350 nm and an intense broad peak at 386 nm, thus being blue-shifted compared to the triazoloquinoxaline complexes **27a**–**d**.

**Table 2:** Absorption maxima (λ_max_) and molar extinction coefficient ε at the absorption maximum [44].

Compound	λ_max_ [nm]	Log(ε) [M^−1^·cm^−1^]

**27a**	256	4.39
**27b**	260	4.54
**27c**	248	4.39
**27d**	254	4.45
**29**	256	4.40
**30**	260	4.37

**Figure 2:**
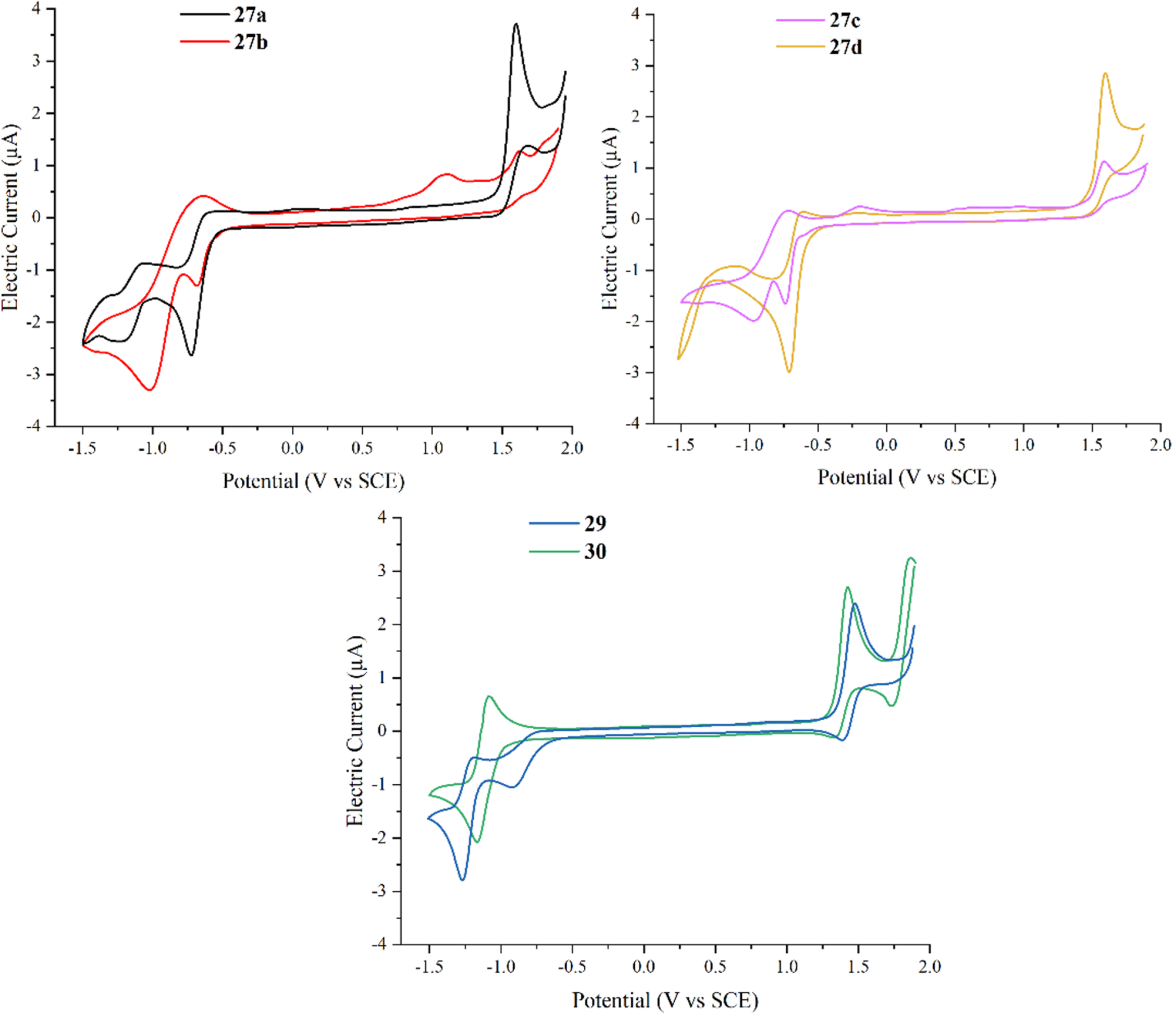
Cyclic voltammetry traces for rhenium complexes **27a**–**d**, **29** and **30**: 0.5 mM in MeCN solution with 0.1 M Bu_4_NPF_6_ under nitrogen at 25 °C, recorded at 0.1 V/s at a glassy carbon electrode and referenced to the saturated calomel electrode (SCE) using Fc/Fc^+^ as an internal standard (0.46 V vs SCE [12]).

To characterize the electrochemical properties of the obtained complexes, cyclic voltammetry measurements were performed. For complexes **27a**–**d**, irreversible oxidation previously assigned to the Re(I)/Re(II) couple [38,45] can be observed at 1.6 V vs SCE (see Table 3 and Figure 2); for complexes **29** and **30**, this peak is shifted towards 1.4 V, indicating the stronger electron-donating nature of the ligands [38]. Moreover, an additional oxidation state at 1.91 V is present for complex **30** (see Supporting Information File 1 for full trace). For the other compounds, this oxidation state is hardly recognizable as it is almost hidden beneath the increase of the curve related to oxidation of the solvent.

**Table 3:** Electrochemical data for rhenium complexes **27a**–**d**, **29** and **30**. For full scan range (−2.0 V to 2.5 V), please refer to the Supporting Information File 1 (Figures S5, S6, and S7).

Entry	Compound	*E*_ox_ [V]	*E*_Red_ [V]

1	**27a**	1.60	−0.72, −1.18
2	**27b**	1.09^a^, 1.62	−0.68, −1.02
3	**27c**	1.59	−0.74, −0.96
4	**27d**	1.60	−0.71
5	**29**	1.47	−0.92, −1.27
6	**30**	1.43, 1.91	−1.17, −1.9

^a^Minor features.

Scanning towards negative potentials, two reduction waves can be observed between −0.6 V and −1.5 V for complexes **27a–d** that can be assigned to reduction of the ligand [45]. For **29** and **30**, reduction features of the ligands are anodically shifted. The reduction of complex **30** seems to be reversible (for further experiments please see Supporting Information File 1). The anodic shift shows that the more electron-rich nature of the TIQ ligand compared to the triazoloquinoxaline ligand has a visible influence on the reduction behavior of the complex.

## Conclusion

New derivatives of 1,2,3-triazoloquinoxalines have been synthesized starting from tetrazolo[1,5-*a*]quinoxalines via CuAAC by varying the alkyne and the residues on the quinoxaline building blocks. During the investigation of the formation of 1,2,3-triazoloquinoxalines, denitrogenative annulation towards imidazole derivatives could be identified as a competing reaction for some substituted quinoxalines. Following the proposed mechanism, a dependency of obtained product ratio on the alkyne concentration was observed. These results expand the scope of accessible 1,2,3-triazoloquinoxalines and provide an alternative synthesis route from tetrazolo[1,5-*a*]quinoxalines to imidazo[1,2-*a*]quinoxalines.

For bis(tetrazolo)[1,5-*a*:5',1'-*c*]quinoxalines, the formation of triazoloimidazoquinoxalines was shown with two derivatives. Five rhenium complexes with 1,2,3-triazoloquinoxalines and a novel TIQ rhenium complex were synthesized, and their structures were confirmed via X-ray crystallography. All complexes were characterized and compared regarding their absorption and electrochemical properties. The TIQ complex could be confirmed to possess rather different properties than the triazoloquinoxaline complexes in these measurements, including a blue-shift in the absorption spectrum and anodically shifted features in cyclic voltammetry measurements.

## Abbreviations

CuAAC, copper-catalyzed azide–alkyne cycloaddition; DIPEA, *N,N*-diisopropylethylamine; OLED, organic-light emitting diode; SCE, saturated calomel electrode; TADF, thermally activated delayed fluorescence; TEMPO, 2,2,6,6-tetramethylpiperidinyloxyl; TIQ, triazoloimidazoquinoxaline.

## Supporting Information

The Supporting Information covers detailed material on the conducted experiments and their results, including unsuccessful experiments. All experimental details, including the analytical description of the obtained target compounds and byproducts, are available in Supporting Information File 1. Information on the availability of the data and the physical material of the target compounds is added to the Supporting Information File 2. Data that refers to the herein described experiments were submitted to the repository chemotion (https://www.chemotion-repository.net/). All DOIs minted for the data are linked in Supporting Information File 1. New data obtained in this study is assigned to the collection embargo numbers LSH_2021-02-02 and CML_2020-12-18. The material that was obtained in this study (target compounds, please see Supporting Information File 2) was submitted to the Molecule Archive at KIT and can be requested from there (https://compound-platform.eu/home).

File 1 Experimental part.

File 2 NMR spectra.

File 3 Information on the availability of the data and the physical material of the target compounds.
