# Supplementary material for: Scope of tetrazolo[1,5-a]quinoxalines in CuAAC reactions for the synthesis of triazoloquinoxalines, imidazoloquinoxalines, and rhenium complexes thereof
Source: Beilstein J Org Chem. 2022 Aug 24;18:1088–99. doi: 10.3762/bjoc.18.111 (PMC9443424; doi:10.3762/bjoc.18.111)
Supplement: File 1 — Experimental part. [file Beilstein_J_Org_Chem-18-1088-s001.pdf]

BEILSTEIN JOURNAL OF ORGANIC CHEMISTRY

Supporting Information

for

Scope of tetrazolo[1,5-*a*]quinoxalines in CuAAC reactions for the synthesis of triazoloquinoxalines, imidazoloquinoxalines, and rhenium complexes thereof

Laura Holzhauer, Chloé Liagre, Olaf Fuhr, Nicole Jung and Stefan Bräse

Beilstein J. Org. Chem. **2022,** *18,* 1088–1099. doi:10.3762/bjoc.18.111

Experimental part

License and Terms: This is a supporting information file under the terms of the Creative Commons Attribution License (https://creativecommons.org/licenses/by/4.0). Please note that the reuse, redistribution and reproduction in particular requires that the author(s) and source are credited and that individual graphics may be subject to special legal provisions.
The license is subject to the *Beilstein Journal of Organic Chemistry* terms and conditions: (https://www.beilstein-journals.org/bjoc/terms)

Contents

1. General remarks S2

2. Synthetic results S5

3. Absorption measurements S86

4. Electrochemical measurements S86

5. Crystallographic data S87

6. References S89

1. General remarks

The starting materials and reagents were purchased from ABCR, ACROS, ALFA AESAR, CHEMPUR, FLUKA, FLUOROCHEM, MERCK, SIGMA ALDRICH, STREM, TCI, or THERMO FISHER SCIENTIFIC and are used without further purification unless stated otherwise. Solvents of technical grade were purified via distillation prior to use (ethyl acetate, dichloromethane, cyclohexane), solvents of *p.a.* quality were purchased from ACROS, FISHER SCIENTIFIC, SIGMA ALDRICH, ROTH, or RIEDEL-DE HAËN and were used without further purification.

Air- and moisture-sensitive reactions were carried out under nitrogen or argon atmosphere in oven-dried glassware using standard Schlenk techniques.
Reactions in vials were sealed with Crimp caps; both vials and caps were purchased at CHROMA GLOBE.
Solvents were evaporated under reduced pressure at 40 °C using a rotary evaporator. For solvent mixtures, each solvent was measured volumetrically.

Flash chromatography:
Purifications via flash chromatography were performed using silica gel (SiO_2, 0.040 mm × 0.063 mm, MERCK) and quartz sand (glowed and purified with hydrochlorid acid). After removing the solvent under reduced pressure, the crude products were immobilized on Celite (SIGMA ALDRICH) and applied to the column as a solid.

For automatic flash chromatography, an INTERCHIM PuriFLASH XS 400, INTERCHIM PuriFLASH 4125 or INTERCHIM PuriFLASH 5.125 was used in combination with hand-packed silica columns (SiO_2, 0.040 mm × 0.063 mm, MERCK) as well as prepacked SIHP (silica high performance, 15 µm, 4 g/12 g/40 g) columns from INTERCHIM. Fractions were separated and collected using a diode array detector (DAD).

Thin-layer chromatography (TLC):

Reactions were monitored by thin-layer chromatography (TLC) using silica-coated aluminum plates (MERCK, silica gel 60, F_{254}). UV active compounds were detected with a UV-lamp (HANAU QUARZLAMPEN, Typ 204 AC) at 254 nm and 366 nm excitation. Moreover Seebach solution (2.5 % phosphomolybdic acid, 1.0 % Cerium(IV)sulfate tetrahydrate, 6,0 % conc. H_2SO_4 in H_2O) with subsequent heating of the TLC plate was used to stain the spots.

Liquid-chromatography mass spectrometry (LC-MS) was conducted using a device from AGILENT with HP 1100 MSD G1946 Mass Detector and a Kinetex XB-C18 column (2.6 µm, 100 x 4.60 mm) from PHENOMENEX. API-ES was used as a method of ionization and the following program was applied:

10_99_P (positive polarity): injector volume 10.0 µl, flow rate 1.0 ml/min, run time 20.0 min, solvent: water (bidistilled) 50%, acetonitrile 20%.

Melting points:
Melting points were detected on an OptiMelt MPA100 device from STANFORD RESEARCH SYSTEM.

Nuclear magnetic resonance spectroscopy (NMR):
A BRUKER Ascend 400 was used to record NMR spectra; ^{1}H NMR spectra were measured at 400 MHz, ^{13}C NMR spectra at 100 MHz and ^{19}F NMR spectra at 376 MHz. All measurements

were conducted at room temperature using $CDCl_3$ or DMSO-d_6 acquired from EURISOTOP and SIGMA ALDRICH as solvents and accordingly referenced to $CDCl_3$ (^{1}H 7.27 ppm, s / ^{13}C 77.0 ppm, t) or DMSO-d_6 (^{1}H 2.50 ppm, s / ^{13}C 39.52 ppm, sep).

Chemical shifts are given in ppm (parts per million) and the spectra were analyzed following first order spectra. The signal area was given for multiplets whereas the signal center was used for centrosymmetric signals. The signal splitting was characterized using the following abbreviations: s (singlet), d (doublet), t (triplet), q (quartet), m (multiplet), bs (broad singlet), dd (doublet of the doublet), td (triplet of the doublet) etc. For ^{13}C spectra the peaks were observed as singlet if not specifically stated otherwise.

Coupling constants *J* are given in Hz (Hertz) and the number of bonds between the coupling cores is indicated as superscripted index in front of the coupling constant. Signals of the ^{13}C spectra were assigned using DEPT90 and DEPT135 spectra (distortionless enhanced polarization transfer) as well as HSQC (heteronuclear single quantum coherence) and HMBC (heteronuclear multiple bond correlation).

Infrared spectroscopy (IR):
IR spectra were measured via ATR (Attenuated Total Reflection) on a BRUKER IFS 88. The positions of the absorption bands are given in wavenumbers $\tilde{\nu}$ in cm^{-1} and were measured in the range from 3600 cm^{-1} to 500 cm^{-1}.

Characterization of the absorption bands was done in dependence of the absorption strength with the following abbreviations: vs (very strong, 0−9%), s (strong, 10−39%), m (medium, 40−69%), w (weak, 70−89%), vw (very weak, 90−100%).

Mass spectrometry (MS):
EI-MS (electron ionization mass spectroscopy) and FAB-MS (fast atom bombardment mass spectrometry) were conducted on a FINNIGAN MAT 95 with 3-nitrobenzyl alcohol (3-NBA) as matrix and reference for high resolution. The intensity of the signals is given relative to the intensity of the highest peak (100%). For the interpretation of the spectra, molecular peaks [M]+, peaks of protonated molecules $[M + H]^+$ and characteristic fragment peaks are indicated with their mass-to-charge ratio (*m*/*z*) and their intensity in percent, relative to the base peak (100%) is given. In case of high-resolution measurements, the maximum tolerated error is ±5 ppm.

ESI-MS (electron spray ionization mass spectrometry) was conducted with a THERMOFISHER Q EXACTIVE PLUS in positive mode with a voltage of 4 kV. The tolerated error is ±5 ppm of the molecular mass. The spectra were interpreted by molecular peaks $[M]^+$, peaks of protonated molecules $[M + H]^+$ and characteristic fragment peaks and indicated with their mass-to-charge ratio (*m*/*z*).

Elemental analysis (EA):

Elemental analysis was conducted using an ELEMENTAR VARIO MICRO and a SARTORIUS M2P analytical balance. Calculated and found percentage for carbon (C), hydrogen (H), sulfur (S) and nitrogen (N) are indicated in fractions of 100%.

High-performance liquid chromatography (HPLC):
Preparative reversed-phase high performance liquid chromatography (RP-HPLC) was performed on the PuriFLASH 4125 system from INTERCHIM. A VDSpher® C18-M-SE precolumn (10 µm, 40 × 16 mm) followed by a PuriFLASH C18-AQ separation column (10 µm, 250 × 21.2 mm) was used as the stationary phase. A gradient of acetonitrile and double distilled water at a flow rate of 15 mL/min served as the mobile phase.

Absorption spectroscopy:
UV–vis spectra were recorded on a HORIBA Duetta spectrometer at 20 °C in glass cuvettes with a path length of 1 cm. For quantitative measurements, 1 mg of the compound was diluted in the appropriate amount of acetonitrile to a concentration of 18 µM. To calculate the molar extinction coefficient ε, the Beer-Lambert Law [1] was used:

$$A = \varepsilon * \mathrm{c} * \mathrm{d}$$

with A = absorbance, c = concentration of the analyte and d = length of the beam in the absorbing medium (path length of the cuvette) [1].

Cyclic voltammetry:
Cyclic Voltammetry experiments were performed at 25 °C using a GAMRY Interface 1010B potentiostat in a three-electrode electrochemical cell. A glassy carbon working electrode, platinum counter electrode and $Ag/AgNO_3$ reference electrode were employed. The working electrode was treated with a 0.05 µM slurry of polishing alumina before experiments were conducted. Scans were run at 100 mV/s under nitrogen and dry acetonitrile was used as a solvent in all cases; the solvent was deaerated with nitrogen for 5-10 minutes prior to use. As the electrolyte, 0.1 M of NBu_4PF_6 (electrochemical grade, SIGMA ALDRICH) was used. The analyte was added in a concentration of 0.5 mM; ferrocene (Fc/Fc^+, 0.46 vs saturated calomel electrode (SCE)[2]) was added as an internal standard with the same concentration after the experiment.

Single crystal X-ray diffraction (XRD):

Single crystal X-ray diffraction data were collected on a STOE STADI VARI diffractometer with monochromated Mo Kα (λ = 0.71073 Å) or Ga Kα (1.34143 Å) radiation at low temperature. Using Olex2 [3], the structures were solved with the ShelXT [4] structure solution program using Intrinsic Phasing and refined with the ShelXL [5] refinement package using Least Squares minimization. Refinement was performed with anisotropic temperature factors for all non-hydrogen atoms; hydrogen atoms were calculated on idealized positions. Disordered atoms were refined isotropically.

Crystallographic data for compounds **25b, 27a–d, 29 and 30** reported in this paper have been deposited with the Cambridge Crystallographic Data Centre as supplementary information no. CCDC-2129160–2129166. Copies of the data can be obtained free of charge from https://www.ccdc.cam.ac.uk/structures/.

Crystal data and structure refinement details of **25b, 27a–d, 29 and 30** are summarized in Table S7.

2. Synthetic results

Synthesis of starting materials:

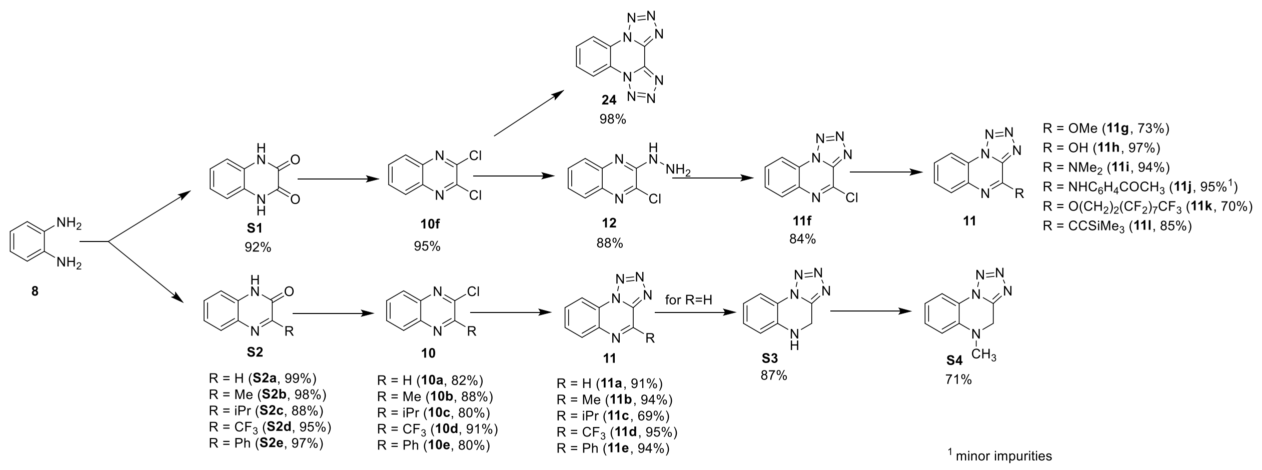

Scheme S1: Synthesis of starting materials for the CuAAC reaction.

Synthesis of triazole library:

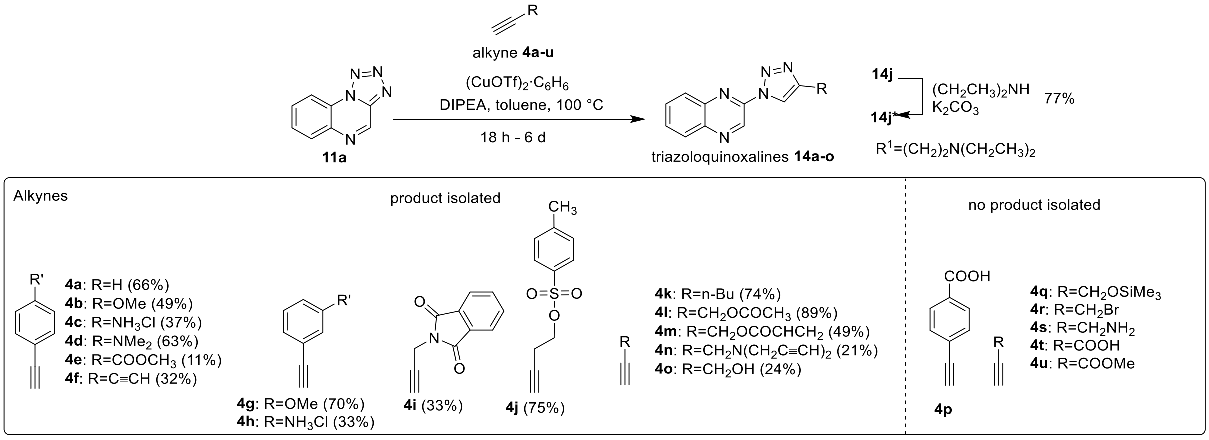

Scheme S2: Synthesis of 1,2,3-triazole-substituted quinoxalines via CuAAC from tetrazolo[1,5-*a*]quinoxaline (**11a**), full results.

Table S1*:* Synthesis of 1,2,3-triazole-substituted quinoxalines via CuAAC with or without addition of DIPEA. Reaction conditions: $(CuOTf)_2 \cdot C_6H_6$, toluene, 16 h, 100 °C.

Entry	Number of starting material	Equiv of hexyne (**4k**)	Additives	Yield [%] **14k**
1	**11a**	1.5	2 equiv. DIPEA	68%
2	**11a**	1.5	-	45%

Synthesis of pyrazole-substituted quinoxaline-triazoles/imidazoles:

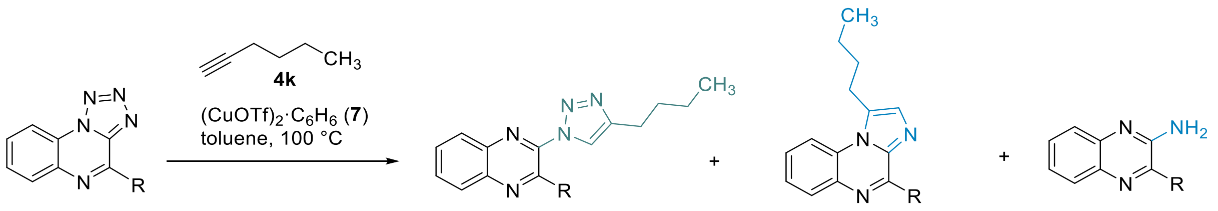

11b-l, S3, S4 15 16 17

Scheme S3: Conversion of tetrazolo[1,5-*a*]quinoxalines **11b–l, S3** and **S4** under CuAAC conditions: 1.1–5 equiv hexyne, 10 mol % $(CuOTf)_2 \cdot C_6H_6$ (**7**), toluene, 100 °C, 3 d.

Table S2: Full results of the reaction of different tetrazolo[1,5-*a*]quinoxalines **9b–e**, **12** and **13a–f** with hexyne (**4k**) after 3 d. Further results for starting material **9d** available in Table S2.

<table>
<tr><th>Entry</th><th>Number of starting material</th><th>R</th><th>Equiv of hexyne (4k)</th><th>Additives</th><th>Yield [%]
15 | 16 | 17</th></tr>
<tr><td></td><td></td><td></td><td></td><td></td><td>15a | 16a | 17a</td></tr>
<tr><td>1</td><td>11b</td><td>Me</td><td>5</td><td>-</td><td>31 | 0 | 18</td></tr>
<tr><td>2</td><td>11b</td><td>Me</td><td>2</td><td>-</td><td>17 | 0 | nd</td></tr>
<tr><td>3</td><td>11b</td><td>Me</td><td>2</td><td>2 equiv DIPEA</td><td>7 | 0 | 27</td></tr>
<tr><td>4</td><td>11b</td><td>Me</td><td>1.1</td><td>-</td><td>15 | 0 | 33[1]</td></tr>
<tr><td></td><td></td><td></td><td></td><td></td><td>15b | 16b | 17b</td></tr>
<tr><td>5</td><td>11c</td><td>iPr</td><td>5</td><td>-</td><td>8 | 17 | 11</td></tr>
<tr><td>6</td><td>11c</td><td>iPr</td><td>2.5</td><td>-</td><td>0 | 13 | 34</td></tr>
<tr><td>7</td><td>11c</td><td>iPr</td><td>1.1</td><td>-</td><td>0 | 22 | 41</td></tr>
<tr><td></td><td></td><td></td><td></td><td></td><td>15c | 16c | 17c</td></tr>
<tr><td>8</td><td>11d</td><td>CF_3</td><td>8</td><td>-</td><td>0 | 0 | 41[1]</td></tr>
<tr><td>9</td><td>11d</td><td>CF_3</td><td>2</td><td>-</td><td>0 | 17 | 66</td></tr>
<tr><td></td><td></td><td></td><td></td><td></td><td>15d | 16d | 17d</td></tr>
<tr><td>10</td><td>11e</td><td>Ph</td><td>5</td><td>-</td><td>11 | 0 | 11</td></tr>
<tr><td>11</td><td>11e</td><td>Ph</td><td>2</td><td>-</td><td>11 | 0 | 24</td></tr>
<tr><td>12</td><td>11e</td><td>Ph</td><td>1.1</td><td>-</td><td>9 | 0 | 31</td></tr>
<tr><td></td><td></td><td></td><td></td><td></td><td>15e | 16e | 17e</td></tr>
<tr><td>13</td><td>11f</td><td>Cl</td><td>5</td><td>-</td><td>0 | 4 | 23</td></tr>
<tr><td>14</td><td>11f</td><td>Cl</td><td>2</td><td>-</td><td>0 | 3 | 34</td></tr>
<tr><td></td><td></td><td></td><td></td><td></td><td>15f | 16f | 17f</td></tr>
<tr><td>15</td><td>11g</td><td>OMe</td><td>2</td><td>-</td><td>49 | 0 | 0</td></tr>
<tr><td>16</td><td>11h</td><td>OH</td><td>2.5</td><td>-</td><td>0 | 0 | 0</td></tr>
<tr><td>17</td><td>11i</td><td>NMe_2</td><td>2.5</td><td>-</td><td>0 | 0 | 0</td></tr>
<tr><td></td><td></td><td></td><td></td><td></td><td>15g | 16g | 17g</td></tr>
<tr><td>18</td><td>11j</td><td>$NHC_6H_4COCH_3$</td><td>2.5</td><td>-</td><td>8 | 0 | 9</td></tr>
<tr><td></td><td></td><td></td><td></td><td></td><td>15h | 16h | 17h</td></tr>
<tr><td>19</td><td>11k</td><td>$O(CH_2)_2(CF_2)_7CF_3$</td><td>15</td><td>-</td><td>62 | 13 | 0</td></tr>
<tr><td>20</td><td>11k</td><td>$O(CH_2)_2(CF_2)_7CF_3$</td><td>5</td><td>-</td><td>50 | 15 | 21</td></tr>
<tr><td>21</td><td>11k</td><td>$O(CH_2)_2(CF_2)_7CF_3$</td><td>2</td><td>-</td><td>10 | 19 | 55</td></tr>
<tr><td>22</td><td>11k</td><td>$O(CH_2)_2(CF_2)_7CF_3$</td><td>1.1</td><td>-</td><td>0 | 22 | 29</td></tr>
</table>

23	**11l**	$CCSiMe_3$	2.5	-	0 | 0 | 0
24	**S3**	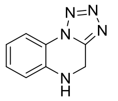	2.5	-	23[2] | 0 | 0[3]
25	**S4**	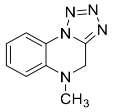	2.5	-	0 | 0 | 0

[1]obtained with impurities, [2]oxidized product, [3]impure traces determined via ^1H NMR, nd = not determined.

For entries 16 and 17, no conversion to the desired product was observed; for entry 17, 80% of starting material were reisolated. When testing entries 23 and 24, decomposition of the starting material occurred. For entry 24, 44% of starting material were reisolated whereas no compound was successfully reisolated for entry 21.

In addition, phenylacetylene **4a** as an aromatic alkyne was tested under analogous conditions. Denitrogenative annulation to compound **S9** could be observed as expected and in similar yields to the reaction with hexyne (see entry 9, Table S2).

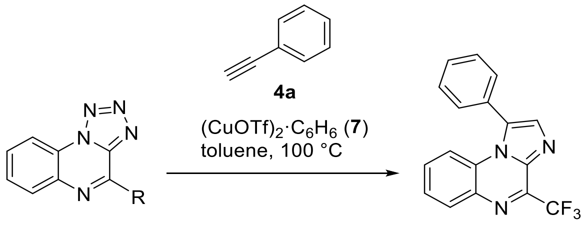

11d **S9** (19%)

Scheme S4: Conversion of tetrazolo[1,5-*a*]quinoxaline **11d** under CuAAC conditions with an aromatic alkyne: 2 equiv. phenylacetylene, 10 mol% (CuOTf)2·C_6H_6, toluene, 100 °C, 6 d.

Optimization of imidazole formation:

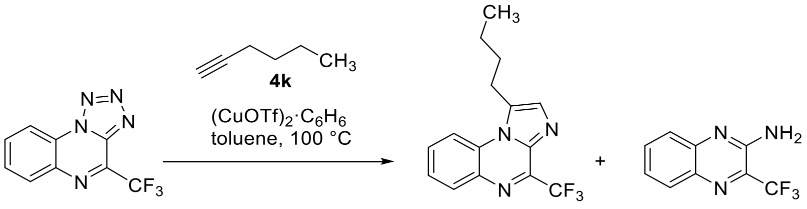

11d **16c** **17c**

Scheme S5: Conversion of tetrazolo[1,5-*a*]quinoxalines **11d** under CuAAC conditions: 1.1– 5 equiv hexyne, 10 mol % $(CuOTf)_2 \cdot C_6H_6$, toluene, 100 °C, 3 d.

Indications for a pressure-dependancy of the reaction were found when using different reaction vessels such as vials and flasks (see entries 1, 2 and 3 in Table S3); however, no conclusive result could be obtained when applying this method to other derivatives. To ensure proper reproducability, reaction vessels are given below.

Table S3: Full results for screening of different reaction conditions regarding the denitrogenative annulation with tetrazolo[1,5-*a*]quinoxaline **11d**. Standard conditions: argon atmosphere, 0.1 equiv. of $(CuOTf)_2{\cdot}C_6H_6$, 2 equiv hexyne, toluene, 100 °C, 3 d.

Entry	Catalyst	Reaction vessel	Other varied conditions	Solvent	Yield [%] **16c** | **17c**	Yield [%] **11d** (reisolated)
**1**	$(CuOTf)_2{\cdot}C_6H_6$	5 mL vial	-	toluene	17 | 66	0
**2**	$(CuOTf)_2{\cdot}C_6H_6$	50 mL vial	-	toluene	24[2] | 59	0
**3**	$(CuOTf)_2{\cdot}C_6H_6$	flask	-	toluene	28 | 11	26
**4**	$(CuOTf)_2{\cdot}C_6H_6$	flask	-	DMF	0[1] | 52	0
**5**	$(CuOTf)_2{\cdot}C_6H_6$	flask	Under air	toluene	3 | 23	53
**6**	$(CuOTf)_2{\cdot}C_6H_6$	flask	0.2 equiv. catalyst	toluene	33 | 16	20
**7**	$(CuOTf)_2{\cdot}C_6H_6$	flask	0.5 equiv. catalyst	toluene	26 | 29	6
**8**	$(CuOTf)_2{\cdot}C_6H_6$	5 mL vial	8 equiv. hexyne	toluene	0[1] | 41	43
**9**	$(CuOTf)_2{\cdot}C_6H_6$	5 mL vial	+ 0.4 equiv. Zn	toluene	30 | 53	0[1]
**10**	$(CuOTf)_2{\cdot}C_6H_6$	5 mL vial	+ 0.4 equiv. $Zn(OTf)_2$	toluene	15 | 68	0
**11**	$(CuOTf)_2{\cdot}C_6H_6$	vial	+ 3 equiv. DIPEA	toluene	0[1] | 55	0
**12**	$(CuOTf)_2{\cdot}C_6H_6$	flask	+ 2 equiv. $AlCl_3$	toluene	0 | 58	18
**13**	AgOTf	5 mL vial	-	toluene	0 | 0	92
**14**	CuI	5 mL vial	-	toluene	0 | 7	78

[1]potential traces, [2]repeated with lower yield of **16c**: see Table S5, Entry 1

Table S4: Results for screening of reaction conditions regarding the denitrogenative annulation with tetrazolo[1,5-*a*]quinoxaline **11f**. Standard Conditions: argon atmosphere, 0.1 equiv. of $(CuOTf)_2{\cdot}C_6H_6$, 2 equiv hexyne, toluene, 100 °C, 3 d.

Entry	Catalyst	Reaction vessel	Other varied conditions	Solvent	Yield [%] **16e** | **17e**	Yield [%] **11f** (reisolated)
**1**	$(CuOTf)_2{\cdot}C_6H_6$	5 mL vial	-	toluene	3 | 34	34
**2**	$(CuOTf)_2{\cdot}C_6H_6$	flask	-	toluene	6 | 6	19
**3**	$(CuOTf)_2{\cdot}C_6H_6$	5 mL vial	+ 0.4 equiv. Zn	toluene	0 | 0	72

Mechanistical studies:

The denitrogenative annulation was conducted with **11d** and addition of TEMPO in comparison to the reaction without any additives. No changes in the yield of the imidazole product **16c** were observed, indicating that the reaction does not occur via a radical pathway as described by Roy et al. [7].

Table S5: Results for mechanistical studies regarding the denitrogenative annulation with tetrazolo[1,5-*a*]quinoxaline **11d**. Standard Conditions: argon atmosphere, 0.1 equiv of $(CuOTf)_2{\cdot}C_6H_6$, 2 equiv hexyne, toluene, 100 °C, 3 d.

Entry	Catalyst	Reaction vessel	Additives	Solvent	Yield [%] **16c** | **17c**	Yield [%] **11d** (reisolated)
**1**	$(CuOTf)_2{\cdot}C_6H_6$	50 mL vial	-	toluene	15 | 74	0

**2**	$(CuOTf)_2 \cdot C_6H_6$	50 mL vial	2 equiv TEMPO	toluene	16 | 52	0

NMR of triazole vs imidazole products:

Triazole and imidazole products show noticeable differences in the ^1H NMR chemical shift of the triazole and imidazole hydrogen atoms (Figure S1). Whereas the imidazole signals are usually located around 7.5 ppm, the triazole signals can be found at 8 ppm and higher with the exception of **15c** (signal at 7.66 ppm). The shifts are in accordance with the shifts reported in literature for similar products.[6, 7]

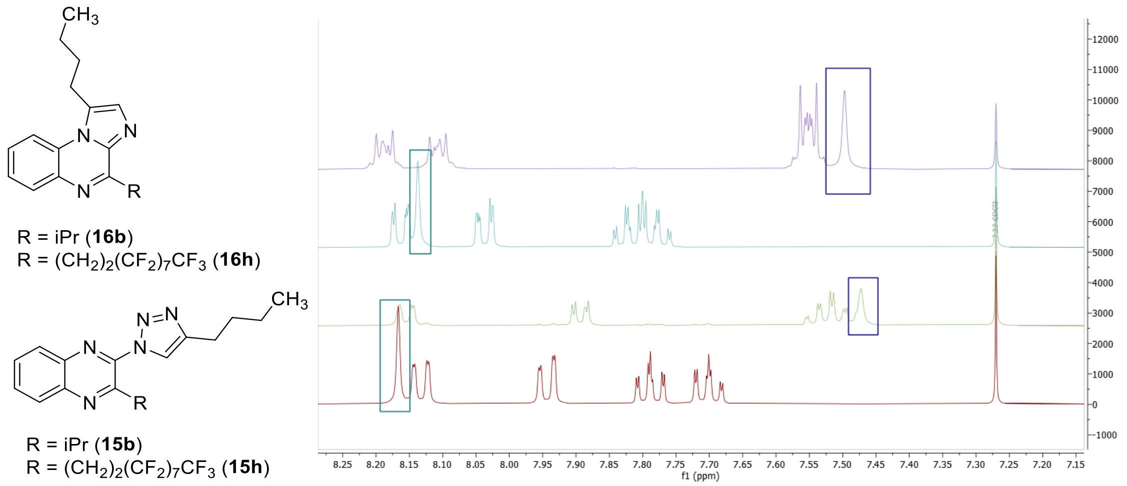

Figure S1: Excerpt from the aromatic region of the ^1H NMR spectra of two respective imidazole (**16b**, **16h**) and triazole products (**15b**, **15h**). The signals of the triazole H (green frame) are shifted into the deep-field significantly compared to their imidazole counterparts (blue frame).

The same behavior could be observed for the signals of the obtained triazoloimidazoquinoxalines (see Figure S2). In the ^1H NMR spectrum, both triazole and imidazole signals could be differentiated– whereas the triazole signal was located at 9.07 ppm, the imidazole singulet signal could be observed at 7.66 ppm for derivate **25a**.

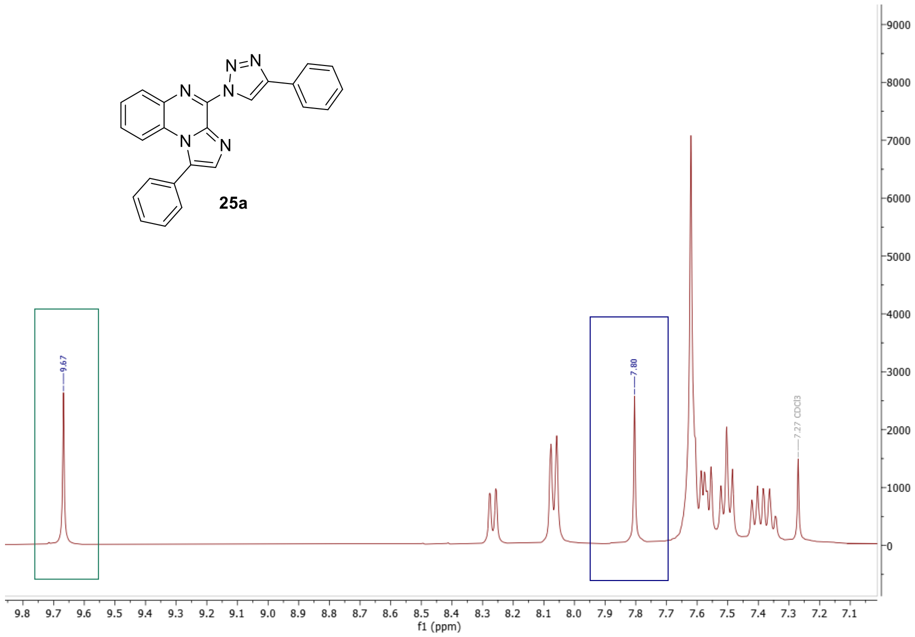

Figure S2: Excerpt from the aromatic region of the ^1H NMR spectra of TIQ **25a**. The signals of the triazole H (green frame) are shifted into the deep-field significantly compared to their imidazole counterparts (blue frame).

Results for TIQ formation:

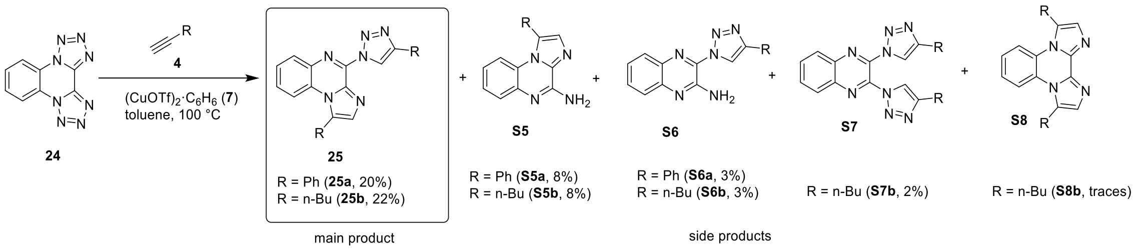

Scheme S6: Full results for the TIQ formation including all minor side products that could be isolated. No definite conclusion regarding the mechanism and the order of the triazole and imidazole formation could be drawn with the obtained products.

Table S6: Influence of reaction time, varied amounts of catalyst and alkyne on the yield of the TIQ formation.

Entry	Equiv of catalyst	Equiv of hexyne (**4k**)	Reaction Time	Yield [%] TIQ **25b**
**1**	0.1	2.5	3 d	22
**2**	0.1	2.5	3.5 h	20
**3**	0.1	10	1 d	25
**4**	0.2	10	1 d	0[1]

[1]11% of **S6b** isolated instead.

Complexation:

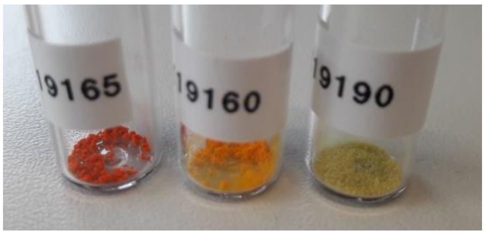

Figure S3: Colour of standard rhenium tricarbonyl triazoloquinoxaline complex **27b** (left, red) versus TIQ complex **30** (middle, orange) and side-chain complex **29** (right, yellow).

Synthesis of the compounds:

1*H*-Quinoxalin-2-one (**S2a**)

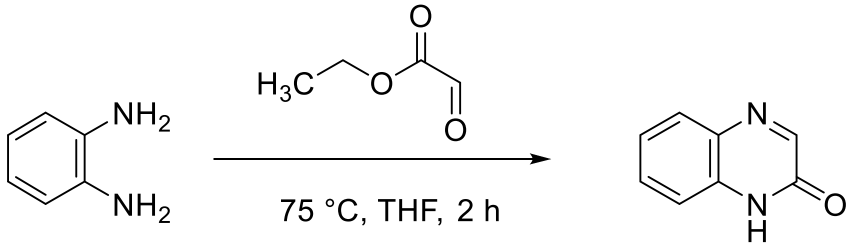

Name {P1|**S2a**}: 1*H*-quinoxalin-2-one; Formula: $C_8H_6N_2O$; Molecular Mass: 146.1460; Exact Mass: 146.0480; Smiles: O=c1cnc2c([nH]1)cccc2; InChIKey: FFRYUAVNPBUEIC-UHFFFAOYSA-N

Benzene-1,2-diamine (5.00 g, 46.2 mmol, 1.0 equiv) and ethyl 2-oxoacetate (50% in toluene, 10.4 g, 10.1 mL, 50.9 mmol, 1.10 equiv) in 80 mL of THF was stirred at 75 °C for 2 h. The reaction mixture was cooled to 25 °C; the resulting white-yellow precipitate was isolated by filtration and THF was evaporated from the filtrate under reduced pressure. The remaining solid was rinsed out with DCM and added to the solid residue. Subsequently the combined solid precipitate was washed 3x with distilled water, transferred to a flask and dried under high vacuum. The product 1*H*-quinoxalin-2-one (6.66 g, 45.6 mmol, 99% yield) was obtained as a white solid.

R_f = 0.23 (cyclohexane/ethyl acetate 1:1). ^{1}H NMR (400 MHz, DMSO-d_6, ppm) δ = 12.41 (bs, 1H, NH), 8.16 (s, 1H, NC*H*CO), 7.77–7.75 (m, 1H, CH_{ar}), 7.56–7.52 (m, 1H, CH_{ar}), 7.31–7.28 (m, 2H, CH_{ar}); ^{13}C NMR (100 MHz, DMSO-d_6, ppm) δ = 154.9 (1C, C_qO), 151.6 (1C, CH$_{ar}$), 132.0 (1C, C_q), 131.8 (1C, C_q), 130.8 (1C, CH$_{ar}$), 128.8 (1C, CH$_{ar}$), 123.3 (1C, *C*Har), 115.7 (1C, *C*Har); EI (m/z, 70 eV, 80 °C): 147 (10) $[M+1]^+$, 146 (100) $[M]^+$, 119 (17), 118 (66), 91 (24), 64 (10), 63 (11). HRMS (EI, $C_8H_6O_1N_2$): calcd 146.0475, found 146.0473; IR (ATR, ṽ) = 3077 (w), 2997 (w), 2976 (m), 2942 (m), 2870 (m), 2816 (s), 2745 (m), 2680 (m), 1696 (s), 1672 (vs), 1636 (vs), 1612 (vs), 1537 (vs), 1494 (s), 1472 (s), 1424 (vs), 1373 (s), 1353 (m), 1332 (m), 1322 (m), 1262 (m), 1254 (m), 1200 (m), 1142 (m), 1125 (m), 1021 (m), 963 (m), 950 (s), 924 (m), 891 (vs), 779 (s), 751 (vs), 724 (vs), 681 (m), 606 (vs), 554 (m), 531 (m), 510 (vs), 493 (s), 470 (vs), 399 (vs) cm^{-1}.

Additional information on the chemical synthesis is available via Chemotion repository: https://doi.org/10.14272/reaction/SA-FUHFF-UHFFFADPSC-FFRYUAVNPB-UHFFFADPSC-NUHFF-NUHFF-NUHFF-ZZZ.1
Additional information on the analysis of the target compound is available via Chemotion repository:
https://doi.org/10.14272/FFRYUAVNPBUEIC-UHFFFAOYSA-N.2

The synthesis of this compound has been previously described and the NMR data corresponds with the literature [8].

3-Methyl-1*H*-quinoxalin-2-one (S2b)

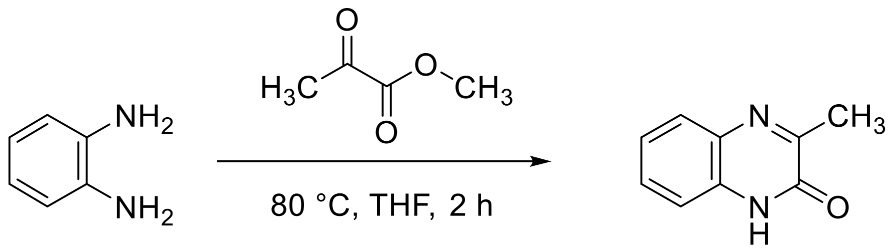

Name {P1|**S2b**}: 3-methyl-1*H*-quinoxalin-2-one; Formula: $C_9H_8N_2O$; Molecular Mass: 160.1726; Exact Mass: 160.0637; Smiles: O=c1[nH]c2ccccc2nc1C; InChIKey: BMIMNRPAEPIYDN-UHFFFAOYSA-N

To a solution of 1,2-phenylenediamine (3.02 g, 28 mmol, 1.00 equiv) in THF (50.0 mL) was added methyl 2-oxopropanoate (3.40 g, 3.01 mL, 33 mmol, 1.19 equiv). The solution was then heated to 80 °C for 2 h. After cooling down to room temperature, the formed precipitate was filtered; the remaining solution was reduced by half and filtered again (3x). The solid was then washed with methylene chloride and transferred to a flask. Traces of solvent were removed under reduced pressure. The product 3-Methyl-1*H*-quinoxalin-2-one was obtained as a white solid (4.37 g, 27 mmol, 98% yield).

R_f = 0.10 (cyclohexane/ethyl acetate 4:1). ^{1}H NMR (400 MHz, DMSO-d_6, ppm) δ = 2.40 (s, 3H, CH_3), 7.23–7.28 (m, 2H, C_6H_4), 7.44–7.48 (m, 1H, C_6H_4), 7.67–7.69 (m, 1H, C_6H_4), 12.29 (s, 1H, N*H*); ^{13}C NMR (400 MHz, DMSO-d_6, ppm) δ = 20.5 (1C, *C*H_3), 115.2 (1C, C_6H_4), 123.0 (1C, C_6H_4), 127.8 (1C, C_6H_4), 129.2 (1C, C_6H_4), 131.6 (1C, C_6H_4), 131.9 (1C, C_6H_4), 154.9 (1C,*C*NCH_3/*C*ONH), 159.1 (1C, *C*NCH_3/*C*ONH); EI (m/z, 70 eV, 100 °C): 161 (11) $[M+H]^+$, 160 (94) $[M]^+$, 132 (100), 131 (70); HRMS ($C_9H_8O_1N_2$): calcd 160.0637, found 160.0637; IR (ATR, ṽ) = 418, 453, 469, 476, 561, 584, 599, 691, 725, 751, 779, 853, 888, 928, 945, 1007, 1122, 1156, 1188, 1208, 1276, 1285, 1344, 1380, 1422, 1432, 1485, 1502, 1567, 1601, 1659, 2707, 2769, 2836, 2881, 2958, 3003, 3098 cm^{-1}.

Additional information on the chemical synthesis is available via Chemotion repository: https://doi.org/10.14272/reaction/SA-FUHFF-UHFFFADPSC-BMIMNRPAEP-UHFFFADPSC-NUHFF-NUHFF-NUHFF-ZZZ
Additional information on the analysis of the target compound is available via Chemotion repository:
https://doi.org/10.14272/BMIMNRPAEPIYDN-UHFFFAOYSA-N.4

The synthesis of this compound has been previously described in literature [9].

3-Propan-2-yl-1*H*-quinoxalin-2-one (**S2c**)

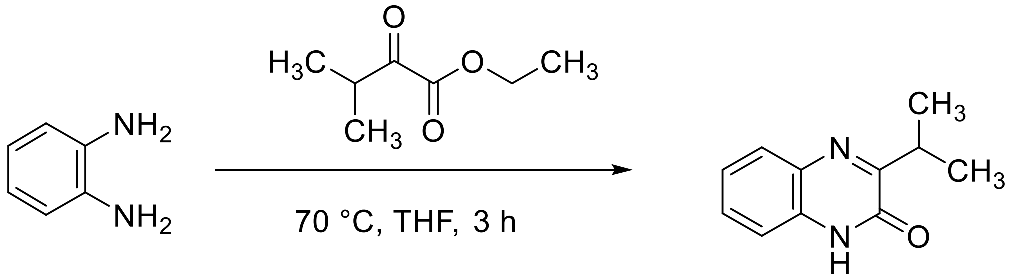

Name {P1|**S2c**}: 3-propan-2-yl-1*H*-quinoxalin-2-one; Formula: $C_{11}H_{12}N_2O$; Molecular Mass: 188.2258; Exact Mass: 188.0950; Smiles: CC(c1nc2ccccc2[nH]c1=O)C; InChIKey: CWFPSKVBKAPBPV-UHFFFAOYSA-N

To a solution of benzene-1,2-diamine (1.00 g, 9.25 mmol, 1.00 equiv) in THF (10.0 mL) was added ethyl 3-methyl-2-oxobutanoate (1.47 g, 1.48 mL, 10.2 mmol, 1.10 equiv) and the solution was heated to 70 °C for 3 h. After cooling down to 25 °C, the formed precipitate was filtered; the remaining solution was reduced by half and filtered again (3x). The solid was then washed 3x with water, transferred to a flask and traces of solvent were removed under reduced pressure. The product 3-propan-2-yl-1*H*-quinoxalin-2-one (1.54 g, 8.16 mmol, 88% yield) was obtained as a white-yellow solid.

R_f = 0.53 (cyclohexane/ethyl acetate 1:1). ^{1}H NMR (400 MHz, DMSO-d$_6$, ppm) δ = 12.29 (bs, 1H, N*H*), 7.71 (dd, ^{3}J = 8.0 Hz, ^{4}J = 1.5 Hz, 1H, C*H*$_{ar}$), 7.48–7.44 (m, 1H, C*H*$_{ar}$), 7.28–7.24 (m, 2H, C*H*$_{ar}$), 3.46 (hept, ^{3}J = 6.8 Hz, 1H, C*H*(CH$_3$)$_2$), 1.21 (d, ^{3}J = 6.8 Hz, 6H, CH(C*H*$_3$)$_2$); ^{13}C NMR (100 MHz, DMSO-*d*$_6$, ppm) δ = 165.6 (1C, NHCO), 154.1 (1C, N*C*CO), 131.6 (1C, CH*C*$_{ar}$), 131.5 (1C, CH*C*$_{ar}$), 129.4 (1C, *C*H$_{ar}$), 128.2 (1C, *C*H$_{ar}$), 123.0 (1C, *C*H$_{ar}$), 115.1 (1C, *C*H$_{ar}$), 29.9 (1C, *C*H(CH$_3$)$_2$), 20.0 (2C, 2x *C*H$_3$); MS (EI, 70 eV, 90 °C), m/z (%): 189 (13) [M+1]$^+$, 188 (100) [M]$^+$, 173 (56), 160 (79), 159 (19), 145 (84), 92 (17). HRMS (EI, $C_{11}H_{12}O_1N_2$): Calcd 188.0944, Found 188.0946; IR (ATR, ṽ) = 2965 (w), 2894 (w), 2861 (w), 2833 (w), 2776 (w), 2720 (w), 1662 (vs), 1611 (w), 1598 (w), 1560 (s), 1503 (w), 1486 (w), 1462 (w), 1451 (w), 1432 (m), 1380 (w), 1350 (w), 1286 (w), 1136 (w), 1071 (s), 946 (m), 925 (w), 905 (s), 861 (w), 834 (w), 752 (vs), 724 (w), 663 (w), 632 (m) cm^{-1}.

Additional information on the chemical synthesis is available via Chemotion repository: https://doi.org/10.14272/reaction/SA-FUHFF-UHFFFADPSC-CWFPSKVBKA-UHFFFADPSC-NUHFF-NUHFF-NUHFF-ZZZ
Additional information on the analysis of the target compound is available via Chemotion repository:
https://doi.org/10.14272/CWFPSKVBKAPBPV-UHFFFAOYSA-N.1
The synthesis of this compound has been previously described and the NMR data corresponds with the literature [10].

3-(Trifluoromethyl)-1*H*-quinoxalin-2-one (S2d)

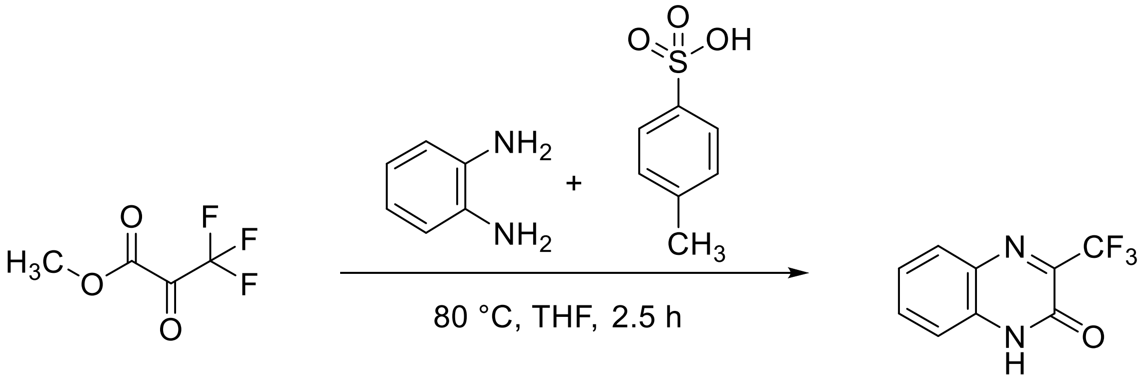

Name {P1|**S2d**}: 3-(trifluoromethyl)-1*H*-quinoxalin-2-one; Formula: $C_9H_5F_3N_2O$; Molecular Mass: 214.1440; Exact Mass: 214.0354; Smiles: O=c1[nH]c2ccccc2nc1C(F)(F)F; InChIKey: NOGLKXWLUDJZDQ-UHFFFAOYSA-N

To a solution of 1.40 g of 1,2-phenylenediamine (13.0 mmol, 1.2 equiv) in 20 mL of THF, 1.10 mL of methyl-3,3,3-trifluoro-2-oxopropanoate (1.68 g, 11.0 mmol, 1.0 equiv) and 0.19 g of *p*-TsOH (1.10 mmol, 0.10 equiv) was added. The solution was then heated to 80 °C for 2.5 h and subsequently stopped via addition of distilled water. The organic phase was separated and the aqueous phase was extracted 3x with DCM. The combined organic phases were dried over Na_2SO_4, filtered and the solvent was removed under reduced pressure. The crude product was purified via column chromatography (dryload on Celite, eluent *c*Hex/EtOAc 2:1) and 2.19 g (10.2 mmol, 95% yield) of a colourless solid were obtained.

R_f = 0.34 (cyclohexane/ethyl acetate 2:1). ^{1}H NMR (400 MHz, DMSO-d_6, ppm) δ = 13.05 (bs, 1H, N*H*), 7.91–7.89 (m, 1H, CH_{ar}N), 7.73–7.69 (m, 1H, CH_{ar}N), 7.42–7.38 (m, 2H, C_6H_4); ^{13}C NMR (100 MHz, DMSO-d_6, ppm) δ = 151.7 (1C, N*C*O), 144.0 (q, ^2J = 32.4 Hz, 1C, *C*CF$_3$), 133.7 (1C, N*C*C$_5$H$_4$), 133.5 (1C, *C*H$_{ar}$), 129.9 (1C, N*C*C$_5$H$_4$), 129.9 (1C, *C*H$_{ar}$), 124.1 (1C, *C*H$_{ar}$), 119.4 (q, ^1J = 276.2 Hz, 1C, *C*F$_3$), 115.8 (1C, *C*H$_{ar}$). ^{19}F NMR (376 MHz, DMSO-d_6, ppm) δ = -68.5; MS (EI, m/z, 70 eV, 90 °C): 215 (11) [M+H]+, 214 (100) [M]+, 186 (30), 166 (67), 90 (21). HRMS ($C_9H_5O_1N_2F_3$): calcd 214.0354, found 214.0353; IR (ATR, ṽ) = 2962, 2893, 2836, 2718, 1666, 1609, 1560, 1502, 1485, 1438, 1367, 1312, 1258, 1222, 1181, 1137, 1129, 1052, 1021, 994, 965, 925, 905, 841, 800, 764, 742, 725, 643, 591, 579, 557, 523, 482, 470, 460 cm^{-1}.

Additional information on the chemical synthesis is available via Chemotion repository: https://doi.org/10.14272/reaction/SA-FUHFF-UHFFFADPSC-NOGLKXWLUD-UHFFFADPSC-NUHFF-NUHFF-NUHFF-ZZZ
Additional information on the analysis of the target compound is available via Chemotion repository:
https://doi.org/10.14272/NOGLKXWLUDJZDQ-UHFFFAOYSA-N.1

The synthesis of this compound has been previously described [11].

3-Phenyl-1*H*-quinoxalin-2-one (S2e)

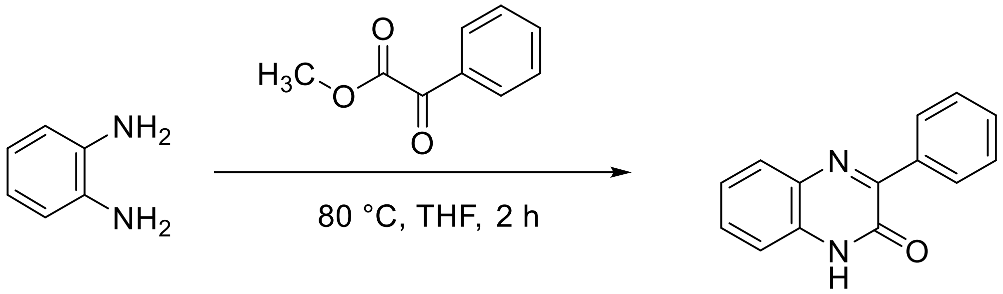

Name {P1|**S2e**}: 3-phenyl-1*H*-quinoxalin-2-one; Formula: $C_{14}H_{10}N_2O$; Molecular Mass: 222.2420 g/mol; Exact Mass: 222.0793 g/mol; Smiles: O=c1[nH]c2ccccc2nc1c1ccccc1; InChIKey: ZBBQSGVRBQKLLE-UHFFFAOYSA-N

To a solution of benzene-1,2-diamine (1.00 g, 9.2 mmol, 1.00 equiv) in THF (20.0 mL) was added methyl 2-oxo-2-phenylacetate (1.67 g, 1.44 mL, 10 mmol, 1.10 equiv). The solution was then heated to reflux for 2 h. The solution is allowed to reach 21 °C. The formed precipitate was then filtrate. The remaining solution was reduced by half and filtered again (2x). The solid was then washed with methylene chloride, dried under high vacuum and 3-phenyl-1*H*-quinoxalin-2-one was obtained in 97% yield (1.989 g, 8.950 mmol).

^{1}H NMR (400 MHz, DMSO-d_6, ppm) δ = 7.33 (m, 2H), 7.63–7.41 (m, 4H), 7.84 (m, 1H), 8.41–8.20 (m, 2H), 12.57 (s, 1H, -NH); ^{13}C NMR (100 MHz, DMSO-d_6, ppm) δ = 115.1, 123.4, 127.8 (2C), 128.8, 129.2 (2C), 130.2, 130.3, 132.0, 132.1, 135.6, 154.1, 154.6; EI (m/z, 70 eV, 140 °C): 223 (11) $[M+H]^+$, 222 (62) [M+], 195 (16), 194 (100), 193 (19), 90 (12), 63 (11). HRMS–EI (m/z): [M]+ Calcd for $C_{14}H_{10}O_1N_2$, 222.0793; Found, 222.0791.; IR (ATR, ṽ) = 401, 422, 469, 526, 551, 588, 616, 632, 687, 732, 755, 764, 806, 834, 850, 861, 905, 928, 948, 993, 1006, 1021, 1040, 1074, 1101, 1122, 1146, 1179, 1188, 1213, 1227, 1245, 1278, 1283, 1309, 1337, 1391, 1429, 1445, 1476, 1489, 1531, 1594, 1606, 1656, 1888, 1932, 1960, 2677, 2715, 2735, 2768, 2817, 2876, 2936, 2959, 2975, 2992, 3054, 3071, 3091, 3150, 3305 cm^{-1}.

Additional information on the chemical synthesis is available via Chemotion repository: https://dx.doi.org/10.14272/reaction/SA-FUHFF-UHFFFADPSC-ZBBQSGVRBQ-UHFFFADPSC-NUHFF-NUHFF-NUHFF-ZZZ

Additional information on the analysis of the target compound is available via Chemotion repository:
https://doi.org/10.14272/ZBBQSGVRBQKLLE-UHFFFAOYSA-N.1

The synthesis of this compound has been previously described [10].

1,4-Dihydroquinoxaline-2,3-dione (**S1**)

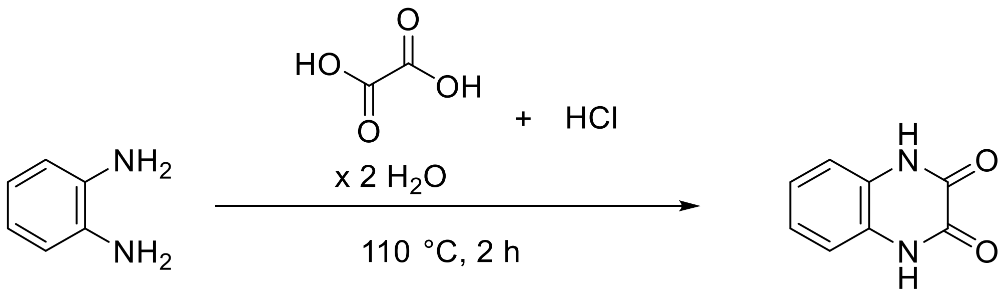

Name {P1|**S1**}: 1,4-dihydroquinoxaline-2,3-dione; Formula: $C_8H_6N_2O_2$; Molecular Mass: 162.1454; Exact Mass: 162.0429; Smiles: O=c1[nH]c2ccccc2[nH]c1=O, InChIKey: ABJFBJGGLJVMAQ-UHFFFAOYSA-N

The starting materials benzene-1,2-diamine (1.03 g, 9.50 mmol, 1.0 equiv) and 1.32 g of oxalic acid dihydrate (10.0 mmol, 1.1 equiv) were dissolved in 20 mL of 4 M aqueous HCl and stirred at 110 °C for 2 h. The reaction mixture was cooled to 25 °C. The resulting precipitate was isolated by filtration, washed with distilled water, and dried. The product 1,4-dihydroquinoxaline-2,3-dione (1.19 g, 7.32 mmol, 77% yield) was obtained in form of a colorless solid. Additional information: A yield of 92% was obtained when repeating the reaction.

R_f = 0.3 (DCM/Methanol 10:1). ^{1}H NMR (400 MHz, DMSO-d_6, ppm) δ = 11.90 (s, 2H, N*H*), 7.14–7.06 (m, 4H, C_6H_4); ^{13}C NMR (100 MHz, DMSO-d_6, ppm) δ = 155.1 (2C, NH*C*O), 125.6 (2C, N*C*$_2$C$_4$H$_4$), 123.0 (2C, *C*H$_{arom}$), 115.1 (2C, *C*H$_{arom}$); EI (m/z, 70 eV, 220 °C): 181 (21) $[M+H_2O+H]^+$, 162 (100) $[M]^+$, 134 (43), 131 (19), 106 (32), 105 (15), 79 (19), 69 (33). HRMS (EI, $C_8H_6O_2N_2$): Calcd 162.0429, Found 162.0430; IR (ATR, ṽ) = 3108, 3077, 3037, 3024, 2961, 2922, 2870, 2775, 2735, 2674, 1669, 1608, 1592, 1499, 1470, 1418, 1388, 1332, 1310, 1245, 1162, 1125, 1031, 943, 928, 898, 853, 751, 721, 700, 637, 578, 459, 391 cm^{-1}.

Additional information on the chemical synthesis is available via Chemotion repository:
https://doi.org/10.14272/reaction/SA-FUHFF-UHFFFADPSC-ABJFBJGGLJ-UHFFFADPSC-NUHFF-NUHFF-NUHFF-ZZZ
Additional information on the analysis of the target compound is available via Chemotion repository:
https://doi.org/10.14272/ABJFBJGGLJVMAQ-UHFFFAOYSA-N.1

The synthesis of this compound has been previously described in literature [12].

2-Chloroquinoxaline (**10a**)

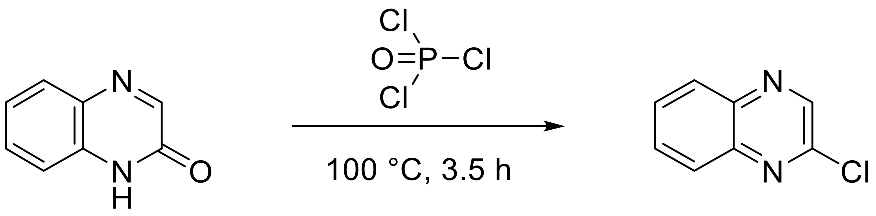

Name {P1|**10a**}: 2-chloroquinoxaline; Formula: $C_8H_5ClN_2$; Molecular Mass: 164.5917; Exact Mass: 164.0141; Smiles: Clc1cnc2c(n1)cccc2; InChIKey: BYHVGQHIAFURIL-UHFFFAOYSA-N

The staring material 1H-quinoxalin-2-one (2.00 g, 13.7 mmol, 1.00 equiv) was dissolved in phosphoryl chloride (41.0 g, 25.0 mL, 267 mmol, 19.5 equiv) and heated to 100 °C for 3.5 h. The reaction was cooled to room temperature, slowly poured on ice and rested for 16 h. The organic phase was separated and the aqueous phase was extracted with 3x DCM. The organic layers were combined, dried over Na_2SO_4 and filtered. The solvent was removed under reduced pressure. The crude product was purified via column chromatography (dryload on Celite, eluent *c*Hex/EtOAc 10:1) and 2-chloroquinoxaline (1.67 g, 10.1 mmol, 74% yield) was obtained as a white solid. Comment: The product 2-chloroquinoxaline was obtained in 82% yield when the reaction was repeated with a reaction time of 4.5 h.

R_f = 0.48 (cyclohexane/ethyl acetate 10:1). ^{1}H NMR (400 MHz, $CDCl_3$, ppm) δ = 8.77 (s, 1H, C*H*CCl), 8.11–8.09 (m, 1H, CH_{ar}), 8.02–7.99 (m, 1H, CH_{ar}), 7.81–7.74 (m, 2H, CH_{ar}); ^{13}C NMR (100 MHz, $CDCl_3$, ppm) δ = 147.3 (1C, C_q), 144.9 (1C, *C*HCCl), 141.9 (1C, C_q), 141.0 (1C, C_q), 131.2 (1C, *C*H_{ar}), 130.1 (1C, *C*H_{ar}), 129.3 (1C, *C*H_{ar}), 128.5 (1C, *C*H_{ar}); MS (EI, m/z, 70 eV, 20 °C): 164/166 $[M]^+$ (100/35), 129 (88), 102 (34), 76 (13), 75 (11). HRMS (EI, $C_8H_5N_2{}^{35}Cl_1$): calcd 164.0136, found 164.0136; IR (ATR, ṽ) = 3047 (m), 1541 (m), 1486 (m), 1459 (w), 1248 (m), 1153 (s), 1128 (m), 1091 (vs), 1057 (m), 958 (vs), 918 (s), 864 (m), 789 (w), 755 (vs), 708 (w), 594 (s), 518 (w), 448 (m), 408 (vs) cm^{-1}.

Additional information on the chemical synthesis is available via Chemotion repository: https://doi.org/10.14272/reaction/SA-FUHFF-UHFFFADPSC-BYHVGQHIAF-UHFFFADPSC-NUHFF-NUHFF-NUHFF-ZZZ.1
Additional information on the analysis of the target compound is available via Chemotion repository:
https://doi.org/10.14272/BYHVGQHIAFURIL-UHFFFAOYSA-N.2

The synthesis of this compound has been previously described and the NMR data corresponds with the literature [13].

2-Chloro-3-methylquinoxaline (10b)

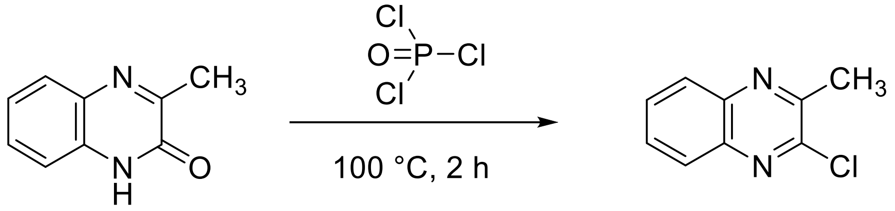

Name {P1|**10b**}: 2-chloro-3-methylquinoxaline; Formula: $C_9H_7ClN_2$; Molecular Mass: 178.6183; Exact Mass: 178.0298; Smiles: Cc1nc2ccccc2nc1Cl; InChIKey: PXDLUYLWPJMGJA-UHFFFAOYSA-N

To 4.30 g of 3-methylquinoxalin-2(1*H*)-one (27 mmol, 1.0 equiv), 55.0 mL of phosphoryl chloride (90.2 g, 0.59 mol, 21.9 equiv) were added and heated to 100 °C

for 2 h. The reaction was cooled to room temperature, poured on ice and was kept for 30 min on ice. The organic phase was separated and the aqueous phase was extracted with 3x DCM. The organic layers were combined, dried over Na_2SO_4 and filtered. The solvent was removed under reduced pressure. The crude product was purified via flash chromatography (dryload on Celite, eluent *c*Hex/EtOAc 10:1). The product was obtained as a red solid (4.21 g, 24 mmol, 88% yield).

R_f = 0.28 (cyclohexane/ethyl acetate 1:1). ^{1}H NMR (400 MHz, $CDCl_3$, ppm) δ = 8.04–8.01 (m, 1H, C_6H_4), 8.00–7.97 (m, 1H, C_6H_4), 7.76–7.70 (m, 2H, C_6H_4), 2.84 (s, 3H, CH_3); ^{13}C NMR (400 MHz, $CDCl_3$, ppm) δ = 152.8 (1C, *C*NCl), 147.8 (1C, *C*CH_3), 140.9 (1C, N*C*C_5H_4), 140.9 (1C, N*C*C_5H_4), 130.1 (1C, *C*H_{arom}), 130.0 (1C, *C*H_{arom}), 128.4 (1C, *C*H_{arom}), 128.1 (1C, *C*H_{arom}), 23.3 (1C, *C*H_3); ESI: 179/181 (100/33) $[M]^+$, 180/182 (10/3) $[M+1]^+$. HRMS [ESI, $C_9H_7ClN_2$+H]+: calcd 179.0371, found 179.0371; IR (ATR, ṽ) = 3055 (w), 1555 (w), 1482 (m), 1466 (w), 1428 (m), 1383 (m), 1341 (w), 1317 (w), 1288 (m), 1245 (w), 1204 (m), 1143 (m), 1125 (m), 1033 (vs), 1001 (s), 986 (m), 962 (s), 894 (m), 805 (m), 789 (m), 754 (vs), 703 (s), 684 (s), 591 (s), 540 (m), 462 (m), 428 (vs) cm^{-1}.

Additional information on the chemical synthesis is available via Chemotion repository: https://doi.org/10.14272/reaction/SA-FUHFF-UHFFFADPSC-PXDLUYLWPJ-UHFFFADPSC-NUHFF-NUHFF-NUHFF-ZZZ
Additional information on the analysis of the target compound is available via Chemotion repository:
https://doi.org/10.14272/PXDLUYLWPJMGJA-UHFFFAOYSA-N.1

The synthesis of this compound has been previously described in literature [14].

2-Chloro-3-propan-2-ylquinoxaline (10c)

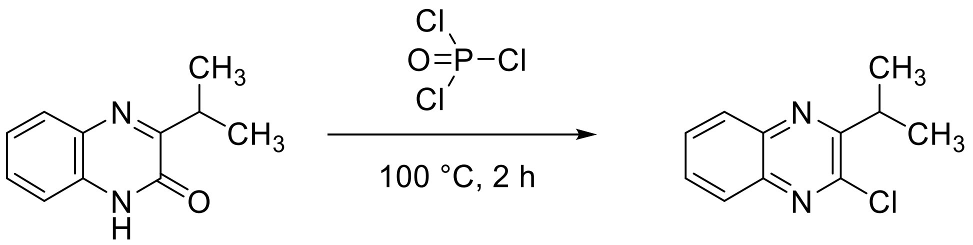

Name {P1|**10c**}: 2-chloro-3-propan-2-ylquinoxaline; Formula: $C_{11}H_{11}ClN_2$; Molecular Mass: 206.6714; Exact Mass: 206.0611; Smiles: CC(c1nc2ccccc2nc1Cl)C; InChIKey: ZRDHYUMEEXJHJN-UHFFFAOYSA-N

The starting material 3-propan-2-yl-1H-quinoxalin-2-one (363 mg, 1.93 mmol, 1.00 equiv) was dissolved in phosphoryl chloride (6.56 g, 4.00 mL, 42.8 mmol, 22.2 equiv) and heated to 100 °C for 2 h. The reaction was cooled to 25 °C, slowly poured on ice and rested for 1 h. The organic phase was separated and the aqueous phase was extracted with 3x DCM. The organic layers were combined, dried over Na_2SO_4 and filtered, the solvent was removed under reduced pressure. The crude product was purified via column chromatography (dryload on Celite, eluent *c*Hex/EtOAc 20:1); 2-

Chloro-3-propan-2-ylquinoxaline (320 mg, 1.55 mmol, 80% yield) was obtained as a colorless solid.

R_f = 0.32 (cyclohexane/ethyl acetate 20:1). ^{1}H NMR (400 MHz, $CDCl_3$, ppm) δ = 8.07–8.05 (m, 1H, CH_{ar}), 7.99–7.96 (m, 1H, CH_{ar}), 7.75–7.68 (m, 2H, CH_{ar}), 3.71 (hept, ^3J = 6.7 Hz, 1H, CH(CH_3)$_2$), 1.41 (d, ^3J = 6.7 Hz, 6H, 2x CH_3); ^{13}C NMR (100 MHz, $CDCl_3$, ppm) δ = 159.9 (1C, C_q), 147.3 (1C, C_q), 141.1 (1C, C_q), 140.7 (1C, C_q), 129.9 (1C, CH_{ar}), 129.8 (1C, CH_{ar}), 128.8 (1C, CH_{ar}), 128.0 (1C, CH_{ar}), 32.6 (1C, CH(CH_3)$_2$), 21.0 (2C, CH_3); MS (EI, 70 eV, 20 °C), m/z (%): 206/208 $[M]^+$ (48/17), 205 (16), 191/193 (100/33), 178 (36), 171 (25), 155 (17), 129 (47), 102 (41). HRMS (EI, $C_{11}H_{11}N_2{}^{35}Cl_1$): calcd 206.0605, found 206.0604; IR (ATR, ṽ) = 3044 (vw), 2965 (m), 2927 (w), 2868 (w), 1561 (w), 1550 (w), 1483 (w), 1466 (w), 1456 (m), 1442 (w), 1380 (w), 1358 (w), 1310 (w), 1265 (s), 1194 (w), 1180 (w), 1157 (m), 1130 (m), 1116 (m), 1092 (vs), 1018 (vs), 965 (m), 926 (w), 902 (w), 874 (w), 798 (w), 764 (vs), 687 (m), 649 (w), 616 (vw), 592 (s), 494 (w), 459 (m), 436 (w), 412 (w) cm^{-1}.

Additional information on the chemical synthesis is available via Chemotion repository: https://doi.org/10.14272/reaction/SA-FUHFF-UHFFFADPSC-ZRDHYUMEEX-UHFFFADPSC-NUHFF-NUHFF-NUHFF-ZZZ
Additional information on the analysis of the target compound is available via Chemotion repository:
https://doi.org/10.14272/ZRDHYUMEEXJHJN-UHFFFAOYSA-N.1

The synthesis of this compound has been previously described in literature [15].

2-Chloro-3-(trifluoromethyl)quinoxaline (10d)

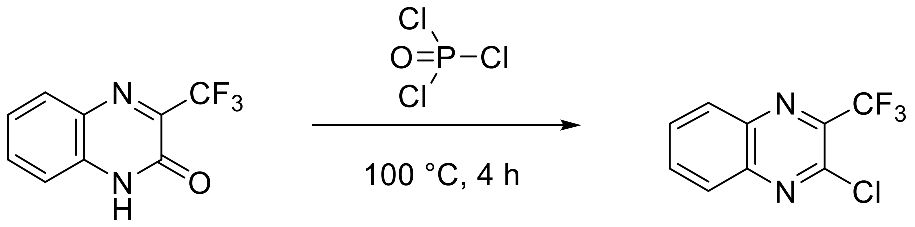

Name {P1|**10d**}: 2-chloro-3-(trifluoromethyl)quinoxaline; Formula: $C_9H_4ClF_3N_2$; Molecular Mass: 232.5897; Exact Mass: 232.0015; Smiles: Clc1nc2ccccc2nc1C(F)(F)F; InChIKey: DSMMAQWRRJQVTQ-UHFFFAOYSA-N

To 1.10 g of 3-(trifluoromethyl)quinoxalin-2(1*H*)-one (5.10 mmol, 1.0 equiv), 10.0 mL of phosphoryl chloride (16.4 g, 0.10 mol, 21 equiv) were added and the reaction mixture was heated to 100 °C for 4 h. The reaction was cooled to room temperature, poured on ice and rested for 30 min. The aqueous phase 3x with DCM, the organic layers were combined, dried over Na_2SO_4 and filtered. The solvent was removed under reduced pressure. The remaining solid was purified by column chromatography (dryload on Celite, eluent *c*Hex/EtOAc 4:1) and the product 2-chloro-3-(trifluoromethyl)quinoxaline (1.04 g, 4.49 mmol, 87% yield) was obtained as a colorless solid. Note: This reaction was repeated with a yield of 91%.

R_f = 0.51 (cyclohexane/ethyl acetate 10:1). ^{1}H NMR (400 MHz, ppm) δ = 8.25–8.22 (m, 1H, C*H*aromN), 8.13–8.11 (m, 1H, C*H*aromN), 7.98–7.89 (m, 2H, C*H*arom); ^{13}C NMR (100 MHz, $CDCl_3$, ppm) δ = 143.3 (1C, N*C*Cl), 142.7 (1C, N*C*C_5H_4), 140.2 (q, ^2J = 36.2 Hz, 1C, *C*CF_3), 138.8 (1C, N*C*C_5H_4), 133.6 (1C, *C*Harom), 131.5 (1C, *C*Harom), 129.9 (1C, *C*Harom), 128.3 (1C, *C*Harom), 120.3 (q, ^1J = 275.5 Hz, 1C, *C*F_3); ^{13}C NMR (100 MHz, $CDCl_3$, ppm) δ = 133.6 (1C, *C*Harom), 131.5 (1C, *C*Harom), 129.9 (1C, *C*Harom), 128.3 (1C, *C*Harom); ^{19}F NMR (376 MHz, $CDCl_3$, ppm) δ = -66.7; ESI: 233/235 (100/32) $[M+1]^+$, 234 (10), 231 (25). HRMS $(C_9H_6ClF_3N_2+H)^+$: calcd 233.0088, found 233.0086; IR (ATR, ṽ) = 3044, 1561, 1547, 1487, 1468, 1394, 1364, 1344, 1305, 1293, 1256, 1244, 1181, 1166, 1132, 1109, 1030, 997, 979, 945, 898, 884, 798, 773, 744, 650, 605, 589, 575, 527, 499, 458 cm^{-1}.

Additional information on the chemical synthesis is available via Chemotion repository: https://doi.org/10.14272/reaction/SA-FUHFF-UHFFFADPSC-DSMMAQWRRJ-UHFFFADPSC-NUHFF-NUHFF-NUHFF-ZZZ
Additional information on the analysis of the target compound is available via Chemotion repository:
https://doi.org/10.14272/DSMMAQWRRJQVTQ-UHFFFAOYSA-N.1

The synthesis of this compound has been previously described and the NMR data corresponds with the literature [16].

2-Chloro-3-phenylquinoxaline (10e)

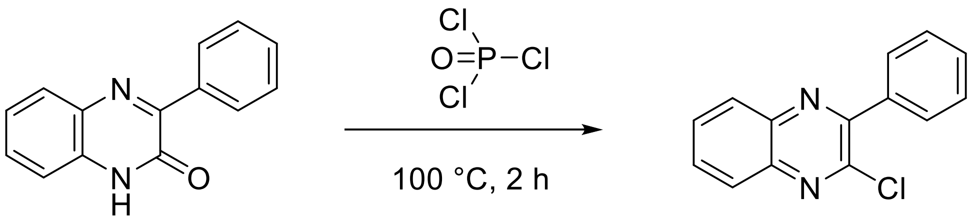

Name {P1|**10e**}: 2-chloro-3-phenylquinoxaline; Formula: $C_{14}H_9ClN_2$; Molecular Mass: 240.6877; Exact Mass: 240.0454; Smiles: Clc1nc2ccccc2nc1c1ccccc1; InChIKey: KPGPIQKEKAEAHM-UHFFFAOYSA-N

In a 100 mL pear-shaped flask, phosphoryl chloride (46.4 mL, 496.8 mmol, 60 eq), was added to 3-phenylquinoxalin-2(1H)-one (1840 mg, 8.3 mmol, 1 eq) and heated to 100 °C for 2 h. The reaction was cooled to 21 °C and poured on ice and rested for overnight. The remaining aqueous layer was extracted with DCM (3x) and the organic layers were combined, dried over Na_2SO_4 and concentrated under reduced pressure. The crude product was purified by column chromatography on silica gel using Cyclohexane/EtOAc (10:1) as the eluent to afford 2-chloro-3-phenylquinoxaline (1598 mg, 6.6 mmol, 80% yield) as a white solid.

R_f = 0.49 (cyclohexane/ethyl acetate 10:1). ^{1}H NMR (400 MHz, $CDCl_3$, ppm) δ = 7.59–7.48 (m, 3H), 7.83–7.77 (m, 2H), 7.90–7.84 (m, 2H), 8.11–8.03 (m, 1H), 8.20–8.12 (m, 1H); ^{13}C NMR (100 MHz, $CDCl_3$, ppm) δ = 128.3, 128.5 (2C), 129.4, 129.8 (2C), 129.9, 130.6, 131.0, 136.9, 141.2, 141.2, 146.3, 153.2; EI (m/z, 70 eV, 40 °C): 242/241/240 [M]+ (21/10/61), 206 (16), 205 (100), 102 (25), 77 (36), 76 (16), 75 (14), 51 (16) HRMS $(C_{14}H_9N_2Cl_1)$: calcd 240.0454, found 240.0454; IR (ATR, ṽ) = 436, 483, 492, 552, 575, 597, 633, 685, 695, 719, 762, 876, 885, 910, 978, 1001, 1029, 1086, 1133, 1147.39,

1158, 1219.63, 1243, 1276, 1297, 1332, 1385, 1443, 1460, 1481, 1497, 1535, 1559, 1610, 3034, 3061 cm^{-1}.

Additional information on the chemical synthesis is available via Chemotion repository: https://doi.org/10.14272/reaction/SA-FUHFF-UHFFFADPSC-KPGPIQKEKA-UHFFFADPSC-NUHFF-NUHFF-NUHFF-ZZZ
Additional information on the analysis of the target compound is available via Chemotion repository:
https://doi.org/10.14272/KPGPIQKEKAEAHM-UHFFFAOYSA-N.1

The synthesis of this compound has been previously described in literature [17].

2,3-Dichloroquinoxaline (10f)

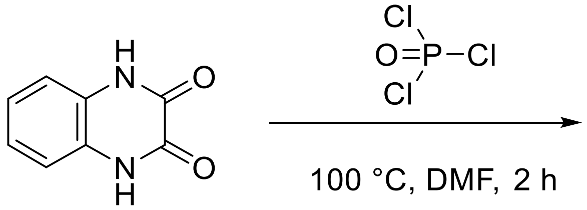

Name {P1|**10f**}: 2,3-dichloroquinoxaline; Formula: $C_8H_4Cl_2N_2$; Molecular Mass: 199.0368; Exact Mass: 197.9752; Smiles: Clc1nc2ccccc2nc1Cl; InChIKey: SPSSDDOTEZKOOV-UHFFFAOYSA-N

Phosphoryl chloride (21.6 g, 13.1 mL, 141 mmol, 20.0 equiv) and 5 mL of DMF were added to the quinoxalinone (1.14 g, 7.0 mmol, 1.00 equiv) and heated to 100 °C for 2 h. The reaction was cooled to 21 °C, poured on ice and rested overnight. The organic phase was separated and the aqueous phase was extracted 3x with ethyl acetate; the combined organic layers were combined were dried over Na_2SO_4, filtered and the solvent was removed under reduced pressure. The remaining solid was purified by column chromatography (*c*Hex/ethyl acetate 10:1). 1.32 g (6.65 mmol, 95%) of a colorless solid were obtained.

R_f = 0.59 (cyclohexane/ethyl acetate 4:1). ^{1}H NMR (400 MHz, $CDCl_3$, ppm) δ = 8.07–8.02 (m, 2H, CH_{ar}), 7.84–7.80 (m, 2H, CH_{ar}); ^{13}C NMR (100 MHz, $CDCl_3$, ppm) δ = 145.4 (2C, $C_2N_2Cl_2$), 140.6 (2C, C_{ar}), 131.3 (2C, C_{ar}), 128.3 (2C, C_{ar}); EI (m/z, 70 eV, 20 °C): 200/198 (66/100) $[M]^+$, 165 (21), 163 (65), 102 (46); HRMS (EI, $C_8H_4N_2{}^{35}Cl_2$): calcd 197.9752, found 197.9752; IR (ATR, ṽ) = 3104, 3063, 3041, 3002, 2944, 1955, 1846, 1645, 1608, 1555, 1530, 1482, 1458, 1343, 1266, 1242, 1176, 1116, 1069, 1018, 1006, 987, 969, 885, 785, 764, 647, 596, 558, 524, 500, 492, 477, 456, 435, 377 cm^{-1}.

Additional information on the chemical synthesis is available via Chemotion repository: https://doi.org/10.14272/reaction/SA-FUHFF-UHFFFADPSC-SPSSDDOTEZ-UHFFFADPSC-NUHFF-NUHFF-NUHFF-ZZZ.1
Additional information on the analysis of the target compound is available via Chemotion repository:
https://doi.org/10.14272/SPSSDDOTEZKOOV-UHFFFAOYSA-N.2

The synthesis of this compound has been previously described and the NMR data corresponds with the literature [13].

Tetrazolo[1,5-*a*]quinoxaline (**11a**)

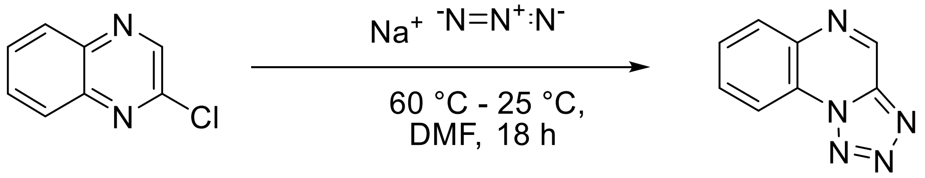

Name {P1|**11a**}: tetrazolo[1,5-*a*]quinoxaline; Formula: $C_8H_5N_5$; Molecular Mass: 171.1588; Exact Mass: 171.0545; Smiles: c1ccc2c(c1)n1nnnc1cn2; InChIKey: LGMVEBQKPYIMMI-UHFFFAOYSA-N

Sodium azide (434 mg, 6.68 mmol, 1.08 equiv) was added to the starting material 2-chloroquinoxaline (1.01 g, 6.16 mmol, 1.00 equiv) in 15 ml of DMF, stirred at 60 °C for 2.5 h and subsequently stirred at 25 °C for 16 h. Water was added and the aqueous phase was extracted with 3× EtOAc. The combined organic phases were dried over Na_2SO_4, filtered and the solvent was removed under reduced pressure. The crude product was purified via column chromatography (dryload on celite, eluent *c*Hex/EtOAc 1:1) and tetrazolo[1,5-*a*]quinoxaline (960 mg, 5.61 mmol, 91% yield) was obtained as a light yellow solid.

R_f = 0.2 (cyclohexane/ethyl acetate 4:1). ^{1}H NMR (400 MHz, $CDCl_3$, ppm) δ = 9.57 (s, 1H, NC*H*CNN), 8.65 (dd, ^3J = 8.2 Hz, ^4J = 1.3 Hz, 1H, CH_{ar}CN), 8.32 (dd, ^3J = 8.1 Hz, ^4J = 1.3 Hz, 1H, CH_{ar}CN), 7.94 (td, ^3J = 7.9 Hz, ^4J = 1.4 Hz, 1H, CH_{ar}CHCN), 7.88 (td, ^3J = 7.8 Hz, ^4J = 1.5 Hz, 1H, CH_{ar}CHCN); ^{13}C NMR (100 MHz, $CDCl_3$ [77.0 ppm], ppm) δ = 142.3 (1C, N*C*N), 141.2 (1C, N*C*HCNN), 136.7 (1C, CHC_qN), 131.7 (1C, CH_{ar}), 130.8 (1C, CH_{ar}), 129.8 (1C, CH_{ar}), 125.0 (1C, CHC_{ar}N), 116.4 (1C, CH_{ar}); MS (EI, 70 eV, 50 °C), m/z (%): 171 $[M]^+$ (6), 144 (10), 143 (100), 116 (32), 89 (10), 63 (11). HRMS (EI, $C_8H_5N_5$): Calcd 171.0539, Found 171.0540; IR (ATR, ṽ) = 3063 (w), 3040 (vw), 3021 (w), 1551 (w), 1509 (w), 1458 (w), 1443 (w), 1408 (w), 1354 (w), 1329 (w), 1310 (w), 1269 (w), 1222 (w), 1193 (w), 1140 (w), 1102 (w), 1078 (s), 1035 (w), 1018 (w), 997 (m), 963 (w), 916 (s), 871 (s), 843 (w), 782 (vs), 766 (vs), 713 (w), 701 (w), 690 (m), 620 (m), 579 (w), 520 (w), 469 (w), 453 (m), 429 (vs) cm^{-1}.

Additional information on the chemical synthesis is available via Chemotion repository: https://doi.org/10.14272/reaction/SA-FUHFF-UHFFFADPSC-LGMVEBQKPY-UHFFFADPSC-NUHFF-NUHFF-NUHFF-ZZZ.1

Additional information on the analysis of the target compound is available via Chemotion repository: https://doi.org/10.14272/LGMVEBQKPYIMMI-UHFFFAOYSA-N.2

The synthesis of this compound has been previously described and the NMR data corresponds with the literature [6].

4-Methyltetrazolo[1,5-*a*]quinoxaline (**11b**)

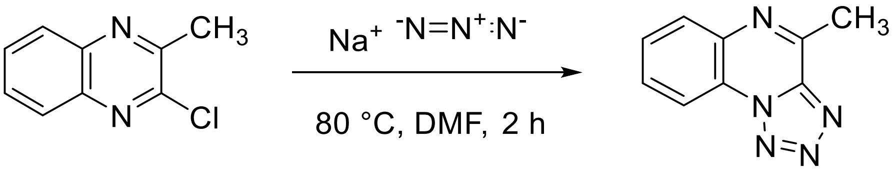

Name {P1|**11b**}: 4-methyltetrazolo[1,5-*a*]quinoxaline; Formula: $C_9H_7N_5$; Molecular Mass: 185.1854; Exact Mass: 185.0701; Smiles: Cc1nc2ccccc2n2c1nnn2; InChIKey: BVEPTXWZDNARLY-UHFFFAOYSA-N

Sodium azide (0.55 g, 8.40 mmol, 1.5 equiv) was added to 1.0 g of 2-chloro-3-methylquinoxaline (5.60 mmol, 1.0 equiv) in 25 mL of DMF and stirred at 80 °C for 2 h. Distilled water was added and the organic phase was separated. The aqueous phase was extracted 3× with EtOAc, the combined organic phases were dried over Na_2SO_4 and filtered. The solvent was removed under reduced pressure. The crude product was purified via column chromatography (dryload on Celite, eluent *c*Hex/EtOAc 10:1). The product was obtained as a yellow solid (0.98 g, 5.26 mmol, 94% yield).

R_f = 0.22 (cyclohexane/ethyl acetate 4:1). ^{1}H NMR (400 MHz, $CDCl_3$, ppm) δ = 8.59–8.57 (m, 1H, C_6H_4), 8.22–8.20 (m, 1H, C_6H_4), 7.87–7.80 (m, 2H, C_6H_4), 3.13 (s, 3H, CH_3); ^{13}C NMR (100 MHz, $CDCl_3$, ppm) δ = 151.0 (1C, N*C*N), 142.9 (1C, N*C*C_5H_4), 136.8 (1C, N*C*C_5H_4), 130.3 (1C, CH_{arom}), 129.8 (1C, CH_{arom}), 129.7 (1C, CH_{arom}), 124.6 (1C, *C*CH_3), 116.3 (1C, CH_{arom}), 21.7 (1C, CH_3); MS (ESI): 187 (10) $[M+2]^+$, 186 (100) $[M+1]^+$, 158 (16). HRMS [ESI, $C_9H_7N_5+H]^+$: Calcd 186.0774, Found 186.0774. IR (ATR, ṽ) = 3081 (w), 3016 (w), 1510 (m), 1477 (m), 1446 (w), 1428 (m), 1411 (m), 1373 (m), 1337 (s), 1290 (w), 1255 (w), 1218 (w), 1177 (s), 1154 (w), 1102 (m), 1055 (w), 1017 (w), 993 (s), 970 (m), 854 (s), 781 (s), 768 (vs), 730 (w), 697 (m), 650 (m), 618 (m), 543 (w), 534 (w), 469 (m), 460 (m), 446 (m) cm^{-1}.

Additional information on the chemical synthesis is available via Chemotion repository: https://doi.org/10.14272/reaction/SA-FUHFF-UHFFFADPSC-BVEPTXWZDN-UHFFFADPSC-NUHFF-NUHFF-NUHFF-ZZZ

Additional information on the analysis of the target compound is available via Chemotion repository: https://doi.org/10.14272/BVEPTXWZDNARLY-UHFFFAOYSA-N.1

The synthesis of this compound has been previously described and the ^{1}H NMR data corresponds with the literature [18].

4-Isopropyltetrazolo[1,5-*a*]quinoxaline (**11c**)

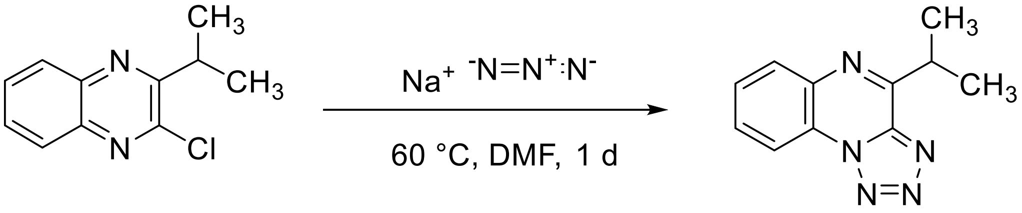

Name {P1|**11c**}: 4-isopropyltetrazolo[1,5-*a*]quinoxaline; Formula: $C_{11}H_{11}N_5$; Molecular Mass: 213.2385; Exact Mass: 213.1014; Smiles: CC(c1nc2ccccc2n2c1nnn2)C; InChIKey: UEHBPGWWVJRPTJ-UHFFFAOYSA-N

Sodium azide (97.6 mg, 1.50 mmol, 1.10 equiv) was added to the starting material 2-chloro-3-propan-2-ylquinoxaline (282 mg, 1.36 mmol, 1.00 equiv) in 5 mL of DMF and stirred at 60 °C for 26 h. Water was added and the aqueous phase was extracted with 3× EtOAc. The combined organic phases were dried over Na_2SO_4, filtered and the solvent was removed under reduced pressure. The crude product was purified via column chromatography (dryload on celite, *c*Hex -> *c*Hex/EtOAc 1:1) and 4-isopropyltetrazolo[1,5-*a*]quinoxaline (202 mg, 947 µmol, 69% yield) was obtained as a colorless solid.

R_f = 0.19 (cyclohexane/ethyl acetate 2:1). ^{1}H NMR (400 MHz, DMSO-d_6, ppm) δ = 8.54 (dd, ^3J = 7.8 Hz, ^4J = 1.9 Hz, 1H, CH_{ar}), 8.23 (dd, ^3J = 8.0 Hz, ^4J = 1.7 Hz, 1H, CH_{ar}), 7.94–7.86 (m, 2H, CH_{ar}), 3.80 (hept, ^3J = 6.9 Hz, 1H, CH(CH_3)$_2$), 1.49 (d, ^3J = 7.0 Hz, 6H, CH_3); ^{13}C NMR (100 MHz, DMSO-d_6,) δ = 158.2 (1C, C_q), 142.0 (1C, C_q), 136.2 (1C, C_q), 130.4 (1C, CH$_{ar}$), 129.6 (1C, CH$_{ar}$), 129.5 (1C, CH$_{ar}$), 124.3 (1C, C_q), 116.0 (1C, CH$_{ar}$), 33.5 (1C, CH(CH_3)$_2$), 20.2 (2C, CH$_3$); MS (EI, 70 eV, 50 °C), m/z (%): 213 [M]$^+$ (6), 185 (29), 184 (61), 170 (100), 157 (15), 145 (80), 143 (53), 131 (20), 116 (31), 90 (21). HRMS (EI, $C_{11}H_{11}N_5$): Calcd 213.1009, Found 213.1011; IR (ATR, ṽ) = 2975 (w), 2921 (w), 2876 (w), 1561 (vw), 1506 (s), 1465 (m), 1459 (m), 1422 (w), 1412 (w), 1383 (w), 1360 (vw), 1339 (w), 1324 (w), 1248 (vw), 1217 (vw), 1183 (vw), 1140 (w), 1133 (w), 1094 (m), 1072 (m), 994 (w), 984 (w), 959 (w), 897 (w), 766 (vs), 703 (w), 680 (vw), 659 (w), 632 (vw), 613 (vw), 552 (vw), 479 (w), 455 (w) cm^{-1}.

Additional information on the chemical synthesis is available via Chemotion repository: https://doi.org/10.14272/reaction/SA-FUHFF-UHFFFADPSC-UEHBPGWWVJ-UHFFFADPSC-NUHFF-NUHFF-NUHFF-ZZZ
Additional information on the analysis of the target compound is available via Chemotion repository:
https://doi.org/10.14272/UEHBPGWWVJRPTJ-UHFFFAOYSA-N.1

4-(Trifluoromethyl)tetrazolo[1,5-*a*]quinoxaline (**11d**)

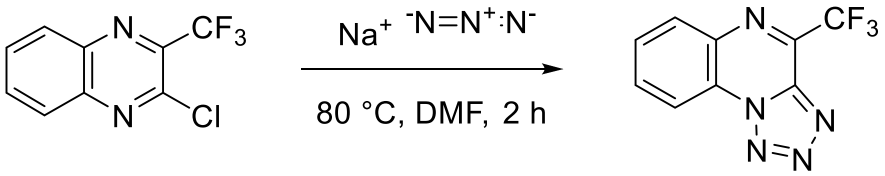

Name {P1|**11d**}: 4-(trifluoromethyl)tetrazolo[1,5-*a*]quinoxaline; Formula: $C_9H_4F_3N_5$; Molecular Mass: 239.1568; Exact Mass: 239.0419; Smiles: FC(c1nc2ccccc2n2c1nnn2)(F)F; InChIKey: ALUDOBBDYFOFJP-UHFFFAOYSA-N

Sodium azide (0.40 g, 6.10 mmol, 1.5 equiv) was added to 0.95 g of 2-chloro-3-(trifluoromethyl)quinoxaline (4.10 mmol, 1.0 equiv) in 25 mL of DMF and stirred at 80 °C for 2 h. Distilled water was added, the organic phase was separated and the aqueous phase was extracted 3× with EtOAc. The combined organic phases were dried over Na_2SO_4, filtered and the solvent was removed under reduced pressure. The crude product was purified via column chromatography (dryload on Celite, eluent *c*Hex/EtOAc 10:1) and 0.93 g (3.87 mmol, 95% yield) of a colourless solid were obtained.

R_f = 0.32 (cyclohexane/ethyl acetate 2:1). ^{1}H NMR (400 MHz, $CDCl_3$, ppm) δ = 8.74 (dd, ^3J = 8.3 Hz, ^2J = 1.1 Hz, 1H, CH_{ar}N), 8.46 (dd, ^3J = 8.2 Hz, ^2J = 1.1 Hz, 1H, CH_{ar}N), 8.13–8.08 (m, 1H, CH_{ar}), 8.00 (ddd, ^3J = 8.6 Hz, ^3J = 7.4 Hz, ^2J = 1.3 Hz, 1H, CH_{ar}); ^{13}C NMR (100 MHz, $CDCl_3$ [77.0 ppm], ppm) δ = 139.7 (1C, N*C*N), 138.9 (q, ^2J = 40.3 Hz, 1C, *C*CF_3), 134.9 (1C, N*C*C_5H_4), 134.1 (1C, *C*H_{ar}), 131.6 (1C, *C*H_{ar}), 130.7 (1C, *C*H_{ar}), 125.6 (1C, N*C*C_5H_4), 119.3 (q, ^1J = 276 Hz, 1C, *C*F_3), 116.6 (1C, *C*H_{ar}); ^{19}F NMR (376 MHz, $CDCl_3$, ppm) δ = – 67.7; ESI: 241 (10) $[M+1]^+$, 240 (100), 231 (13), 212 (33). HRMS $[C_9H_4F_3N_5+H]^+$: Calcd 240.0492, Found 240.0488; IR (ATR, ṽ) = 3091 (w), 1571 (w), 1517 (w), 1475 (w), 1417 (w), 1356 (s), 1299 (s), 1271 (w), 1262 (m), 1249 (w), 1213 (s), 1186 (vs), 1154 (vs), 1137 (vs), 1103 (vs), 1088 (vs), 993 (w), 969 (vs), 853 (w), 773 (vs), 732 (vs), 704 (m), 669 (w), 660 (w), 588 (s), 534 (w), 501 (w), 477 (s), 456 (m) cm^{-1}.

Additional information on the chemical synthesis is available via Chemotion repository: https://doi.org/10.14272/reaction/SA-FUHFF-UHFFFADPSC-ALUDOBBDYF-UHFFFADPSC-NUHFF-NUHFF-NUHFF-ZZZ
Additional information on the analysis of the target compound is available via Chemotion repository:
https://doi.org/10.14272/ALUDOBBDYFOFJP-UHFFFAOYSA-N.1

4-Phenyltetrazolo[1,5-*a*]quinoxaline (11e)

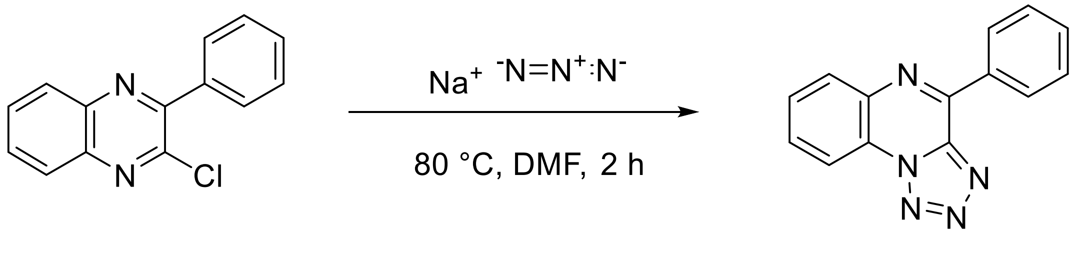

Name {P1|**11e**}: 4-phenyltetrazolo[1,5-*a*]quinoxaline; Formula: $C_{14}H_9N_5$; Molecular Mass: 247.2548; Exact Mass: 247.0858; Smiles: c1ccc(cc1)c1nc2ccccc2n2c1nnn2; InChIKey: AOUCPYFCRAWRNU-UHFFFAOYSA-N

The starting material 2-chloro-3-phenylquinoxaline (899 mg, 3.74 mmol, 1.00 equiv) was dissolved in 10 mL of DMF and sodium;azide (271 mg, 4.17 mmol, 1.12 equiv) was added; the reaction mixture was stirred at 80 °C for 2 h. The reaction mixture was cooled to 25 °C and distilled water was added, then the organic phase was separated and the aqueous phase was extracted 3× with EtOAc. The combined organic phases were dried over Na_2SO_4, filtered and the solvent was removed under reduced pressure. The crude product was purified via column chromatography (dryload on Celite, *c*Hex -> eluent *c*Hex/ethyl acetate 1:1) and 4-phenyltetrazolo[1,5-*a*]quinoxaline (865 mg, 3.50 mmol, 94% yield) was obtained as a colorless solid.

R_f = 0.77 (cyclohexane/ethyl acetate 2:1). ^{1}H NMR (400 MHz, $CDCl_3$, ppm) δ = 8.93–8.88 (m, 2H, CH_{Phenyl}), 8.63–8.59 (m, 1H, CH_{ar}), 8.31–8.27 (m, 1H, CH_{ar}), 7.87–7.81 (m, 2H, CH_{ar}), 7.63–7.60 (m, 3H, CH_{Phenyl}); ^{13}C NMR (100 MHz, $CDCl_3$ [77.0 ppm], ppm) δ = 148.0 (1C, C_q), 142.1 (1C, C_q), 136.8 (1C, C_q), 134.0 (1C, C_q), 132.1 (1C, CH_{Phenyl}), 130.6 (1C, CH_{ar}), 130.4 (1C, CH_{ar}), 130.0 (1C, CH_{ar}), 129.5 (2C, CH_{Phenyl}) , 128.9 (2C, CH_{Phenyl}), 124.2, 116.2 (1C, CH_{ar}); MS (EI, 70 eV, 120 °C), m/z (%): 247 $[M]^+$ (9), 220 (16), 219 (100), 218 (18), 91 (11). HRMS (EI, $C_{14}H_9N_5$): calcd 247.0852, found 247.0854; IR (ATR, ṽ) = 3063 (w), 2924 (w), 1506 (w), 1492 (m), 1465 (m), 1448 (m), 1438 (m), 1401 (w), 1343 (s), 1330 (m), 1285 (w), 1256 (w), 1239 (m), 1200 (m), 1181 (m), 1154 (w), 1139 (m), 1102 (m), 1082 (m), 1026 (w), 994 (m), 948 (s), 868 (w), 850 (w), 795 (w), 766 (vs), 728 (vs), 703 (s), 687 (vs), 670 (vs), 657 (s), 630 (vs), 574 (m), 493 (vs), 456 (m) cm^{-1}.

Additional information on the chemical synthesis is available via Chemotion repository: https://doi.org/10.14272/reaction/SA-FUHFF-UHFFFADPSC-AOUCPYFCRA-UHFFFADPSC-NUHFF-NUHFF-NUHFF-ZZZ

Additional information on the analysis of the target compound is available via Chemotion repository:
https://doi.org/10.14272/AOUCPYFCRAWRNU-UHFFFAOYSA-N.1

The use of this compound has been previously described in literature [19].

4-Chlorotetrazolo[1,5-*a*]quinoxaline (11f)

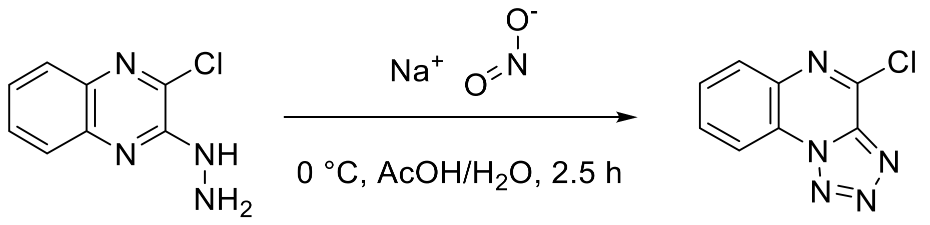

Name {P1|**11f**}: 4-chlorotetrazolo[1,5-*a*]quinoxaline; Formula: $C_8H_4ClN_5$; Molecular Mass: 205.6039; Exact Mass: 205.0155; Smiles: Clc1nc2ccccc2n2c1nnn2; InChIKey: KOWYBYDSUFDMGG-UHFFFAOYSA-N

Sodium nitrite (53 mg in in 0.5 mL of water, 771 µmol, 3.00 equiv) was added dropwise over a period of 30 min to 50.0 mg (257 µmol, 1.00 equiv) of (3-chloroquinoxalin-2-yl)hydrazine in a 3:1 mixture of acetic acid (1.5 mL) and water (0.5 mL) at 0 °C. The reaction was stirred at 0 °C for 2.5 h and subsequently neutralized with solid Na_2CO_3

while cooled in an ice bath to 0 °C. Ethyl acetate and water was added and the aqueous phase was extracted 3× with EtOAc. The combined organic phases were dried over Na_2SO_4 and the solvent was removed under reduced pressure. The crude product was purified via flash chromatography (dryload on Celite, eluent *c*Hex/EtOAc 4:1) and 4-chlorotetrazolo[1,5-*a*]quinoxaline (44.6 mg, 217 µmol, 84% yield) was obtained as a yellow-beige solid.

R_f = 0.43 (cyclohexane/ethyl acetate 4:1). ^{1}H NMR (400 MHz, ppm) δ = 8.62 (dd, ^3J = 8.2 Hz, ^4J = 1.2 Hz, 1H, C*H*NCl), 8.23 (dd, ^3J = 8.1 Hz, ^4J = 1.2 Hz, 1H, C*H*NN), 7.95 (td, ^3J = 7.8 Hz, ^4J = 1.5 Hz, 1H, C*H*$_{ar}$CH), 7.89 (td, ^3J = 7.8 Hz, ^4J = 1.5 Hz, 1H, C*H*$_{ar}$CH); ^{13}C NMR (100 MHz, $CDCl_3$ [77.0 ppm], ppm) δ = 142.1 (1C, C_q), 140.3 (1C, C_q), 136.0 (1C, C_q), 131.7 (1C, *C*H$_{ar}$CH), 130.5 (1C, *C*H$_{ar}$CH), 129.8 (1C, *C*H$_{ar}$NN), 124.6 (1C, C_q), 116.5 (1C, *C*H$_{ar}$NCl); EI (m/z, 70 eV, 80 °C): 205/207 $[M]^+$ (9/3), 179 (34), 177 (100), 142 (14), 116 (16), 90 (20), 89 (16), 63 (14). HRMS (EI, $C_8H_4N_5{}^{35}Cl_1$): calcd 205.0155, found 205.0155; IR (ATR, ṽ) = 3082 (w), 3023 (w), 1973 (w), 1846 (w), 1662 (w), 1609 (w), 1547 (w), 1506 (s), 1477 (w), 1456 (s), 1412 (w), 1322 (m), 1315 (m), 1217 (w), 1208 (w), 1142 (s), 1111 (m), 1098 (vs), 1045 (w), 975 (vs), 962 (s), 932 (w), 878 (w), 841 (w), 778 (vs), 701 (w), 694 (m), 639 (m) cm^{-1};

Additional information on the chemical synthesis is available via Chemotion repository: https://doi.org/10.14272/reaction/SA-FUHFF-UHFFFADPSC-KOWYBYDSUF-UHFFFADPSC-NUHFF-NUHFF-NUHFF-ZZZ

Additional information on the analysis of the target compound is available via Chemotion repository: https://doi.org/10.14272/KOWYBYDSUFDMGG-UHFFFAOYSA-N.1

The synthesis of this compound has been previously described in literature [7].

4-Methoxytetrazolo[1,5-*a*]quinoxaline (**11g**), **5*H*-tetrazolo[1,5-*a*]quinoxalin-4-one** (**11h**)

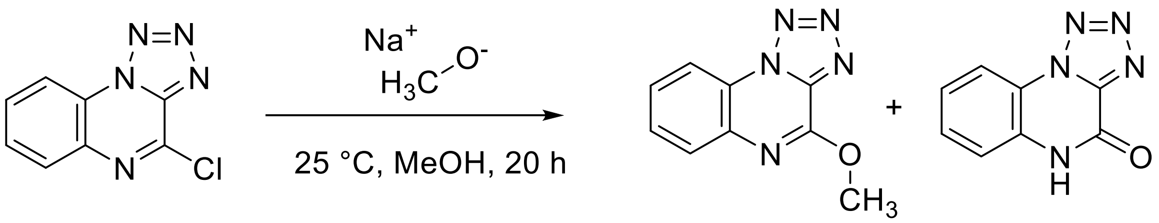

Name {P1|**11g**}: 4-methoxytetrazolo[1,5-*a*]quinoxaline; Formula: $C_9H_7N_5O$; Molecular Mass: 201.1848; Exact Mass: 201.0651; Smiles: COc1nc2ccccc2n2c1nnn2; InChIKey: VEKBUDDBMJNFNK-UHFFFAOYSA-N

Name {P2|**11h**}: 5*H*-tetrazolo[1,5-*a*]quinoxalin-4-one; Formula: $C_8H_5N_5O$; Molecular Mass: 187.1582; Exact Mass: 187.0494; Smiles: O=c1[nH]c2ccccc2n2c1nnn2; InChIKey: SECOVEPJEIBBPV-UHFFFAOYSA-N

The starting material 4-chlorotetrazolo[1,5-a]quinoxaline (90.0 mg, 438 µmol, 1.00 equiv) and sodium;methanolate (68.0 mg, 1.26 mmol, 2.88 equiv) were added to a crimp vial and methanol (2.00 mL) was added. The reaction mixture was stirred at 25 °C for 20 h, then water and EtOAc were added and the aqueous phase was extracted

3× with EtOAc. The combined organic phases were dried over Na_2SO_4, filtered and the solvent was removed under reduced pressure. The crude product was purified via column chromatography (dryload on Celite, eluent *c*Hex/EtOAc 4:1); 4-methoxytetrazolo[1,5-*a*]quinoxaline (64.0 mg, 318 µmol, 73% yield) and 5*H*-tetrazolo[1,5-*a*]quinoxalin-4-one (12.0 mg, 64.1 µmol, 15% yield) were obtained as colourless solids.

R_f = 0.36 (cyclohexane/ethyl acetate 2:1). ^{1}H NMR (400 MHz, DMSO-d_6, ppm) δ = 8.46–8.44 (m, 1H, C*H*ar), 7.99–7.97 (m, 1H, C*H*ar), 7.82–7.73 (m, 2H, C*H*ar), 4.25 (s, 3H, C*H*3); ^{13}C NMR (100 MHz, DMSO-d_6, ppm) δ = 151.3 (1C, *C*q), 138.9 (1C, *C*q), 135.2 (1C, *C*q), 129.8 (1C, *C*Har), 127.8 (1C, *C*Har), 127.6 (1C, *C*Har), 123.6 (1C, *C*q), 116.0 (1C, *C*Har), 54.9 (1C, *C*H3); MS (EI, 70 eV, 90 °C), m/z (%): 201 $[M]^+$ (10), 173 (60), 158 (100), 106 (49), 90 (19), 78 (20). HRMS (EI, $C_9H_7O_1N_5$): calcd 201.0645, found 201.0646; IR (ATR, ṽ) = 3092 (w), 2955 (w), 2166 (vw), 1578 (vs), 1523 (vs), 1486 (m), 1432 (s), 1346 (vs), 1322 (m), 1293 (m), 1245 (vs), 1215 (w), 1200 (vs), 1133 (m), 1106 (m), 1016 (w), 992 (w), 955 (vs), 901 (w), 771 (vs), 735 (w), 718 (m), 705 (w), 632 (s), 477 (m), 469 (vs), 414 (w) cm^{-1}.

^{1}H NMR (400 MHz, DMSO-d_6, ppm) δ = 12.56 (bs, 1H, N*H*), 8.27 (d, ^3J = 7.9 Hz, 1H, NCC*H*ar), 7.63 (t, ^3J = 7.8 Hz, 1H, C*H*ar), 7.50 (d, ^3J = 7.9 Hz, 1H, NCC*H*ar), 7.45 (t, ^3J = 7.8 Hz, 1H, C*H*ar).

Additional information on the chemical synthesis is available via Chemotion repository: https://doi.org/10.14272/reaction/SA-FUHFF-UHFFFADPSC-DJDCNOUVOP-UHFFFADPSC-NUHFF-NUHFF-NUHFF-ZZZ

Additional information on the analysis of the target compound is available via Chemotion repository:
https://doi.org/10.14272/VEKBUDDBMJNFNK-UHFFFAOYSA-N.1
https://doi.org/10.14272/SECOVEPJEIBBPV-UHFFFAOYSA-N.2

The synthesis of the target compound 4-methoxytetrazolo[1,5-*a*]quinoxaline has been previously described in literature [20].

5*H*-tetrazolo[1,5-*a*]quinoxalin-4-one (11h)

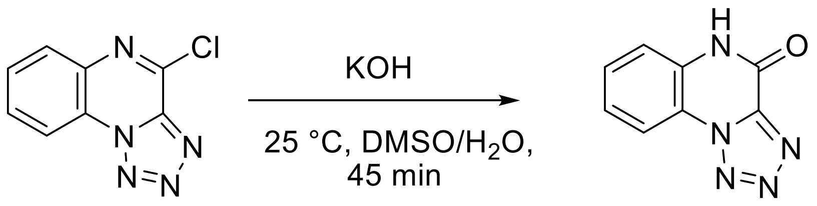

Name {P1|**11h**}: 5*H*-tetrazolo[1,5-*a*]quinoxalin-4-one; Formula: $C_8H_5N_5O$; Molecular Mass: 187.1582; Exact Mass: 187.0494; Smiles: O=c1[nH]c2ccccc2n2c1nnn2; InChIKey: SECOVEPJEIBBPV-UHFFFAOYSA-N

The starting material 4-chlorotetrazolo[1,5-*a*]quinoxaline (198 mg, 963 µmol, 1.00 equiv) was dissolved in DMSO (6.00 mL); then 2 mL of water and potassium;hydroxide (273 mg, 4.86 mmol, 5.00 equiv) were added. The dark red reaction mixture was stirred at 25 °C for 45 min, then a 1 M solution of HCl was added until the pH was acidic. The precipitated product was collected via filtration and washed 3× with water;

5*H*-tetrazolo[1,5-*a*]quinoxalin-4-one (174 mg, 930 µmol, 97% yield) was obtained as a light yellow solid.

R_f = 0.11 (cyclohexane/ethyl acetate 1:1). ^{1}H NMR (400 MHz, DMSO-d_6, ppm) δ = 12.56 (bs, 1H, NH), 8.26 (dd, ^3J = 8.3 Hz, ^4J = 1.4 Hz, 1H, CH_{ar}), 7.64–7.60 (m, 1H, CH_{ar}), 7.49 (dd, ^3J = 8.3 Hz, ^4J = 1.4 Hz, 1H, CH_{ar}), 7.46–7.42 (m, 1H, CH_{ar}); ^{13}C NMR (100 MHz, DMSO-d_6, ppm) δ = 151.2 (1C, N*C*O), 144.4 (1C, N*C*N), 129.9 (1C, *C*H_{ar}), 129.6 (1C, C_q), 124.0 (1C, *C*H_{ar}), 120.0 (1C, C_q), 116.9 (1C, *C*H_{ar}), 116.5 (1C, *C*H_{ar}); MS (ESI, negative Mode), m/z (%): 186 [M-1]$^-$ (100), 158 (34), 111 (27). HRMS (ESI, $C_8H_5N_5O$): calcd 186.0415, found 186.0412; IR (ATR, ṽ) = 3174 (w), 3104 (m), 3051 (w), 3016 (w), 2959 (w), 2928 (w), 2898 (w), 2863 (w), 1704 (m), 1666 (vs), 1622 (s), 1519 (m), 1472 (s), 1453 (s), 1418 (m), 1336 (vs), 1323 (s), 1264 (m), 1245 (s), 1210 (s), 1156 (w), 1139 (w), 1113 (w), 1069 (m), 1017 (w), 993 (w), 943 (w), 864 (w), 792 (m), 761 (vs), 728 (m), 704 (vs), 677 (vs), 652 (vs), 540 (w), 459 (vs), 446 (s) cm^{-1}.

Additional information on the chemical synthesis is available via Chemotion repository: https://doi.org/10.14272/reaction/SA-FUHFF-UHFFFADPSC-SECOVEPJEI-UHFFFADPSC-NUHFF-NUHFF-NUHFF-ZZZ

Additional information on the analysis of the target compound is available via Chemotion repository:
https://doi.org/10.14272/SECOVEPJEIBBPV-UHFFFAOYSA-N.1

The synthesis of this compound has been previously described and the ^{1}H NMR data corresponds with the literature [21].

***N,N*-dimethyltetrazolo[1,5-*a*]quinoxalin-4-amine (11i)**

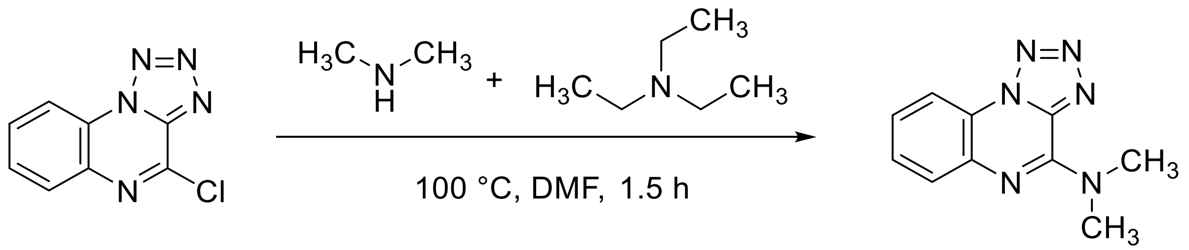

Name {P1|**11i**}: *N,N*-dimethyltetrazolo[1,5-*a*]quinoxalin-4-amine; Formula: $C_{10}H_{10}N_6$; Molecular Mass: 214.2266; Exact Mass: 214.0967; Smiles: CN(c1nc2ccccc2n2c1nnn2)C; InChIKey: MKRBRLWZSNAOKA-UHFFFAOYSA-N

The starting material 4-chlorotetrazolo[1,5-*a*]quinoxaline (50.0 mg, 243 µmol, 1.00 equiv) was added to a crimp vial and dissolved in *N,N*-dimethylformamide (1.00 mL), then dimethylamine (137 mg, 154 µL, 1.22 mmol, 5.00 equiv) and *N,N*-diethylethanamine (246 mg, 339 µL, 2.43 mmol, 10.0 equiv) were added. The reaction mixture was stirred at 100 °C for 1.5 h, water and EtOAc were added and the aqueous phase was extracted 3× with EtOAc. The combined organic phases were dried over Na_2SO_4, filtered and the solvent was removed under reduced pressure. The crude product was purified via column chromatography (dryload on Celite, eluent *c*Hex/EtOAc 2:1) and *N,N*-dimethyltetrazolo[1,5-*a*]quinoxalin-4-amine (49.0 mg, 229 µmol, 94% yield) was obtained as a yellow solid.

R_f = 0.36 (cyclohexane/ethyl acetate 4:1). ^{1}H NMR (400 MHz, DMSO-d_6, ppm) δ = 8.29 (d, ^{3}J = 8.2 Hz, 1H, CH_{ar}), 7.71 (d, ^{3}J = 8.3 Hz, 1H, CH_{ar}), 7.65–7.61 (m, 1H, CH_{ar}), 7.47–7.43 (m, 1H, CH_{ar}), 3.58 (bs, 6H, CH_3); ^{13}C NMR (100 MHz, DMSO-d_6, ppm) δ = 145.9 (1C, NC_qN), 139.2 (1C, C_q), 137.5 (1C, C_q), 129.6 (1C, CH$_{ar}$), 126.0 (1C, CH$_{ar}$), 124.1 (1C, CH$_{ar}$), 121.4 (1C, C_q), 115.6 (1C, CH$_{ar}$). Missing C (2C, CH$_3$) due to overlap with solvent peak at 39.5 ppm; confirmed via HSQC; MS (EI, 70 eV, 80 °C), m/z (%): 214 $[M]^+$ (17), 186 (18), 185 (100), 171 (26), 146 (20), 144 (35), 118 (15). HRMS (EI, $C_{10}H_{10}N_6$): calcd 214.0961, found 214.0960; IR (ATR, ṽ) = 3085 (w), 3067 (w), 2922 (w), 2891 (w), 2851 (w), 1585 (s), 1565 (vs), 1511 (m), 1493 (m), 1448 (m), 1435 (m), 1417 (s), 1400 (s), 1385 (s), 1320 (m), 1305 (m), 1268 (m), 1230 (m), 1163 (m), 1129 (m), 1105 (s), 1058 (m), 1038 (m), 1014 (m), 1007 (m), 952 (m), 931 (m), 867 (w), 849 (s), 768 (vs), 734 (m), 705 (m), 670 (m), 630 (s), 484 (w), 469 (s), 452 (m), 375 (m) cm^{-1}; EA ($C_{10}H_{10}N_6$): Calcd C 56.07; H 4.70; N 39.23. Found C 56.06; H 4.67; N 38.51.

Additional information on the chemical synthesis is available via Chemotion repository: https://doi.org/10.14272/reaction/SA-FUHFF-UHFFFADPSC-MKRBRLWZSN-UHFFFADPSC-NUHFF-NUHFF-NUHFF-ZZZ

Additional information on the analysis of the target compound is available via Chemotion repository:
https://doi.org/10.14272/MKRBRLWZSNAOKA-UHFFFAOYSA-N.1

1-[4-(Tetrazolo[1,5-*a*]quinoxalin-4-ylamino)phenyl]ethanone (11j)

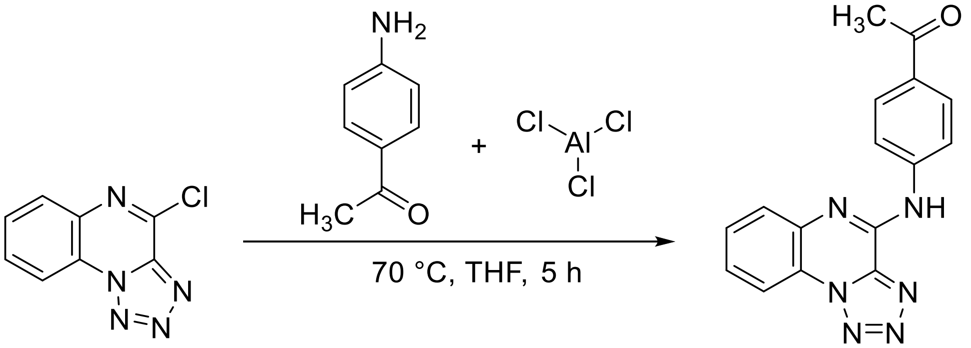

Name {P1|**11j**}: 1-[4-(tetrazolo[1,5-*a*]quinoxalin-4-ylamino)phenyl]ethanone; Formula: $C_{16}H_{12}N_6O$; Molecular Mass: 304.3061; Exact Mass: 304.1073; Smiles: CC(=O)c1ccc(cc1)Nc1nc2ccccc2n2c1nnn2; InChIKey: OIHNAVUWMWBKPA-UHFFFAOYSA-N

The starting material 4-chlorotetrazolo[1,5-*a*]quinoxaline (100 mg, 486 µmol, 1.00 equiv), 1-(4-aminophenyl)ethanone (78.9 mg, 584 µmol, 1.20 equiv) and aluminum;trichloride (97.3 mg, 730 µmol, 1.50 equiv) were suspended in 2 mL of dry THF. The orange reaction mixture was heated under nitrogen to 70 °C and stirred for 5 hours. Water was added to the reaction mixture and the aqueous phase was extracted 3× with EtOAc. The combined organic phases were dried over Na_2SO_4 and filtered; the solvent was removed under reduced pressure. The crude product was purified twice using column chromatography (dryload on Celite, eluent *c*Hex/ethyl acetate 2:1, then *c*Hex + 0.5% Et_3N/ethyl acetate 2:1) and 1-[4-(tetrazolo[1,5-*a*]quinoxalin-4-ylamino)phenyl]ethanone (141 mg, 463 µmol, 95% yield) was isolated

as a yellow solid. 20 mg of the product could be obtained in pure form; the remaining 121 mg contained minor impurities.

R_f = 0.56 (cyclohexane/ethyl acetate 2:1). ^{1}H NMR (400 MHz, DMSO-d$_6$, ppm) δ = 10.99 (s, 1H, N*H*), 8.40–8.35 (m, 3H, 2x NHC*H*$_{ar}$, 1x C*H*$_{ar}$CN), 8.01–7.99 (m, 2H, C*H*$_{ar}$CCO), 7.95–7.93 (d, ^3J = 7.6 Hz, 1H, C*H*$_{ar}$CN), 7.76–7.72 (m, 1H, C*H*$_{ar}$CHCN), 7.66–7.62 (m, 1H, C*H*$_{ar}$CHCN), 2.57 (s, 3H, C*H*$_3$); ^{13}C NMR (100 MHz, DMSO-d$_6$, 39.51 ppm) δ = 196.4 (1C, CO), 143.7 (1C, NH*C*$_{ar}$), 142.3 (1C, N*C*N), 138.9 (1C, N*C*N), 136.7 (1C, *C*$_{ar}$N), 131.5 (1C, *C*$_{ar}$CO), 129.6 (1C, *C*H$_{ar}$CHCN), 129.2 (2C, CH$_{ar}$CCO), 127.2 (CH$_{ar}$CN), 126.3 (1C, *C*H$_{ar}$CHCN), 122.7 (1C, *C*$_{ar}$N), 119.7 (2C, NH*C*H$_{ar}$), 115.8 (CH$_{ar}$CN), 26.4 (1C, *C*H$_3$). MS (EI, m/z, 70 eV, 170 °C): 304 (42) [M]+, 262 (19), 261 (100), 234 (62), 233 (58), 232 (21), 206 (20), 179 (16), 163 (19), 91 (34), 90 (16), 85 (15), 73 (27), 71 (51), 69 (15), 58 (23), 27 (26), 55 (38). HRMS ($C_{16}H_{12}O_1N_6$): calcd 304.1073, found 304.1071. IR (ATR, ṽ) = 3312 (m), 3200 (w), 1663 (s), 1602 (s), 1565 (vs), 1538 (vs), 1502 (vs), 1482 (vs), 1431 (m), 1408 (vs), 1360 (s), 1349 (vs), 1329 (s), 1316 (s), 1273 (vs), 1244 (vs), 1184 (vs), 1132 (s), 1103 (s), 1016 (m), 992 (s), 962 (s), 946 (m), 925 (m), 844 (vs), 827 (vs), 764 (vs), 731 (m), 717 (m), 637 (s), 628 (s), 620 (vs), 591 (vs), 579 (vs), 527 (s), 517 (s), 497 (s), 469 (vs), 411 (m) cm^{-1}.

Additional information on the chemical synthesis is available via Chemotion repository: https://doi.org/10.14272/reaction/SA-FUHFF-UHFFFADPSC-OIHNAVUWMW-UHFFFADPSC-NUHFF-NUHFF-NUHFF-ZZZ

Additional information on the analysis of the target compound is available via Chemotion repository:
https://doi.org/10.14272/OIHNAVUWMWBKPA-UHFFFAOYSA-N.1

The synthesis of this compound has been previously in literature [22].

4-((3,3,4,4,5,5,6,6,7,7,8,8,9,9,10,10,10-Heptadecafluorodecyl)oxy)tetrazolo[1,5-*a*]quinoxaline (11k)

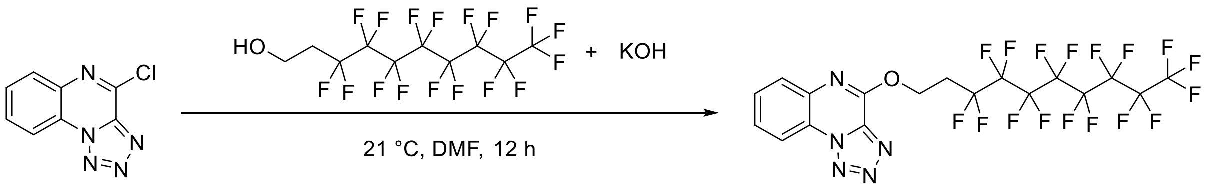

Name {P1|**11k**}: 4-((3,3,4,4,5,5,6,6,7,7,8,8,9,9,10,10,10-heptadecafluorodecyl)oxy)tetrazolo[1,5-*a*]quinoxaline; Formula: $C_{18}H_8F_{17}N_5O$; Molecular Mass: 633.2619; Exact Mass: 633.0457; Smiles: FC(C(C(C(C(C(C(C(F)(F)F)(F)F)(F)F)(F)F)(F)F)(F)F)(F)F)(CCOc1nc2ccccc2n2c1nnn2)F; InChIKey: GAIYTCZAQRWQIX-UHFFFAOYSA-N

N,N-Dimethylformamide (8.0 mL), potassium hydroxide (81.9 mg, 1.46 mmol, 1.50 equiv), 3,3,4,4,5,5,6,6,7,7,8,8,9,9,10,10,10-heptadecafluoro-1-decanol (497 mg, 1.07 mmol, 1.10 equiv) and 4-chloranyl-[1,2,3,4]tetrazolo[1,5-a]quinoxaline (200 mg, 973 µmol, 1.00 equiv) were mixed together under N_2 atmosphere at 21 °C. The mixture

was stirred at 21 °C for 12 h. *N,N*-Dimethylformamide was evaporated under strong reduced pressure (55 °C waterbath). Then methylene chloride (50 mL) and brine (50 mL) were added. The aqueous layer was extracted 3× with methylene chloride (50 mL). The combined organic layers were dried over Na_2SO_4, filtered and solvent was removed under reduced pressure. The obtained crude product was purified via flash-chromatography on silica gel using cyclohexane/ethyl acetate 1:0 to 6:1 to afford 4-((3,3,4,4,5,5,6,6,7,7,8,8,9,9,10,10,10-heptadecafluorodecyl)oxy)tetrazolo[1,5-*a*]quinoxaline (430 mg, 679 µmol, 70% yield) as a colorless solid.

R_f = 0.46 (cyclohexane/ethyl acetate 4:1). ^{1}H NMR (400 MHz, $CDCl_3$ [7.27 ppm], ppm) δ = 8.51 (dd, *J* = 7.8 Hz, *J* = 1.8 Hz, 1H, -CHCN), 8.00 (dd, *J* = 7.6 Hz, *J* = 1.7 Hz, 1H, -CHCN), 7.74 (dtd, *J* = 16.6 Hz, *J* = 7.4 Hz, *J* = 1.6 Hz, 2H, -CHCHCN), 5.05 (t, *J* = 6.9 Hz, 2H, $-OCH_2$), 2.75–3.01 (m, 2H, $-OCH_2CH_2$); ^{13}C NMR (100 MHz, $CDCl_3$ [77.0 ppm], ppm) δ = 150.2 (C_q), 138.5 (C_q), 135.3 (C_q), 130.2 (C_H), 128.5 (C_H), 128.4 (C_H), 124.0 (C_q), 116.6 (C_H), 60.3 (t, *J* = 5.0 Hz), 30.8 (t, *J* = 21.8 Hz). Missing signals (8C, CF_2 and CF_3). ^{19}F NMR (377 MHz, CDCl3, ppm) δ = -80.82 (t, J = 10.0 Hz, CF3), -113.12 – -113.73 (m, CF2), -121.30 – -121.74 (m, CF2), -121.74 – -122.10 (m, 2 x CF2), -122.51 – -122.97 (m, CF2), -123.17 – -123.74 (m, CF2), -125.82 – -126.45 (m, CF2). MS (FAB m/z, Matrix: 3-NBA): 635 (23) $[M+H]^+$, 634 (100) [M]+, 177 (13), 133 (45), 89 (89), 87 (37). HRMS–FAB (m/z): $[M+H]^+$ calcd for $C_{18}H_9O_1N_5F_{17}$, 634.0531; found, 634.0530. IR (ATR, ṽ) = 1616, 1591, 1577, 1523, 1486, 1473, 1459, 1434, 1402, 1370, 1351, 1326, 1295, 1196, 1145, 1135, 1115, 1084, 1068, 1037, 1016, 1009, 984, 962, 952, 912, 902, 877, 857, 844, 824, 806, 782, 764, 738, 725, 704, 688, 654, 639, 620, 606 cm^{-1}.

Additional information on the chemical synthesis is available via Chemotion repository: https://doi.org/10.14272/reaction/SA-FUHFF-UHFFFADPSC-GAIYTCZAQR-UHFFFADPSC-NUHFF-NUHFF-NUHFF-ZZZ

Additional information on the analysis of the target compound is available via Chemotion repository: https://doi.org/10.14272/GAIYTCZAQRWQIX-UHFFFAOYSA-N.1

4-((Trimethylsilyl)ethynyl)tetrazolo[1,5-*a*]quinoxaline (11l)

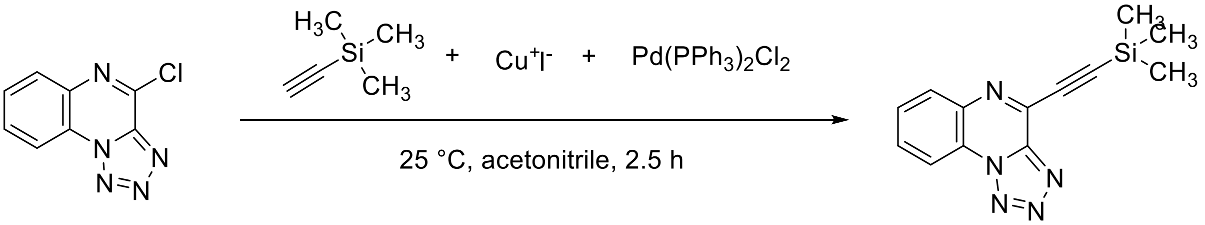

Name {P1|**11l**}: 4-((trimethylsilyl)ethynyl)tetrazolo[1,5-*a*]quinoxaline; Formula: $C_{13}H_{13}N_5Si$; Molecular Mass: 267.3613; Exact Mass: 267.0940; Smiles: C[Si](C#Cc1nc2ccccc2n2c1nnn2)(C)C; InChIKey: RRWJGDBWTOAAFG-UHFFFAOYSA-N

The starting material 4-chlorotetrazolo[1,5-*a*]quinoxaline (49.0 mg, 238 µmol, 1.00 equiv), copper(1+);iodide (20.0 mg, 105 µmol, 0.441 equiv) and dichloropalladium;triphenylphosphane (17.1 mg, 24.3 µmol, 0.102 equiv) were dissolved in 1 mL of dry acetonitrile. Then trimethylsilylacetylene (71.7 mg, 103 µL,

730 µmol, 3.06 equiv) and triethylamine (0.25 mL) were added and the reaction was stirred at 25 °C for 2.5 h under argon. The reaction mixture was filtered over Celite and water and EtOAc were added. The organic phase was separated, the aqueous phase was extracted 3× with EtOAc and the combined organic phases were dried over Na_2SO_4. The combined organic phases were filtered and the solvent was removed under reduced pressure. The crude product was purified via column chromatography (dryload on Celite, *c*Hex -> *c*Hex/EtOAc 2:1) and 4-((trimethylsilyl)ethynyl)tetrazolo[1,5-*a*]quinoxaline (54.0 mg, 202 µmol, 85% yield) was obtained as a brown solid.

R_f = 0.52 (cyclohexane/ethyl acetate 2:1). ^{1}H NMR (400 MHz, $CDCl_3$, ppm) δ = 8.61 (dd, ^3J = 8.0 Hz, ^4J = 1.8 Hz, 1H, NCCH_{ar}), 8.28 (dd, ^3J = 8.2 Hz, ^4J = 1.6 Hz, 1H, NCCH_{ar}), 7.93–7.84 (m, 2H, CH_{ar}), 0.39 (s, 9H, CH_3); ^{13}C NMR (100 MHz, $CDCl_3$, ppm) δ = 143.2 (1C, N*C*N), 136.9 (1C, C_q), 134.3 (1C, C_q), 131.9 (1C, *C*H_{ar}), 130.5 (1C, NC*C*H_{ar}), 130.1 (1C, *C*H_{ar}), 124.5 (1C, C_q), 116.3 (1C, NC*C*H_{ar}), 107.6 (1C, *C*CSi), 97.4 (1C, *C*Si), -0.59 (3C, *C*H_3); MS (EI, 70 eV, 90 °C), m/z (%): 267 $[M]^+$ (4), 225 (20), 224 (100), 108 (13). HRMS (EI, $C_{13}H_{13}N_5{}^{28}Si_1$): Calcd 267.0935, Found 267.0934; IR (ATR, ṽ) = 2963 (w), 2902 (vw), 1611 (vw), 1509 (s), 1465 (w), 1402 (w), 1339 (w), 1324 (w), 1290 (w), 1248 (s), 1228 (m), 1214 (w), 1171 (s), 1156 (w), 1125 (w), 1095 (w), 1044 (w), 1010 (w), 986 (w), 881 (w), 840 (vs), 772 (vs), 762 (vs), 711 (m), 697 (m), 657 (s), 625 (w) cm^{-1}.

Additional information on the chemical synthesis is available via Chemotion repository: https://doi.org/10.14272/reaction/SA-FUHFF-UHFFFADPSC-RRWJGDBWTO-UHFFFADPSC-NUHFF-NUHFF-NUHFF-ZZZ

Additional information on the analysis of the target compound is available via Chemotion repository:
https://doi.org/10.14272/RRWJGDBWTOAAFG-UHFFFAOYSA-N.1

(3-Chloroquinoxalin-2-yl)hydrazine (12)

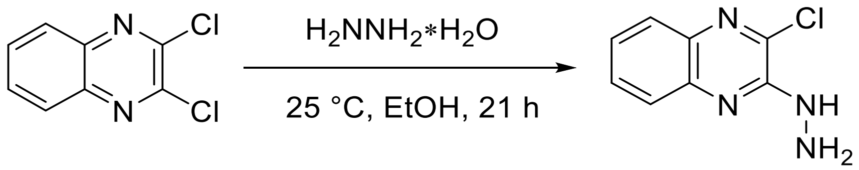

Name {P1|**12**}: (3-chloroquinoxalin-2-yl)hydrazine; Formula: $C_8H_7ClN_4$; Molecular Mass: 194.6210; Exact Mass: 194.0359; Smiles: NNc1nc2ccccc2nc1Cl; InChIKey: RODNZCIFRICALV-UHFFFAOYSA-N

The starting material 2,3-dichloroquinoxaline (500 mg, 2.51 mmol, 1.00 equiv) was dissolved in 15 mL of ethanol, hydrazine;hydrate (252 mg, 244 µL, 5.02 mmol, 2.00 equiv) was added and the yellow solution was stirred at 25 °C for 21 hours. Water was added and the aqueous phase was extracted 3x with EtOAc. The aqueous phase was quenched with an aqueous solution of 3% H_2O_2 in order to remove any remaining hydrazine; then a saturated solution of $Na_2S_2O_3$ was added to quench remaining hydrogen peroxide. The combined organic phases were dried over Na_2SO_4 and filtered, the solvent was removed under reduced pressure. The crude product was purified using column chromatography (dryload on Celite, eluent *c*Hex/EtOAc 1:1).

The product was obtained as a yellow solid in 88% yield (432 mg, 2.22 mmol) that turns orange after contact with air for some days.

R_f = 0.13 (cyclohexane/ethyl acetate 4:1). ^{1}H NMR (400 MHz, DMSO-d_6, ppm) δ = 8.86 (bs, 1H, NH), 7.74 (d, ^3J = 8.1 Hz, 1H, CH_{arom}N), 7.68–7.60 (m, 2H, CH_{arom}), 7.40 (t, ^3J = 7.2 Hz, 1H, CH_{arom}CH), 4.61 (bs, 2H, NH_2); ^{13}C NMR (100 MHz, DMSO-d_6, ppm) δ = 149.4 (1C, *C*NHNH_2), 140.7 (1C, N*C*Cl), 136.8 (1C, C_{arom}N), 135.6 (1C, C_{arom}N), 130.3 (1C, *C*H_{arom}), 127.5 (1C, CH_{arom}CN), 125.2 (1C, CH_{arom}), 124.5 (1C, CH_{arom}CHCN); EI (m/z, 70 eV, 60 °C): 194/196 (100/33) $[M]^+$, 158 (16), 130 (27), 129 (62), 103 (25), 102 (44), 90 (18). HRMS ($C_8H_7N_4{}^{35}Cl_1$): calcd 194.0359, found 194.0361; IR (ATR, ṽ) = 3310 (w), 3224 (m), 1629 (w), 1571 (w), 1554 (m), 1503 (s), 1493 (s), 1459 (m), 1411 (m), 1350 (w), 1339 (m), 1298 (m), 1248 (w), 1123 (m), 1069 (vs), 1017 (w), 962 (s), 932 (m), 871 (w), 826 (w), 765 (vs), 653 (s), 606 (s), 588 (vs), 558 (s), 486 (m), 445 (vs) cm^{-1}.

Additional information on the chemical synthesis is available via Chemotion repository: https://doi.org/10.14272/reaction/SA-FUHFF-UHFFFADPSC-RODNZCIFRI-UHFFFADPSC-NUHFF-NUHFF-NUHFF-ZZZ.1

Additional information on the analysis of the target compound is available via Chemotion repository:
https://doi.org/10.14272/RODNZCIFRICALV-UHFFFAOYSA-N.2

The synthesis of this compound has been previously in literature [7].

4,5-Dihydrotetrazolo[1,5-*a*]quinoxaline (S3)

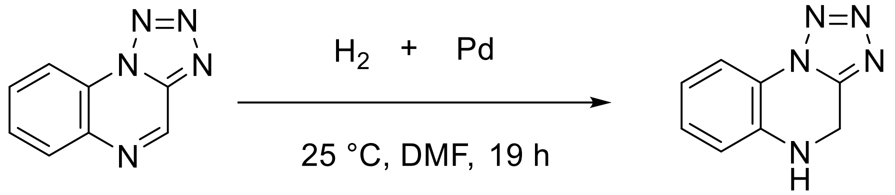

Name {P1|**S3**}: 4,5-dihydrotetrazolo[1,5-*a*]quinoxaline; Formula: $C_8H_7N_5$; Molecular Mass: 173.1747; Exact Mass: 173.0701; Smiles: c1ccc2c(-n3nnnc3CN2)c1; InChIKey: JJCQHCJBVSOFTM-UHFFFAOYSA-N

The starting material tetrazolo[1,5-*a*]quinoxaline (50.0 mg, 292 µmol, 1.00 equiv) and palladium (10% on active charcoal, 31.1 mg, 29.2 µmol, 0.100 equiv) were added to a flame-dried flask and the flask was evacuated. Then 2.5 mL of DMF was added and the reaction mixture was stirred under a hydrogen gas atmosphere for 19 h. Water and EtOAc were added and the aqueous phase was extracted 3× with ethyl acetate. The combined organic layers were dried over Na_2SO_4 and the solvent was removed under reduced pressure. The crude product was purified via column chromatography (dryload on Celite, *c*Hex -> ethyl acetate) and 4,5-dihydrotetrazolo[1,5-*a*]quinoxaline (44.0 mg, 254 µmol, 87% yield) was obtained as a colourless solid.

R_f = 0.13 (cyclohexane/ethyl acetate 2:1). ^{1}H NMR (400 MHz, $CDCl_3$, ppm) δ = 7.89 (dd, ^3J = 8.0 Hz, ^4J = 1.4 Hz, 1H, CH_{ar}), 7.21(td, ^3J = 7.8 Hz, ^4J = 1.8 Hz, 1H, CH_{ar}), 6.95 (td, ^3J = 7.6 Hz, ^4J = 1.2 Hz, 1H, CH_{ar}), 6.85 (dd, ^3J = 8.1 Hz, ^4J = 1.2 Hz, 1H, CH_{ar}), 4.96 (s, 2H, CH_2), 3.11 (bs, 1H, N*H*); ^{13}C NMR (100 MHz, $CDCl_3$ [77.0 ppm],

ppm) δ = 146.7 (1C, *C*q), 135.3 (1C, *C*q), 129.5 (1C, *C*Har), 120.1 (1C, *C*q), 119.9 (1C, *C*Har), 117.3 (1C, *C*Har), 115.2 (1C, *C*Har), 39.5 (1C, *C*H2); MS (EI, 70 eV, 90 °C), m/z (%): 173 $[M]^+$ (48), 145 (55), 144 (100), 119 (22), 118 (88), 91 (26), 90 (19). HRMS (EI, $C_8H_7N_5$): calcd 173.0696, found 173.0695; IR (ATR, ṽ) = 3327 (s), 3064 (w), 1622 (m), 1509 (m), 1489 (s), 1475 (s), 1465 (s), 1434 (m), 1350 (w), 1323 (s), 1309 (m), 1265 (s), 1245 (s), 1159 (m), 1145 (m), 1112 (m), 1088 (s), 1060 (m), 1040 (m), 1018 (m), 1000 (m), 980 (m), 921 (w), 864 (w), 850 (w), 747 (vs), 732 (vs), 705 (m), 690 (vs), 681 (s), 628 (s), 569 (s), 554 (s), 514 (s), 459 (m), 441 (vs), 435 (vs) cm^{-1}.

Additional information on the chemical synthesis is available via Chemotion repository: https://doi.org/10.14272/reaction/SA-FUHFF-UHFFFADPSC-JJCQHCJBVS-UHFFFADPSC-NUHFF-NUHFF-NUHFF-ZZZ

Additional information on the analysis of the target compound is available via Chemotion repository:
https://doi.org/10.14272/JJCQHCJBVSOFTM-UHFFFAOYSA-N.1

The synthesis of this compound has been previously in literature [23].

5-Methyl-4*H*-tetrazolo[1,5-*a*]quinoxaline (S4)

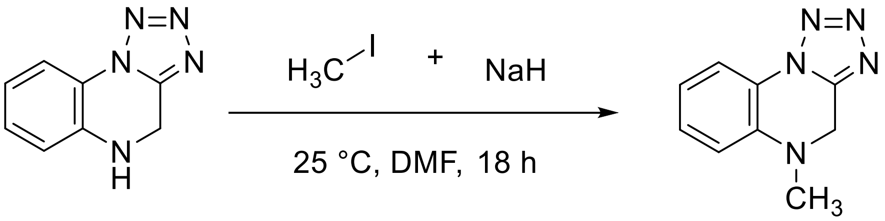

Name {P1|**S4**}: 5-methyl-4*H*-tetrazolo[1,5-*a*]quinoxaline; Formula: $C_9H_9N_5$; Molecular Mass: 187.2013; Exact Mass: 187.0858; Smiles: CN1Cc2nnnn2-c2c1cccc2; InChIKey: VJXDMNNLLZYTRN-UHFFFAOYSA-N

The starting material 4,5-dihydrotetrazolo[1,5-a]quinoxaline (51.0 mg, 295 µmol, 1.00 equiv) was dissolved in 2 mL of dry DMF and sodium hydride (24.0 mg, 600 µmol, 2.04 equiv) was added. Then iodomethane (123 mg, 53.9 µL, 866 µmol, 3.00 equiv) was introduced into the solution and the reaction mixture was stirred for 18 h at 25 °C. Subsequently, a solution of 10% ammonia in water was added was added in order to quench the reaction. The aqueous phase was extracted 3× with EtOAc; the combined organic phases were dried over Na_2SO_4, filtered and the solvent was removed under reduced pressure. The crude product was purified via column chromatography (dryload on Celite, eluent *c*Hex/EtOAc 1:4) and 5-methyl-4*H*-tetrazolo[1,5-*a*]quinoxaline (39.0 mg, 208 µmol, 71% yield) was obtained as a beige solid.

R_f = 0.39 (cyclohexane/ethyl acetate 1:1). ^{1}H NMR (400 MHz, $CDCl_3$, ppm) δ = 7.94 (dd, ^3J = 7.9 Hz, ^4J = 1.5 Hz, 1H, C*H*ar), 7.35–7.31 (m, 1H, C*H*ar), 6.98 (td, ^3J = 7.7 Hz, ^4J = 1.2 Hz, 1H, C*H*ar), 6.85 (d, ^3J = 8.3 Hz, 1H, C*H*ar), 4.77 (s, 2H, NC*H*2), 3.04 (s, 3H, C*H*3); ^{13}C NMR (100 MHz, $CDCl_3$ [77.0 pp], ppm) δ = 146.7 (1C, N*C*N), 137.0 (1C, *C*q), 129.7 (1C, *C*Har), 120.8 (1C, *C*q), 119.3 (1C, *C*Har), 117.1 (1C, *C*Har), 113.1 (1C, *C*Har), 47.0 (1C, N*C*H2), 38.0 (1C, *C*H3); MS (EI, 70 eV, 70 °C), m/z (%): 187 (15) $[M]^+$, 159

(24), 158 (100), 90 (9). HRMS (EI, $C_9H_9N_5$): calcd 187.0852, found 187.0851; IR (ATR, ṽ) = 2823 (w), 2802 (w), 1621 (m), 1509 (s), 1486 (s), 1459 (m), 1428 (m), 1387 (m), 1324 (s), 1272 (m), 1248 (m), 1214 (m), 1164 (w), 1143 (m), 1113 (s), 1078 (m), 1041 (w), 1020 (m), 1003 (s), 967 (m), 919 (w), 849 (w), 796 (w), 745 (vs), 704 (m), 676 (s), 571 (w), 554 (w), 524 (w), 463 (m), 422 (w) cm^{-1}.

Additional information on the chemical synthesis is available via Chemotion repository: https://doi.org/10.14272/reaction/SA-FUHFF-UHFFFADPSC-VJXDMNNLLZ-UHFFFADPSC-NUHFF-NUHFF-NUHFF-ZZZ

Additional information on the analysis of the target compound is available via Chemotion repository:
https://doi.org/10.14272/VJXDMNNLLZYTRN-UHFFFAOYSA-N.1

The synthesis of this compound has been previously in literature [23].

2-(4-Phenyl-1*H*-1,2,3-triazol-1-yl)quinoxaline (14a)

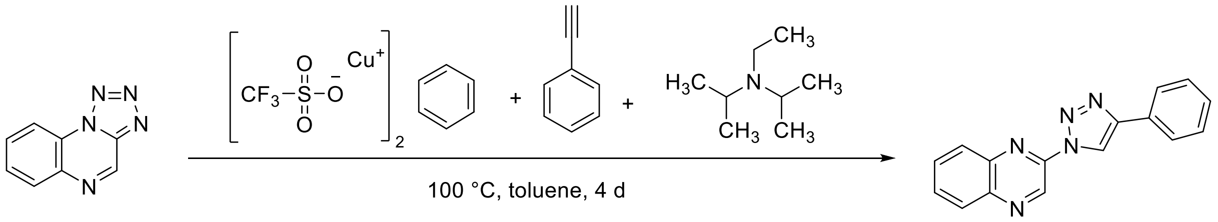

Name {P1|**14a**}: 2-(4-phenyl-1*H*-1,2,3-triazol-1-yl)quinoxaline; Formula: $C_{16}H_{11}N_5$; Molecular Mass: 273.2920; Exact Mass: 273.1014; Smiles: c1ccc(cc1)c1nnn(c1)c1cnc2c(n1)cccc2; InChIKey: QYOUUXWQIVRDNZ-UHFFFAOYSA-N

The starting material tetrazolo[1,5-*a*]quinoxaline (51.0 mg, 298 µmol, 1.00 equiv) and the catalyst benzene;copper(1+);trifluoromethanesulfonate (15.0 mg, 29.8 µmol, 0.100 equiv) were dissolved in 1 mL of dry toluene under nitrogen, followed by ethynylbenzene (59.7 mg, 64.2 µL, 584 µmol, 1.96 equiv) and *N*-ethyl-*N*-propan-2-ylpropan-2-amine (113 mg, 149 µL, 876 µmol, 2.94 equiv). The reaction mixture was stirred at 100 °C for 42 h and subsequently stirred at 21 °C for 2 days. Then water and EtOAc were added, the organic phase was separated and the aqueous phase was extracted 3× with DCM . The combined organic phases were dried over Na_2SO_4, filtered and the solvent was removed under reduced pressure. The crude product was purified via column chromatography (*c*Hex+2% Et_3N/EtOAc 10:1) and the product 2-(4-phenyl-1*H*-1,2,3-triazol-1-yl)quinoxaline (54.0 mg, 198 µmol, 66% yield) was obtained as an orange solid.

R_f = 0.59 (cyclohexane/ethyl acetate 2:1). ^{1}H NMR (400 MHz, $CDCl_3$, ppm) δ = 9.88 (s, 1H, NC*H*CN), 8.95 (s, 1H, C$H_{triazole}$), 8.23–8.21 (m, 1H, CH_{ar}CN), 8.11–8.08 (m, 1H, CH_{ar}CN), 8.01–7.99 (m, 2H, CH_{phenyl}), 7.89–7.81 (m, 2H, CH_{ar}CHCN), 7.52–7.48 (m, 2H, CH_{phenyl}), 7.43–7.39 (m, 1H, CH_{phenyl}). ^{13}C NMR (100 MHz, $CDCl_3$ [77.0 ppm], ppm) δ = 148.5 (1C, C_q), 142.9 (1C, C_q), 142.3 (1C, C_q), 140.0 (1C, C_q), 137.7 (1C, *C*HCN), 131.5 (1C, *C*H_{ar}CHCN), 130.2 (1C, *C*H_{ar}CHCN), 129.7 (1C, C_q), 129.6 (1C, *C*H_{ar}CN), 129.0 (2C, *C*H_{phenyl}), 128.8 (1C, *C*H_{phenyl}), 128.7 (1C, *C*H_{ar}CN), 126.1 (2C,

CH_{phenyl}), 116.8 (1C, $CH_{triazole}$); MS (EI, m/z, 70 eV, 100 °C): 273 (10) [M]+, 246 (28), 245 (100), 244 (56), 129 (79), 117 (16), 102 (57). HRMS (EI, $C_{16}H_{11}N_5$): Calcd 273.1009, Found 273.1009; IR (ATR, ṽ) = 3176 (w), 3054 (w), 1567 (w), 1500 (vs), 1472 (m), 1456 (m), 1441 (s), 1358 (w), 1234 (w), 1214 (s), 1190 (w), 1142 (w), 1126 (w), 1074 (w), 1041 (w), 1003 (vs), 962 (w), 949 (m), 919 (m), 806 (w), 762 (vs), 694 (vs), 674 (w), 622 (w), 594 (m), 545 (w), 503 (w), 449 (w), 415 (vs) cm^{-1}; UV-VIS (acetonitrile), λ = 344 (1.24), 332 (1.30), 252 (2.04) nm.

Additional information on the chemical synthesis is available via Chemotion repository: https://doi.org/10.14272/reaction/SA-FUHFF-UHFFFADPSC-QYOUUXWQIV-UHFFFADPSC-NUHFF-NUHFF-NUHFF-ZZZ

Additional information on the analysis of the target compound is available via Chemotion repository:
https://doi.org/10.14272/QYOUUXWQIVRDNZ-UHFFFAOYSA-N.1

The synthesis of this compound has been previously described and the ^1H NMR data corresponds with the literature [6].

2-(4-(4-Methoxyphenyl)-1*H*-1,2,3-triazol-1-yl)quinoxaline (14b)

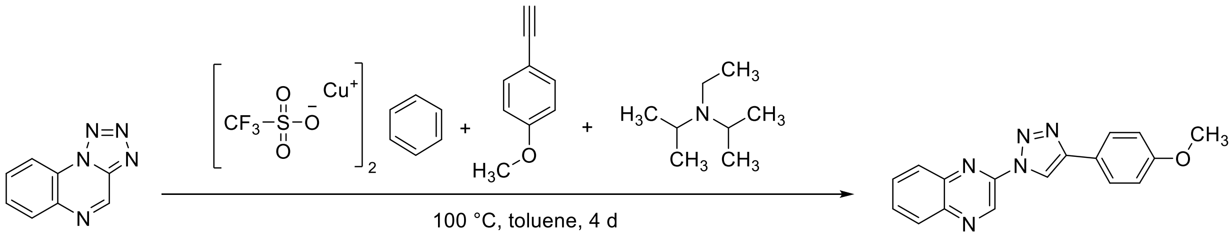

Name {P1|**14b**}: 2-(4-(4-methoxyphenyl)-1*H*-1,2,3-triazol-1-yl)quinoxaline; Formula: $C_{17}H_{13}N_5O$; Molecular Mass: 303.3180; Exact Mass: 303.1120; Smiles: COc1ccc(cc1)c1nnn(c1)c1cnc2c(n1)cccc2; InChIKey: SJCZTVUAGLTCCV-UHFFFAOYSA-N

The starting material tetrazolo[1,5-*a*]quinoxaline (49.9 mg, 292 µmol, 1.00 equiv) and the catalyst benzene;copper(1+);trifluoromethanesulfonate (14.4 mg, 28.6 µmol, 0.0981 equiv) were dissolved in 1 mL of dry toluene under argon, followed by 1-ethynyl-4-methoxybenzene (81.5 mg, 80.0 µL, 617 µmol, 2.12 equiv) and *N*-ethyl-*N*-propan-2-ylpropan-2-amine (76.0 mg, 100 µL, 588 µmol, 2.02 equiv). The green reaction mixture was stirred at 100 °C for 4 days until the TLC showed complete conversion of the starting material. Then water and DCM were added and the aqueous phase was extracted 3 times with DCM. The combined organic phases were dried over Na_2SO_4, filtered and the solvents were removed under reduced pressure. The obtained crude product was purified twice via flash-chromatography on silica gel using cHex/EtOAc 20:1 to cHex/EtOAc 4:1 and the expected product 2-(4-(4-methoxyphenyl)-1*H*-1,2,3-triazol-1-yl)quinoxaline (42.9 mg, 141 µmol, 49% yield) was obtained as a brown solid.

R_f = 0.3 (cyclohexane/ethyl acetate 4:1). ^1H NMR (400 MHz, Chloroform-d [7.27 ppm], ppm) δ = 9.87 (s, 1H, C*H*), 8.85 (s, 1H, C*H*), 8.22–8.20 (m, 1H, CH_{Ar}), 8.10–8.07 (m,

1H, CH_{Ar}), 7.92 (d, J = 8.8 Hz, 2H, CH_{Ar}), 7.88–7.80 (m, 2H, CH_{Ar}), 7.02 (d, J = 8.8 Hz, 2H, CH_{Ar}), 3.88 (s, 3H, CH_3); ^{13}C NMR (100 MHz, Chloroform-d [77.0 ppm], ppm) δ = 160.2 (1C, C_q), 148.4 (1C, C_q), 148,2 (1C, C_q) 143,1 (1C, C_q) 142.2 (1C, C_q), 137.8 (1C, CH_{Ar}), 131.5 (1C, CH_{Ar}), 130.2 (1C, CH_{Ar}), 129.6 (1C, CH_{Ar}), 128.7 (1C, CH_{Ar}), 127.5 (2C, CH_{Ar}), 122.4 (1C, C_q), 115.8 (1C, CH_{Ar}), 114.4 (2C, CH_{Ar}), 55.4 (1C, CH_3); MS (EI, m/z, 70 eV, 160 °C): 303 $[M]^+$ (11), 275 (100), 261 (12), 260 (63), 231 (20), 146 (30), 129 (18), 102 (26), 75 (26). HRMS ($C_{17}H_{13}O_1N_5$): Calcd 303.1116, Found 303.1115; IR (ATR, ṽ) = 3163 (w), 2918 (m), 2840 (w), 1615 (m), 1567 (s), 1497 (vs), 1482 (m), 1473 (s), 1448 (vs), 1419 (m), 1356 (m), 1330 (w), 1303 (m), 1289 (w), 1248 (vs), 1232 (vs), 1213 (vs), 1180 (vs), 1142 (m), 1123 (m), 1109 (m), 1092 (w), 1028 (s), 1016 (vs), 1003 (vs), 965 (m), 949 (vs), 919 (s), 833 (vs), 795 (vs), 768 (vs), 673 (s), 642 (m), 608 (vs), 589 (m), 540 (s), 531 (m), 516 (s), 487 (m), 442 (w), 416 (vs) cm^{-1}.

Additional information on the chemical synthesis is available via Chemotion repository: https://doi.org/10.14272/reaction/SA-FUHFF-UHFFFADPSC-SJCZTVUAGL-UHFFFADPSC-NUHFF-NUHFF-NUHFF-ZZZ.1

Additional information on the analysis of the target compound is available via Chemotion repository:
https://doi.org/10.14272/SJCZTVUAGLTCCV-UHFFFAOYSA-N.2

4-(1-(Quinoxalin-2-yl)-1*H*-1,2,3-triazol-4-yl)benzenaminium chloride (14c)

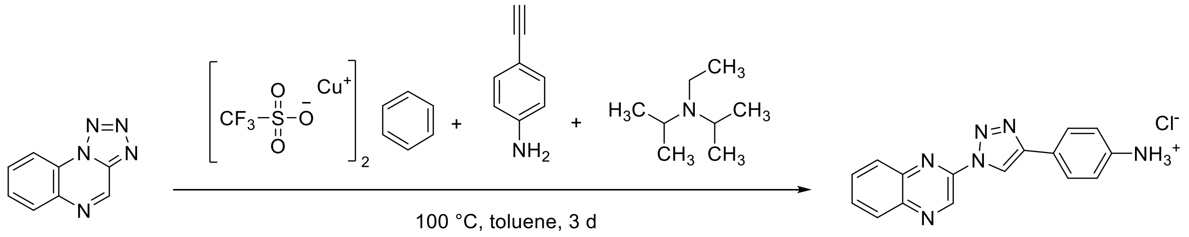

Name {P1|**14c**}: 4-(1-(quinoxalin-2-yl)-1*H*-1,2,3-triazol-4-yl)benzenaminium chloride; Formula: $C_{16}H_{13}ClN_6$; Molecular Mass: 324.7676; Exact Mass: 324.0890; Smiles: [NH3+]c1ccc(cc1)c1nnn(c1)c1cnc2c(n1)cccc2.[Cl-]; InChIKey: GRLFMIQVCULKIR-UHFFFAOYSA-N

The starting material tetrazolo[1,5-*a*]quinoxaline (99.7 mg, 576 µmol, 1.00 equiv), 4-ethynylaniline (131 mg, 119 µL, 1.12 mmol, 1.94 equiv) and the catalyst benzene;copper(1+);trifluoromethanesulfonate (27.1 mg, 53.8 µmol, 0.0935 equiv) were dissolved in 2 mL of dry toluene, under nitrogen, followed by the addition of *N*-ethyl-*N*-propan-2-ylpropan-2-amine (152 mg, 200 µL, 1.17 mmol, 2.04 equiv). The brown reaction mixture was stirred at 100 °C for 3 days. The TLC showed that starting materials were consumed. Then water and DCM were added and the aqueous phase was extracted 3 times with DCM. The combined organic phases were dried over Na_2SO_4, filtered and the solvents were removed under reduced pressure. The crude brown product was coated on Celite. After dry loading, the crude product was purified via column chromatography using cHex/EtOAc 20:1 to EtOAc. A second purification was necessary: A solution of HCl (20 mL, 0.5 M) was added to the product with a small amount of EtOAc. The product precipitated as a salt and the residue of the precipitation was filtered. The expected product 4-(1-(quinoxalin-2-yl)-1*H*-1,2,3-triazol-4-

yl)benzenaminium chloride (69.2 mg, 213 µmol) was obtained as a green solid with 37% yield. Moreover, an unknown product (20 mg) was obtained.

R_f = 0.17 (cyclohexane/ethyl acetate 4:1). ^{1}H NMR (400 MHz, DMSO-d_6 [2.50 ppm], ppm) δ = 9.77 (s, 1H, C*H*), 9.59 (s, 1H, C*H*), 8.25 (d, *J* = 7.8 Hz, 1H, C*H*$_{Ar}$), 8.15 (d, *J* = 8.1 Hz, 3H, C*H*$_{Ar}$), 8.03–7.94 (m, 2H, C*H*$_{Ar}$), 7.41 (d, *J* = 8.1 Hz, 2H, C*H*$_{Ar}$), 3.73 (s, 3H, N*H*$_3$). ^{13}C NMR (100 MHz, DMSO-d_6 [39.5 ppm], ppm) δ = 147.4 (1C, Cq), 143.4 (1C, Cq), 142.1 (1C, Cq), 139.8 (1C, Cq), 138.7 (1C, *C*H), 135.5 (1C, Cq), 132.4 (1C, *C*H$_{Ar}$), 131.1 (1C, *C*H$_{Ar}$), 130.4 (1C, Cq), 129.7 (1C, *C*H$_{Ar}$), 128.9 (1C, *C*H$_{Ar}$), 127.5 (2C, *C*H$_{Ar}$), 122.8 (2C, *C*H$_{Ar}$), 119.1 (1C, *C*H); IR (ATR, ṽ) = 3336 (w), 2817 (w), 2591 (w), 1611 (w), 1564 (m), 1500 (vs), 1472 (s), 1449 (vs), 1428 (m), 1373 (w), 1357 (w), 1235 (m), 1215 (s), 1181 (w), 1140 (w), 1126 (m), 1094 (m), 1043 (w), 1004 (vs), 966 (m), 950 (s), 926 (w), 860 (w), 827 (w), 805 (vs), 764 (vs), 677 (w), 636 (w), 609 (m), 588 (s), 544 (s), 516 (vs), 460 (s), 418 (s), 390 (vs) cm^{-1}; HRMS ($C_{16}H_{12}N_6$): Calcd 288.1118, Found 288.1119. MS (EI, m/z, 70 eV, 200 °C): 288 [M]+ (15), 260 (100), 259 (21), 132 (8), 131 (59), 129 (12), 102 (13).

Additional information on the chemical synthesis is available via Chemotion repository: https://doi.org/10.14272/reaction/SA-FUHFF-UHFFFADPSC-GRLFMIQVCU-UHFFFADPSC-NUHFF-NUHFF-NUHFF-ZZZ

Additional information on the analysis of the target compound is available via Chemotion repository:
https://doi.org/10.14272/GRLFMIQVCULKIR-UHFFFAOYSA-N.1

***N,N*-dimethyl-4-(1-(quinoxalin-2-yl)-1*H*-1,2,3-triazol-4-yl)aniline (14d)**

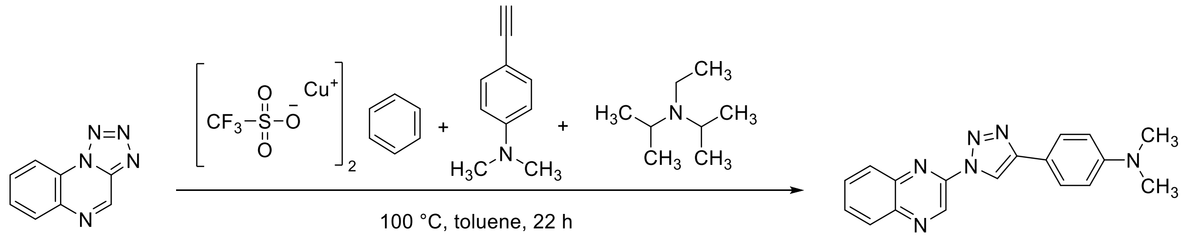

Name {P1|**14d**}: *N,N*-dimethyl-4-(1-(quinoxalin-2-yl)-1*H*-1,2,3-triazol-4-yl)aniline; Formula: $C_{18}H_{16}N_6$; Molecular Mass: 316.3598; Exact Mass: 316.1436; Smiles: CN(c1ccc(cc1)c1nnn(c1)c1cnc2c(n1)cccc2)C; InChIKey: TXQZSMLFWOHBGX-UHFFFAOYSA-N

The starting material tetrazolo[1,5-*a*]quinoxaline (101 mg, 588 µmol, 1.00 equiv), (4-ethynylphenyl)-dimethyl-amine (169 mg, 173 µL, 1.16 mmol, 1.98 equiv) and the catalyst benzene;copper(1+);trifluoromethanesulfonate (28.3 mg, 56.2 µmol, 0.0957 equiv) were dissolved in 2 mL of dry toluene under argon, followed by *N*-ethyl-*N*-propan-2-ylpropan-2-amine (152 mg, 200 µL, 1.18 mmol, 2.00 equiv). The brown reaction mixture was stirred at 100 °C for 22 hours. Then water and DCM were added and the brown aqueous phase was extracted 4 times with DCM. The combined organic phases were dried over Na_2SO_4, filtered and the solvents were removed under reduced pressure. The obtained crude product was twice purified via flash-chromatography (Interchim devices puriFLASH 5.125) on silica gel (PF-15SIHP-

F0012) using cHex to cHex/EtOAc 2:1 in 10 column volumes. The impure fraction was purified again via flash-chromatography (Interchim devices puriFLASH 5.125) on silica gel (PF-15SIHP-F0012) using DCM to EtOAc in 20 column volumes. The expected product *N,N*-dimethyl-4-(1-(quinoxalin-2-yl)-1*H*-1,2,3-triazol-4-yl)aniline (89.0 mg, 281 μmol) was obtained as an orange solid in 48% yield. Note: The reaction was repeated in larger scale with a yield of 63%.

R_f = 0.52 (cyclohexane/ethyl acetate 2:1). ^{1}H NMR (400 MHz, Chloroform-d [7.27 ppm], ppm) δ = 9.87 (s, 1H, C*H*), 8.79 (s, 1H, C*H*), 8.22–8.19 (m, 1H, CH_{Ar}), 8.09 (dd, *J* = 1.3 Hz, *J* = 8.1 Hz, 1H, CH_{Ar}), 7.87–7.79 (m, 4H, CH_{Ar}), 6.81 (d, *J* = 8.8 Hz, 2H, CH_{Ar}), 3.03 (s, 6H, CH_3) ^{13}C NMR (100 MHz, Chloroform-d [77.0 ppm], ppm) δ = 150.8 (1C, C_q), 149.0 (1C, C_q), 143.1 (1C, C_q), 142.1 (1C, C_q), 140.1 (1C, C_q), 137.9 (1C, *C*H), 131.4 (1C, CH_{Ar}), 129.9 (1C, CH_{Ar}), 129.5 (1C, CH_{Ar}), 128.7 (1C, CH_{Ar}), 127.0 (2C, CH_{Ar}), 117.6 (1C, C_q), 114.8 (1C, *C*H), 112.4 (2C, CH_{Ar}), 40.4 (2C, CH_3); HRMS ($C_{18}H_{16}N_6$): Calcd 316.1431, Found 316.1429 MS (EI, m/z, 70 eV, 150 °C): 316 [M]+ (24), 289 (22), 288 (100), 287 (23), 159 (18), 144 (18), 143 (30), 102 (13); IR (ATR, ṽ) = 2885 (w), 2809 (w), 1612 (s), 1571 (s), 1557 (w), 1500 (vs), 1479 (vs), 1448 (vs), 1429 (s), 1356 (vs), 1281 (m), 1213 (vs), 1193 (vs), 1166 (s), 1143 (s), 1128 (s), 1089 (m), 1061 (m), 1037 (m), 1010 (vs), 999 (vs), 963 (s), 948 (vs), 915 (s), 822 (vs), 799 (vs), 788 (vs), 758 (vs), 671 (s), 601 (vs), 582 (m), 541 (s), 528 (s), 516 (s), 487 (s), 416 (vs) cm^{-1}.

Additional information on the chemical synthesis is available via Chemotion repository: https://doi.org/10.14272/reaction/SA-FUHFF-UHFFFADPSC-TXQZSMLFWO-UHFFFADPSC-NUHFF-NUHFF-NUHFF-ZZZ

Additional information on the analysis of the target compound is available via Chemotion repository:
https://doi.org/10.14272/TXQZSMLFWOHBGX-UHFFFAOYSA-N.1

Methyl 4-(1-(quinoxalin-2-yl)-1*H*-1,2,3-triazol-4-yl)benzoate (14e), quinoxalin-2-amine (S10)

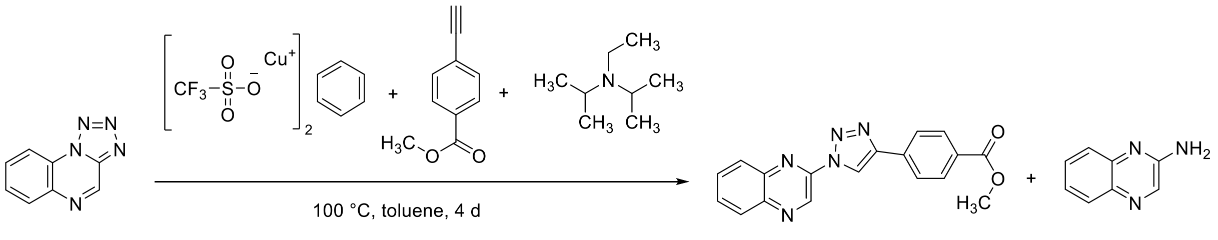

Name {P1|**14e**}: methyl 4-(1-(quinoxalin-2-yl)-1*H*-1,2,3-triazol-4-yl)benzoate; Formula: $C_{18}H_{13}N_5O_2$; Molecular Mass: 331.3281; Exact Mass: 331.1069; Smiles: COC(=O)c1ccc(cc1)c1nnn(c1)c1cnc2c(n1)cccc2; InChIKey: FEZUXVAXRREZEP-UHFFFAOYSA-N

Name {P2|**S10**}: quinoxalin-2-amine; Formula: $C_8H_7N_3$; Molecular Mass: 145.1613; Exact Mass: 145.0640; Smiles: Nc1cnc2c(n1)cccc2; InChIKey: YOWAEZWWQFSEJD-UHFFFAOYSA-N

The starting material tetrazolo[1,5-*a*]quinoxaline (99.3 mg, 573 µmol, 1.00 equiv), methyl 4-ethynylbenzoate (169 mg, 154 µL, 1.05 mmol, 1.82 equiv) and the catalyst benzene;copper(1+);trifluoromethanesulfonate (29.1 mg, 57.8 µmol, 0.0997 equiv) were dissolved in 2 mL of dry toluene under argon, followed by *N*-ethyl-*N*-propan-2-ylpropan-2-amine (153 mg, 201 µL, 1.18 mmol, 2.04 equiv). The green reaction mixture was stirred at 100 °C for 4 days. Then water and EtOAc were added and the aqueous phase was extracted 3 times with EtOAc. The combined organic phases were dried over Na_2SO_4, filtered and the solvents were removed under reduced pressure. The crude brown product was purified via column chromatography *c*Hex -> *c*Hex/EtOAc 2:1 (dryload on Celite). The expected product methyl 4-(1-(quinoxalin-2-yl)-1*H*-1,2,3-triazol-4-yl)benzoate (21.1 mg, 63.7 µmol) was obtained as a brown solid in 11% yield and quinoxalin-2-amine (24.3 mg, 167 µmol, 29% yield) was obtained as an impure side product. Moreover, an unknown product (24.0 mg) was obtained.

R_f = 0.21 (cyclohexane/ethyl acetate 2:1). ^{1}H NMR (400 MHz, Chloroform-d [7.27 ppm], ppm) δ = 9.89 (s, 1H, C*H*), 9.05 (s, 1H, C*H*), 8.25–8.22 (m, 1H, CH_{Ar}), 8.18–8.16 (m, 2H, CH_{Ar}), 8.12–8.07 (m, 3H, CH_{Ar}), 7.87 (m, 2H, CH_{Ar}), 3.96 (s, 3H, CH_3); ^{13}C NMR (100 MHz, Chloroform-d [77.0 ppm], ppm) δ = 166.6 (1C, *C*OOCH$_3$), 147.4 (1C, C_q), 144.6 (1C, C_q), 142.4 (1C, C_q), 140.0 (1C, C_q), 137.6 (1C, *C*H), 139.0 (1C, C_q), 131.6 (1C, *C*H$_{Ar}$), 130.4 (1C, C_q), 130.3 (2C, *C*H$_{Ar}$), 130.3 (1C, *C*H$_{Ar}$), 129.6 (1C, *C*H$_{Ar}$), 128.7 (1C, *C*H$_{Ar}$), 125.9 (2C, *C*H$_{Ar}$), 117.7 (1C, *C*H), 52.2 (1C, *C*H$_3$); MS (EI, m/z, 70 eV, 150 °C): 331 $[M]^+$ (4), 318 (39), 306 (22), 304 (26), 303 (100), 302 (13), 287 (22), 272 (16), 244 (19), 243 (10), 145 (20), 130 (10), 129 (90), 102 (41). HRMS ($C_{18}H_{13}O_2N_5$): Calcd 331.1064, Found 331.1063; IR (ATR, ṽ) = 3138 (w), 2949 (w), 1717 (vs), 1612 (w), 1561 (w), 1502 (m), 1475 (w), 1451 (m), 1438 (m), 1414 (m), 1276 (vs), 1239 (s), 1215 (s), 1198 (m), 1188 (m), 1145 (m), 1111 (s), 1041 (m), 1006 (vs), 966 (m), 950 (s), 932 (m), 860 (s), 822 (s), 768 (vs), 713 (s), 696 (s), 679 (m), 649 (w), 630 (w), 595 (m), 535 (m), 510 (m), 499 (m), 470 (w), 415 (s) cm^{-1}.

^{1}H NMR (400 MHz, $CDCl_3$, ppm) δ = 8.83 (s, 1H, NCH_{ar}), 7.92 (d, ^3J = 8.2 Hz, 1H, CH_{ar}), 7.68–7.59 (m, 2H, CH_{ar}), 7.47–7.42 (m, 1H, CH_{ar}), 5.05 (bs, 2H, NH_2).

Additional information on the chemical synthesis is available via Chemotion repository: https://doi.org/10.14272/reaction/SA-FUHFF-UHFFFADPSC-ZKYSXYLHQL-UHFFFADPSC-NUHFF-NUHFF-NUHFF-ZZZ

Additional information on the analysis of the target compound is available via Chemotion repository:
https://doi.org/10.14272/FEZUXVAXRREZEP-UHFFFAOYSA-N.2
https://doi.org/10.14272/YOWAEZWWQFSEJD-UHFFFAOYSA-N.2

The synthesis of the side product quinoxalin-2-amine has been previously reported in literature [24].

2-(4-(4-Ethynylphenyl)-1*H*-1,2,3-triazol-1-yl)quinoxaline (**14f**)

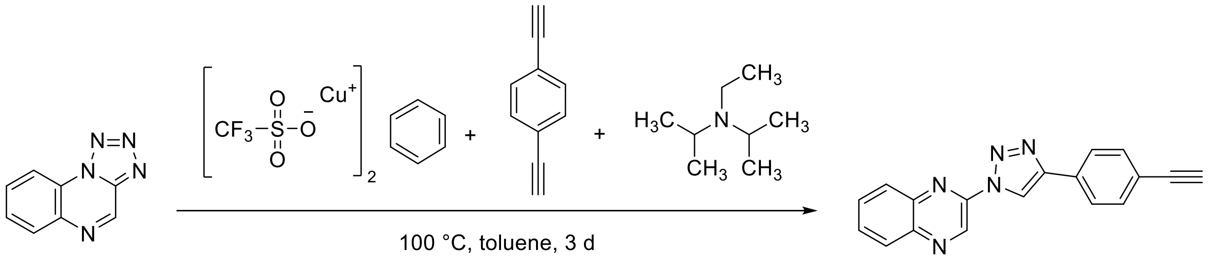

Name {P1|**14f**}: 2-(4-(4-ethynylphenyl)-1*H*-1,2,3-triazol-1-yl)quinoxaline; Formula: $C_{18}H_{11}N_5$; Molecular Mass: 297.3134; Exact Mass: 297.1014; Smiles: C#Cc1ccc(cc1)c1nnn(c1)c1cnc2c(n1)cccc2; InChIKey: UFXVPOPCEVYKQB-UHFFFAOYSA-N

The starting material tetrazolo[1,5-*a*]quinoxaline (50.0 mg, 292 µmol, 1.00 equiv), 1,4-diethynylbenzene (77.0 mg, 610 µmol, 2.09 equiv) and the catalyst benzene;copper(1+);trifluoromethanesulfonate (14.7 mg, 29.2 µmol, 0.100 equiv) were dissolved in 1.5 mL of dry toluene under nitrogen, followed by *N*-ethyl-*N*-propan-2-ylpropan-2-amine (113 mg, 153 µL, 876 µmol, 3.00 equiv). The reaction mixture was stirred at 100 °C for 3 d. Then water and ethyl acetate were added, the organic phase was separated and the aqueous phase was extracted 3x with ~30 mL of DCM each. The combined organic phases were dried over Na_2SO_4, filtered and the solvent was removed under reduced pressure. The crude product was purified via column chromatography (dryload on Celite, *c*Hex -> *c*Hex/EtOAc 1:1) and 2-(4-(4-ethynylphenyl)-1*H*-1,2,3-triazol-1-yl)quinoxaline (28.0 mg, 94.2 µmol, 32% yield) was obtained as a light yellow solid.

R_f = 0.43 (cyclohexane/ethyl acetate 4:1). ^{1}H NMR (400 MHz, $CDCl_3$, ppm) δ = 9.86 (s, 1H, NCH_{ar}), 8.95 (s, 1H, $CH_{triazole}$), 8.23–8.20 (m, 1H, CH_{ar}), 8.10–8.07 (m, 1H, CH_{ar}), 7.95 (m, 2H, CH_{phenyl}), 7.89–7.81 (m, 2H, CH_{ar}), 7.61 (m, 2H, CH_{phenyl}), 3.17 (s, 1H, CH_{alkyne}); ^{13}C NMR (100 MHz, $CDCl_3$ [77.0 ppm], ppm) δ = 147.7 (1C, $C_{triazole}$), 142.8 (1C, C_q), 142.3 (1C, C_q), 140.0 (1C, C_q), 137.6 (1C, NCH_{ar}), 132.7 (2C, CH_{phenyl}), 131.6 (1C, CH_{ar}), 130.3 (1C, CH_{ar}), 130.0 (1C, C_q), 129.6 (1C, CH_{ar}), 128.7 (1C, CH_{ar}), 125.9 (2C, CH_{phenyl}), 122.5 (1C, C_q), 117.1 (1C, $CH_{triazole}$), 83.3 (1C, C_{alkyne}), 78.3 (1C, CH_{alkyne}); MS (EI, 70 eV, 120 °C), m/z (%): 297 $[M]^+$ (11), 270 (24), 269 (100), 268 (31), 141 (12), 129 (52), 102 (35). HRMS (EI, $C_{18}H_{11}N_5$): calcd 297.1009, found 297.1010. IR (ATR, ṽ) = 3268 (m), 3128 (w), 3047 (w), 2922 (w), 1561 (m), 1499 (vs), 1472 (s), 1449 (vs), 1417 (w), 1388 (w), 1361 (w), 1326 (w), 1286 (w), 1272 (w), 1238 (vs), 1217 (s), 1188 (m), 1142 (w), 1128 (w), 1091 (m), 1047 (w), 1007 (vs), 966 (m), 949 (vs), 919 (m), 840 (m), 824 (vs), 761 (vs), 734 (w), 703 (s), 676 (m), 635 (s), 599 (s), 543 (vs), 531 (m), 511 (w), 442 (m), 411 (s) cm^{-1}.

Additional information on the chemical synthesis is available via Chemotion repository: https://doi.org/10.14272/reaction/SA-FUHFF-UHFFFADPSC-UFXVPOPCEV-UHFFFADPSC-NUHFF-NUHFF-NUHFF-ZZZ

Additional information on the analysis of the target compound is available via Chemotion repository: https://doi.org/10.14272/UFXVPOPCEVYKQB-UHFFFAOYSA-N.1

2-(4-(3-Methoxyphenyl)-1*H*-1,2,3-triazol-1-yl)quinoxaline (14g)

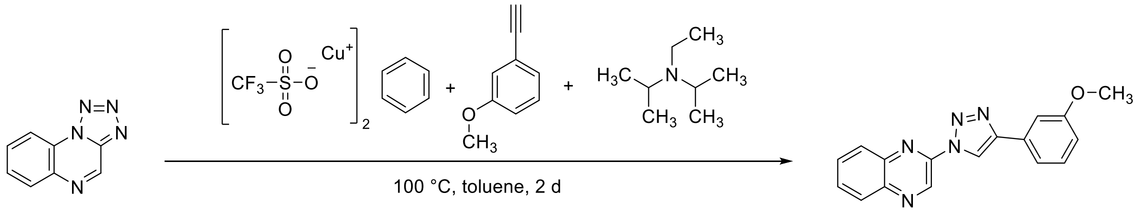

Name {P1|**14g**}: 2-(4-(3-methoxyphenyl)-1*H*-1,2,3-triazol-1-yl)quinoxaline; Formula: $C_{17}H_{13}N_5O$; Molecular Mass: 303.3180; Exact Mass: 303.1120; Smiles: COc1cccc(c1)c1nnn(c1)c1cnc2c(n1)cccc2; InChIKey: MXGSQRZNSJHGEP-UHFFFAOYSA-N

The starting material tetrazolo[1,5-*a*]quinoxaline (102 mg, 587 µmol, 1.00 equiv) and the catalyst benzene;copper(1+);trifluoromethanesulfonate (28.5 mg, 56.6 µmol, 0.0965 equiv) were dissolved in 2 mL of dry toluene under argon, followed by 1-ethynyl-3-methoxybenzene (150 mg, 150 µL, 1.13 mmol, 1.93 equiv) and *N*-ethyl-*N*-propan-2-ylpropan-2-amine (153 mg, 201 µL, 1.18 mmol, 2.06 equiv). The green reaction mixture was stirred at 100 °C for 2 days. Then water and EtOAc were added and the brown solution was extracted 3 times with EtOAc. The combined organic phases were dried over Na_2SO_4, filtered and the solvents were removed under reduced pressure. The crude brown product was purified via column chromatography using cHex to EtOAc (dryload on Celite) and the expected product 2-(4-(3-methoxyphenyl)-1*H*-1,2,3-triazol-1-yl)quinoxaline (65.5 mg, 216 µmol) was obtained as a white solid in 36% yield. Note: This reaction was repeated with a yield of 70%.

R_f = 0.43 (cyclohexane/ethyl acetate 2:1). ^{1}H NMR (400 MHz, Chloroform-d [7.27 ppm], ppm) δ = 9.89 (s, 1H, C*H*), 8.95 (s, 1H, C*H*), 8.24–8.22 (m, 1H, CH_{Ar}), 8.12–8.09 (m, 1H, CH_{Ar}), 7.90–7.82 (m, 2H, CH_{Ar}), 7.60–7.59 (m, 1H, CH_{Ar}), 7.56–7.54 (m, 1H, CH_{Ar}), 7.41 (t, *J* = 7.8 Hz, 1H, CH_{Ar}), 6.96 (ddd, *J* = 1.0 Hz, *J* = 2.7 Hz, *J* = 8.3 Hz, 1H, CH_{Ar}), 3.92 (s, 3H, CH_3); ^{13}C NMR (100 MHz, Chloroform-d [77.0 ppm], ppm) δ = 160.1 (1C, C_q), 148.4 (1C, C_q), 142.2 (1C, C_q), 140.0 (1C, C_q), 138.4 (1C, C_q), 137.7 (1C, CH), 131.5 (1C, CH_{Ar}), 130.9 (1C, C_q), 130.2 (1C, CH_{Ar}), 130.0 (1C, CH_{Ar}), 129.6 (1C, CH_{Ar}), 128.7 (1C, CH), 118.5 (1C, CH_{Ar}), 116.9 (1C, CH_{Ar}), 114.9 (1C, CH_{Ar}), 111.1 (1C, CH_{Ar}), 55.4 (1C, CH_3); MS (EI, m/z, 70 eV, 130 °C): 303 $[M]^+$ (11), 276 (27), 275(98), 274 (43), 245 (10), 244 (11), 232 (11), 231 (11), 147 (21), 130 (17), 129 (100), 116 (10), 103 (15), 102 (80), 89 (15), 86 (10). HRMS ($C_{17}H_{13}O_1N_5$): Calcd 303.1115, Found 303.1114; IR (ATR, ṽ) = 3150 (w), 2961 (w), 2837 (w), 1615 (w), 1581 (s), 1567 (m), 1496 (vs), 1475 (vs), 1451 (vs), 1439 (s), 1428 (s), 1364 (m), 1327 (w), 1288 (m), 1245 (vs), 1208 (vs), 1171 (vs), 1133 (s), 1082 (m), 1047 (vs), 1009 (vs), 977 (s), 950 (vs), 918 (s), 887 (s), 833 (s), 809 (m), 785 (vs), 764 (vs), 714 (m), 688 (vs), 647 (m), 626 (m), 588 (s), 569 (s), 483 (s), 460 (s), 412 (vs) cm^{-1}.

Additional information on the chemical synthesis is available via Chemotion repository: https://doi.org/10.14272/reaction/SA-FUHFF-UHFFFADPSC-MXGSQRZNSJ-UHFFFADPSC-NUHFF-NUHFF-NUHFF-ZZZ

Additional information on the analysis of the target compound is available via Chemotion repository:
https://doi.org/10.14272/MXGSQRZNSJHGEP-UHFFFAOYSA-N.1

3-(1-(Quinoxalin-2-yl)-1*H*-1,2,3-triazol-4-yl)benzenaminium (14h)

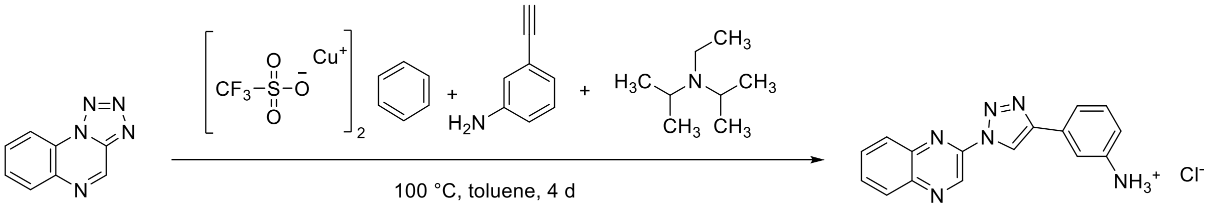

Name {P1|**14h**}: 3-(1-(quinoxalin-2-yl)-1*H*-1,2,3-triazol-4-yl)benzenaminium; Formula: $C_{16}H_{13}ClN_6$; Molecular Mass: 324.7676; Exact Mass: 324.0890; Smiles: [NH3+]c1cccc(c1)c1nnn(c1)c1cnc2c(n1)cccc2.[Cl-]; InChIKey: KUVSJEDELBLMHP-UHFFFAOYSA-N

The starting material tetrazolo[1,5-*a*]quinoxaline (100 mg, 584 µmol, 1.00 equiv) and the catalyst benzene;copper(1+);trifluoromethanesulfonate (25.6 mg, 50.9 µmol, 0.0871 equiv) were dissolved in 2 mL of dry toluene under argon, followed by 3-ethynylaniline (137 mg, 130 µL, 1.17 mmol, 2.00 equiv) and *N*-ethyl-*N*-propan-2-ylpropan-2-amine (152 mg, 200 µL, 1.18 mmol, 2.01 equiv). The dark reaction mixture was stirred at 100 °C for 4 days. Then water and DMC were added and the brown aqueous phase was extracted 4 times with DCM. The combined organic phases were dried over Na_2SO_4, filtered and the solvents were removed under reduced pressure. The obtained crude product was purified via flash-chromatography on silica gel using cHex to EtOAc. An unknown product (12 mg) as well as a mixture of the reduced product quinoxalin-2-amine and the starting material (44 mg) were obtained. The product 3-(1-(quinoxalin-2-yl)-1*H*-1,2,3-triazol-4-yl)aniline (66 mg) was obtained with impurities and further precipitated as a salt via addition of an aqueous solution of HCl (40 mL, 0.5 M) together with a small amount of DCM. The formed solid residue of the precipitation was filtered and 3-(1-(quinoxalin-2-yl)-1*H*-1,2,3-triazol-4-yl)benzenaminium (62.1 mg, 191 µmol) was obtained as a white-yellow solid in 33% yield.

R_f = 0.33 (CH_2Cl_2/MeOH 20:1). ^{1}H NMR (400 MHz, DMSO-d6 [2.50 ppm], ppm) δ = 9.78 (s, 1H, C*H*), 9.66 (s, 1H, C*H*), 8.26 (dd, *J* = 1.3 Hz, *J* = 8.1 Hz, 1H, CH_{Ar}), 8.18 (dd, *J* = 1.3 Hz, *J* = 8.3 Hz, 1H, CH_{Ar}), 8.04–7.95 (m, 4H, CH_{Ar}), 7.59 (t, *J* = 7.8 Hz, 1H, CH_{Ar}), 7.34 (d, *J* = 7.7 Hz, 1H, CH_{Ar}), 3.81 (bs, 3H, NH_3); 13C NMR (100 MHz, DMSO-d_6, [39.5 ppm], ppm) δ = 146.5 (1C, C_q), 142.8 (1C, C_q), 141.6 (1C, C_q), 139.27 (1C, C_q), 138.18 (1C, CH_{ar}), 134.19 (1C, C_q), 131.95 (1C, CH_{ar}), 130.96 (1C, C_q), 130.68 (1C, CH_{ar}), 130.48 (1C, CH_{ar}), 129.16 (1C, CH_{ar}), 128.45 (1C, CH_{ar}), 124.50 (1C, CH_{ar}), 122.59 (1C, CH_{ar}), 119.29 (2C, CH_{ar}); HRMS ($C_{16}H_{12}N_6$): calcd 288.1118, found 288.1119 (Counterion Cl not observed due to positive ionization mode) MS (EI, 70 eV, m/z, 160 °C): 289 [M]+ (7), 288 (31), 261 (14), 260 (100), 259 (58), 234 (7), 233 (10), 132 (24), 131 (18), 130 (11), 129 (50), 104 (11), 102 (41); IR (ATR, ṽ) = 2850 (m), 2601 (w), 1598 (w), 1568 (m), 1502 (vs), 1473 (s), 1446 (vs), 1373 (w), 1244 (w), 1225 (s), 1174 (w), 1140 (m), 1103 (w), 1045 (w), 1016 (vs), 977 (m), 950 (s), 919 (w), 861 (w), 839 (w), 792 (m), 768 (vs), 715 (w), 686 (m), 674 (m), 647 (w), 594 (m), 544 (w), 531 (w), 517 (w), 469 (w), 441 (s), 412 (s) cm^{-1}.

Additional information on the chemical synthesis is available via Chemotion repository: https://doi.org/10.14272/reaction/SA-FUHFF-UHFFFADPSC-KUVSJEDELB-UHFFFADPSC-NUHFF-NUHFF-NUHFF-ZZZ

Additional information on the analysis of the target compound is available via Chemotion repository:
https://doi.org/10.14272/KUVSJEDELBLMHP-UHFFFAOYSA-N.1

2-((1-(Quinoxalin-2-yl)-1*H*-1,2,3-triazol-4-yl)methyl)isoindoline-1,3-dione (14i)

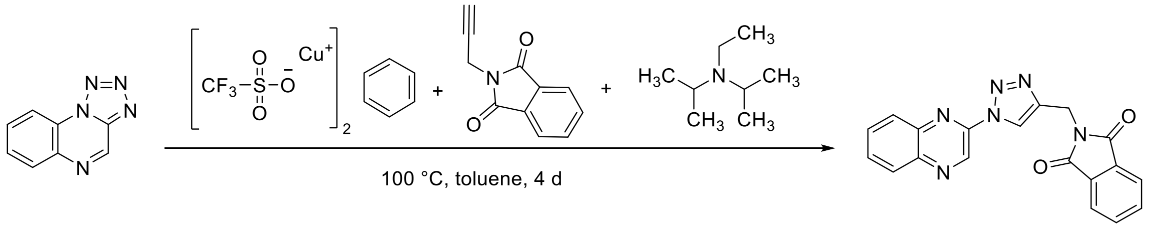

Name {P1|**14i**}: 2-((1-(quinoxalin-2-yl)-1*H*-1,2,3-triazol-4-yl)methyl)isoindoline-1,3-dione; Formula: $C_{19}H_{12}N_6O_2$; Molecular Mass: 356.3376; Exact Mass: 356.1022; Smiles: O=C1N(Cc2nnn(c2)c2cnc3c(n2)cccc3)C(=O)c2c1cccc2; InChIKey: GCJKJVPZVSPCQI-UHFFFAOYSA-N

The starting material tetrazolo[1,5-*a*]quinoxaline (101 mg, 592 µmol, 1.00 equiv), 2-prop-2-ynylisoindole-1,3-dione (214 mg, 161 µL, 1.16 mmol, 1.95 equiv) and the catalyst benzene;copper(1+);trifluoromethanesulfonate (29.6 mg, 58.8 µmol, 0.0993 equiv) were dissolved in 2 mL of dry toluene under argon, followed by *N*-ethyl-*N*-propan-2-ylpropan-2-amine (153 mg, 201 µL, 1.18 mmol, 1.99 equiv). The brown reaction mixture was stirred at 100 °C for 4 days until TLC indicated complete conversion of the starting material. Then water and EtOAc were added and the brown solution was extracted 3 times with EtOAc. The combined organic phases were dried over Na_2SO_4, filtered and the solvent was removed under reduced pressure. The crude brown product was purified thrice using column chromatography (dryload on Celite, cHex -> cHex/EtOAc 2:1, DCM -> DCM/MeOH 50:1, DCM -> DCM/MeOH 10:1). The expected product 2-((1-(quinoxalin-2-yl)-1*H*-1,2,3-triazol-4-yl)methyl)isoindoline-1,3-dione (68.7 mg, 193 µmol) was obtained as a brown solid in 33% yield.

R_f = 0.23 (cyclohexane/ethyl acetate 2:1). ^{1}H NMR (400 MHz, Chloroform-d [7.27 ppm], ppm) δ = 9.80 (s, 1H, C*H*), 8.78 (s, 1H, C*H*), 8.21–8.19 (m, 1H, CH_{Ar}), 8.07–8.04 (m, 1H, CH_{Ar}), 7.91–7.90 (m, 2H, CH_{Ar}), 7.84 (ddd, *J* = 1.7 Hz, *J* = 6.3 Hz, *J* = 7.5 Hz, 2H, CH_{Ar}), 7.77–7.73 (m, 2H, CH_{Ar}), 5.16 (s, 2H, CH_2); ^{13}C NMR (100 MHz, Chloroform-d [77.0 ppm], ppm) δ = 167.6 (2C, *C*O), 143.8 (2C, C_q), 142.7 (1C, C_q), 142.2 (1C, C_q), 139.9 (1C, C_q), 137.6 (1C, CH), 134.2 (1C, CH_{Ar}), 132.0 (1C, C_q), 131.5 (1C, CH_{Ar}), 130.3 (1C, CH_{Ar}), 129.5 (1C, CH_{Ar}), 128.7 (2C, CH_{Ar}), 123.5 (2C, CH_{Ar}), 120.5 (1C, *C*H), 33.0 (1C, CH_2); HRMS ($C_{19}H_{13}O_2N_6$): Calcd 357.1095, Found 357.1095. MS (FAB, 3-NBA, m/z): 358 [M]+ (18), 357 (62), 307 (16), 289 (14), 182 (15), 160 (15), 156 (10), 155 (36), 154 (100), 153 (12), 152 (13), 139 (23), 138 (45), 137 (76), 136 (87), 129 (24), 121 (17), 120 (17), 119 (16), 109 (16), 107 (33), 105 (18), 97 (24), 95 (29), 91 (32), 90 (18), 89 (25); IR (ATR, ṽ) = 3131 (w), 1772 (w), 1713 (vs), 1612 (w), 1561 (w), 1496 (m), 1466 (w), 1448 (w), 1415 (s), 1395 (s), 1371 (m), 1339 (s), 1305 (m), 1234 (s), 1177 (m), 1136 (w), 1126 (w), 1098 (m), 1085 (m), 1035 (s), 1016 (w), 997 (m), 949 (m), 931 (vs), 846 (w), 836 (w), 789 (w), 762 (vs), 713 (vs), 676 (s), 649 (s), 618 (m), 591 (m), 548 (w), 530 (vs), 409 (vs), 382 (m) cm^{-1}.

Additional information on the chemical synthesis is available via Chemotion repository: https://doi.org/10.14272/reaction/SA-FUHFF-UHFFFADPSC-GCJKJVPZVS-UHFFFADPSC-NUHFF-NUHFF-NUHFF-ZZZ

Additional information on the analysis of the target compound is available via Chemotion repository:
https://doi.org/10.14272/GCJKJVPZVSPCQI-UHFFFAOYSA-N.1

But-3-ynyl 4-methylbenzenesulfonate (**4j**)

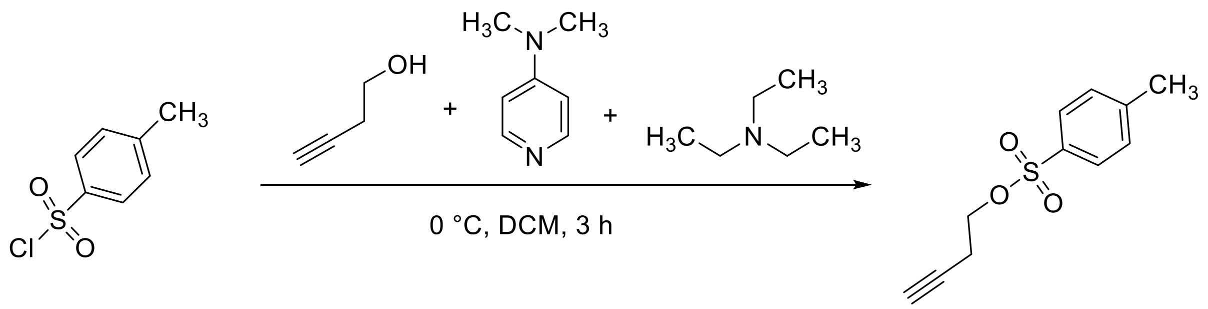

Name {P1|**4j**}: but-3-ynyl 4-methylbenzenesulfonate; Formula: $C_{11}H_{12}O_3S$; Molecular Mass: 224.2762; Exact Mass: 224.0507; Smiles: C#CCCOS(=O)(=O)c1ccc(cc1)C; InChIKey: STOASOOVVADOKH-UHFFFAOYSA-N

The starting material 4-methylbenzenesulfonyl chloride (1.38 g, 7.22 mmol, 1.00 equiv) as well as but-3-yn-1-ol (509 mg, 550 µL, 7.27 mmol, 1.01 equiv), *N,N*-dimethylpyridin-4-amine (87.2 mg, 713 µmol, 0.100 equiv) and triethylamine (722 mg, 994 µL, 7.13 mmol, 1.00 equiv) were dissolved in 10 mL of DCM and stirred at 0 °C for 3 h. Water and DCM were added and the reaction was extracted 3x with DCM. The combined organic phases were dried over Na_2SO_4, filtered and the solvent was removed under reduced pressure. The crude product was purified via column chromatography (dryload on celite, eluent *c*Hex/EtOAc 4:1) and but-3-ynyl 4-methylbenzenesulfonate (1.37 g, 6.13 mmol, 85% yield) was obtained as a colourless oil.

R_f = 0.63 (cyclohexane/ethyl acetate 2:1). ^{1}H NMR (400 MHz, $CDCl_3$, ppm) δ = 7.81–7.79 (m, 2H, CH_{ar}), 7.36–7.34 (m, 2H, CH_{ar}), 4.10 (t, ^3J = 7.0 Hz, 2H, OCH_2), 2.55 (td, ^3J = 7.0 Hz, ^4J = 2.7 Hz, 2H, C$H_2$$C_{alkyne}$), 2.45 (2, 3H, C$H_3$), 1.97 (t, ^4J = 2.7 Hz, 1H, CH_{alkyne}); ^{13}C NMR (100 MHz, $CDCl_3$, ppm) δ = 145.0 (1C, C_q), 132.9 (1C, C_q), 129.9 (2C, *C*H_{ar}), 128.0 (2C, *C*H_{ar}), 78.3 (1C, C_q), 70.7 (1C, *C*H_{alkyne}), 67.4 (1C, O*C*H_2), 21.6 (1C, *C*H_3), 19.4 (1C, *C*$H_2$$C_{alkyne}$). MS (EI, 70 eV, 40 °C), m/z (%): 224 $[M]^+$ (12), 185 (14), 172 (11), 155 (100), 91 (78), 65 (15). HRMS (EI, $C_{11}H_{12}O_3{}^{32}S_1$): calcd 224.0502, found 224.0501. IR (ATR, ṽ) = 3288 (w), 1598 (w), 1494 (w), 1459 (w), 1356 (vs), 1307 (w), 1292 (w), 1220 (w), 1188 (s), 1173 (vs), 1120 (w), 1096 (s), 1071 (w), 1020 (m), 976 (vs), 902 (vs), 815 (vs), 765 (vs), 686 (s), 662 (vs), 585 (m), 554 (vs), 490 (m) cm^{-1}.

Additional information on the chemical synthesis is available via Chemotion repository: https://doi.org/10.14272/reaction/SA-FUHFF-UHFFFADPSC-STOASOOVVA-UHFFFADPSC-NUHFF-NUHFF-NUHFF-ZZZ

Additional information on the analysis of the target compound is available via Chemotion repository:
https://doi.org/10.14272/STOASOOVVADOKH-UHFFFAOYSA-N.1

The synthesis of this compound has been previously described and the ^{1}H NMR data corresponds with the literature [25].

2-(1-(Quinoxalin-2-yl)-1*H*-1,2,3-triazol-4-yl)ethyl 4-methylbenzenesulfonate (14j)

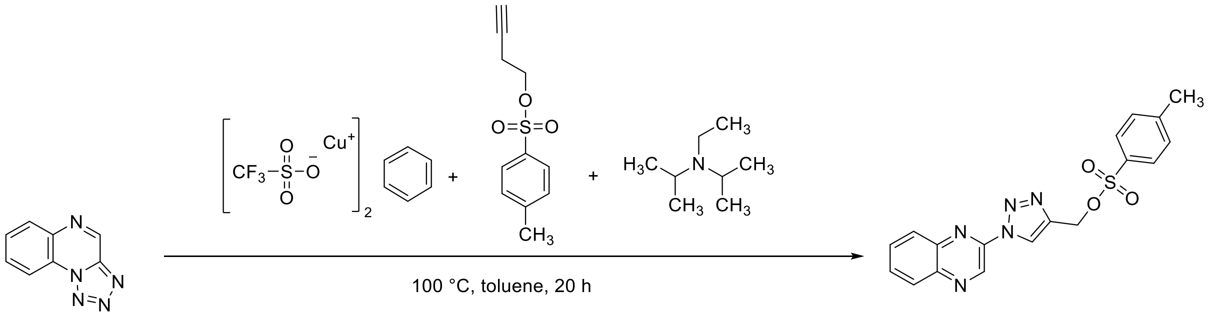

Name {P1|**14j**}: 2-(1-(quinoxalin-2-yl)-1*H*-1,2,3-triazol-4-yl)ethyl 4-methylbenzenesulfonate; Formula: $C_{19}H_{17}N_5O_3S$; Molecular Mass: 395.4350; Exact Mass: 395.1052; Smiles: Cc1ccc(cc1)S(=O)(=O)OCCc1nnn(c1)c1cnc2c(n1)cccc2; InChIKey: UTQXOLZOCMOFPG-UHFFFAOYSA-N

The starting material tetrazolo[1,5-*a*]quinoxaline (51.0 mg, 298 µmol, 1.00 equiv), but-3-ynyl 4-methylbenzenesulfonate (131 mg, 584 µmol, 1.96 equiv) and the catalyst benzene;copper(1+);trifluoromethanesulfonate (15.0 mg, 29.8 µmol, 0.100 equiv) were dissolved in 1 mL of dry toluene under argon, followed by addition of diisopropylamine (88.7 mg, 123 µL, 876 µmol, 2.94 equiv). The reaction mixture was stirred at 100 °C for 20 h; then water and EtOAc were added, the organic phase was separated and the aqueous phase was extracted 3× with EtOAc. The combined organic phases were dried over Na_2SO_4, filtered and the solvent was removed under reduced pressure. The crude product was purified via column chromatography (dryload on Celite, eluent *c*Hex/EtOAc 1:2) and 2-(1-(quinoxalin-2-yl)-1*H*-1,2,3-triazol-4-yl)ethyl 4-methylbenzenesulfonate (88.0 mg, 223 µmol, 75% yield) was obtained as a light brown solid.

R_f = 0.27 (cyclohexane/ethyl acetate 2:1). ^{1}H NMR (400 MHz, $CDCl_3$, ppm) δ = 9.79 (s, 1H, NCH_{ar}), 8.56 (s, 1H, NC$H_{triazole}$), 8.21 (dd, ^{3}J = 8.3 Hz, ^{4}J = 1.7 Hz, 1H, CH_{ar}), 8.09 (dd, ^{3}J = 8.3 Hz, ^{4}J = 1.7 Hz, 1H, CH_{ar}), 7.90–7.82 (m, 2H, CH_{ar}), 7.77–7.75 (m, 2H, CH_{tosyl}), 7.29–7.27 (m, 2H, CH_{tosyl}), 4.41 (t, ^{3}J = 6.4 Hz, 2H, CH_2), 3.24 (t, ^{3}J = 6.3 Hz, 2H, CH_2), 2.38 (s, 3H, CH_3); ^{13}C NMR (100 MHz, $CDCl_3$ [77.0 ppm], ppm) δ = 144.9 (1C, $C_{tosyl}CH_3$), 143.8 (1C, NCH$C_{triazole}$), 142.8, 142.2, 140.0, 137.6 (1C, NCH_{ar}), 132.7 (1C, O_3SC_q), 131.6 (1C, CH_{ar}), 130.3 (1C, CH_{ar}), 129.9 (2C, CH_{tosyl}), 129.6 (1C, CH_{ar}), 128.8 (1C, CH_{ar}), 127.9 (2C, CH_{tosyl}), 119.8 (1C, N$CH_{triazole}$), 68.6 (1C, CH_2), 25.9 (1C, CH_2), 21.6 (1C, CH_3); MS (FAB, 3-NBA), m/z (%): 397 (29), 396 (100) $[M]^+$, 155 (22), 154 (68), 138 (24), 137 (46), 136 (50), 129 (18). HRMS (FAB, $C_{19}H_{18}O_3N_5{}^{32}S_1$): Calcd 396.1125, Found 396.1126; IR (ATR, ṽ) = 3139 (w), 1596 (w), 1568 (w), 1500 (s), 1473 (w), 1451 (s), 1349 (vs), 1309 (m), 1235 (s), 1186 (s), 1164 (vs), 1136 (m), 1128 (w), 1096 (m), 1065 (w), 1054 (w), 1034 (s), 1016 (w), 1004 (s),

977 (vs), 953 (vs), 914 (vs), 837 (w), 819 (vs), 807 (s), 779 (vs), 764 (vs), 722 (m), 707 (w), 681 (w), 676 (w), 663 (vs), 649 (m), 613 (w), 586 (m), 577 (vs), 554 (vs), 543 (m), 506 (m), 477 (s), 462 (m), 416 (vs), 392 (m) cm^{-1}.

Additional information on the chemical synthesis is available via Chemotion repository: https://doi.org/10.14272/reaction/SA-FUHFF-UHFFFADPSC-UTQXOLZOCM-UHFFFADPSC-NUHFF-NUHFF-NUHFF-ZZZ

Additional information on the analysis of the target compound is available via Chemotion repository:
https://doi.org/10.14272/UTQXOLZOCMOFPG-UHFFFAOYSA-N.1

N,N*-diethyl-2-(1-(quinoxalin-2-yl)-1*H*-1,2,3-triazol-4-yl)ethan-1-amine** (**14j)

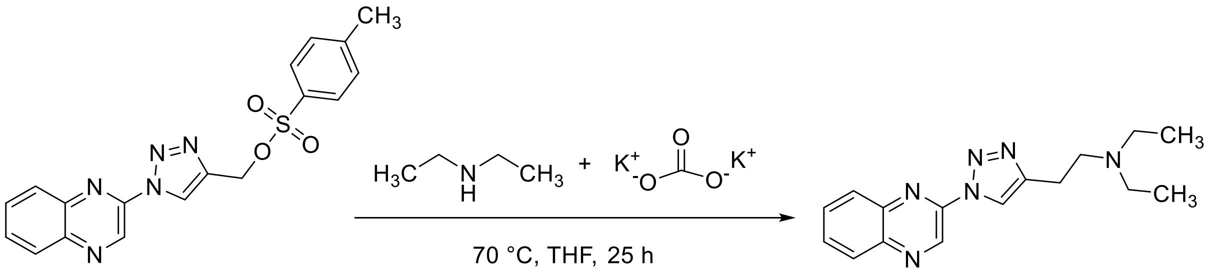

Name {P1|**14j***}: *N,N*-diethyl-2-(1-(quinoxalin-2-yl)-1*H*-1,2,3-triazol-4-yl)ethan-1-amine; Formula: $C_{16}H_{20}N_6$; Molecular Mass: 296.3702; Exact Mass: 296.1749; Smiles: CCN(CCc1nnn(c1)c1cnc2c(n1)cccc2)CC; InChIKey: CGUDGUUWCJKDIE-UHFFFAOYSA-N

The starting material 2-(1-(quinoxalin-2-yl)-1*H*-1,2,3-triazol-4-yl)ethyl 4-methylbenzenesulfonate (66.0 mg, 167 µmol, 1.00 equiv) was dissolved in 2 mL of THF and dipotassium;carbonate (24.0 mg, 174 µmol, 1.04 equiv) and diethylamine (48.1 mg, 658 µmol, 4.00 equiv) were added. The reaction mixture was heated to 70 °C for 25 h, then water and EtOAc were added and the organic phase was separated. The aqueous phase was extracted 3x with EtOAc, the combined organic phases were dried over Na_2SO_4, filtered and the solvent was removed under reduced pressure. The crude product was purified via column chromatography (dryload on Celite, eluent *c*Hex/EtOAc 1:1 +2% Et_3N) and *N,N*-diethyl-2-(1-(quinoxalin-2-yl)-1*H*-1,2,3-triazol-4-yl)ethan-1-amine (38.0 mg, 128 µmol, 77% yield) was obtained as a yellow solid.

R_f = 0.16 (CH_2Cl_2/MeOH 10:1). ^{1}H NMR (400 MHz, $CDCl_3$, ppm) δ = 9.81 (s, 1H, NC*H*CN), 8.54 (s, 1H, C$H_{triazole}$), 8.20–8.17 (m, 1H, CH_{ar}), 8.06–8.04 (m, 1H, CH_{ar}), 7.86–7.78 (m, 2H, CH_{ar}), 3.03–3.00 (m, 2H, CH_2), 2.91–2.87 (m, 2H, CH_2), 2.64 (q, ^3J = 7.2 Hz, 4H, C$H_2$$CH_3$), 1.08 (t, ^3J = 7.2 Hz, 6H, CH_3); ^{13}C NMR (100 MHz, $CDCl_3$ [77.0 ppm], ppm) δ = 147.6 (1C, C_q), 143.1 (1C, C_q), 142.1 (1C, C_q), 140.0 (1C, C_q), 137.8 (1C, NCHCN), 131.4 (1C, CH_{ar}), 130.0 (1C, CH_{ar}), 129.5 (1C, CH_{ar}), 128.7 (1C, CH_{ar}), 118.8 (1C, $CH_{triazole}$), 52.1 (1C, CH_2), 46.9 (2C, CH_2CH_3), 23.6 (1C, CH_2), 11.8 (2C, CH_3); MS (EI, 70 eV, 100 °C), m/z (%): 296 (2) $[M]^+$, 129 (4), 102 (3), 86 (100), 58 (4). HRMS (EI, $C_{16}H_{20}N_6$): calcd 296.1744, found 296.1742; IR (ATR, ṽ) = 3452 (vw), 3142 (w), 2966 (m), 2931 (w), 2873 (w), 2798 (w), 1612 (vw), 1561 (w), 1499 (s), 1475 (m), 1449 (s), 1373 (m), 1357 (m), 1330 (w), 1289 (w), 1237 (m), 1218 (m), 1204 (m), 1181 (s), 1137 (w), 1125 (w), 1065 (m), 1037 (s), 1016 (m), 992 (s), 949 (vs), 921 (m), 870 (w), 863 (w), 826 (m), 790 (m), 758 (vs), 737 (m), 698 (w), 676 (m), 643 (m),

620 (w), 592 (m), 557 (m), 540 (m), 469 (m), 411 (vs) cm^{-1}; UV/VIS (acetonitrile), λ = 340 (1.78), 328 (1.95), 258 (2.72) nm.

Additional information on the chemical synthesis is available via Chemotion repository: https://doi.org/10.14272/reaction/SA-FUHFF-UHFFFADPSC-CGUDGUUWCJ-UHFFFADPSC-NUHFF-NUHFF-NUHFF-ZZZ

Additional information on the analysis of the target compound is available via Chemotion repository:
https://doi.org/10.14272/CGUDGUUWCJKDIE-UHFFFAOYSA-N.1

2-(4-Butyl-1*H*-1,2,3-triazol-1-yl)quinoxaline (14k)

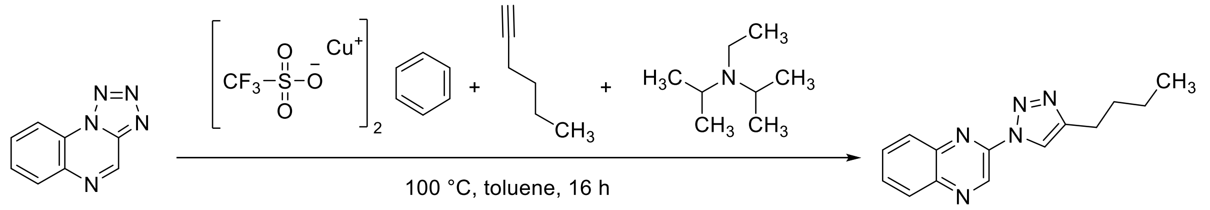

Name {P1|**14k**}: 2-(4-butyl-1*H*-1,2,3-triazol-1-yl)quinoxaline; Formula: $C_{14}H_{15}N_5$; Molecular Mass: 253.3024; Exact Mass: 253.1327; Smiles: CCCCc1nnn(c1)c1cnc2c(n1)cccc2; InChIKey: AHSWENVYXHLEHF-UHFFFAOYSA-N

The starting material tetrazolo[1,5-*a*]quinoxaline (50.0 mg, 292 µmol, 1.00 equiv) and the catalyst benzene;copper(1+);trifluoromethanesulfonate (14.7 mg, 29.2 µmol, 0.100 equiv) were dissolved in 1 mL of dry toluene under nitrogen, followed by hex-1-yne (48.0 mg, 67.1 µL, 584 µmol, 2.00 equiv) and *N*-ethyl-*N*-propan-2-ylpropan-2-amine (91.2 mg, 120 µL, 706 µmol, 2.42 equiv). The reaction mixture was stirred at 100 °C for 16 h. Then water and EtOAc were added, the organic phase was separated and the aqueous phase was extracted 3× with DCM . The combined organic phases were dried over Na_2SO_4, filtered and the solvent was removed under reduced pressure. The crude product was purified via column chromatography (cHex -> cHex/EtOAc 4:1) and 2-(4-butyl-1*H*-1,2,3-triazol-1-yl)quinoxaline (55.0 mg, 217 µmol, 74% yield) was obtained as a light brown solid.

R_f = 0.52 (cyclohexane/ethyl acetate 4:1). ^{1}H NMR (400 MHz, $CDCl_3$, ppm) δ = 9.82 (s, 1H, C*H*), 8.46 (s, 1H, C*H*), 8.18 (dd, ^3J = 8.1 Hz, ^4J = 1.8 Hz, 1H, CH_{ar}CN), 8.04 (dd, ^3J = 8.3 Hz, ^4J = 1.7 Hz, 1H, CH_{ar}CN), 7.85–7.77 (m, 2H, CH_{ar}), 2.86 (t, ^3J = 7.6 Hz, 2H, NCCH_2), 1.82–1.75 (m, 2H, NCCH_2CH_2), 1.46 (sext, ^3J = 7.4 Hz, 2H, C$H_2$$CH_3$), 0.98 (t, ^3J = 7.4 Hz, 3H, CH_3); ^{13}C NMR (100 MHz, $CDCl_3$ [77.0 ppm], ppm) δ = 149.5 (1C, C_q), 143.1 (1C, C_q), 142.0 (1C, C_q), 140.0 (1C, C_q), 137.8 (1C, *C*H), 131.4 (1C, CH_{ar}), 130.0 (1C, CH_{ar}), 129.5 (1C, CH_{ar}), 128.7 (1C, CH_{ar}), 118.2 (1C, *C*H), 31.3 (1C, NC$CH_2$$CH_2$), 25.3 (1C, NC$CH_2$), 22.3 (1C, $CH_2$$CH_3$), 13.8 (1C, CH_3); MS (EI, m/z, 70 eV, 60 °C): 253 [M]$^+$ (2), 224 (13), 210 (13), 197 (34), 196 (37), 182 (38), 157 (10), 130 (27), 129 (100), 103 (11), 102 (39). HRMS (EI, $C_{14}H_{15}N_5$): Calcd 253.1322, Found 253.1323; IR (ATR, ṽ) = 3146 (w), 3080 (w), 2951 (m), 2924 (m), 2859 (m), 1568 (m), 1555 (w), 1499 (s), 1479 (m), 1469 (m), 1451 (s), 1357 (m), 1334 (w), 1322 (m), 1262 (w), 1237 (s), 1210 (m), 1198 (m), 1174 (m), 1136 (m), 1129 (m), 1105 (w), 1079 (w), 1030 (s), 1013 (w), 987 (s), 980 (s), 952 (vs), 922 (s), 918 (s), 827 (m), 759 (vs), 728 (m), 674 (s), 646 (m), 619 (w), 613 (w), 588 (w), 541 (w), 409 (vs), 388 (m) cm^{-1}; λ= 340 (0.93), 328 (1.03), 252 (2.13) nm.

Additional information on the chemical synthesis is available via Chemotion repository: https://doi.org/10.14272/reaction/SA-FUHFF-UHFFFADPSC-AHSWENVYXH-UHFFFADPSC-NUHFF-NUHFF-NUHFF-ZZZ

Additional information on the analysis of the target compound is available via Chemotion repository:
https://doi.org/10.14272/AHSWENVYXHLEHF-UHFFFAOYSA-N.1

(1-(Quinoxalin-2-yl)-1*H*-1,2,3-triazol-4-yl)methyl acetate (14l)

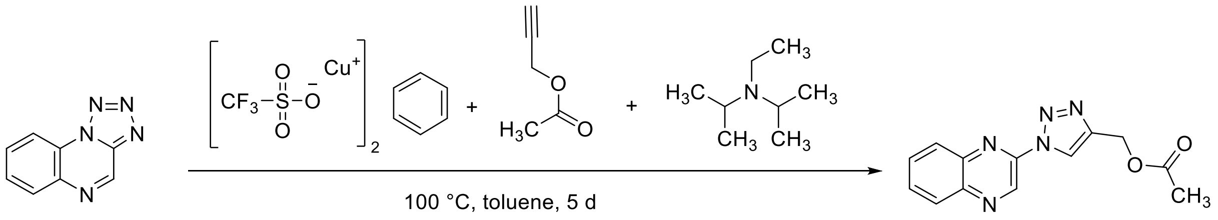

Name {P1|**14l**}: (1-(quinoxalin-2-yl)-1*H*-1,2,3-triazol-4-yl)methyl acetate; Formula: $C_{13}H_{11}N_5O_2$; Molecular Mass: 269.2587; Exact Mass: 269.0913; Smiles: CC(=O)OCc1nnn(c1)c1cnc2c(n1)cccc2; InChIKey: DGJCBVVQEVQILV-UHFFFAOYSA-N

The starting material tetrazolo[1,5-*a*]quinoxaline (49.8 mg, 291 µmol, 1.00 equiv), the catalyst benzene;copper(1+);trifluoromethanesulfonate (14.4 mg, 28.6 µmol, 0.0983 equiv) and prop-2-ynyl acetate (59.3 mg, 60.0 µL, 605 µmol, 2.08 equiv) were dissolved in 1 mL of dry toluene under argon, followed by *N*-ethyl-*N*-propan-2-ylpropan-2-amine (76.0 mg, 100 µL, 588 µmol, 2.03 equiv). The green reaction mixture was stirred at 100 °C for 5 days. Then water and DCM were added and the aqueous phase was extracted 3 times with DCM. The combined organic phases were dried over Na_2SO_4, filtered and the solvents were removed under reduced pressure. The crude product was purified via column chromatography on silica gel (cHex/EtOAc 20:1 to EtOAc). The brown product was applied using dry loading on celite. The product (1-(quinoxalin-2-yl)-1*H*-1,2,3-triazol-4-yl)methyl acetate (69.3 mg, 257 µmol) was obtained as a white solid in 89% yield.

R_f = 0.30 (cyclohexane/ethyl acetate 2:1). ^{1}H NMR (400 MHz, Chloroform-d [7.27 ppm], ppm) δ = 9.83 (s, 1H, NCH_{Ar}), 8.80 (s, 1H, $NCH_{triazole}$), 8.23–8.21 (m, 1H, CH_{Ar}), 8.09–8.07 (m, 1H, CH_{Ar}), 7.89–7.82 (m, 2H, CH_{Ar}), 5.37 (s, 2H, CH_2), 2.14 (s, 3H, CH_3); ^{13}C NMR (100 MHz, Chloroform-d [77.0 ppm], ppm) δ = 170.8 (1C, $COOCH_3$), 143.8 (1C, C^{IV}), 142.7 (1C, C^{IV}), 142.3 (1C, C^{IV}), 139.9 (1C, C^{IV}), 137.6 (1C, NCH_{Ar}), 131.6 (1C, CH_{Ar}), 130.4 (1C, CH_{Ar}), 129.6 (1C, CH_{Ar}), 128.8 (1C, CH_{Ar}), 121.4 (1C, $NCH_{triazole}$), 57.4 (1C, CH_2), 20.9 (1C, CH_3); HRMS (EI, $C_{13}H_{11}O_2N_5$): calcd 269.0907, found 269.0910. MS (EI, m/z, 70 eV, 90 °C): 269 $[M]^+$ (9), 199 (59), 198 (52), 182 (21), 170 (31), 144 (12), 130 (49), 129 (100), 103 (13), 102 (48); IR (ATR, ṽ) = 3165 (w), 1747 (s), 1735 (vs), 1562 (w), 1500 (s), 1475 (w), 1451 (m), 1390 (w), 1368 (m), 1349 (w), 1220 (vs), 1181 (s), 1137 (m), 1052 (w), 1037 (m), 1023 (s), 1000 (s), 986 (vs), 969 (s), 950 (vs), 921 (s), 880 (w), 858 (w), 823 (s), 796 (w), 764 (vs), 697 (w), 676 (m), 645 (w), 611 (m), 588 (m), 540 (w), 483 (w), 415 (vs), 385 (w) cm^{-1}.

Additional information on the chemical synthesis is available via Chemotion repository: https://doi.org/10.14272/reaction/SA-FUHFF-UHFFFADPSC-DGJCBVVQEV-UHFFFADPSC-NUHFF-NUHFF-NUHFF-ZZZ

Additional information on the analysis of the target compound is available via Chemotion repository:
https://doi.org/10.14272/DGJCBVVQEVQILV-UHFFFAOYSA-N.1

(1-(Quinoxalin-2-yl)-1*H*-1,2,3-triazol-4-yl)methyl acrylate (**14m**)

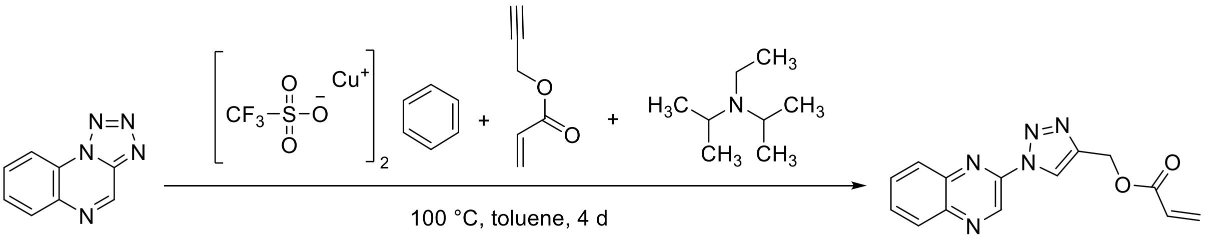

Name {P1|**14m**}: (1-(quinoxalin-2-yl)-1*H*-1,2,3-triazol-4-yl)methyl acrylate; Formula: $C_{14}H_{11}N_5O_2$; Molecular Mass: 281.2694; Exact Mass: 281.0913; Smiles: C=CC(=O)OCc1nnn(c1)c1cnc2c(n1)cccc2; InChIKey: XJHPHMLSCVFADT-UHFFFAOYSA-N

The starting material tetrazolo[1,5-*a*]quinoxaline (100 mg, 585 µmol, 1.00 equiv) and the catalyst benzene;copper(1+);trifluoromethanesulfonate (28.6 mg, 56.8 µmol, 0.0972 equiv) were dissolved in 2 mL of dry toluene under argon, followed by acrylic acid propargyl ester (133 mg, 133 µL, 1.21 mmol, 2.07 equiv) and *N*-ethyl-*N*-propan-2-ylpropan-2-amine (76.0 mg, 100 µL, 588 µmol, 2.03 equiv). The brown reaction mixture was stirred at 100 °C for 4 days. Then water was added and the brown aqueous phase was extracted 4 times with EtOAc. The combined organic phases were dried over Na_2SO_4, filtered and the solvents were removed under reduced pressure. The obtained crude product was purified via flash-chromatography (dryload on celite, Interchim devices puriFLASH 5.125) on silica gel (PF-15SIHP-F0012) using cHex to EtOAc in 16 column volumes. The expected product (1-(quinoxalin-2-yl)-1*H*-1,2,3-triazol-4-yl)methyl acrylate (79.8 mg, 284 µmol) was obtained as a yellow solid in 49% yield.

R_f = 0.37 (cyclohexane/ethyl acetate 2:1). ^{1}H NMR (400 MHz, Chloroform-d [7.27 ppm], ppm) δ = 9.83 (s, 1H, C*H*), 8.83 (s, 1H, C*H*), 8.23–8.20 (m, 1H, CH_{Ar}), 8.09–8.07 (m, 1H, CH_{Ar}), 7.89–7.82 (m, 2H, CH_{Ar}), 6.50 (dd, *J* = 1.3 Hz, *J* = 17.4 Hz, 1H, CH_{alkene}), 6.23–6.16 (m, 1H, C*H*COO), 5.90 (dd, *J* = 1.3 Hz, *J* = 10.5 Hz, 1H, CH_{alkene}), 5.47 (s, 2H, CH_2); ^{13}C NMR (100 MHz, Chloroform-d [77.0 ppm], ppm) δ = 165.9 (1C, C_q), 143.7 (1C, C_q), 142.7 (1C, C_q), 142.3 (1C, C_q), 139.9 (1C, C_q), 137.6 (1C, C*H*), 131.8 (1C, CH_2), 131.6 (1C, CH_{Ar}), 130.4 (1C, CH_{Ar}), 129.6 (1C, CH_{Ar}), 128.8 (1C, CH_{Ar}), 127.8 (1C, *C*H), 121.6 (1C, *C*H), 57.4 (1C, CH_2); MS (EI, m/z, 70 eV, 100 °C): 281 (10), 199 (34), 198 (72), 182 (20), 170 (28), 130 (36), 129 (100), 103 (12), 102 (46), 55 (39). HRMS ($C_{14}H_{11}O_2N_5$): Calcd 281.0907, Found 281.0907; IR (ATR, ṽ) = 3162 (w), 1720 (vs), 1619 (w), 1565 (w), 1500 (s), 1476 (m), 1451 (m), 1408 (s), 1368 (w), 1351 (w), 1283 (w), 1254 (vs), 1169 (vs), 1139 (m), 1054 (s), 1031 (vs), 963 (vs),

950 (vs), 921 (s), 827 (s), 807 (vs), 761 (vs), 696 (m), 674 (m), 659 (w), 649 (m), 616 (w), 603 (m), 589 (m), 534 (w), 414 (s), 378 (m) cm^{-1}.

Additional information on the chemical synthesis is available via Chemotion repository: https://doi.org/10.14272/reaction/SA-FUHFF-UHFFFADPSC-XJHPHMLSCV-UHFFFADPSC-NUHFF-NUHFF-NUHFF-ZZZ

Additional information on the analysis of the target compound is available via Chemotion repository:
https://doi.org/10.14272/XJHPHMLSCVFADT-UHFFFAOYSA-N.1

***N*-(prop-2-yn-1-yl)-*N*-((1-(quinoxalin-2-yl)-1*H*-1,2,3-triazol-4-yl)methyl)prop-2-yn-1-amine (14n)**

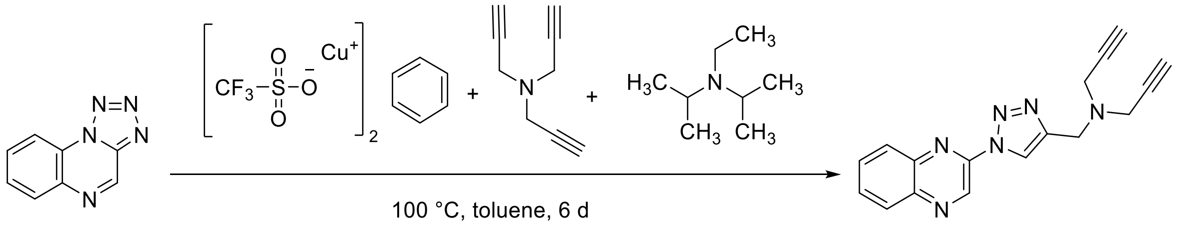

Name {P1|**14n**}: *N*-(prop-2-yn-1-yl)-*N*-((1-(quinoxalin-2-yl)-1*H*-1,2,3-triazol-4-yl)methyl)prop-2-yn-1-amine; Formula: $C_{17}H_{14}N_6$; Molecular Mass: 302.3333; Exact Mass: 302.1280; Smiles: C#CCN(Cc1nnn(c1)c1cnc2c(n1)cccc2)CC#C; InChIKey: FDGHOWIPSINAHA-UHFFFAOYSA-N

The starting material tetrazolo[1,5-*a*]quinoxaline (97.6 mg, 564 µmol, 1.00 equiv) and the catalyst benzene;copper(1+);trifluoromethanesulfonate (28.5 mg, 56.6 µmol, 0.100 equiv) were dissolved in 2 mL of dry toluene, under argon, followed by *N,N*-bis(prop-2-ynyl)prop-2-yn-1-amine (148 mg, 160 µL, 1.13 mmol, 2.01 equiv) and *N*-ethyl-*N*-propan-2-ylpropan-2-amine (153 mg, 201 µL, 1.18 mmol, 2.10 equiv). The brown reaction mixture was stirred at 100 °C for 6 days. Formation of the desired product was confirmed via LC-MS. Then water and EtOAc were added and the aqueous phase was extracted 3 times with EtOAc. The combined organic phases were dried over Na_2SO_4, filtered and the solvents were removed under reduced pressure. The crude brown product was purified via column chromatography *c*Hex -> EtOAc (dryload on Celite). The expected product *N*-(prop-2-yn-1-yl)-*N*-((1-(quinoxalin-2-yl)-1*H*-1,2,3-triazol-4-yl)methyl)prop-2-yn-1-amine (35.7 mg, 118 µmol) was obtained as a brown solid in 21% yield.

R_f = 0.1 (cyclohexane/ethyl acetate 2:1). ^{1}H NMR (400 MHz, Chloroform-d [7.27 ppm], ppm) δ = 9.84 (d, *J* = 1.6 Hz, 1H, C*H*), 8.74 (d, *J* = 0.7 Hz, 1H, C*H*), 8.22 (d, *J* = 7.6 Hz, 1H, C*H*Ar), 8.07 (d, *J* = 7.6 Hz, 1H, C*H*Ar), 7.88–7.81 (m, 2H, C*H*Ar), 4.04 (d, *J* = 0.9 Hz, 2H, C*H2*), 3.57 (s, 4H, NC*H2*), 2.32 (d, *J* = 1.8 Hz, 2H, CH_{alkyne}); ^{13}C NMR (100 MHz, Chloroform-d [77.0 ppm], ppm) δ = 145.6 (1C, C_q), 142.9 (1C, C_q), 142.2 (1C, C_q), 140.0 (1C, C_q), 137.7 (1C, *C*H), 131.5 (1C, CH_{Ar}), 130.2 (1C, CH_{Ar}), 129.5 (1C, CH_{Ar}), 128.7 (1C, CH_{Ar}), 120.6 (1C, *C*H), 78.3 (2C, C_q), 73.6 (2C, CH_{alkyne}), 47.9 (1C, CH_2), 42.4 (2C, CH_2); HRMS ($C_{17}H_{14}N_6$): calcd 302.1274, found 302.1275 MS (EI, m/z, 70 eV, 130 °C): 302 $[M]^+$ (8), 274 (32), 273 (100), 264 (18), 263 (100), 211 (11), 182 (12), 129 (32), 106 (10), 102 (18); IR (ATR, ṽ) = 3293 (w), 3244 (w), 3163 (w), 2819 (w), 1561 (w), 1500 (s), 1476 (w), 1449 (s), 1436 (m), 1400 (w), 1364 (w), 1350 (w),

1327 (m), 1298 (m), 1251 (w), 1215 (s), 1183 (m), 1140 (w), 1118 (s), 1035 (vs), 1003 (s), 993 (s), 952 (vs), 904 (m), 839 (m), 820 (m), 766 (vs), 637 (vs), 626 (vs), 585 (vs), 418 (vs), 377 (vs) cm^{-1}.

Additional information on the chemical synthesis is available via Chemotion repository: https://doi.org/10.14272/reaction/SA-FUHFF-UHFFFADPSC-FDGHOWIPSI-UHFFFADPSC-NUHFF-NUHFF-NUHFF-ZZZ

Additional information on the analysis of the target compound is available via Chemotion repository:
https://doi.org/10.14272/FDGHOWIPSINAHA-UHFFFAOYSA-N.1

(1-(Quinoxalin-2-yl)-1*H*-1,2,3-triazol-4-yl)methanol (14o), quinoxalin-2-ylamine

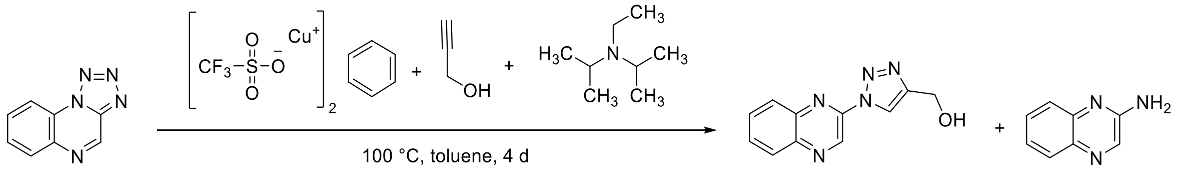

Name {P1|**14o**}: (1-(quinoxalin-2-yl)-1*H*-1,2,3-triazol-4-yl)methanol; Formula: $C_{11}H_9N_5O$; Molecular Mass: 227.2221; Exact Mass: 227.0807; Smiles: OCc1nnn(c1)c1cnc2c(n1)cccc2; InChIKey: YEBFONFGCJEMAG-UHFFFAOYSA-N

Name {P2}: quinoxalin-2-ylamine; Formula: $C_8H_7N_3$; Molecular Mass: 145.1613; Exact Mass: 145.0640; Smiles: Nc1cnc2c(n1)cccc2; InChIKey: YOWAEZWWQFSEJD-UHFFFAOYSA-N

The starting material tetrazolo[1,5-*a*]quinoxaline (150 mg, 876 µmol, 1.00 equiv) and the catalyst benzene;copper(1+);trifluoromethanesulfonate (44.0 mg, 87.4 µmol, 0.0998 equiv) were dissolved in 3 mL of dry toluene under argon, followed by prop-2-yn-1-ol (94.8 mg, 100 µL, 1.69 mmol, 1.92 equiv) and *N*-ethyl-*N*-propan-2-ylpropan-2-amine (342 mg, 450 µL, 2.65 mmol, 3.00 equiv). The green reaction mixture was stirred at 100 °C for 4 days. Then water was added and the brown aqueous phase was extracted 4 times with DCM. The combined organic phases were dried over Na_2SO_4, filtered and the solvents were removed under reduced pressure. The obtained crude product was purified via flash-chromatography (dryload on Celite, Interchim devices puriFLASH XS420) on silica gel (PF-15SIHP-F0025) using *c*Hex to EtOAc in 12 column volumes. The expected product (1-(quinoxalin-2-yl)-1*H*-1,2,3-triazol-4-yl)methanol (47.7 mg, 210 µmol) was obtained as a brown solid in 24% yield; quinoxalin-2-ylamine (39.8 mg, 274 µmol, 31% yield) was obtained as a slightly impure side product and 25 mg of starting material were reisolated.

R_f = 0.38 (cyclohexane/ethyl acetate 2:1). ^{1}H NMR (400 MHz, DMSO-d_6 [2.50 ppm], ppm) δ = 9.73 (s, 1H, CH), 8.88 (s, 1H, CH), 8.25–8.22 (m, 1H, CH_{Ar}), 8.16–8.13 (m, 1H, CH_{Ar}), 8.01–7.93 (m, 2H, CH_{Ar}), 5.44 (t, *J* = 5.7 Hz, 1H, OH), 4.70 (d, *J* = 5.6 Hz, 2H, CH_2); ^{13}C NMR (100 MHz, DMSO-d6 [39.5 ppm], ppm) δ = 150.1 (C, C_q), 143.4 (1C, C_q), 142.0 (1C, C_q), 139.8 (1C, C_q), 138.6 (1C, CH), 132.3 (1C, CH_{Ar}), 131.0 (1C, CH_{Ar}), 129.6 (1C, CH_{Ar}), 129.1 (1C, CH_{Ar}), 120.6 (1C, CH), 55.3 (1C, CH_2); MS (EI, 70

eV, 100 °C), m/z (%): 227 (9) $[M]^+$, 202 (63), 199 (23), 198 (55), 185 (67), 172 (16), 171 (45), 170 (31), 159 (18), 147 (56), 146 (27), 145 (43), 143 (20), 130 (32), 129 (100), 118 (48), 103 (20), 102 (76), 90 (17), 76 (18), 75 (15). HRMS ($C_{11}H_9O_1N_5$): calcd 227.0802, found 227.0802; IR (ATR, ṽ) = 3293 (s), 3116 (m), 3084 (m), 2970 (w), 2935 (w), 2861 (w), 1649 (w), 1602 (w), 1572 (m), 1499 (vs), 1477 (s), 1451 (vs), 1392 (m), 1375 (m), 1350 (s), 1336 (m), 1264 (w), 1244 (s), 1224 (s), 1200 (vs), 1180 (s), 1143 (s), 1130 (s), 1055 (vs), 1017 (vs), 997 (vs), 972 (s), 953 (vs), 908 (s), 867 (vs), 795 (w), 765 (vs), 696 (s), 643 (vs), 618 (vs), 589 (vs), 537 (vs), 487 (s), 446 (s), 416 (vs), 378 (vs) cm^{-1}.

^{1}H NMR (400 MHz, Chloroform-d [7.27 ppm], ppm) δ = 8.35–8.33 (m, 1H), 7.92 (d, *J* = 8.1 Hz, 1H), 7.67–7.59 (m, 2H), 7.46–7.42 (m, 1H), 5.07 (s, 2H).

Additional information on the chemical synthesis is available via Chemotion repository: https://doi.org/10.14272/reaction/SA-FUHFF-UHFFFADPSC-RICHEZCOLF-UHFFFADPSC-NUHFF-NUHFF-NUHFF-ZZZ

Additional information on the analysis of the target compound is available via Chemotion repository:
https://doi.org/10.14272/YEBFONFGCJEMAG-UHFFFAOYSA-N.1
https://doi.org/10.14272/YOWAEZWWQFSEJD-UHFFFAOYSA-N.1

The synthesis of the side product quinoxalin-2-amine has been previously reported in literature [24].

4-Ethynylbenzoic acid (**4p**)

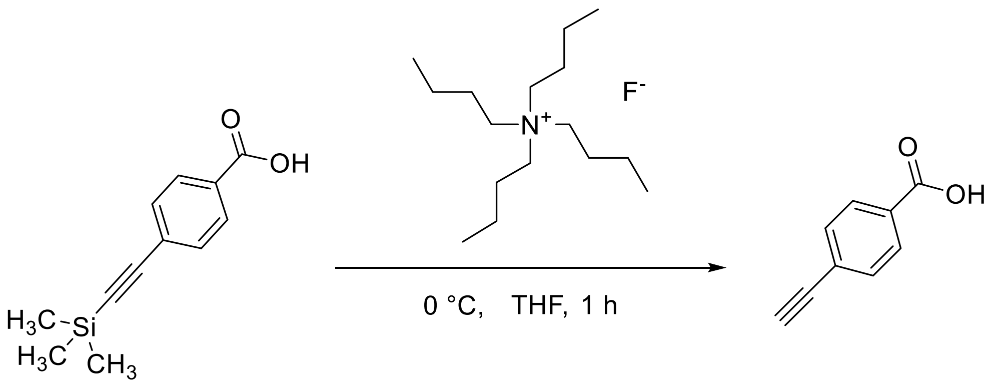

Name {P1|**4p**}: 4-ethynylbenzoic acid; Formula: $C_9H_6O_2$; Molecular Mass: 146.1427; Exact Mass: 146.0368; Smiles: C#Cc1ccc(cc1)C(=O)O; InChIKey: SJXHLZCPDZPBPW-UHFFFAOYSA-N

The starting material 4-(2-trimethylsilylethynyl)benzoic acid (420 mg, 1.92 mmol, 1.00 equiv) was dissolved in dry THF (24 mL) and tetrabutylazanium;fluoride (575 mg, 2.20 mL, 2.20 mmol, 1.00M, 1.14 equiv) was added at 0 °C under argon. The reaction was stirred for 1 hour and quenched via addition of distilled water. The aqueous phase was extracted 3x with ethyl acetate; the combined organic phases were dried over Na_2SO_4, filtered and the solvent was removed under reduced pressure. The obtained crude product was purified via flash-chromatography on silica gel using DCM to DCM/MeOH

10:1 and 4-ethynylbenzoic acid 4-ethynylbenzoic acid (175 mg, 1.20 mmol) was obtained as a white solid in 62% yield. Note: This reaction was repeated with a yield of 92%.

R_f = 0.19 (CH_2Cl_2/MeOH 10:1). ^{1}H NMR (400 MHz, DMSO-d_6, ppm) δ = 7.94–7.92 (m, 2H, CH_{ar}), 7.60–7.58 (m, 2H, CH_{ar}), 4.42 (s, 1H, CC*H*). Missing 1H (1H, O*H*) due to overlapping with water peak (broad signal at 3.41 ppm). ^{13}C NMR (100 MHz, DMSO-d_6, ppm) δ = 166.7 (1C, *C*OOH), 131.2 (2C, *C*H_{ar}), 131.0 (1C, C_qCOOH), 129.5 (2C, *C*H_{ar}), 126.0 (1C, C_q), 83.5 (1C, C*C*H), 82.8 (1C, *C*CH); MS (EI, m/z, 70 eV, 40 °C): 146 (100) $[M]^+$, 129 (56), 101 (33), 75 (16). HRMS ($C_9H_6O_2$): calcd 146.0362, found 146.0361; IR (ATR, ṽ) = 3265 (s), 2809 (m), 2660 (m), 2550 (m), 1673 (vs), 1605 (s), 1560 (s), 1425 (s), 1404 (s), 1319 (vs), 1298 (vs), 1282 (vs), 1177 (vs), 1126 (s), 1113 (s), 1065 (m), 1017 (m), 1010 (m), 979 (m), 922 (vs), 858 (vs), 827 (s), 768 (vs), 745 (s), 694 (s), 670 (vs), 635 (vs), 571 (s), 551 (vs), 524 (vs), 507 (vs), 378 (s) cm^{-1}.

Additional information on the chemical synthesis is available via Chemotion repository: https://doi.org/10.14272/reaction/SA-FUHFF-UHFFFADPSC-SJXHLZCPDZ-UHFFFADPSC-NUHFF-NUHFF-NUHFF-ZZZ

Additional information on the analysis of the target compound is available via Chemotion repository:
https://doi.org/10.14272/SJXHLZCPDZPBPW-UHFFFAOYSA-N.1

The synthesis of this compound has been previously described and the ^{1}H NMR data corresponds with the literature [26].

2-(4-Butyl-1*H*-1,2,3-triazol-1-yl)-3-methylquinoxaline (15a), 3-methylquinoxalin-2-amine (17a)

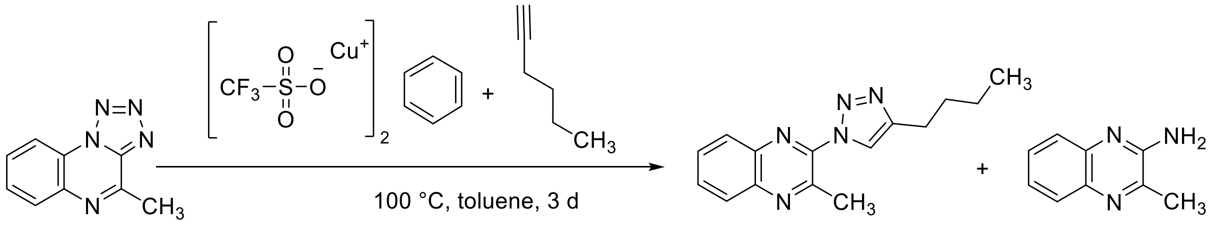

Name {P1|**15a**}: 2-(4-butyl-1*H*-1,2,3-triazol-1-yl)-3-methylquinoxaline; Formula: $C_{15}H_{17}N_5$; Molecular Mass: 267.3290; Exact Mass: 267.1484; Smiles: CCCCc1nnn(c1)c1nc2ccccc2nc1C; InChIKey: MGHMBCSNZLEULM-UHFFFAOYSA-N

Name {P2|**17a**}: 3-methylquinoxalin-2-amine; Formula: $C_9H_9N_3$; Molecular Mass: 159.1879; Exact Mass: 159.0796; Smiles: Cc1nc2ccccc2nc1N; InChIKey: WGHZDFAULZNZJE-UHFFFAOYSA-N

The starting material 4-methyltetrazolo[1,5-a]quinoxaline (51.0 mg, 275 µmol, 1.00 equiv) and the catalyst benzene;copper(1+);trifluoromethanesulfonate (13.6 mg, 27.0 µmol, 0.0980 equiv) were dissolved in 1 mL of dry toluene in a crimp vial under argon, followed by hex-1-yne (111 mg, 155 µL, 1.35 mmol, 4.90 equiv). The reaction mixture was stirred at 100 °C for 3 days. Then water and ethyl acetate were added to the black

solution, the organic phase was separated and the aqueous phase was extracted 3x with ethyl acetate. The combined organic phases were dried over Na_2SO_4, filtered and the solvent was removed under reduced pressure. The crude mixture was purified via column chromatography (dryload on Celite, *c*Hex -> EtOAc). 2-(4-Butyl-1*H*-1,2,3-triazol-1-yl)-3-methylquinoxaline (23.0 mg, 86.0 µmol, 31% yield) was eluted with *c*Hex/ethyl acetate (3:1), 3-methylquinoxalin-2-amine (8.00 mg, 50.3 µmol, 18% yield) was eluted with pure ethyl acetate. Both compounds were obtained as brown solids.

R_f = 0.5 (cyclohexane/ethyl acetate 2:1). ^{1}H NMR (400 MHz, $CDCl_3$, ppm) δ = 8.28 (s, 1H, C*H*$_{triazole}$), 8.11–8.09 (m, 1H, C*H*$_{ar}$), 8.04–8.01 (m, 1H, C*H*$_{ar}$), 7.82–7.75 (m, 2H, C*H*$_{ar}$), 3.10 (s, 3H, C*H*$_3$), 2.87 (t, ^3J = 8.1 Hz, 2H, C_qC*H*$_2$), 1.79 (p, ^3J = 7.4 Hz, 2H, CH_2C*H*$_2$), 1.48 (h, ^3J = 7.5 Hz, 2H, C*H*$_2$$CH_3$), 0.99 (t, ^3J = 7.3 Hz, 3H, CH_2C*H*$_3$) Further analysis available at https://dx.doi.org/10.14272/reaction/SA-FUHFF-UHFFFADPSC-MGHMBCSNZL-UHFFFADPSC-NUHFF-NUHFF-NUHFF-ZZZ; EA ($C_{15}H_{17}N_5$): Calcd C 67.39; H 6.41; N 26.20. Found C 67.19; H 6.41; N 24.81; UV/VIS (acetonitrile), λ = 326 (1.54), 248 (2.60) nm.

^{1}H NMR (400 MHz, $CDCl_3$, ppm) δ = 7.86 (d, ^3J = 8.2 Hz, 1H, C*H*$_{ar}$), 7.65 (d, ^3J = 8.3 Hz, 1H, C*H*$_{ar}$), 7.55 (t, ^3J = 7.6 Hz, 1H, C*H*$_{ar}$), 7.42 (t, ^3J = 7.6 Hz, 1H, C*H*$_{ar}$), 5.10 (bs, 2H, N*H*$_2$), 2.60 (s, 3H, C*H*$_3$); ^{13}C NMR (100 MHz, $CDCl_3$ [77.0 ppm], ppm) δ = 151.2 (1C, *C*$_q$), 144.4 (1C, *C*$_q$), 141.0 (1C, *C*$_q$), 138.8 (1C, *C*$_q$), 129.1 (1C, *C*H$_{ar}$), 128.1 (1C, *C*H$_{ar}$), 125.6 (1C, *C*H$_{ar}$), 125.0 (1C, *C*H$_{ar}$), 21.3 (1C, *C*H$_3$); MS (EI, m/z, 70 eV, 50 °C): 159 $[M]^+$ (100), 132 (21), 118 (8), 117 (9), 91 (7), 90 (11), 76 (9). HRMS ($C_9H_9N_3$): calcd 159.0791, found 159.0792; IR (ATR, ṽ) = 3479 (w), 3302 (w), 3109 (w), 3060 (w), 2956 (w), 2925 (w), 2856 (w), 1642 (s), 1605 (w), 1577 (w), 1571 (w), 1496 (w), 1472 (w), 1434 (vs), 1383 (m), 1371 (m), 1351 (m), 1312 (w), 1275 (w), 1254 (m), 1238 (m), 1188 (m), 1145 (w), 1136 (w), 1116 (w), 1030 (w), 1007 (m), 946 (w), 914 (w), 850 (w), 755 (vs), 720 (s), 694 (m), 674 (s), 637 (m), 611 (s), 552 (m), 483 (w), 465 (w) cm^{-1}.

Additional information on the chemical synthesis is available via Chemotion repository: https://doi.org/10.14272/reaction/SA-FUHFF-UHFFFADPSC-UAPOWWNEVS-UHFFFADPSC-NUHFF-NUHFF-NUHFF-ZZZ

Additional information on the analysis of the target compound is available via Chemotion repository:
https://doi.org/10.14272/MGHMBCSNZLEULM-UHFFFAOYSA-N.2
https://doi.org/10.14272/WGHZDFAULZNZJE-UHFFFAOYSA-N.2

The use of the side product 3-methylquinoxalin-2-amine has been previously reported in literature [27].

2-(4-Butyl-1*H*-1,2,3-triazol-1-yl)-3-methylquinoxaline (15a)

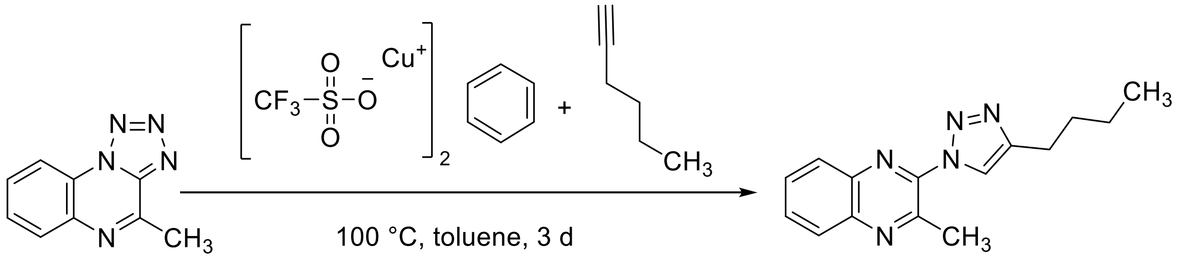

Name {P1|**15a**}: 2-(4-butyl-1*H*-1,2,3-triazol-1-yl)-3-methylquinoxaline; Formula: $C_{15}H_{17}N_5$; Molecular Mass: 267.3290; Exact Mass: 267.1484; Smiles: CCCCc1nnn(c1)c1nc2ccccc2nc1C; InChIKey: MGHMBCSNZLEULM-UHFFFAOYSA-N

The starting material 4-methyl-[1,2,3,4]tetrazolo[1,5-*a*]quinoxaline (101 mg, 544 µmol, 1.00 equiv) and the catalyst benzene;copper(1+);trifluoromethanesulfonate (29.1 mg, 57.8 µmol, 0.106 equiv) were dissolved in 2 mL of dry toluene, under argon, followed by 1-hexyne (85.9 mg, 120 µL, 1.05 mmol, 1.92 equiv). The brown reaction mixture was stirred at 100 °C for 3 days. Then water was added and the aqueous phase was extracted 4 times with DCM. The combined organic phases were dried over Na_2SO_4, filtered and the solvents were removed under reduced pressure. The obtained crude product was purified via flash-chromatography (dryload on celite, Interchim devices puriFLASH XS420) on silica gel (PF-15SIHP-F0025) using cHex to cHex/EtOAc 2:1 in 12 column volumes. The expected product 2-(4-butyl-1*H*-1,2,3-triazol-1-yl)-3-methylquinoxaline (24.1 mg, 90.2 µmol) was obtained as a brown solid in 17% yield and 20 mg of starting material were reisolated.

R_f = 0.48 (cyclohexane/ethyl acetate 2:1). ^{1}H NMR (400 MHz, Chloroform-d [7.27 ppm], ppm) δ = 8.29 (s, 1H, C*H*), 8.12–8.10 (m, 1H, C*H*$_{Ar}$), 8.04–8.02 (m, 1H, C*H*$_{Ar}$), 7.83–7.77 (m, 2H, C*H*$_{Ar}$), 3.10 (s, 3H, C*H*$_3$), 2.87 (t, *J* = 7.7 Hz, 2H, C*H*$_2$), 1.79 (quint, *J* = 7.6 Hz, 2H, C*H*$_2$), 1.48 (quint, *J* = 7.3 Hz, 2H, C*H*$_2$), 0.99 (t, *J* = 7.3 Hz, 3H, C*H*$_3$); ^{13}C NMR (100 MHz, Chloroform-d [77.0 ppm], ppm) δ = 148.7 (1C, *C*$_q$), 148.3 (1C, *C*$_q$), 143.1 (1C, *C*$_q$), 141.5 (1C, *C*$_q$), 139.0 (1C, *C*$_q$), 130.5 (1C, *C*H$_{Ar}$), 130.3 (1C, *C*H$_{Ar}$), 128.5 (1C, *C*H$_{Ar}$), 128.5 (1C, *C*H$_{Ar}$), 121.0 (1C, *C*H), 31.3 (1C, *C*H$_2$), 25.3 (1C, *C*H$_2$), 24.6 (1C, *C*H$_3$), 22.3 (1C, *C*H$_2$), 13.8 (1C, *C*H$_3$); HRMS ($C_{15}H_{17}N_5$): calcd 267.1478, found 267.1479. MS (EI, m/z, 70 eV, 100 °C): 267 $[M]^+$ (6), 239 (46), 238 (100), 224 (18), 211 (16), 210 (14), 196 (27), 144 (26), 143 (100), 102 (29); IR (ATR, ṽ) = 3190 (w), 3055 (vw), 3012 (vw), 2953 (m), 2931 (m), 2870 (w), 2853 (w), 1611 (vw), 1561 (w), 1492 (s), 1466 (w), 1435 (vs), 1375 (m), 1356 (w), 1312 (m), 1292 (w), 1244 (w), 1214 (vs), 1156 (s), 1139 (w), 1033 (vs), 1010 (s), 973 (vs), 895 (m), 807 (s), 783 (m), 769 (vs), 728 (m), 708 (s), 635 (m), 615 (m), 589 (m), 551 (w), 492 (w), 475 (w), 455 (s) cm^{-1}.

Additional information on the chemical synthesis is available via Chemotion repository: https://doi.org/10.14272/reaction/SA-FUHFF-UHFFFADPSC-MGHMBCSNZL-UHFFFADPSC-NUHFF-NUHFF-NUHFF-ZZZ

Additional information on the analysis of the target compound is available via Chemotion repository:
https://doi.org/10.14272/MGHMBCSNZLEULM-UHFFFAOYSA-N.1

2-(4-Butyl-1*H*-1,2,3-triazol-1-yl)-3-isopropylquinoxaline (15b), 1-butyl-4-isopropylimidazo[1,2-*a*]quinoxaline (16b), 3-propan-2-ylquinoxalin-2-amine (17b)

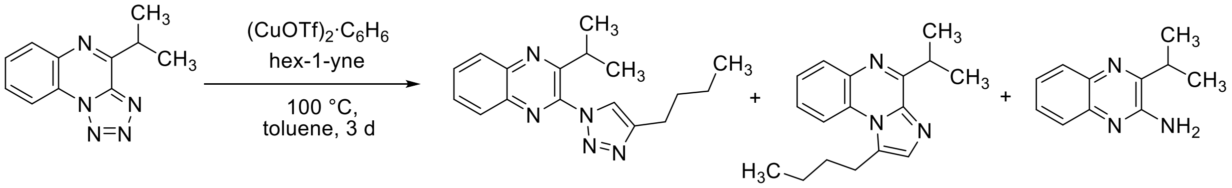

Name {P1|**15b**}: 2-(4-butyl-1*H*-1,2,3-triazol-1-yl)-3-isopropylquinoxaline; Formula: $C_{17}H_{21}N_5$; Molecular Mass: 295.3821; Exact Mass: 295.1797; Smiles: CCCCc1nnn(c1)c1nc2ccccc2nc1C(C)C; InChIKey: YKTZEDKRYOIMNU-UHFFFAOYSA-N

Name {P2|**16b**}: 1-butyl-4-isopropylimidazo[1,2-*a*]quinoxaline; Formula: $C_{17}H_{21}N_3$; Molecular Mass: 267.3687; Exact Mass: 267.1735; Smiles: CCCCc1cnc2n1c1ccccc1nc2C(C)C; InChIKey: AFQHYVRVQPQNNN-UHFFFAOYSA-N

Name {P3|**17b**}: 3-propan-2-ylquinoxalin-2-amine; Formula: $C_{11}H_{13}N_3$; Molecular Mass: 187.2410; Exact Mass: 187.1109; Smiles: CC(c1nc2ccccc2nc1N)C; InChIKey: IRSRPTQGLZTLGL-UHFFFAOYSA-N

The starting material 4-isopropyltetrazolo[1,5-*a*]quinoxaline (51.0 mg, 239 µmol, 1.00 equiv) and the catalyst benzene;copper(1+);trifluoromethanesulfonate (11.8 mg, 23.4 µmol, 0.0980 equiv) were dissolved in 1 mL of dry toluene in a crimp vial under argon, followed by addition of hex-1-yne (96.3 mg, 135 µL, 1.17 mmol, 4.90 equiv). The reaction mixture was stirred at 100 °C for 3 d; then water and EtOAc were added to the brown-black reaction mixture, the organic phase was separated and the aqueous phase was extracted 3× with EtOAc. The combined organic phases were dried over Na_2SO_4, filtered and the solvent was removed under reduced pressure. The crude product mixture was purified via column chromatography (dryload on Celite, *c*Hex -> ethyl acetate) and 2-(4-butyl-1*H*-1,2,3-triazol-1-yl)-3-isopropylquinoxaline (elution at 4:1 *c*Hex/ethyl acetate, 6.00 mg, 20.3 µmol, 8% yield), 3-propan-2-ylquinoxalin-2-amine (elution with ethyl acetate, 5.00 mg, 26.7 µmol, 11% yield) and 1-butyl-4-isopropylimidazo[1,2-*a*]quinoxaline (elution at 3:1 cHex/ethyl acetate, further purified using DCM -> DCM/ethyl acetate 3:1, 11.0 mg, 41.1 µmol, 17% yield) were obtained; 13 mg (elution at 3:1 *c*Hex/ethyl acetate, 25% yield) of starting material were reisolated.

R_f = 0.72 (triazole product) (cyclohexane/ethyl acetate 2:1). ^{1}H NMR (400 MHz, $CDCl_3$, ppm) δ = 8.18–8.15 (m, 1H, CH_{ar}), 8.14 (s, 1H, C$H_{triazole}$), 8.05–8.02 (m, 1H, CH_{ar}), 7.84–7.76 (m, 2H, CH_{ar}), 4.02 (hept, ^3J = 6.7 Hz, 1H, CH(CH_3)$_2$), 2.88 (t, ^3J = 7.6 Hz, 2H, C_qCH_2), 1.80 (p, ^3J = 7.6 Hz, 2H, CH_2CH_2), 1.49 (h, ^3J = 7.5 Hz, 2H, C$H_2$$CH_3$), 1.40 (d, ^3J = 6.7 Hz, 6H, CH_3), 1.00 (t, ^3J = 7.4 Hz, 3H, CH_2CH_3); ^{13}C NMR (10 MHz, $CDCl_3$ [77.0 ppm], ppm) δ = 157.2 (1C, C_q), 148.3 (1C, $C_{triazole}$), 142.6 (1C, C_q), 142.1 (1C, C_q), 138.9 (1C, C_q), 130.5 (1C, CH_{ar}), 130.2 (1C, CH_{ar}), 128.9 (1C, CH_{ar}), 128.6 (1C, CH_{ar}), 121.6 (1C, $CH_{triazole}$), 31.5 (1C, CH(CH_3)$_2$), 31.3 (1C, CH_2CH_2), 25.3 (1C, C_qCH_2), 22.4 (1C, CH_2CH_3), 21.9 (2C, CH_3), 13.8 (1C, CH_2CH_3). MS (FAB, 3-NBA), m/z (%): 297 $[M+1]^+$ (23), 296 $[M]^+$ (100), 268 (13), 171 (30), 129 (21). HRMS (FAB, $C_{17}H_{22}N_5$): calcd 296.1870, found 296.1871. IR (ATR, ṽ) = 3189 (w), 2956 (m), 2922

(s), 2871 (w), 2856 (m), 1557 (w), 1487 (m), 1460 (m), 1438 (s), 1426 (vs), 1375 (m), 1354 (m), 1302 (m), 1273 (w), 1242 (w), 1213 (vs), 1193 (m), 1170 (w), 1142 (m), 1132 (m), 1105 (w), 1085 (m), 1031 (vs), 1013 (s), 973 (vs), 932 (w), 898 (w), 877 (w), 805 (m), 779 (m), 766 (vs), 745 (m), 731 (m), 684 (w), 642 (m), 612 (s), 588 (m), 560 (w), 530 (m), 492 (w), 470 (w), 426 (w), 378 (m) cm^{-1}.

^{1}H NMR (400 MHz, $CDCl_3$, ppm) δ = 8.21–8.17 (m, 1H, CH_{ar}), 8.12–8.10 (m, 1H, CH_{ar}), 7.57–7.53 (m, 2H, CH_{ar}), 7.50 (s, 1H, C$H_{imidazol}$), 4.03 (p, ^{3}J = 6.9 Hz, 1H, CH(CH_3)$_2$), 3.29 (t, ^{3}J = 7.8 Hz, 2H, CqCH_2), 1.91 (p, ^{3}J = 7.6 Hz, 2H, CH_2CH_2), 1.58 (h, ^{3}J = 7.4 Hz,, 2H, CH_2CH_3), 1.50 (d, ^{3}J = 6.8 Hz, 6H, CH(CH_3)$_2$), 1.05 (t, ^{3}J = 7.4 Hz, 3H, CH_3); ^{13}C NMR (100 MHz, $CDCl_3$, ppm) δ = 160.7 (1C, N*C*N), 138.6 (1C, C_q), 136.9 (1C, C_q), 131.6 (1C, *C*$H_{imidazole}$), 131.0 (1C, C_qCH(CH_3)$_2$), 130.3 (1C, CH_{ar}), 129.0 (1C, C_q), 127.0 (1C, *C*H_{ar}), 125.7 (1C, *C*H_{ar}), 115.3 (1C, *C*H_{ar}), 31.6 (1C, *C*H(CH_3)), 30.0 (1C, *C*H_2CH_2), 27.9 (1C, C_q*C*H_2), 22.5 (1C, *C*H_2CH_3), 20.8 (2C, CH(*C*H_3)$_2$), 13.9 (1C, *C*H_3); MS (EI, 70 eV, 50 °C), m/z (%): 269 [M+2]+ (30), 268 [M+1]+ (19), 267 [M]+ (100), 266 (16), 252 (71), 239 (55), 231 (22), 225 (16), 224 (50), 219 (37), 209 (23), 208 (20), 196 (20), 181 (36), 169 (56), 131 (41), 119 (42), 84 (15), 69 (96). HRMS (EI, $C_{17}H_{21}N_3$): calcd 267.1730, found 267.1732; IR (ATR, ṽ) = 2959 (s), 2928 (s), 2864 (m), 1707 (w), 1659 (w), 1606 (w), 1585 (w), 1536 (w), 1493 (s), 1465 (s), 1412 (s), 1378 (m), 1357 (m), 1319 (m), 1289 (m), 1184 (w), 1169 (w), 1156 (m), 1136 (m), 1081 (s), 1037 (w), 975 (w), 952 (m), 933 (w), 882 (w), 847 (w), 829 (m), 809 (w), 744 (vs), 700 (w), 640 (m), 632 (m), 606 (w), 584 (m), 531 (w), 479 (w), 455 (m), 416 (w) cm^{-1}.

^{1}H NMR (400 MHz, $CDCl_3$, ppm) δ = 7.91 (d, ^{3}J = 7.5 Hz, 1H, CH_{ar}), 7.65–7.61 (m, 1H, CH_{ar}), 7.54 (t, ^{3}J = 8.3 Hz, 1H, CH_{ar}), 7.42 (d, ^{3}J = 15.2 Hz, 1H, CH_{ar}), 5.12 (bs, 2H, NH_2), 3.09 (hept, ^{3}J = 6.7 Hz, 1H, CH(CH_3)$_2$), 1.42 (d, ^{3}J = 6.8 Hz, 6H, CH_3); ^{13}C NMR (100 MHz, $CDCl_3$, ppm) δ = 151.8 (1C, C_q), 150.2 (1C, C_q), 140.3 (1C, C_q), 137.9 (1C, C_q), 130.5 (1C, C_q), 129.0 (1C, *C*H_{ar}), 128.6 (1C, *C*H_{ar}), 125.4 (1C, *C*H_{ar}) 124.8 (1C, *C*H_{ar}), 31.4 (1C, *C*H(CH_3)$_2$), 20.3 (2C, *C*H_3); MS (ESI), m/z (%): 189 [M+1]$^+$ (11), 188.1181 [M]$^+$ (100). HRMS ($C_{11}H_{14}N_3$): calcd 188.1182, found 188.1181; IR (ATR, ṽ) = 3482 (m), 3303 (w), 3257 (vw), 3216 (w), 3106 (w), 3031 (w), 2973 (m), 2961 (m), 2929 (m), 2870 (w), 2737 (w), 1643 (vs), 1606 (m), 1562 (s), 1494 (w), 1463 (s), 1431 (vs), 1381 (s), 1351 (s), 1316 (m), 1251 (m), 1232 (m), 1193 (w), 1130 (s), 1072 (vs), 1041 (m), 1016 (m), 962 (w), 949 (m), 914 (m), 866 (w), 756 (vs), 722 (s), 704 (s), 657 (s), 611 (vs), 585 (m), 472 (m), 422 (w), 377 (s) cm^{-1}.

Additional information on the chemical synthesis is available via Chemotion repository: https://doi.org/10.14272/reaction/SA-FUHFF-UHFFFADPSC-OHPQSKQMZH-UHFFFADPSC-NUHFF-NUHFF-NUHFF-ZZZ

Additional information on the analysis of the target compound is available via Chemotion repository:
https://doi.org/10.14272/YKTZEDKRYOIMNU-UHFFFAOYSA-N.1
https://doi.org/10.14272/AFQHYVRVQPQNNN-UHFFFAOYSA-N.1
https://doi.org/10.14272/IRSRPTQGLZTLGL-UHFFFAOYSA-N.1

1-Butyl-4-(trifluoromethyl)imidazo[1,2-*a*]quinoxaline (16c), 3-(trifluoromethyl)quinoxalin-2-amine (17c)

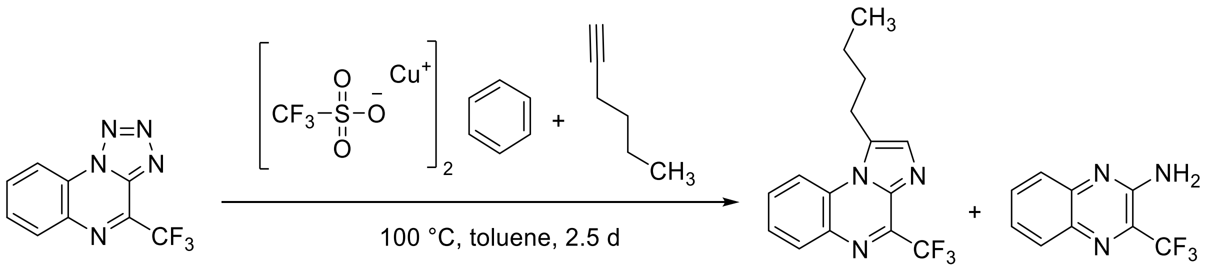

Name {P1|**16c**}: 1-butyl-4-(trifluoromethyl)imidazo[1,2-*a*]quinoxaline; Formula: $C_{15}H_{14}F_3N_3$; Molecular Mass: 293.2870; Exact Mass: 293.1140; Smiles: CCCCc1cnc2n1c1ccccc1nc2C(F)(F)F; InChIKey: YXIJMMYJFBYEEB-UHFFFAOYSA-N

Name {P2|**17c**}: 3-(trifluoromethyl)quinoxalin-2-amine; Formula: $C_9H_6F_3N_3$; Molecular Mass: 213.1592; Exact Mass: 213.0514; Smiles: Nc1nc2ccccc2nc1C(F)(F)F; InChIKey: STMGCUUVVSSYBM-UHFFFAOYSA-N

The starting material 4-(trifluoromethyl)tetrazolo[1,5-*a*]quinoxaline (49.0 mg, 205 µmol, 1.00 equiv) and the catalyst benzene;copper(1+);trifluoromethanesulfonate (10.0 mg, 19.9 µmol, 0.0970 equiv) were dissolved in 1 mL of dry toluene in a 5 mL crimp vial under argon, followed by hex-1-yne (34.3 mg, 48.0 µL, 418 µmol, 2.00 equiv). The reaction mixture was stirred at 100 °C for 2.5 days; then water and EtOAc were added, the organic phase was separated and the aqueous phase was extracted 3× with EtOAc. The combined organic phases were dried over Na_2SO_4, filtered and the solvent was removed under reduced pressure. The crude product was purified via column chromatography (dryload on Celite, *c*Hex -> *c*Hex/EtOAc 4:1) and 1-butyl-4-(trifluoromethyl)imidazo[1,2-*a*]quinoxaline (10.5 mg, 35.8 µmol, 17% yield) as well as 3-(trifluoromethyl)quinoxalin-2-amine (29.0 mg, 136 µmol, 66% yield) were obtained as brown solids.

R_f = 0.63 (cyclohexane/ethyl acetate 2:1). ^{1}H NMR (400 MHz, $CDCl_3$, ppm) δ = 8.28 (d, ^3J = 8.6 Hz, 1H, CH_{ar}), 8.23 (dd, ^3J = 8.2 Hz, ^4J = 1.7 Hz, 1H, CH_{ar}), 7.78–7.73 (m, 1H, CH_{ar}), 7.68 (s, 1H, C$H_{triazole}$), 7.68–7.63 (m, 1H, CH_{ar}), 3.34 (t, ^3J = 7.5 Hz, 2H, C_qCH_2), 1.92 (p, ^3J = 7.5 Hz, 2H, CH_2CH_2), 1.59 (h, ^3J = 7.4 Hz, 2H, CH_2CH_3), 1.06 (t, ^3J = 7.4 Hz, 3H, CH_3); ^{13}C NMR (100 MHz, $CDCl_3$, ppm) δ = 140.5 (q, ^2J = 36.0 Hz, 1C, CCF_3), 135.5 (1C, C_q), 134.8 (1C, C_q), 133.9 (1C, $CH_{imidazole}$), 132.2 (1C, $C_{imidazole}$), 131.9 (1C, CH_{ar}), 130.4 (1C, CH_{ar}), 130.0 (1C, C_q), 126.7 (1C, CH_{ar}), 120.4 (q, ^1J = 276.6 Hz, CF_3), 115.6 (1C, CH_{ar}), 29.9 (1C, CH_2), 27.7 (1C, CH_2C_q), 22.5 (1C, CH_2CH_3), 13.8 (1C, CH_3); ^{19}F NMR (376 MHz, $CDCl_3$, ppm) δ = -67.38; MS (EI, 70 eV, 40 °C, m/z): 294 (62) $[M+1]^+$, 266 (11), 253 (18), 252 (100), 251 (12), 238 (40), 232 (11), 213 (16). HRMS ($C_{15}H_{15}N_3F_3$): calcd 294.1213, found 294.1214; IR (ATR, ṽ) = 3037 (vw), 2962 (w), 2929 (w), 2867 (w), 1578 (w), 1551 (m), 1536 (w), 1494 (w), 1468 (m), 1458 (w), 1429 (w), 1408 (m), 1377 (m), 1316 (m), 1303 (m), 1258 (m), 1237 (m), 1230 (m), 1201 (s), 1183 (vs), 1129 (vs), 1072 (vs), 1055 (vs), 1035 (m), 926 (s), 871 (w), 850 (m), 809 (w), 761 (vs), 741 (vs), 718 (s), 659 (m), 633 (m), 591 (s), 569 (w), 528 (w), 476 (m), 452 (m) cm^{-1}; EA ($C_{15}H_{14}F_3N_3$): Calcd C 61.43; H 4.81; N 14.33. Found C 61.44; H 4.77; N 14.05.

^{1}H NMR (400 MHz, $CDCl_3$, ppm) δ = 8.02 (d, ^3J = 7.9 Hz, 1H, CH_{ar}), 7.76–7.71 (m, 2H, CH_{ar}), 7.56–7.52 (m, 1H, CH_{ar}), 5.32 (bs, 2H, NH_2); ^{13}C NMR (100 MHz, $CDCl_3$, ppm) δ = 148.5 (1C, C_q), 142.9 (1C, C_q), 135.9 (1C, C_q), 132.7 (1C, CH_{ar}), 131.5 (q, $^2J_{CCF3}$ = 35.4 Hz, CCF_3), 129.8 (1C, CH_{ar}), 126.3 (1C, CH_{ar}), 125.9 (1C, CH_{ar}), 122.8 (q, $^1J_{CF3}$ = 275.4 Hz, CF_3); ^{19}F NMR (376 MHz, $CDCl_3$, ppm) δ = -67.98; MS (EI, m/z, 70 eV, 20 °C): 214 $[M+1]^+$ (11), 213 $[M]^+$ (100), 166 (15), 144 (21), 117 (11), 90 (15). HRMS (EI, $C_9H_6N_3F_3$): calcd 213.0508, found 213.0507; IR (ATR, ṽ) = 3510 (w), 3310 (w), 3131 (w), 3063 (w), 2962 (w), 2924 (w), 2853 (w), 1649 (s), 1581 (m), 1561 (s), 1492 (m), 1479 (w), 1441 (s), 1361 (m), 1339 (s), 1317 (s), 1245 (m), 1222 (m), 1173 (vs), 1132 (vs), 1099 (vs), 1044 (vs), 1014 (vs), 960 (s), 914 (s), 798 (w), 758 (vs), 741 (vs), 722 (vs), 670 (vs), 620 (s), 605 (s), 582 (vs), 484 (m), 473 (m), 377 (vs) cm^{-1}.

Additional information on the chemical synthesis is available via Chemotion repository: https://doi.org/10.14272/reaction/SA-FUHFF-UHFFFADPSC-LFFREMWXTJ-UHFFFADPSC-NUHFF-NUHFF-NUHFF-ZZZ

Additional information on the analysis of the target compound is available via Chemotion repository:
https://doi.org/10.14272/YXIJMMYJFBYEEB-UHFFFAOYSA-N.1
https://doi.org/10.14272/STMGCUUVVSSYBM-UHFFFAOYSA-N.1

The synthesis of side product 3-(trifluoromethyl)quinoxalin-2-amine has been previously reported and the NMR spectra are corresponding with literature [28].

2-(4-Butyl-1*H*-1,2,3-triazol-1-yl)-3-phenylquinoxaline (15d), 3-phenylquinoxalin-2-amine (17d)

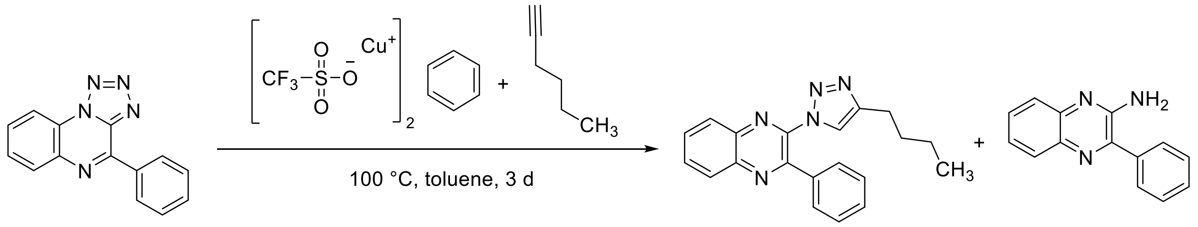

Name {P1|**15d**}: 2-(4-butyl-1*H*-1,2,3-triazol-1-yl)-3-phenylquinoxaline; Formula: $C_{20}H_{19}N_5$; Molecular Mass: 329.3984; Exact Mass: 329.1640; Smiles: CCCCc1nnn(c1)c1nc2ccccc2nc1c1ccccc1; InChIKey: XJGYBMKEUQIRGA-UHFFFAOYSA-N

Name {P2|**17d**}: 3-phenylquinoxalin-2-amine; Formula: $C_{14}H_{11}N_3$; Molecular Mass: 221.2572; Exact Mass: 221.0953; Smiles: Nc1nc2ccccc2nc1c1ccccc1; InChIKey: ABTZHDQWMUXIFW-UHFFFAOYSA-N

The starting material 4-phenyl-[1,2,3,4]tetrazolo[1,5-*a*]quinoxaline (50.0 mg, 202 µmol, 1.00 equiv) and the catalyst benzene;copper(1+);trifluoromethanesulfonate (12.6 mg, 25.0 µmol, 0.124 equiv) were dissolved in 1 mL of dry toluene under argon, followed by 1-hexyne (42.9 mg, 60.0 µL, 523 µmol, 2.58 equiv). The green reaction mixture was stirred at 100 °C for 3 days. Then water was added and the brown aqueous phase was extracted 4 times with DCM. The combined organic phases were dried over Na_2SO_4, filtered and the solvents were removed under reduced pressure. The obtained crude product was purified twice via flash-chromatography (Interchim

devices puriFLASH XS420) on silica gel (PF-15SIHP-F0025) using *c*Hex to *c*Hex/EtOAc 2:1 in 12 column volumes. The expected product 2-(4-butyl-1*H*-1,2,3-triazol-1-yl)-3-phenylquinoxaline (7.20 mg, 21.9 µmol) was obtained as a brown solid in 11% yield; 3-phenylquinoxalin-2-amine (10.9 mg, 49.3 µmol) was obtained as a brown solid in 24% yield. Moreover, 21 mg (42%) of starting material were reisolated.

R_f = 0.48 (cyclohexane/ethyl acetate 2:1). ^{1}H NMR (400 MHz, Chloroform-d [7.27 ppm], ppm) δ = 8.28–8.26 (m, 1H, CH_{Ar}), 8.20–8.18 (m, 1H, CH_{Ar}), 7.93–7.86 (m, 2H, CH_{Ar}), 7.66 (s, 1H, C*H*), 7.46–7.38 (m, 5H, CH_{Ar}), 2.78 (t, *J* = 7.6 Hz, 2H, CH_2), 1.72–1.66 (m, 2H, CH_2), 1.40–1.34 (m, 2H, CH_2), 0.96–0.91 (m, 3H, CH_3); ^{13}C NMR (100 MHz, Chloroform-d [77.0 ppm], ppm) δ = 149.6 (1C, C_q), 148.6 (1C, C_q), 142.5 (1C, C_q), 142.4 (1C, C_q), 139.8 (1C, C_q), 136.2 (1C, C_q), 131.5 (1C, CH_{Ar}), 131.2 (1C, CH_{Ar}), 129.8 (1C, CH_{Ar}), 129.4 (1C, CH_{Ar}), 129.1 (1C, CH_{Ar}), 128.7 (2C, CH_{Ar}), 128.5 (2C, CH_{Ar}), 121.6 (1C, CH_{Ar}), 31.3 (1C, CH_2), 25.2 (1C, CH_2), 22.1 (1C, CH_2), 13.8 (1C, CH_3); MS (EI, 70 eV, 130 °C), m/z (%): 329 $[M]^+$ (1), 301 (17), 300 (31), 273 (26), 272 (23), 258 (29), 220 (19), 219 (20), 206 (24), 205 (100). HRMS (EI, $C_{20}H_{19}N_5$): calcd 329.1635, found 329.1635; IR (ATR, ṽ) = 3146 (w), 3057 (vw), 2956 (m), 2924 (m), 2851 (m), 1725 (vw), 1599 (w), 1548 (w), 1486 (m), 1468 (m), 1449 (s), 1443 (s), 1377 (w), 1346 (m), 1286 (w), 1214 (m), 1179 (m), 1140 (w), 1130 (w), 1077 (w), 1060 (w), 1033 (vs), 1011 (s), 966 (vs), 929 (w), 919 (w), 885 (w), 819 (w), 803 (w), 793 (w), 764 (vs), 734 (m), 696 (vs), 670 (m), 620 (w), 609 (m), 589 (m), 562 (m), 537 (s), 492 (w), 449 (w), 387 (m) cm^{-1} EA ($C_{20}H_{19}N_5$): Calcd C 72.93; H 5.81; N 21.26. Found C 72.86; H 5.99; N 19.65; UV/VIS (acetonitrile), λ = 340 (2.06), 258 (2.96) nm.

^{1}H NMR (400 MHz, Chloroform-d [7.27 ppm], ppm) δ = 7.98 (d, *J* = 8.1 Hz, 1H, C*H*Ar), 7.81–7.76 (m, 2H, CH_{Ar}), 7.71 (d, *J* = 7.9 Hz, 1H, CH_{Ar}), 7.63–7.44 (m, 5H, CH_{Ar}), 5.15 (s, 2H, NH_2); 13C NMR (100 MHz, Chloroform-d [77.0 ppm], ppm) δ = 130.0 (1C, CH_{Ar}), 129.8 (1C, CH_{Ar}), 129.4 (1C, CH_{Ar}), 129.1 (2C, CH_{Ar}), 128.4 (2C, CH_{Ar}), 125.8 (1C, CH_{Ar}), 125.3 (1C, CH_{Ar}). Missing 5C (5C, C_q) due to low intensity. CH_{ar} peaks are consistent with literature [29]; MS (EI, 70 eV, 50 °C), m/z (%): 222 $[M+1]^+$ (13), 221 $[M]^+$ (78), 220 (100), 169 (15), 97 (15), 83 (15), 69 (32), 58 (17), 57 (18), 55 (15). HRMS (EI, $C_{14}H_{11}N_3$): calcd 221.0947, found 221.0947; IR (ATR, ṽ) = 3356 (w), 3306 (w), 3128 (w), 3085 (w), 3061 (w), 2953 (w), 2921 (w), 2851 (w), 2781 (w), 1645 (w), 1608 (w), 1557 (m), 1492 (w), 1470 (w), 1463 (w), 1445 (w), 1425 (s), 1370 (w), 1353 (m), 1329 (w), 1265 (m), 1238 (w), 1224 (w), 1174 (w), 1139 (w), 1125 (w), 1074 (w), 1044 (w), 1010 (m), 912 (m), 856 (w), 834 (w), 803 (w), 751 (vs), 718 (s), 687 (vs), 620 (m), 612 (s), 591 (s), 558 (m), 514 (s), 496 (s), 465 (vs), 438 (vs) cm^{-1}.

Additional information on the chemical synthesis is available via Chemotion repository: https://doi.org/10.14272/reaction/SA-FUHFF-UHFFFADPSC-BKLKYSKWAG-UHFFFADPSC-NUHFF-NUHFF-NUHFF-ZZZ

Additional information on the analysis of the target compound is available via Chemotion repository:
https://doi.org/10.14272/XJGYBMKEUQIRGA-UHFFFAOYSA-N.1
https://doi.org/10.14272/ABTZHDQWMUXIFW-UHFFFAOYSA-N.4

The synthesis of side product 3-phenylquinoxalin-2-amine has been previously reported and the NMR spectra are corresponding with literature [29].

1-Butyl-4-chloroimidazo[1,2-*a*]quinoxaline (16e), 3-chloroquinoxalin-2-amine (17e)

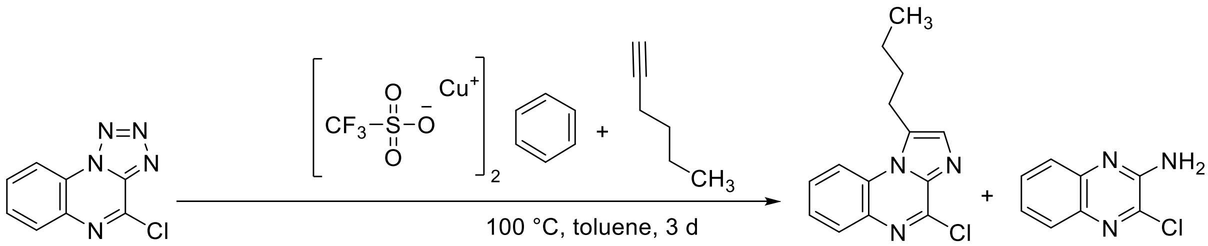

Name {P1|**16e**}: 1-butyl-4-chloroimidazo[1,2-*a*]quinoxaline; Formula: $C_{14}H_{14}ClN_3$; Molecular Mass: 259.7341; Exact Mass: 259.0876; Smiles: CCCCc1cnc2n1c1ccccc1nc2Cl; InChIKey: CWXIXBIIISVJRL-UHFFFAOYSA-N

Name {P2|**17e**}: 3-chloroquinoxalin-2-amine; Formula: $C_8H_6ClN_3$; Molecular Mass: 179.6063; Exact Mass: 179.0250; Smiles: Nc1nc2ccccc2nc1Cl; InChIKey: NOFJFBHOKPHILH-UHFFFAOYSA-N

The starting material 4-chlorotetrazolo[1,5-*a*]quinoxaline (150 mg, 730 µmol, 1.00 equiv) and the catalyst benzene;copper(1+);trifluoromethanesulfonate (73.4 mg, 146 µmol, 0.200 equiv) were dissolved in 5 mL of dry toluene in a two-necked flask under argon, followed by hex-1-yne (120 mg, 168 µL, 1.46 mmol, 2.00 equiv). The reaction mixture was stirred at 100 °C for 3 days; then water and ethyl acetate were added, the organic phase was separated and the aqueous phase was extracted 3× with ethyl acetate. The combined organic phases were dried over Na_2SO_4, filtered and the solvent was removed under reduced pressure. The crude mixture was purified twice via column chromatography (*c*Hex/EtOAc+2% Et_3N, then DCM/EtOAc) and 1-butyl-4-chloroimidazo[1,2-*a*]quinoxaline (11.0 mg, 42.4 µmol, 6% yield) and 3-chloroquinoxalin-2-amine (8.00 mg, 44.5 µmol, 6% yield) were obtained; 28 mg of starting material were reisolated (19%). Note: This reaction was conducted in a two-necked flask using 0.2 equiv. of catalyst. Under standard conditions (50 mg of starting material, crimp vial, 0.1 equiv. of catalyst), the desired product was isolated in mixture with the amine product; respective yields were calculated from the NMR ratios and gave a yield of 4% of 1-butyl-4-chloroimidazo[1,2-*a*]quinoxaline and 23% of 3-chloroquinoxalin-2-amine; 34% of the starting material were reisolated.

R_f = 0.41 (CH_2Cl_2/ethyl acetate 20:1). ^{1}H NMR (400 MHz, $CDCl_3$, ppm) δ = 8.20 (dd, ^3J = 8.3 Hz, ^4J = 1.6 Hz, 1H, CH_{ar}), 8.04 (dd, ^3J = 7.9 Hz, ^4J = 1.7 Hz, 1H, CH_{ar}), 7.66–7.58 (m, 2H, CH_{ar}), 7.57 (m, 1H, C$H_{imidazole}$), 3.29 (t, ^3J = 7.6 Hz, 2H, C_qCH_2), 1.90 (p, ^3J = 7.5 Hz, 2H, CH_2CH_2), 1.58 (h, ^3J = 7.3 Hz, 2H, C$H_2$$CH_3$), 1.05 (t, ^3J = 7.4 Hz, 3H, CH_3); ^{13}C NMR (100 MHz, $CDCl_3$ [77.0 ppm], ppm) δ = 143.7 (1C, C_q), 136.6 (1C, C_q), 135.7 (1C, C_q), 133.2 (1C, C_q), 133.1 (1C, $CH_{triazole}$), 130.1 (1C, CH_{ar}), 129.1 (1C, C_q), 128.5 (1C, CH_{ar}), 126.6 (1C, CH_{ar}), 115.6 (1C, CH_{ar}), 29.9 (1C, CH_2CH_2), 27.8 (1C, C_qCH_2), 22.5 (1C, CH_2CH_3), 13.8 (1C, CH_3). MS (EI, 70 eV, 90 °C), m/z (%): 259/261 $[M]^+$ (35/12), 218 (37), 217 (19), 216 (100), 204 (16), 102 (11). HRMS (EI, $C_{14}H_{14}N_3{}^{35}Cl_1$): calcd 259.0871, found 259.0870. IR (ATR, ṽ) = 2953 (w), 2917 (vs), 2849 (vs), 1737 (m), 1718 (w), 1526 (w), 1479 (m), 1465 (s), 1451 (s), 1398 (w), 1370 (m), 1347 (m), 1303 (w), 1285 (w), 1241 (s), 1169 (m), 1153 (m), 1130 (m), 1101 (w), 1077 (s), 1055 (m), 1018 (s), 962 (w), 919 (vs), 867 (w), 839 (m), 806 (m), 766 (vs), 744 (s), 730 (m), 720 (m), 688 (w), 636 (m), 592 (m), 582 (m), 470 (m), 453 (s) cm^{-1}.

^{1}H NMR (400 MHz, $CDCl_3$, ppm) δ = 7.86 (dd, ^3J = 8.3 Hz, ^4J = 1.6 Hz, 1H, CH_{ar}), 7.69 (dd, ^3J = 8.4 Hz, ^4J = 1.7 Hz, 1H, CH_{ar}), 7.65–7.61 (m, 1H, CH_{ar}), 7.49–7.45 (m, 1H, CH_{ar}), 5.50 (bs, 2H, NH_2) Pure spectrum and further analysis available at https://dx.doi.org/10.14272/reaction/SA-FUHFF-UHFFFADPSC-NOFJFBHOKP-UHFFFADPSC-NUHFF-NUHFF-NUHFF-ZZZ.1.

Additional information on the chemical synthesis is available via Chemotion repository: https://doi.org/10.14272/reaction/SA-FUHFF-UHFFFADPSC-SAOWBEPSEQ-UHFFFADPSC-NUHFF-NUHFF-NUHFF-ZZZ

Additional information on the analysis of the target compound is available via Chemotion repository:
https://doi.org/10.14272/CWXIXBIIISVJRL-UHFFFAOYSA-N.1
https://doi.org/10.14272/NOFJFBHOKPHILH-UHFFFAOYSA-N.3

The synthesis of side product 3-chloroquinoxalin-2-amine has been previously reported and the NMR spectra are corresponding with literature [30].

2-(4-Butyl-1*H*-1,2,3-triazol-1-yl)-3-methoxyquinoxaline (15f)

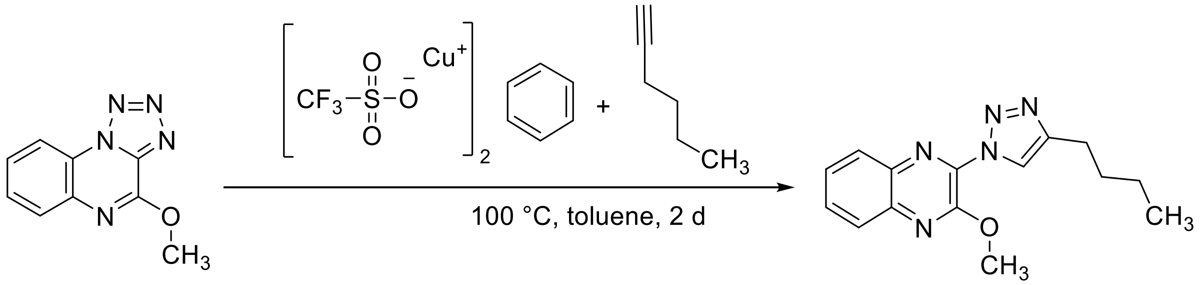

Name {P1|**15f**}: 2-(4-butyl-1*H*-1,2,3-triazol-1-yl)-3-methoxyquinoxaline; Formula: $C_{15}H_{17}N_5O$; Molecular Mass: 283.3284; Exact Mass: 283.1433; Smiles: CCCCc1nnn(c1)c1nc2ccccc2nc1OC; InChIKey: LDJGUGHGVWKPPC-UHFFFAOYSA-N

The starting material 4-methoxy-[1,2,3,4]tetrazolo[1,5-*a*]quinoxaline (43.5 mg, 216 µmol, 1.00 equiv) and the catalyst benzene;copper(1+);trifluoromethanesulfonate (17.2 mg, 34.2 µmol, 0.158 equiv) were dissolved in 1 mL of dry toluene under argon, followed by 1-hexyne (35.8 mg, 50.0 µL, 435 µmol, 2.01 equiv). The dark reaction mixture was stirred at 100 °C for 2 days, then water was added and the brown aqueous phase was extracted 4 times with DCM. The combined organic phases were dried over Na_2SO_4, filtered and the solvents were removed under reduced pressure. The obtained crude product was purified via HPLC (acetonitrile/water); the expected product 2-(4-butyl-1*H*-1,2,3-triazol-1-yl)-3-methoxyquinoxaline (30.2 mg, 107 µmol) was obtained as a yellow oil in 49% yield.

R_f = 0.15 (CH_2Cl_2/MeOH 50:1). ^{1}H NMR (400 MHz, Chloroform-d [7.27 ppm], ppm) δ = 8.19 (s, 1H, CH), 8.08 (d, J = 8.2 Hz, 1H, CH_{Ar}), 7.92 (d, J = 8.3 Hz, 1H, CH_{Ar}), 7.74 (t, J = 7.5 Hz, 1H, CH_{Ar}), 7.64 (t, J = 7.6 Hz, 1H, CH_{Ar}), 4.24 (s, 3H, OCH_3), 2.86 (t, J = 7.8 Hz, 2H, CH_2), 1.76 (quint, J = 7.6 Hz, 2H, CH_2), 1.45 (q, J = 7.5 Hz, 2H, CH_2), 0.97 (t, J = 7.3 Hz, 3H, CH_3); ^{13}C NMR (100 MHz, Chloroform-d [77.0 ppm], ppm) δ = 150.5 (1C, C_q), 148.2 (1C, C_q), 140.3 (1C, C_q), 137.0 (1C, C_q), 135.6 (1C, C_q), 130.9

(1C, CH_{Ar}), 128.9 (1C, CH_{Ar}), 127.9 (1C, CH_{Ar}), 126.8 (1C, CH_{Ar}), 121.7 (1C, CH), 54.9 (1C, OCH_3), 31.5 (1C, CH_2), 25.3 (1C, CH_2), 22.3 (1C, CH_2), 13.9 (1C, CH_3); MS (EI, 70 eV, 100 °C), m/z (%): 283 $[M]^+$ (1), 255 (19), 254 (23), 240 (26), 227 (69), 226 (65), 213 (23), 212 (94), 187 (29), 159 (38), 144 (26), 131 (18), 130 (25), 129 (100), 116 (18), 90 (25). HRMS (EI, $C_{15}H_{17}O_1N_5$): calcd 283.1428, found 283.1427; IR (ATR, ṽ) = 3166 (w), 3061 (w), 2956 (m), 2927 (m), 2857 (m), 1611 (w), 1581 (w), 1568 (w), 1460 (vs), 1421 (s), 1412 (s), 1390 (s), 1378 (s), 1332 (vs), 1298 (s), 1227 (s), 1208 (s), 1190 (s), 1167 (vs), 1139 (s), 1038 (vs), 1003 (s), 987 (vs), 973 (vs), 919 (m), 904 (w), 873 (w), 822 (m), 807 (m), 788 (w), 768 (vs), 742 (s), 730 (m), 714 (m), 687 (w), 662 (w), 649 (w), 628 (m), 603 (m), 591 (w), 561 (w), 497 (s), 476 (s), 397 (w) cm^{-1}; EA ($C_{15}H_{17}N_5O$): Calcd C 63.59; H 6.05; N 24.72. Found C 63.57; H 6.16; N 23.70.

Additional information on the chemical synthesis is available via Chemotion repository: https://doi.org/10.14272/reaction/SA-FUHFF-UHFFFADPSC-LDJGUGHGVW-UHFFFADPSC-NUHFF-NUHFF-NUHFF-ZZZ.1

Additional information on the analysis of the target compound is available via Chemotion repository: https://doi.org/10.14272/LDJGUGHGVWKPPC-UHFFFAOYSA-N.2

1-(4-((3-(4-Butyl-1*H*-1,2,3-triazol-1-yl)quinoxalin-2-yl)amino)phenyl)ethan-1-one (15g), 1-(4-((3-aminoquinoxalin-2-yl)amino)phenyl)ethan-1-one (17g)

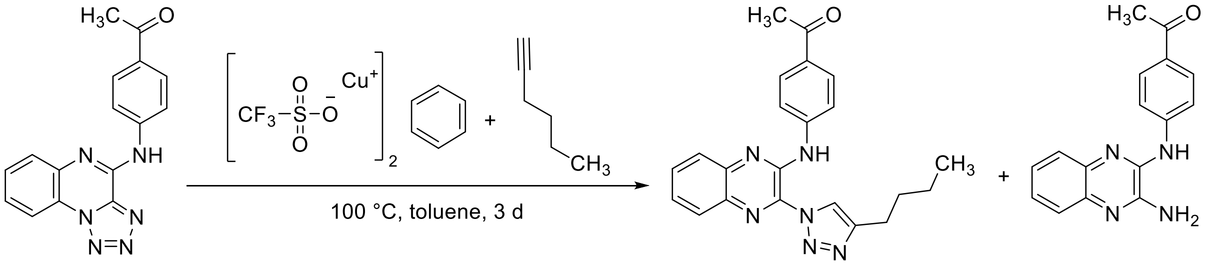

Name {P1|**15g**}: 1-(4-((3-(4-butyl-1*H*-1,2,3-triazol-1-yl)quinoxalin-2-yl)amino)phenyl)ethan-1-one; Formula: $C_{22}H_{22}N_6O$; Molecular Mass: 386.4497; Exact Mass: 386.1855; Smiles: CCCCc1nnn(c1)c1nc2ccccc2nc1Nc1ccc(cc1)C(=O)C; InChIKey: WGMOCILRUYTMIW-UHFFFAOYSA-N

Name {P2|**17g**}: 1-(4-((3-aminoquinoxalin-2-yl)amino)phenyl)ethan-1-one; Formula: $C_{16}H_{14}N_4O$; Molecular Mass: 278.3086; Exact Mass: 278.1168; Smiles: Nc1nc2ccccc2nc1Nc1ccc(cc1)C(=O)C; InChIKey: BLJPCFCXXBZYPN-UHFFFAOYSA-N

1-[4-(Tetrazolo[1,5-*a*]quinoxalin-4-ylamino)phenyl]ethanone (50.0 mg, 164 µmol, 1.00 equiv) and the catalyst benzene;copper(1+);trifluoromethanesulfonate (7.00 mg, 13.9 µmol, 0.0846 equiv) were dissolved in 1 mL of dry toluene under argon, followed by addition of hex-1-yne (33.7 mg, 47.2 µL, 411 µmol, 2.50 equiv). The reaction mixture was stirred at 100 °C for 3 days. Then, water and EtOAc were added, the organic phase was separated and the aqueous phase was extracted 3× with EtOAc. The combined organic phases were dried over Na_2SO_4, filtered and the solvent was removed under reduced pressure. The crude mixture was purified via column chromatography (dryload on Celite, *c*Hex -> EtOAc) and 1-(4-((3-(4-butyl-1*H*-1,2,3-triazol-1-yl)quinoxalin-2-yl)amino)phenyl)ethan-1-one (5.00 mg, 12.9 µmol, 8% yield)

was obtained as a yellow solid. 1-(4-((3-aminoquinoxalin-2-yl)amino)phenyl)ethan-1-one (4.00 mg, 14.4 µmol, 9% yield) was obtained as a yellow-brown colored solid and 29 mg of the starting material were re-isolated.

R_f = 0.52 (cyclohexane/ethyl acetate 2:1). ^{1}H NMR (400 MHz, $CDCl_3$, ppm) δ = 11.15 (s, 1H, NH), 8.70 (s, 1H, C*H*$_{triazol}$), 8.14–8.12 (m, 2H, C*H*$_{ar}$), 8.06–8.03 (m, 2H, C*H*$_{ar}$), 7.95–7.90 (m, 2H, C*H*$_{ar}$), 7.75–7.71 (m, 1H, C*H*$_{ar}$), 7.60–7.56 (m, 1H, C*H*$_{ar}$), 2.91 (t, ^3J = 7.5 Hz, 2H, C_qCH_2), 2.63 (s, 3H, $COCH_3$), 1.82 (p, ^3J = 7.5 Hz, 2H, $CH_2CH_2CH_2$), 1.50 (h, ^3J = 7.3 Hz, 2H, CH_2CH_3), 1.01 (t, ^3J = 7.4 Hz, 3H, CH_3); ^{13}C NMR (100 MHz, $CDCl_3$, ppm) δ = 196.8 (1C, *C*O), 143.8 (1C, C_q), 141.3 (1C, C_q), 140.2 (1C, C_q), 134.9 (1C, C_q), 132.8 (1C, C_q), 132.0 (1C, C_q), 130.8 (1C, CH_{ar}), 129.8 (2C, CH_{ar}), 128.1 (1C, CH_{ar}), 127.1 (1C, CH_{ar}), 126.6 (1C, CH_{ar}), 120.6 (1C, $CH_{triazole}$), 119.4 (2C, CH_{ar}), 31.2 (1C, $CH_2CH_2CH_2$), 26.4 (1C, $COCH_3$), 25.2 (C_qCH_2), 22.3 (1C, CH_2CH_3), 13.8 (1C, CH_3). Missing 1C (1C, $C_{triazole}$) due to low intensity; MS (EI, 70 eV, 200 °C), m/z (%): 386 $[M]^+$ (6), 316 (24), 315 (100), 271 (11), 221 (15), 220 (80), 219 (14), 90 (21). HRMS (EI, $C_{22}H_{22}O_1N_6$): calcd 386.1850, found 386.1851. IR (ATR, ṽ) = 3262 (w), 3179 (w), 3140 (w), 3118 (w), 3070 (w), 2959 (m), 2919 (m), 2851 (m), 1674 (s), 1621 (m), 1601 (vs), 1572 (m), 1536 (vs), 1507 (s), 1483 (s), 1472 (m), 1439 (vs), 1407 (s), 1356 (vs), 1305 (m), 1268 (vs), 1251 (vs), 1234 (vs), 1214 (vs), 1173 (vs), 1137 (vs), 1123 (s), 1030 (vs), 1018 (vs), 989 (vs), 959 (vs), 939 (s), 902 (m), 866 (m), 834 (vs), 823 (vs), 793 (m), 756 (vs), 730 (s), 722 (s), 696 (vs), 684 (vs), 635 (vs), 622 (s), 602 (vs), 589 (vs), 564 (vs), 506 (m), 490 (vs), 479 (vs), 465 (vs), 388 (s) cm^{-1}.

^{1}H NMR (400 MHz, DMSO-d_6, ppm) δ = 9.20 (bs, 1H, N*H*), 8.14–8.12 (m, 2H, C*H*$_{ar}$), 8.00–7.98 (m, 2H, C*H*$_{ar}$), 7.60 (dd, ^3J = 7.8 Hz, ^3J = 1.7 Hz, 1H, C*H*$_{ar}$), 7.48–7.46 (m, 1H, C*H*$_{ar}$), 7.39–7.29 (m, 2H, C*H*$_{ar}$), 3.36 (bs, 2H, NH_2), 2.55 (s, 3H, CH_3); ^{13}C NMR (100 MHz, DMSO-d_6, ppm) δ = 196.3 (1C, *C*O), 130.6 (1C, C_q), 129.4 (2C, CH_{ar}), 125.9 (1C, CH_{ar}), 125.8 (1C, CH_{ar}), 124.2 (1C, CH_{ar}), 118.7 (2C, CH_{ar}), 26.4 (1C, CH_3). Missing C (6C, C_q/CH_{ar}) due to low intensity. MS (EI, 70 eV, 170 °C), m/z (%): 279 [M+1]+ (20), 278 [M]+ (100), 277 (69), 271 (16), 263 (40), 255 (16), 246 (15), 235 (31), 144 (21), 133 (15), 109 (78), 105 (28), 102 (20), 90 (31), 84 (15), 83 (15), 66 (20), 59 (22), 57 (59), 55 (21). HRMS (EI, $C_{16}H_{14}O_1N_4$): calcd 278.1162, found 278.1163. IR (ATR, ṽ) = 3289 (w), 3116 (m), 3064 (m), 2959 (w), 2922 (w), 2853 (w), 2806 (w), 1687 (w), 1670 (s), 1657 (s), 1601 (vs), 1562 (m), 1534 (vs), 1510 (vs), 1496 (vs), 1476 (vs), 1465 (vs), 1409 (s), 1387 (m), 1357 (s), 1339 (vs), 1307 (m), 1264 (vs), 1244 (vs), 1201 (s), 1174 (vs), 1146 (s), 1125 (s), 1044 (s), 1020 (vs), 990 (vs), 958 (vs), 911 (s), 832 (vs), 748 (vs), 720 (vs), 632 (vs), 606 (vs), 589 (vs), 560 (vs), 528 (vs), 476 (vs), 456 (vs), 426 (vs), 387 (s) cm^{-1}.

Additional information on the chemical synthesis is available via Chemotion repository: https://doi.org/10.14272/reaction/SA-FUHFF-UHFFFADPSC-XXYVKQPHIB-UHFFFADPSC-NUHFF-NUHFF-NUHFF-ZZZ

Additional information on the analysis of the target compound is available via Chemotion repository:
https://doi.org/10.14272/WGMOCILRUYTMIW-UHFFFAOYSA-N.1
https://doi.org/10.14272/BLJPCFCXXBZYPN-UHFFFAOYSA-N.1

2-(4-Butyl-1*H*-1,2,3-triazol-1-yl)-3-((3,3,4,4,5,5,6,6,7,7,8,8,9,9,10,10,10-heptadecafluorodecyl)oxy)quinoxaline (15h), 3-((3,3,4,4,5,5,6,6,7,7,8,8,9,9,10,10,10-heptadecafluorodecyl)oxy)quinoxalin-2-amine (17h), 1-butyl-4-((3,3,4,4,5,5,6,6,7,7,8,8,9,9,10,10,10-heptadecafluorodecyl)oxy)imidazo[1,2-a]quinoxaline (16h)

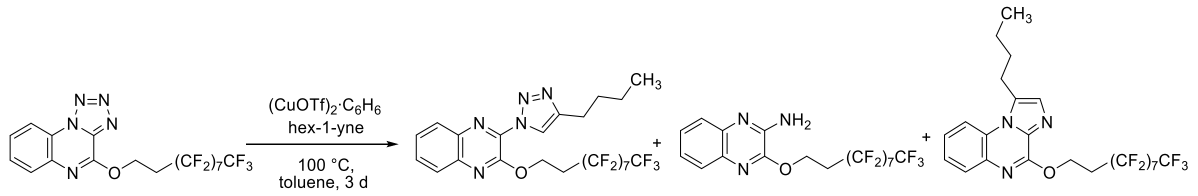

Name {P1|**15h**}: 2-(4-butyl-1*H*-1,2,3-triazol-1-yl)-3-((3,3,4,4,5,5,6,6,7,7,8,8,9,9,10,10,10-heptadecafluorodecyl)oxy)quinoxaline; Formula: $C_{24}H_{18}F_{17}N_5O$; Molecular Mass: 715.4055; Exact Mass: 715.1240; Smiles: CCCCc1nnn(c1)c1nc2ccccc2nc1OCCC(C(C(C(C(C(C(C(F)(F)F)(F)F)(F)F)(F)F)(F)F)(F)F)(F)F)(F)F; InChIKey: HZQOUMBELHPHLV-UHFFFAOYSA-N

Name {P2|**17h**}: 3-((3,3,4,4,5,5,6,6,7,7,8,8,9,9,10,10,10-heptadecafluorodecyl)oxy)quinoxalin-2-amine; Formula: $C_{18}H_{10}F_{17}N_3O$; Molecular Mass: 607.2644; Exact Mass: 607.0552; Smiles: Nc1nc2ccccc2nc1OCCC(C(C(C(C(C(C(C(F)(F)F)(F)F)(F)F)(F)F)(F)F)(F)F)(F)F)(F)F; InChIKey: VVNPRYRWMNIXCI-UHFFFAOYSA-N

Name {P3|**16h**}: 1-butyl-4-((3,3,4,4,5,5,6,6,7,7,8,8,9,9,10,10,10-heptadecafluorodecyl)oxy)imidazo[1,2-*a*]quinoxaline; Formula: $C_{24}H_{18}F_{17}N_3O$; Molecular Mass: 687.3921; Exact Mass: 687.1178; Smiles: CCCCc1cnc2n1c1ccccc1nc2OCCC(C(C(C(C(C(C(C(F)(F)F)(F)F)(F)F)(F)F)(F)F)(F)F)(F)F)(F)F; InChIKey: ICMDLGQTBHAPKS-UHFFFAOYSA-N

The starting material 4-((3,3,4,4,5,5,6,6,7,7,8,8,9,9,10,10,10-heptadecafluorodecyl)oxy)tetrazolo[1,5-*a*]quinoxaline (50.0 mg, 79.0 µmol, 1.00 equiv) and the catalyst benzene;copper(1+);trifluoromethanesulfonate (5.50 mg, 10.9 µmol, 0.138 equiv) were dissolved in 1 mL of dry toluene in a crimp vial under argon, followed by hex-1-yne (32.4 mg, 45.3 µL, 395 µmol, 5.00 equiv). The reaction mixture was stirred at 100 °C for 3 days; then water and ethyl acetate were added, the organic phase was separated and the aqueous phase was extracted 3× with EtOAc. The combined organic phases were dried over Na_2SO_4, filtered and the solvent was removed under reduced pressure. The crude mixture was purified via column chromatography (dryload on Celite, *c*Hex -> cHex/EtOAc 4:1) and 2-(4-butyl-1*H*-1,2,3-triazol-1-yl)-3-((3,3,4,4,5,5,6,6,7,7,8,8,9,9,10,10,10-heptadecafluorodecyl)oxy)quinoxaline (28.0 mg, 39.1 µmol, 50% yield), 3-((3,3,4,4,5,5,6,6,7,7,8,8,9,9,10,10,10-heptadecafluorodecyl)oxy)quinoxalin-2-amine (10.0 mg, 16.5 µmol, 21% yield, minor impurities) and 1-butyl-4-((3,3,4,4,5,5,6,6,7,7,8,8,9,9,10,10,10-heptadecafluorodecyl)oxy)imidazo[1,2-a]quinoxaline (8.00 mg, 11.6 µmol, 15% yield) were obtained as white to yellow solids.

R_f = 0.68 (cyclohexane/ethyl acetate 2:1). ^{1}H NMR (400 MHz, $CDCl_3$, ppm) δ = 8.17 (s, 1H, C$H_{triazole}$), 8.13 (dd, ^3J = 8.3 Hz, ^4J = 1.7 Hz, 1H, CH_{ar}), 7.94 (dd, ^3J = 8.3 Hz, ^4J = 1.6 Hz, 1H, CH_{ar}), 7.81–7.77 (m, 1H, CH_{ar}), 7.72–7.68 (m, 1H, CH_{ar}), 4.97 (t, ^3J =

6.5 Hz, 2H, OCH_2), 2.87 (t, ^3J = 7.8 Hz, 2H, CH_2C$_q$), 2.83–2.72 (m, 2H, OCH$_2$CH_2), 1.80–1.72 (m, 2H, CH$_2$CH_2), 1.46 (h, ^3J = 7.4 Hz, 2H, CH_2CH$_3$), 0.97 (t, ^3J = 7.3 Hz, 3H, CH_3); ^{13}C NMR (100 MHz, CDCl$_3$ [77.0 ppm], ppm) δ = 149.1 (1C, C_q), 148.3 (1C, C_q), 139.9 (1C, C_q), 137.3 (1C, C_q), 135.4 (1C, C_q), 131.2 (1C, CH$_{ar}$), 129.0 (1C, CH$_{ar}$), 128.4 (1C, CH$_{ar}$), 126.8 (1C, CH$_{ar}$), 121.6 (1C, CH$_{triazole}$), 59.6 (1C, OCH$_2$), 31.3 (1C, CH$_2$$CH_2$), 30.56 (t, ^3J = 21.6 Hz, 1C, CH$_2$CF$_2$), 25.2 (1C, C$_q$$CH_2$), 22.2 (1C, CH$_2CH_3$), 13.7 (1C, CH$_3$). Missing 8C (8C, CF) due to C-F-coupling and resulting low intensity; ^{19}F NMR (376 MHz, CDCl3, ppm) δ = -80.7 (t, ^3J = 10.2 Hz, 3F, CF$_3$), -113.2 (t, ^3J = 14.3 Hz, CF$_2$), -121.6 (m, CF_2), -121.8 (m, CF_2), -121.9 (m, CF_2), -122.7 (m, CF_2), -123.4 (m, CF_2), -126.1 (m, CF_2). MS (EI, 70 eV, 110 °C), m/z (%): 715 [M]+ (1), 696 (16), 688 (24), 687 (78), 672 (20), 660 (29), 659 (100), 658 (68), 645 (30), 644 (49), 619 (35), 607 (47), 591 (27), 284 (17), 256 (16), 145 (65), 111 (17), 99 (15), 97 (21), 85 (23), 83 (20), 71 (23), 69 (27), 57 (33), 55 (20). HRMS (EI, $C_{24}H_{18}O_1N_5F_{17}$): calcd 715.1234, found 715.1233. IR (ATR, ṽ) = 3165 (vw), 2963 (w), 2921 (w), 2851 (vw), 1582 (vw), 1451 (m), 1404 (vw), 1367 (w), 1333 (m), 1198 (vs), 1143 (vs), 1115 (s), 1082 (m), 1055 (m), 1043 (m), 994 (m), 963 (m), 929 (w), 873 (w), 820 (w), 789 (w), 769 (s), 730 (w), 703 (m), 656 (s), 622 (m), 605 (m), 577 (m), 560 (m), 531 (m), 511 (s), 397 (m), 381 (w) cm^{-1}.

^{1}H NMR (400 MHz, CDCl$_3$, ppm) δ = 7.71 (d, ^3J = 8.1 Hz, 1H, CH_{ar}), 7.63 (d, ^3J = 8.4 Hz, 1H, CH_{ar}), 7.47 (t, ^3J = 6.7 Hz, 1H, CH_{ar}), 7.41–7.37 (m, 1H, CH_{ar}), 5.31 (bs, 2H, NH_2), 4.88 (t, ^3J = 6.3 Hz, 2H, OCH_2), 2.80–2.67 (m, 2H, CH$_2$CH_2); ^{13}C NMR (100 MHz, CDCl3 [77.0 ppm], ppm) δ = 127.4 (1C, CH$_{ar}$), 125.3 (1C, CH$_{ar}$), 58.8 (1C, OCH$_2$), 30.6 (t, ^2J = 22.3 Hz, 1C, CH$_2$CF$_2$). Missing C (14C, CF$_2$CF$_3$ and C$_q$) due to C-F-coupling and resulting low intensity; ^{19}F NMR (376 MHz, CDCl$_3$, ppm) δ = -80.75 (t, ^3J = 9.9 Hz, 3F, CF_3), -113.2 (m, CF_2), -121.6 (m, CF_2), -121.9 (m, CF_2), -122.7 (m, CF_2), -123.4 (m, CF_2), -126.1 (m, CF_2); MS (FAB, 3-NBA), m/z (%): 688 (33), 609 $[M+2]^+$ (23), 608 $[M+1]^+$ (100), 607 $[M]^+$ (32), 162 (31). HRMS (FAB, $C_{18}H_{11}O_1N_3F_{17}$): calcd 608.0625, found 608.0623; IR (ATR, ṽ) = 3461 (w), 3293 (vw), 3257 (vw), 3146 (vw), 1640 (w), 1605 (w), 1587 (vw), 1519 (w), 1503 (w), 1486 (m), 1468 (m), 1453 (w), 1397 (w), 1371 (w), 1339 (w), 1323 (w), 1283 (w), 1234 (s), 1197 (vs), 1146 (vs), 1135 (vs), 1116 (vs), 1081 (w), 1061 (w), 1043 (m), 1006 (w), 953 (m), 919 (w), 894 (w), 861 (w), 846 (w), 761 (s), 704 (m), 652 (s), 606 (s), 574 (m), 558 (s), 544 (m), 528 (s), 419 (s), 404 (s) cm^{-1}.

^{1}H NMR (400 MHz, CDCl$_3$, ppm) δ = 8.16 (dd, ^3J = 8.2 Hz, ^4J = 1.6 Hz, 1H, CH_{ar}), 7.89 (dd, ^3J = 7.8 Hz, ^4J = 1.8 Hz, 1H, CH_{ar}), 7.53 (qd, ^3J = 7.6 Hz, ^4J = 1.7 Hz, 2H, CH_{ar}), 7.47 (s, 1H, C$H_{imidazole}$), 4.97 (t, ^3J = 7.1 Hz, 2H, OCH_2), 3.28 (t, ^3J = 7.6 Hz, 2H, C$_q$CH_2), 2.96–2.70 (m, 2H, OCH$_2$CH_2), 1.90 (p, ^3J = 7.7 Hz, 2H, CH$_2$CH_2), 1.58 (h, ^3J = 7.3 Hz, 2H, CH_2CH$_3$), 1.04 (t, ^3J = 7.3 Hz, 3H, CH_3) ^{13}C NMR (100 MHz, CDCl$_3$ [77.0 ppm], ppm) δ = 152.12 (1C, C_q), 135.27 (1C, C_q), 132.72 (1C, C_q), 132.21 (1C, C_q), 131.72 (1C, CH$_{triazole}$), 128.70 (1C, CH$_{ar}$), 128.23 (1C, C_q), 126.20 (1C, CH$_{ar}$), 125.70 (1C, CH$_{ar}$), 115.40 (1C, CH$_{ar}$), 58.65 (1C, OCH$_2$), 30.8 (t, ^2J = 21.6 Hz, 1C, OCH$_2$$CH_2CF_2$), 29.99 (1C, CH$_2$$CH_2$), 27.70 (1C, C$_q$$CH_2$), 22.48 (1C, CH$_2CH_3$), 13.83 (1C, CH$_3$). Missing C (8C, CF$_2$/CF$_3$) due to C-F coupling and resulting low intensity; ^{19}F NMR (376 MHz, CDCl$_3$, ppm) δ = -80.8 (t, ^3J = 9.9 Hz, 3F, CF_3), -113.3 (p, J = 18.1 Hz, 2F, CF_2), -121.6 (m, CF_2), -121.9 (m, CF_2), -122.7 (m, CF_2), -123.4 (m, CF_2), -126.1 (m, CF_2); MS (FAB, 3-NBA), m/z (%): 689 $[M+1]^+$(27), 688 $[M]^+$(100), 242 (17). HRMS ($C_{24}H_{19}F_{17}N_3O$): calcd 688.1251, found 688.1249; IR (ATR, ṽ) = 2959 (vw), 2932 (w), 2860 (vw), 1619 (vw), 1561 (w), 1540 (w), 1509 (s), 1463 (w), 1418 (w), 1357 (m), 1330 (w), 1316 (w), 1295 (w), 1248 (s), 1198 (vs), 1143 (vs), 1130 (vs),

1113 (vs), 1082 (s), 1060 (s), 1021 (w), 1010 (w), 999 (w), 984 (m), 959 (w), 935 (w), 908 (vw), 857 (w), 839 (w), 807 (w), 762 (s), 747 (m), 717 (w), 701 (w), 650 (s), 639 (vs), 606 (w), 575 (w), 558 (m), 530 (m), 513 (s), 473 (w), 453 (w), 397 (m) cm^{-1}.

Additional information on the chemical synthesis is available via Chemotion repository: https://doi.org/10.14272/reaction/SA-FUHFF-UHFFFADPSC-GODAPXMMMY-UHFFFADPSC-NUHFF-NUHFF-NUHFF-ZZZ

Additional information on the analysis of the target compound is available via Chemotion repository:
https://doi.org/10.14272/HZQOUMBELHPHLV-UHFFFAOYSA-N.1
https://doi.org/10.14272/VVNPRYRWMNIXCI-UHFFFAOYSA-N.1
https://doi.org/10.14272/ICMDLGQTBHAPKS-UHFFFAOYSA-N.1

[1,2,3,4]Tetrazolo[1,5-*a*]quinoxaline (11a), 2-(4-butyl-1*H*-1,2,3-triazol-1-yl)quinoxaline (14k)

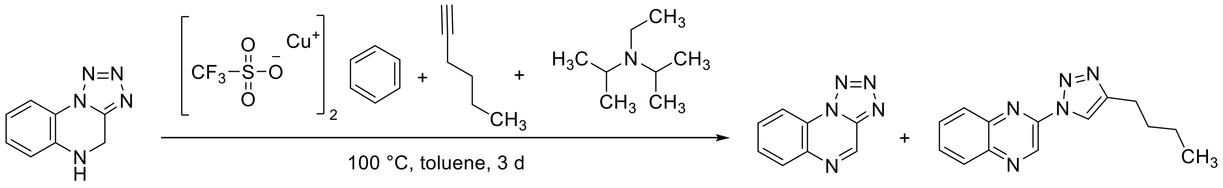

Name {P1|**11a**}: [1,2,3,4]tetrazolo[1,5-*a*]quinoxaline; Formula: $C_8H_5N_5$; Molecular Mass: 171.1588; Exact Mass: 171.0545; Smiles: c1ccc2c(c1)n1nnnc1cn2; InChIKey: LGMVEBQKPYIMMI-UHFFFAOYSA-N

Name {P2|**14k**}: 2-(4-butyl-1*H*-1,2,3-triazol-1-yl)quinoxaline; Formula: $C_{14}H_{15}N_5$; Molecular Mass: 253.3024; Exact Mass: 253.1327; Smiles: CCCCc1nnn(c1)c1cnc2c(n1)cccc2; InChIKey: AHSWENVYXHLEHF-UHFFFAOYSA-N

The starting material 4,5-dihydrotetrazolo[1,5-*a*]quinoxaline (49.7 mg, 287 µmol, 1.00 equiv) and the catalyst benzene;copper(1+);trifluoromethanesulfonate (15.4 mg, 30.6 µmol, 0.107 equiv) were dissolved in 1 mL of dry toluene under argon, followed by 1-hexyne (57.2 mg, 80.0 µL, 697 µmol, 2.41 equiv) and *N*-ethyl-*N*-propan-2-ylpropan-2-amine (76.0 mg, 100 µL, 588 µmol, 2.04 equiv). The orange reaction mixture was stirred at 100 °C for 3 days. Then water was added and the dark aqueous phase was extracted 4 times with DCM. The combined organic phases were dried over Na_2SO_4, filtered and the solvents were removed under reduced pressure. The obtained crude mixture was purified via flash-chromatography (Interchim devices puriFLASH XS420) on silica gel (PF-15SIHP-F0012) using *c*Hex to *c*Hex/ethyl acetate 2:1 in 12 column volumes. The products [1,2,3,4]tetrazolo[1,5-*a*]quinoxaline (9.50 mg, 55.5 µmol, 19% yield) and 2-(4-butyl-1*H*-1,2,3-triazol-1-yl)quinoxaline (16.4 mg, 64.7 µmol, 23% yield) were obtained as brown solids. Moreover 7 mg of an impure compound (presumably 3,4-dihydroquinoxalin-2-amine) were obtained, but not analyzed further.

^{1}H NMR (400 MHz, Chloroform-d [7.27 ppm], ppm) δ = 9.58 (s, 1H, CH), 8.67 (dd, ^3J = 8.2 Hz, ^4J = 1.5 Hz, 1H, CH_{ar}), 8.34 (dd, J = 8.2 Hz, ^4J = 1.5 Hz, 1H, CH_{ar}), 7.98–

7.88 (m, 2H, CH_{ar}) Further analysis can be found at https://dx.doi.org/10.14272/reaction/SA-FUHFF-UHFFFADPSC-LGMVEBQKPY-UHFFFADPSC-NUHFF-NUHFF-NUHFF-ZZZ.1.

^{1}H NMR (400 MHz, Chloroform-d [7.27 ppm], ppm) δ = 9.83 (s, 1H, CH), 8.48 (s, 1H, CH), 8.21–8.19 (m, 1H, CH_{Ar}), 8.07–8.05 (m, 1H, CH_{Ar}), 7.87–7.79 (m, 2H, CH_{Ar}), 2.88 (t, ^3J = 7.7 Hz, 2H, CH_2), 1.79 (quint, ^3J = 7.6 Hz, 2H, CH_2), 1.52–1.43 (m, 2H, CH_2), 0.99 (t, ^3J = 7.4 Hz, 3H, CH_3). Further analysis can be found at https://dx.doi.org/10.14272/reaction/SA-FUHFF-UHFFFADPSC-AHSWENVYXH-UHFFFADPSC-NUHFF-NUHFF-NUHFF-ZZZ.

Additional information on the chemical synthesis is available via Chemotion repository: https://doi.org/10.14272/reaction/SA-FUHFF-UHFFFADPSC-LPPUONCCTY-UHFFFADPSC-NUHFF-NUHFF-NUHFF-ZZZ

Additional information on the analysis of the target compound is available via Chemotion repository:
https://doi.org/10.14272/LGMVEBQKPYIMMI-UHFFFAOYSA-N.3
https://doi.org/10.14272/AHSWENVYXHLEHF-UHFFFAOYSA-N.2

1-phenyl-4-(trifluoromethyl)imidazo[1,2-a]quinoxaline (**S9**)

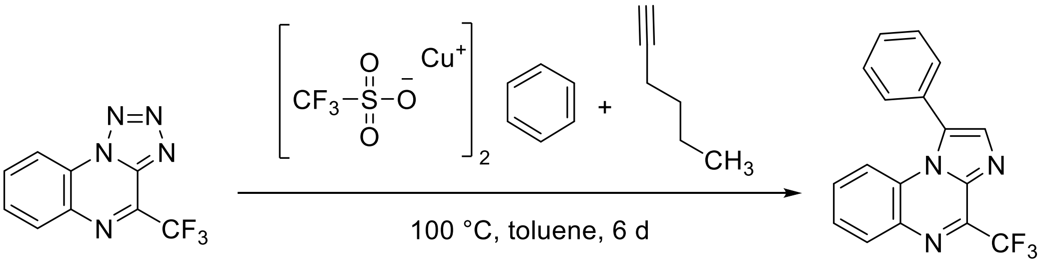

Name {P1|**S9**}: 1-phenyl-4-(trifluoromethyl)imidazo[1,2-a]quinoxaline; Formula: $C_{17}H_{10}F_3N_3$; Molecular Mass: 313.2766; Exact Mass: 313.0827; Smiles: FC(c1nc2ccccc2n2c1ncc2c1ccccc1)(F)F; InChIKey: VQYHDOFNDCKODK-UHFFFAOYSA-N

The starting material 4-(trifluoromethyl)tetrazolo[1,5-a]quinoxaline (49.0 mg, 205 µmol, 1.00 equiv) and the catalyst benzene;copper(1+);trifluoromethanesulfonate (10.5 mg, 20.9 µmol, 0.102 equiv) were dissolved in 1 mL of dry toluene under nitrogen, followed by ethynylbenzene (42.7 mg, 45.9 µL, 418 µmol, 2.04 equiv). The reaction mixture was stirred at 100 °C for 6 days. Then water and EtOAc were added, the organic phase was separated and the aqueous phase was extracted 3× with EtOAc. The combined organic phases were dried over Na_2SO_4, filtered and the solvent was removed under reduced pressure. The crude product was purified via column chromatography (dryload on Celite, *c*Hex -> *c*Hex/EtOAc 10:1) and 1-phenyl-4-(trifluoromethyl)imidazo[1,2-a]quinoxaline (12.0 mg, 38.3 µmol, 19% yield) was obtained as a yellow-brown solid.

R_f = 0.37 (cyclohexane/ethyl acetate 4:1). ^{1}H NMR (400 MHz, $CDCl_3$, ppm) δ = 8.22 (dd, ^{3}J = 8.1 Hz, ^{4}J = 1.5 Hz, 1H, CH_{ar}), 7.81 (s, 1H, C$H_{imidazole}$), 7.62–7.56 (m, 7H, CH_{ar}), 7.42 (ddd, ^{3}J = 8.8 Hz, ^{3}J = 7.1 Hz, ^{4}J = 1.8 Hz, 1H, CH_{ar}); ^{13}C NMR (100 MHz, $CDCl_3$ [77.0 ppm], ppm) δ = 140.6 (d, ^{2}J = 36.6 Hz, 1C, CCF_3), 135.8 (1C, $CH_{imidazole}$), 135.5 (1C, C_q), 134.8 (1C, C_q), 131.8 (1C, CH), 131.0 (1C, C_q), 130.2 (2C, CH), 130.1 (CH), 130.0 (CH), 129.7 (1C, C_q), 129.2 (2C, CH), 126.9 (CH), 120.5 (q, ^{1}J = 275.9 Hz, 1C, CF_3), 116.2 (1C, CH). Missing 1C (1C, C_q), probably due to overlapping with the signal at 129.2; MS (EI, 70 eV, 80 °C), m/z (%): 314 $[M+1]^+$ (24), 313 $[M]^+$ (100), 312 (28), 292 (24), 102 (23), 69 (17). HRMS (EI, $C_{17}H_{10}N_3F_3$): calcd 313.0821, found 313.0819; IR (ATR, ṽ) = 3055 (vw), 2921 (w), 2859 (vw), 1553 (w), 1462 (w), 1446 (w), 1395 (m), 1370 (w), 1312 (w), 1264 (w), 1228 (m), 1187 (s), 1180 (s), 1164 (m), 1145 (vs), 1137 (vs), 1088 (m), 1054 (s), 1030 (m), 969 (w), 921 (s), 885 (w), 860 (w), 849 (w), 758 (vs), 734 (vs), 701 (vs), 690 (s), 656 (m), 615 (w), 591 (m), 572 (m), 534 (w), 526 (w), 501 (w), 480 (m), 455 (m) cm^{-1}.

Bis(tetrazolo)[1,5-*a*:5',1'-*c*]quinoxaline (**24**)

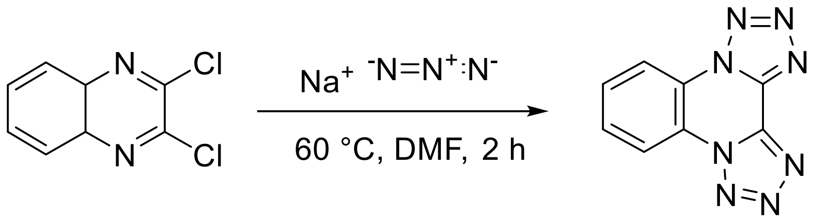

Name {P1|**24**}: Bis(tetrazolo)[1,5-*a*:5',1'-*c*]quinoxaline; Formula: $C_8H_4N_8$; Molecular Mass: 212.1710; Exact Mass: 212.0559; Smiles: c1ccc2c(c1)n1nnnc1c1n2nnn1; InChIKey: CXZSDEGQLBZWEC-UHFFFAOYSA-N

Sodium azide (0.19 g, 3.00 mmol, 3.0 equiv) was added to 0.20 g of 2,3-dichloroquinoxaline (1.00 mmol, 1.0 equiv) in 5 mL of DMF and stirred at 60 °C for 2 h. Distilled water was added, the organic phase was separated and the aqueous phase was extracted 3x with EtOAc. The combined organic phases were dried over Na_2SO_4, filtered and the solvent was removed under reduced pressure. A small portion of EtOAc was added to transfer the solid crude product into a funnel and the product was filtered and washed with water. The product was obtained in form of a colorless solid (0.20 g, 0.94 mmol, 93% yield). Note: This reaction was repeated with a yield of 98%. Hereby, the reaction mixture was cooled to 25 °C after 2 h of stirring at 60 °C, then water was added; the precipitated product was collected via filtration and washed 3x with water.

R_f = 0.26 (cyclohexane/ethyl acetate 4:1). ^{1}H NMR (400 MHz, DMSO-d_6, ppm) δ = 8.79–8.74 (m, 2H, CH_{ar}), 8.09–8.04 (m, 2H, CH_{ar}); ^{13}C NMR (100 MHz, DMSO-d_6, ppm) δ = 140.4 (2C, N*C*N), 130.9 (2C, C_{ar}), 122.7 (2C, C_{ar}), 117.7 (2C, CH_{ar}); MS (EI, m/z, 70 eV, 170 °C): 212 (6) $[M]^+$, 156 (33), 104 (100), 77 (18), 52 (11). HRMS (EI, $C_8H_4N_8$): Calcd 212.0559, Found 212.0560; IR (ATR, ṽ) = 3077 (w), 3057 (w), 1645 (w), 1581 (m), 1509 (m), 1482 (vs), 1460 (w), 1404 (m), 1392 (m), 1353 (w), 1326 (w), 1289 (s), 1262 (w), 1198 (s), 1173 (w), 1145 (w), 1129 (s), 1111 (m), 1092 (w), 1018 (w), 987 (w), 972 (s), 778 (vs), 722 (m), 707 (m), 666 (s), 463 (vs), 458 (s), 438 (m) cm^{-1}.

Additional information on the chemical synthesis is available via Chemotion repository: https://doi.org/10.14272/reaction/SA-FUHFF-UHFFFADPSC-CXZSDEGQLB-UHFFFADPSC-NUHFF-NUHFF-NUHFF-ZZZ

Additional information on the analysis of the target compound is available via Chemotion repository:
https://doi.org/10.14272/CXZSDEGQLBZWEC-UHFFFAOYSA-N.1

The synthesis of this compound has been previously described and the ^{1}H NMR data corresponds with the literature [31]**.**

1-Phenyl-4-(4-phenyl-1*H*-1,2,3-triazol-1-yl)imidazo[1,2-a]quinoxaline (**25a**), **1-phenylimidazo[1,2-*a*]quinoxalin-4-amine** (**S5a**), **3-(4-phenyl-1*H*-1,2,3-triazol-1-yl)quinoxalin-2-amine** (**S6a**)

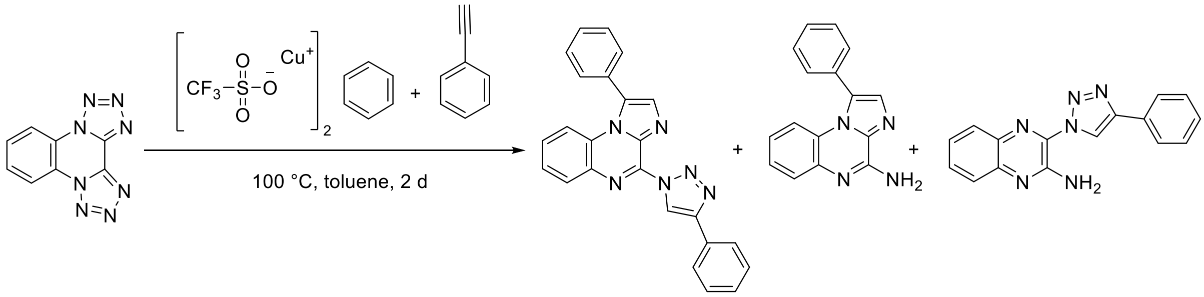

Name {P1|**25a**}: 1-phenyl-4-(4-phenyl-1*H*-1,2,3-triazol-1-yl)imidazo[1,2-*a*]quinoxaline; Formula: $C_{24}H_{16}N_6$; Molecular Mass: 388.4240; Exact Mass: 388.1436; Smiles: c1ccc(cc1)c1nnn(c1)c1nc2ccccc2n2c1ncc2c1ccccc1; InChIKey: DHVXGDPBGBTTOC-UHFFFAOYSA-N

Name {P2|**S5a**}: 1-phenylimidazo[1,2-*a*]quinoxalin-4-amine; Formula: $C_{16}H_{12}N_4$; Molecular Mass: 260.2933; Exact Mass: 260.1062; Smiles: Nc1nc2ccccc2n2c1ncc2c1ccccc1; InChIKey: LDKDBUVLGOHXSC-UHFFFAOYSA-N

Name {P3|**S6a**}: 3-(4-phenyl-1*H*-1,2,3-triazol-1-yl)quinoxalin-2-amine; Formula: $C_{16}H_{12}N_6$; Molecular Mass: 288.3067; Exact Mass: 288.1123; Smiles: Nc1nc2ccccc2nc1n1nnc(c1)c1ccccc1; InChIKey: XAKPOUMBAWIAJL-UHFFFAOYSA-N

The starting material bis(tetrazolo)[1,5-*a*:5',1'-*c*]quinoxaline and the catalyst benzene;copper(1+);trifluoromethanesulfonate (29.0 mg, 57.6 µmol, 0.121 equiv) were dissolved in 2 mL of dry toluene under argon, followed by ethynylbenzene (120 mg, 129 µL, 1.18 mmol, 2.48 equiv). The yellow-brown reaction mixture was stirred at 100 °C for 2 days. Then water and EtOAc were added, the organic phase was separated and the aqueous phase was extracted 3x with EtOAc. The combined organic phases were dried over Na_2SO_4, filtered and the solvent was removed under reduced pressure. The crude mixture was separated multiple times via column chromatography (*c*Hex/EtOAc+2% Et_3N, DCM/EtOAc+2% Et_3N). Moreover, part of the product was isolated via filtration; impure fractions were combined and the solvent was evaporated under reduced pressure until pure product precipitated. The precipitate was washed 2x with 2-3 mL of EtOAc. The desired product 1-phenyl-4-(4-phenyl-1*H*-

1,2,3-triazol-1-yl)imidazo[1,2-a]quinoxaline (37.0 mg, 95.3 µmol, 20% yield) was obtained as a beige solid; 1-phenylimidazo[1,2-*a*]quinoxalin-4-amine (10.0 mg, 38.4 µmol, 8% yield) and 3-(4-phenyl-1*H*-1,2,3-triazol-1-yl)quinoxalin-2-amine (4.00 mg, 13.9 µmol, 3% yield) were obtained both as yellow solids.

R_f = 0.41 (cyclohexane/ethyl acetate 2:1). ^{1}H NMR (400 MHz, $CDCl_3$, ppm) δ = 9.67 (s, 1H, NC$H_{triazole}$), 8.27 (dd, ^3J = 8.2 Hz, ^4J = 1.6 Hz, 1H, CH_{ar}), 8.08–8.06 (m, 2H, CH_{ar}), 7.81 (s, 1H, NC$H_{imidazole}$), 7.62–7.61 (m, 5H, CH_{ar}), 7.59–7.56 (m, 2H, CH_{ar}), 7.52–7.48 (m, 2H, CH_{ar}), 7.42–7.34 (m, 2H, CH_{ar}); ^{13}C NMR (100 MHz, $CDCl_3$ [77.0 ppm], ppm) δ = 147.8 (1C, C_q), 139.1 (1C, C_q), 135.2 (1C, CH$_{imidazole}$), 134.8 (1C, C_q), 132.6 (1C, C_q), 132.0 (1C, C_q), 131.0 (1C, CH$_{ar}$), 130.3 (2C, CH$_{ar}$), 130.1 (1C, C_q), 130.0 (1C, CH$_{ar}$), 129.6 (1C, C_q), 129.2 (2C, CH$_{ar}$), 128.8 (2C, CH$_{ar}$), 128.5 (1C, CH$_{ar}$), 128.5 (1C, CH$_{ar}$), 128.3 (1C, C_q), 127.1 (1C, CH$_{ar}$), 126.2 (2C, CH$_{ar}$), 121.6 (1C, CH$_{triazole}$), 116.1 (1C, CH$_{ar}$); MS (FAB, 3-NBA), m/z (%): 390 $[M+1]^+$ (10), 389 $[M]^+$ (29), 361 (32), 360 (16), 307 (15), 155 (36), 154 (100), 136 (80), 107 (35), 97 (41), 95 (42), 91 (51). HRMS ($C_{24}H_{17}N_6$): calcd 389.1509, found 389.1511; IR (ATR, ṽ) = 3140 (w), 3101 (w), 3072 (w), 3059 (w), 3041 (w), 2956 (w), 2921 (w), 2851 (w), 1543 (w), 1504 (s), 1471 (vs), 1459 (vs), 1450 (s), 1417 (s), 1389 (m), 1364 (w), 1343 (m), 1308 (w), 1284 (w), 1253 (w), 1242 (m), 1197 (vs), 1166 (w), 1160 (w), 1131 (w), 1073 (s), 1028 (w), 1005 (vs), 968 (w), 956 (m), 917 (w), 906 (m), 894 (w), 834 (m), 760 (vs), 727 (w) cm^{-1}.

^{1}H NMR (400 MHz, $CDCl_3$, ppm) δ = 7.71 (d, ^3J = 8.2 Hz, 1H, CH_{ar}), 7.56 (s, 5H, CH_{ar}), 7.52 (s, 1H, C$H_{imidazole}$), 7.40–7.35 (m, 2H, CH_{ar}), 7.04–7.00 (m, 1H, CH_{ar}), 6.17 (bs, 2H, NH_2). Spectrum contains residual EtOAc at 4.13 ppm, 2.05 ppm and 1.27 ppm; ^{13}C NMR (100 MHz, $CDCl_3$ [77.0 ppm], ppm) δ = 148.2 (1C, C_q), 135.7 (1C, C_q), 133.2 (1C, CH$_{imidazole}$), 132.9, 131.6 (1C, C_q), 130.3 (2C, CH$_{ar}$), 130.1 (1C, C_q), 129.6 (1C, CH$_{ar}$), 129.0 (2C, CH$_{ar}$), 126.6 (1C, CH$_{ar}$), 126.0 (1C, CH$_{ar}$), 123.5 (1C, CH$_{ar}$), 116.1 (1C, CH$_{ar}$). Missing 13C (1C, C_q) due to low intesity/overlapping with other signals. MS (EI, 70 eV, 130 °C), m/z (%): 261 [M+1]+ (19), 260 [M]+ (100), 259 (42), 90 (17). HRMS (EI, $C_{16}H_{12}N_4$): calcd 260.1056, found 260.1058. IR (ATR, ṽ) = 3350 (w), 3286 (w), 3248 (w), 3165 (w), 3146 (w), 3060 (w), 2953 (w), 2919 (w), 2850 (w), 1638 (s), 1608 (m), 1537 (w), 1520 (vs), 1479 (m), 1469 (m), 1448 (m), 1422 (s), 1373 (w), 1334 (w), 1302 (w), 1273 (w), 1242 (w), 1170 (w), 1133 (w), 1103 (w), 1079 (w), 1026 (w), 1001 (w), 979 (w), 955 (w), 919 (w), 877 (w), 856 (m), 768 (m), 755 (vs), 724 (m), 701 (vs), 622 (m), 585 (vs), 569 (s), 541 (vs), 518 (vs), 477 (vs), 459 (s) cm^{-1}.

^{1}H NMR (400 MHz, $CDCl_3$, ppm) δ = 9.09 (s, 1H, C$H_{triazole}$), 8.02–8.00 (m, 2H, CH_{ar}), 7.95 (d, ^3J = 9.8 Hz, 1H, CH_{ar}), 7.80 (d, ^3J = 8.3 Hz, 1H, CH_{ar}), 7.74–7.70 (m, 1H, CH_{ar}), 7.61–7.43 (m, 4H, CH_{ar}). Missing 2H (2H, NH_2) due to H-D exchange in $CDCl_3$; ^{13}C NMR (100 MHz, $CDCl_3$ [77.0 ppm], ppm) δ = 131.8 (1H, CH_{ar}), 129.2 (1C, CH_{ar}), 129.1 (2C, CH_{ar}), 128.5 (1C, CH_{ar}), 126.8 (1C, CH_{ar}), 126.2 (2C, CH_{ar}), 123.5 (1C, CH_{ar}), 118.4 (1C, C$H_{triazole}$). Missing 6C (6C, C_q) due to low amount of compound and resulting low intensity; 13C NMR (101 MHz, CDCl3) δ 126.13, 128.30, 123.61, 131.91, 126.86, 129.02, 129.02 1H NMR (400 MHz, CDCl3) δ 7.92, 7.88, 7.75, 7.65, 7.50, 7.44, 7.37; MS (EI, 70 eV, 160 °C), m/z (%): 288 $[M]^+$ (4), 261 (21), 260 (100), 259 (34), 144 (98), 117 (36), 102 (16), 90 (37). HRMS (EI, $C_{16}H_{12}N_6$): calcd 288.1118, found 288.1116; IR (ATR, ṽ) = 3398 (w), 3289 (w), 3173 (w), 3129 (w), 3060 (w), 2956 (w), 2922 (w), 2851 (w), 1667 (w), 1640 (s), 1605 (w), 1582 (w), 1557 (m), 1496 (w), 1480 (m), 1460 (vs), 1448 (s), 1409 (m), 1354 (m), 1323 (w), 1305 (w), 1285 (w), 1239 (s), 1211 (m), 1180 (m), 1154 (w), 1133 (w), 1072 (w), 1030 (s), 1020 (s), 993 (vs),

962 (m), 914 (m), 863 (w), 809 (m), 758 (vs), 725 (s), 691 (vs), 613 (s), 594 (s), 511 (m), 456 (vs), 402 (s) cm^{-1}.

Additional information on the chemical synthesis is available via Chemotion repository: https://doi.org/10.14272/reaction/SA-FUHFF-UHFFFADPSC-XIPNBMHBNZ-UHFFFADPSC-NUHFF-NUHFF-NUHFF-ZZZ

Additional information on the analysis of the target compound is available via Chemotion repository:
https://doi.org/10.14272/DHVXGDPBGBTTOC-UHFFFAOYSA-N.1
https://doi.org/10.14272/LDKDBUVLGOHXSC-UHFFFAOYSA-N.1
https://doi.org/10.14272/XAKPOUMBAWIAJL-UHFFFAOYSA-N.1

The synthesis of side product 1-phenylimidazo[1,2-*a*]quinoxalin-4-amine has been previously reported in literature [32].

1-Butyl-4-(4-butyl-1*H*-1,2,3-triazol-1-yl)imidazo[1,2-a]quinoxaline (25b), 1-butylimidazo[1,2-*a*]quinoxalin-4-amine (S5b), 2,3-bis(4-butyl-1*H*-1,2,3-triazol-1-yl)quinoxaline (S7b), 3,10-dibutyldiimidazo[1,2-*a*:2',1'-*c*]quinoxaline (S8b), 3-(4-butyl-1*H*-1,2,3-triazol-1-yl)quinoxalin-2-amine (S6b)

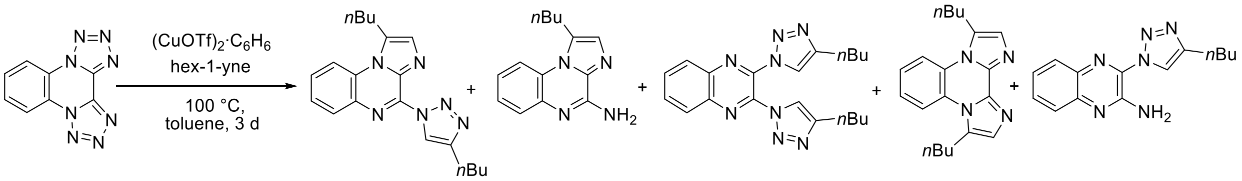

Name {P1|**25b**}: 1-butyl-4-(4-butyl-1*H*-1,2,3-triazol-1-yl)imidazo[1,2-a]quinoxaline; Formula: $C_{20}H_{24}N_6$; Molecular Mass: 348.4448; Exact Mass: 348.2062; Smiles: CCCCc1nnn(c1)c1nc2ccccc2n2c1ncc2CCCC; InChIKey: OGPHMCIIGIGFPL-UHFFFAOYSA-N

Name {P2|**S5b**}: 1-butylimidazo[1,2-*a*]quinoxalin-4-amine; Formula: $C_{14}H_{16}N_4$; Molecular Mass: 240.3036; Exact Mass: 240.1375; Smiles: CCCCc1cnc2n1c1ccccc1nc2N; InChIKey: IMOCGYGQTYCGDY-UHFFFAOYSA-N

Name {P3|**S7b**}: 2,3-bis(4-butyl-1*H*-1,2,3-triazol-1-yl)quinoxaline; Formula: $C_{20}H_{24}N_8$; Molecular Mass: 376.4582; Exact Mass: 376.2124; Smiles: CCCCc1nnn(c1)c1nc2ccccc2nc1n1nnc(c1)CCCC; InChIKey: BZUADUCGXDAGNQ-UHFFFAOYSA-N

Name {P4|**S8b**}: 3,10-dibutyldiimidazo[1,2-*a*:2',1'-*c*]quinoxaline; Formula: $C_{20}H_{24}N_4$; Molecular Mass: 320.4314; Exact Mass: 320.2001; Smiles: CCCCc1cnc2n1c1ccccc1n1c2ncc1CCCC; InChIKey: NSBRVZPRPYVSJO-UHFFFAOYSA-N

Name {P5|**S6b**}: 3-(4-butyl-1*H*-1,2,3-triazol-1-yl)quinoxalin-2-amine; Formula: $C_{14}H_{16}N_6$; Molecular Mass: 268.3170; Exact Mass: 268.1436; Smiles: CCCCc1nnn(c1)c1nc2ccccc2nc1N; InChIKey: HPWSELBHSNTVDQ-UHFFFAOYSA-N

The starting material bis(tetrazolo)[1,5-*a*:5',1'-*c*]quinoxaline (300 mg, 1.41 mmol, 1.00 equiv) and the catalyst benzene;copper(1+);trifluoromethanesulfonate (68.9 mg, 137 µmol, 0.0968 equiv) were dissolved in 5 mL of dry toluene under argon, followed by hex-1-yne (232 mg, 325 µL, 2.83 mmol, 2.00 equiv). The reaction mixture was stirred at 100 °C for 3 days; then 0.1 mL of hexyne were added again and the reaction was stirred at 100 °C for another 3 hours. Subsequently water and ethyl acetate were added, the organic phase was separated and the aqueous phase was extracted 3× with DCM. The combined organic phases were dried over Na_2SO_4, filtered and the solvent was removed under reduced pressure. The crude mixture was separated twice via column chromatography (dryload on Celite, DCM/EtOAc+2%Et_3N); a mixed fraction was further purified via HPLC (acetonitrile/water). 1-butyl-4-(4-butyl-1*H*-1,2,3-triazol-1-yl)imidazo[1,2-a]quinoxaline (107 mg, 307 µmol, 22% yield) was isolated as a white to light brown solid. 1-butylimidazo[1,2-*a*]quinoxalin-4-amine (28.0 mg, 117 µmol, 8% yield) was obtained as a light brown solid, 2,3-bis(4-butyl-1*H*-1,2,3-triazol-1-yl)quinoxaline (13.0 mg, 34.5 µmol, 2% yield) was obtained as a yellow solid and 3-(4-butyl-1*H*-1,2,3-triazol-1-yl)quinoxalin-2-amine (13.0 mg, 48.5 µmol, 3% yield) was obtained as a white to light yellow solid. Moreover, impure traces of 3,10-dibutyldiimidazo[1,2-*a*:2',1'-*c*]quinoxaline (12.0 mg, 37.4 µmol, 3% yield) were presumably obtained. Note: This reaction was repeated with a reaction time of 3.5 h and a yield of 20% for the main product 1-butyl-4-(4-butyl-1*H*-1,2,3-triazol-1-yl)imidazo[1,2-a]quinoxaline (no other fractions isolated).

R_f = 0.24 (cyclohexane/ethyl acetate 2:1). ^{1}H NMR (400 MHz, $CDCl_3$, ppm) δ = 9.07 (s, 1H, $CH_{triazole}$), 8.31–8.26 (m, 2H, CH_{ar}), 7.72–7.64 (m, 3H, CH_{ar}+$CH_{imidazole}$), 3.38 (t, ^3J = 7.6 Hz, 2H, C_qCH_2), 2.91 (t, ^3J = 7.7 Hz, 2H, C_qCH_2), 1.95 (p, ^3J = 7.5 Hz, 2H, CH_2CH_2), 1.80 (p, ^3J = 7.5 Hz, 2H, CH_2CH_2), 1.61 (h, ^3J = 7.3 Hz, 2H, CH_2CH_3), 1.47 (h, ^3J = 7.4 Hz, 2H, CH_2CH_3), 1.07 (t, ^3J = 7.4 Hz, 3H, CH_3), 0.98 (t, ^3J = 7.4 Hz, 3H, CH_3); ^{13}C NMR (100 MHz, $CDCl_3$ [77.0 ppm], ppm) δ = 148.4 (1C, $C_{triazole}$), 139.3 (1C, C_q), 134.9 (1C, C_q), 133.2 (1C, C_q), 133.1 (1C, $CH_{imidazole}$), 132.6 (1C, C_q), 131.1 (1C, CH_{ar}), 129.1 (1C, C_q), 128.6 (1C, CH_{ar}), 126.9 (1C, CH_{ar}), 122.7 (1C, $CH_{triazole}$), 115.5 (1C, CH_{ar}), 31.5 (1C, CH_2CH_2), 29.9 (1C, CH_2CH_2), 27.9 (1C, C_qCH_2), 25.4 (1C, C_qCH_2), 22.4 (1C, CH_2CH_3), 22.3 (1C, CH_2CH_3), 13.8 (2C, CH_3). MS (EI, 70 eV, 170 °C), m/z (%): 348 $[M]^+$ (2), 320 (40), 319 (20), 305 (38), 293 (23), 292 (82), 291 (100), 279 (32), 278 (82), 277 (100), 265 (26), 240 (28), 225 (44), 224 (41), 197 (51), 196 (54), 183 (18), 182 (44), 181 (30), 129 (28). HRMS (EI, $C_{20}H_{24}N_6$): calcd 348.2057, found 348.2057. IR (ATR, ṽ) = 3153 (w), 2951 (m), 2922 (s), 2857 (s), 1534 (w), 1492 (vs), 1462 (vs), 1438 (m), 1417 (vs), 1398 (m), 1373 (m), 1361 (m), 1341 (m), 1313 (m), 1279 (w), 1258 (w), 1232 (s), 1207 (m), 1196 (m), 1174 (s), 1157 (s), 1126 (m), 1103 (m), 1034 (vs), 977 (s), 925 (m), 911 (m), 866 (w), 844 (s), 834 (s), 810 (m), 761 (vs), 742 (vs), 660 (m), 643 (m), 635 (vs), 615 (w), 588 (m), 476 (m), 450 (s), 405 (w), 398 (w) cm^{-1}. Crystals suitable for Single Crystal X-Ray Diffraction Analysis obtained via slow evaporation of a solution in MeOH under ambient conditions. Crystal Data for $C_{20}H_{24}N_6$ (M =348.45 g/mol): triclinic, space group P-1 (no. 2), a = 7.2494(4) Å, b = 9.0176(5) Å, c = 14.3850(8) Å, α = 75.401(4)°, β = 85.805(4)°, γ = 79.431(4)°, V = 894.22(9) Å3, Z = 2, T = 150.0 K, μ(GaKα) = 0.410 mm-1, Dcalc = 1.294 g/cm^3, 10270 reflections measured (5.526° ≤ 2Θ ≤ 124.996°), 4123 unique (Rint = 0.0447, Rsigma = 0.0456) which were used in all calculations. The final R1 was 0.0944 (I > 2σ(I)) and wR2 was 0.3021 (all data). UV/VIS (acetonitrile), λ = 332 (1.66), 262 (2.24), 234 (2.13), 226 (1.89) nm.

^{1}H NMR (400 MHz, $CDCl_3$, ppm) δ = 8.07 (d, ^3J = 7.5 Hz, 1H, CH_{ar}), 7.75 (d, ^3J = 8.8 Hz, 1H, CH_{ar}), 7.47 (t, ^3J = 7.2 Hz, 1H, CH_{ar}), 7.39–7.34 (m, 2H, CH_{ar}+$CH_{imidazole}$), 6.19, (bs, 2H, NH_2), 3.26 (t, ^3J = 7.6 Hz, 2H, C_qCH_2), 1.89 (p, ^3J = 7.5 Hz, 2H, CH_2CH_2), 1.58 (h, ^3J = 7.4 Hz, 2H, CH_2CH_3), 1.05 (t, ^3J = 7.3 Hz, 3H, CH_3); ^{13}C NMR (100 MHz, $CDCl_3$ [77.0 ppm], ppm) δ = 148.3, 136.1, 132.6, 132.5, 131.1 (1C, $CH_{imidazole}$), 126.4 (1C, CH_{ar}), 126.3 (1C, CH_{ar}), 123.7 (1C, CH_{ar}), 115.5 (1C, CH_{ar}), 30.0 (1C, CH_2CH_2), 27.6 (1C, CH_qCH_2), 22.5 (1C, CH_2CH_3), 13.9 (1C, CH_3). Missing 1C (1C, C_q) due to low intensity. MS (ESI), m/z (%): 282 (24), 241 $[M+1]^+$ (100), 219 (29), 187 (13). HRMS (ESI, $C_{17}H_{14}N_4$): calcd 241.1453, found 241.1444. IR (ATR, ṽ) = 3305 (m), 3140 (m), 2972 (w), 2951 (m), 2928 (m), 2860 (w), 1646 (vs), 1606 (m), 1537 (s), 1519 (vs), 1469 (s), 1451 (s), 1432 (vs), 1377 (s), 1333 (m), 1313 (m), 1272 (s), 1255 (s), 1197 (m), 1159 (m), 1129 (m), 1099 (m), 996 (m), 977 (w), 932 (m), 874 (m), 851 (s), 819 (w), 749 (vs), 717 (s), 687 (m), 670 (s), 653 (m), 606 (vs), 557 (vs), 526 (s), 472 (vs), 453 (s), 421 (m), 388 (m) cm^{-1}.

^{1}H NMR (400 MHz, $CDCl_3$, ppm) δ = 8.22–8.20 (m, 2H, CH_{ar}), 8.05 (s, 2H, $CH_{triazole}$), 7.98–7.95 (m, 2H, CH_{ar}), 2.83 (t, ^3J = 7.5 Hz, 4H, C_qCH_2), 1.75 (p, ^3J = 7.5 Hz, 4H, CH_2CH_2), 1.45 (h, ^3J = 7.3 Hz, 4H, CH_2CH_3), 0.98 (t, ^3J = 7.3 Hz, 6H, CH_3); ^{13}C NMR (100 MHZ, $CDCl_3$ [77.0 ppm], ppm) δ = 148.6 (2C, $C_{triazole}$), 140.4 (2C, C_q), 138.5 (2C, C_q) , 132.4 (2C, CH_{ar}), 129.1 (2C, CH_{ar}), 121.3 (2C, $CH_{triazole}$), 31.2 (2C, CH_2CH_2), 25.2 (2C, C_qCH_2), 22.3 (2C, CH_2CH_3), 13.8 (2C, CH_3); 13C NMR (101 MHz, CDCl3) δ 129.02, 121.08, 132.27, 25.46, 31.24, 22.22, 13.92 1H NMR (400 MHz, CDCl3) δ 8.13, 7.97, 7.89, 2.75, 1.67, 1.37, 0.89. MS (ESI), m/z (%): 377 $[M+1]^+$ (64), 283 (19), 282 (100), 254 (15), 163 (25), 156 (15), 155 (20), 145 (27), 120 (46), 100 (19). HRMS ($C_{20}H_{25}N_8$): calcd 377.2197, found 377.2193. IR (ATR, ṽ) = 3169 (vw), 3122 (vw), 3078 (w), 2956 (m), 2927 (s), 2860 (m), 1707 (vw), 1656 (vw), 1609 (w), 1561 (w), 1526 (w), 1499 (s), 1468 (s), 1421 (s), 1360 (m), 1351 (m), 1340 (m), 1312 (m), 1241 (s), 1222 (s), 1210 (s), 1159 (s), 1140 (m), 1099 (w), 1045 (vs), 1034 (vs), 1020 (m), 999 (s), 959 (vs), 933 (w), 897 (w), 881 (w), 840 (m), 829 (w), 806 (m), 792 (w), 772 (vs), 755 (s), 731 (m), 705 (w), 691 (w), 674 (w), 654 (m), 619 (w), 606 (w), 591 (w), 517 (m), 503 (w), 469 (w), 395 (w), 378 (w) cm^{-1}.

^{1}H NMR (400 MHz, $CDCl_3$, ppm) δ = 8.60 (s, 1H, $CH_{triazole}$), 7.86 (dd, ^3J = 8.3 Hz, ^4J = 1.6 Hz, 1H, CH_{ar}), 7.72 (dd, ^3J = 8.4 Hz, ^4J = 1.6 Hz, 1H, CH_{ar}), 7.66–7.62 (m, 1H, CH_{ar}), 7.50–7.46 (m, 1H, CH_{ar}), 7.01 (bs, 2H, NH_2), 2.87 (t, ^3J = 7.7 Hz, 2H, C_qCH_2), 1.79 (p, ^3J = 7.5 Hz, 2H, CH_2CH_2), 1.47 (h, ^3J = 7.4 Hz, 2H, CH_2CH_3), 0.99 (t, ^3J = 7.4 Hz, 3H, CH_3); ^{13}C NMR (100 MHz, $CDCl_3$ [77.0 ppm], ppm) δ = 148.3 (1C, C_q), 145.3 (1C, C_q), 140.7 (1C, C_q), 134.7 (1C, C_q), 132.8 (1C, C_q), 130.7 (1C, CH_{ar}), 128.2 (1C, CH_{ar}), 125.9 (1C, CH_{ar}), 125.2 (1C, CH_{ar}), 120.0 (1C, $CH_{triazole}$), 31.2 (1C, CH_2CH_2), 25.2 (1C, C_qCH_2), 22.3 (1C, CH_2CH_3), 13.8 (1C, CH_3). MS (EI, 70 eV, 110 °C), m/z (%): 268 $[M]^+$ (6), 212 (18), 211 (23), 198 (19), 197 (81), 145 (15), 144 (100), 118 (16), 117 (27), 90 (28). HRMS (EI, $C_{14}H_{16}N_6$): calcd 268.1431, found 268.1431. IR (ATR, ṽ) = 3393 (m), 3288 (w), 3132 (m), 2951 (m), 2924 (m), 2867 (w), 2854 (w), 1636 (vs), 1605 (m), 1582 (w), 1558 (s), 1496 (m), 1479 (s), 1459 (vs), 1409 (s), 1375 (w), 1358 (m), 1332 (w), 1296 (w), 1248 (m), 1220 (vs), 1164 (m), 1143 (m), 1128 (m), 1033 (vs), 1017 (s), 983 (vs), 948 (m), 912 (m), 863 (w), 810 (s), 785 (w), 759 (vs), 728 (s), 681 (m), 635 (m), 613 (s), 592 (s), 568 (w), 535 (m), 465 (vs), 375 (s) cm^{-1}.

Additional information on the chemical synthesis is available via Chemotion repository: https://doi.org/10.14272/reaction/SA-FUHFF-UHFFFADPSC-WFIIEKHKQM-UHFFFADPSC-NUHFF-NUHFF-NUHFF-ZZZ

Additional information on the analysis of the target compound is available via Chemotion repository:
https://doi.org/10.14272/OGPHMCIIGIGFPL-UHFFFAOYSA-N.1
https://doi.org/10.14272/IMOCGYGQTYCGDY-UHFFFAOYSA-N.1
https://doi.org/10.14272/BZUADUCGXDAGNQ-UHFFFAOYSA-N.1
https://doi.org/10.14272/HPWSELBHSNTVDQ-UHFFFAOYSA-N.1

2-(4-Butyl-1*H*-1,2,3-triazol-1-yl)quinoxalinetricarbonylrhenium(I)-bromide (**27a**)

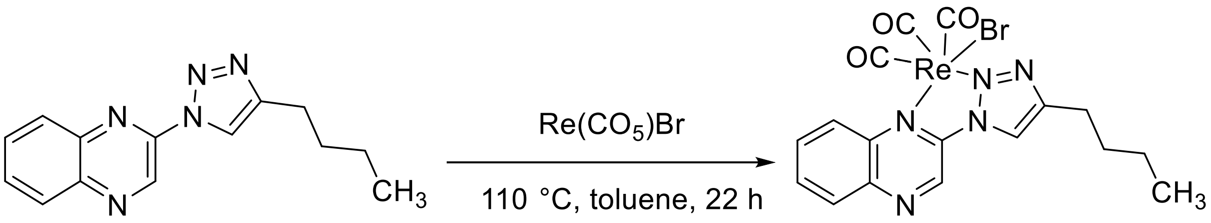

Name {P1|**27a**}: 2-(4-butyl-1*H*-1,2,3-triazol-1-yl)quinoxalinetricarbonylrhenium(I)-bromide; Formula: $C_{17}H_{15}BrN_5O_3Re$; Molecular Mass: 603.4437; Exact Mass: 602.9916; Smiles: [O]#C[Re](C#[O])(C#[O])Br.CCCCc1nnn(c1)c1cnc2c(n1)cccc2; InChIKey: CCAUEKYEPREEHO-UHFFFAOYSA-M

The ligand 2-(4-butyl-1*H*-1,2,3-triazol-1-yl)quinoxaline (49.8 mg, 197 µmol, 1.00 equiv) was dissolved in anhydrous toluene (3.00 mL) and heated to 110 °C under argon. Then bromorhenium;carbon monoxide (80.0 mg, 197 µmol, 1.00 equiv), and another 0.5 mL of dry toluene were added. The solution was stirred at 110 °C under argon for 6 h and subsequently, the red mixture was cooled to 25 °C and stirred for 16 h; then the solvent was evaporated under reduced pressure. The obtained crude product was purified via flash chromatography on silica gel using *c*Hex to EtOAc and 2-(4-butyl-1*H*-1,2,3-triazol-1-yl)quinoxalinetricarbonylrhenium(I)-bromide (87.5 mg, 145 µmol) was obtained as a red solid in 74% yield. Note: This reaction was repeated with a yield of 87%.

R_f = 0.1 (cyclohexane/ethyl acetate 2:1). ^{1}H NMR (400 MHz, Chloroform-d [7.27 ppm], ppm) δ = 9.37 (s, 1H, C*H*), 8.75 (d, *J* = 8.8 Hz, 1H, CH_{Ar}), 8.48 (s, 1H, C*H*), 8.35 (dd, *J* = 1.2 Hz, *J* = 8.3 Hz, 1H, CH_{Ar}), 8.20–8.16 (m, 1H, CH_{Ar}), 8.09–8.05 (m, 1H, CH_{Ar}), 2.96 (dt, *J* = 4.0 Hz, *J* = 7.6 Hz, 2H, CH_2), 1.85–1.81 (m, 2H, CH_2), 1.50 (q, *J* = 7.3 Hz, 2H, C*H*2), 1.02 (t, *J* = 7.3 Hz, 3H, CH_3); ^{13}C NMR (100 MHz, Chloroform-d [77.0 ppm], ppm) δ = 195.7 (1C, *C*O), 192.5 (1C, *C*O), 186.2 (1C, *C*O), 153.6 (1C, C_q), 143.0 (1C, C_q), 141.6 (1C, C_q), 139.2 (1C, C_q), 135.0 (1C, *C*H_2), 134.4 (1C, *C*H_{Ar}), 132.7 (1C, *C*H_{Ar}), 130.9 (1C, *C*H_{Ar}), 129.8 (1C, *C*H_{Ar}), 120.2 (1C, *C*H), 30.6 (1C, *C*H_2), 25.4 (1C, *C*H_2), 22.3 (1C, *C*H_2), 13.7 (1C, *C*H_3); MS (FAB, 3-NBA), m/z (%): 603 $[M]^+$ (67), 586 (100), 584 (87), 525 (21), 524 (78), 522 (50), 519 (24), 307 (23), 155 (30), 154 (100), 136 (73). HRMS ($C_{17}H_{15}O_3N_5{}^{79}Br{}^{187}Re_1$): calcd 602.9910, found 602.9909; IR (ATR, ṽ) = 3115 (w), 2961 (w), 2935 (w), 2861 (w), 2024 (vs), 1929 (vs), 1895 (vs), 1608 (w), 1551 (m), 1499 (s), 1445 (m), 1360 (s), 1273 (m), 1205 (m), 1139 (m), 1065 (m), 1041 (s), 1010 (m), 1000 (m), 966 (m), 902 (s), 871 (m), 799 (m), 765 (vs), 694 (w), 670 (w), 632 (vs), 565 (w), 535 (m), 506 (m), 479 (s), 419 (s), 384 (m) cm^{-1}; EA ($C_{17}H_{15}BrN_5O_3Re$): Calcd C 33.84; H 2.51; N 11.61. Found C 34.58; H 2.47; N 11.61;

Crystals suitable for Single Crystal X-ray Diffraction Analysis obtained via slow evaporation of a solution in DCM under ambient conditions. Crystal Data for $C_{17}H_{15}BrN_5O_3Re$ (M =603.45 g/mol): triclinic, space group P-1 (no. 2), a = 8.1736(3) Å, b = 9.8940(3) Å, c = 12.3886(4) Å, α = 68.789(3)°, β = 81.703(3)°, γ = 85.226(3)°, V = 923.69(6) Å^3, Z = 2, T = 180.0 K, μ(MoKα) = 8.769 mm^{-1}, $Dcalc$ = 2.170 g/cm^3, 12981 reflections measured (3.554° ≤ 2Θ ≤ 59.994°), 5361 unique (R_{int} = 0.0216, R_{sigma} = 0.0214) which were used in all calculations. The final R_1 was 0.0313 (I > 2σ(I)) and wR_2 was 0.0833 (all data); Data taken from another reaction with the same product compound UV/VIS (acetonitrile, 18 µM solution), λ = 424 (0.06), 354 (0.18), 344 (0.18), 300 (0.14), 254 (0.44) nm.

Additional information on the chemical synthesis is available via Chemotion repository: https://doi.org/10.14272/reaction/SA-FUHFF-UHFFFADPSC-CCAUEKYEPR-UHFFFADPSC-NUHFF-MUHFF-NUHFF-ZZZ.2

Additional information on the analysis of the target compound is available via Chemotion repository:
https://doi.org/10.14272/CCAUEKYEPREEHO-UHFFFAOYSA-M.2

[(2-(4-Phenyl-1*H*-1,2,3-triazol-1-yl)quinoxaline)]bromotricarbonylrhenium(I) (27b)

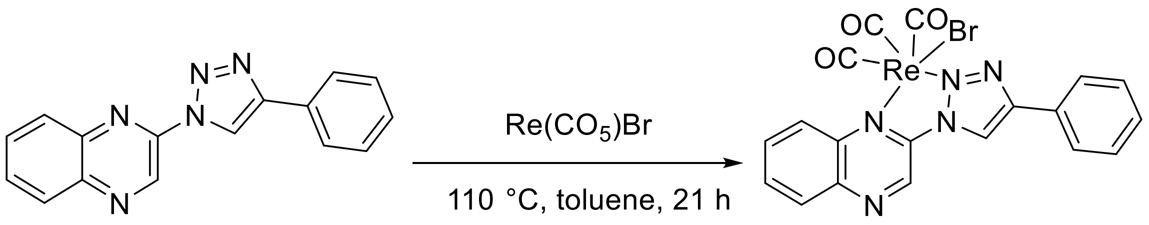

Name {P1|**27b**}: [(2-(4-phenyl-1*H*-1,2,3-triazol-1-yl)quinoxaline)]bromotricarbonylrhenium(I); Formula: $C_{19}H_{11}BrN_5O_3Re$; Molecular Mass: 623.4333; Exact Mass: 622.9603; Smiles: c1ccc(cc1)c1nnn(c1)c1cnc2c(n1)cccc2.[C-]#[O+].[C-]#[O+].[C-]#[O+].Br[Re]; InChIKey: JFQQXHCZLVWKOL-UHFFFAOYSA-M

The ligand 2-(4-phenyl-1*H*-1,2,3-triazol-1-yl)quinoxaline (39.0 mg, 143 µmol, 1.00 equiv) was dissolved in dry toluene (2.00 mL) and heated to 110 °C under argon, then bromorhenium;carbon monoxide (66.0 mg, 162 µmol, 1.14 equiv) was added and another 0.50 mL of toluene was added to rinse the walls of the flask. The solution was stirred at 110 °C under argon for 5 h and subsequently the red mixture was cooled to 25 °C and stirred for 16 h; then the solvent was pipetted off and the red precipitate was dried under high vacuum. The rhenium complex [(2-(4-phenyl-1*H*-1,2,3-triazol-1-yl)quinoxaline)]bromotricarbonylrhenium(I) (56.0 mg, 89.8 µmol, 63% yield) was obtained as a red solid.

^{1}H NMR (400 MHz, DMSO-d_6, ppm) δ = 10.51 (s, 1H, NC*H*), 10.04 (s, 1H, C$H_{triazole}$), 8.56 (d, ^3J = 8.8 Hz, 1H, CH_{ar}), 8.47 (dd, ^3J = 8.3 Hz, ^4J = 1.6 Hz, 1H, CH_{ar}), 8.37–8.32 (m, 1H, CH_{ar}), 8.06 (d, ^3J = 7.1 Hz, 2H), 7.66 (t, ^3J = 7.5 Hz, 2H, CH_{ar}), 7.59–7.57 (m, 1H, CH_{ar}), 7.26–7.12 (m, 1H, CH_{ar}). Signals at 9.78, 9.60, 8.27–8.11, 8.03–7.94, 7.55–

7.41 ppm belong to the free ligand due to dissociation of the complex (spectrum of free ligand in DMSO-d_6: https://dx.doi.org/10.14272/QYOUUXWQIVRDNZ-UHFFFAOYSA-N.2); ^{13}C NMR (100 MHz, DMSO-d_6, ppm) δ = 196.5 (1C, *C*O), 194.3 (1C, *C*O), 186.7 (1C, *C*O), 150.0, 142.6, 142.0, 138.5, 137.4, 134.5, 132.6, 130.5, 130.2, 129.6 (2C), 128.4, 127.2, 125.8 (2C), 123.2 Signals from free ligand: 147.5, 142.9, 141.6, 139.2, 138.2, 131.9, 130.6, 129.6, 129.2, 129.0, 128.6, 128.5, 125.8, 118.9; IR (ATR, ṽ) = 3067 (m), 3053 (m), 3019 (m), 2955 (m), 2921 (m), 2851 (m), 2027 (vs), 1951 (vs), 1918 (vs), 1883 (vs), 1863 (vs), 1732 (m), 1667 (m), 1553 (m), 1502 (s), 1476 (s), 1455 (s), 1438 (s), 1370 (s), 1357 (s), 1290 (m), 1268 (s), 1242 (m), 1205 (s), 1183 (m), 1160 (m), 1135 (s), 1082 (s), 1037 (vs), 1027 (s), 969 (s), 959 (s), 925 (m), 911 (s), 834 (s), 772 (s), 761 (vs), 703 (s), 694 (vs), 640 (vs), 635 (s), 623 (s), 569 (m), 530 (s), 507 (s), 490 (vs), 482 (vs), 465 (s), 416 (s) cm^{-1}; EA ($C_{19}H_{11}BrN_5O_3Re$): Calcd C 36.60; H 1.78; N 11.23. Found C 38.10; H 2.16; N 10.54; Crystals suitable for Single Crystal X-Ray Diffraction Analysis obtained via slow evaporation of a diluted solution in EtOAc under ambient conditions. Crystal Data for $C_{19}H_{11}BrN_5O_3Re$ (M =623.44 g/mol): monoclinic, space group P21/n (no. 14), a = 11.5453(4) Å, b = 14.0610(5) Å, c = 12.4364(4) Å, β = 110.258(3)°, V = 1894.02(12) Å3, Z = 4, T = 180.0 K, μ(GaKα) = 10.489 mm-1, *Dcalc* = 2.186 g/cm3, 12569 reflections measured (8.568° ≤ 2Θ ≤ 124.984°), 4465 unique (*R*int = 0.0138, Rsigma = 0.0131) which were used in all calculations. The final *R*1 was 0.0251 (I > 2σ(I)) and *wR*2 was 0.0630 (all data); UV/VIS (acetonitrile, 18 µM solution) = 428 (0.05), 358 (0.15), 316 (0.13), 260 (0.63) nm.

Additional information on the chemical synthesis is available via Chemotion repository: https://doi.org/10.14272/reaction/SA-FUHFF-UHFFFADPSC-JFQQXHCZLV-UHFFFADPSC-NUHFF-MUHFF-NUHFF-ZZZ

Additional information on the analysis of the target compound is available via Chemotion repository:
https://doi.org/10.14272/JFQQXHCZLVWKOL-UHFFFAOYSA-M.1

[2-(4-Butyl-1*H*-1,2,3-triazol-1-yl)-3-methylquinoxaline]bromotricarbonylrhenium(I) (27c)

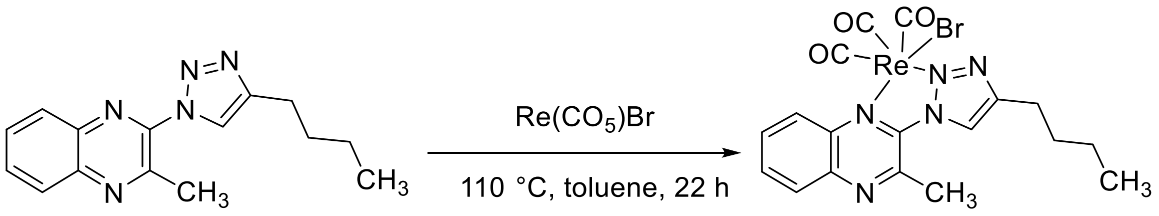

Name {P1|**27c**}: [2-(4-butyl-1*H*-1,2,3-triazol-1-yl)-3-methylquinoxaline]bromotricarbonylrhenium(I); Formula: $C_{18}H_{17}BrN_5O_3Re$; Molecular Mass: 617.4703; Exact Mass: 617.0072; Smiles: CCCCc1nnn(c1)c1nc2ccccc2nc1C.[C-]#[O+].[C-]#[O+].[C-]#[O+].Br[Re]; InChIKey: DDQBGZVUDRTHAU-UHFFFAOYSA-M

The ligand 2-(4-butyl-1*H*-1,2,3-triazol-1-yl)-3-methylquinoxaline (29.0 mg, 108 µmol, 1.00 equiv) was dissolved in dry toluene (1.50 mL) and heated to 110 °C under argon, then bromorhenium;carbon monoxide (54.0 mg, 133 µmol, 1.23 equiv) was added; another 0.50 mL of toluene was added to rinse the walls of the flask. The solution was stirred at 110 °C under argon for 4.5 h and subsequently the red mixture was cooled to 25 °C and stirred for 16 h at 25 °C. Then the solvent was evaporated under reduced pressure. The obtained crude product was purified via flash chromatography (dryload on Celite, *c*Hex -> EtOAc) and the rhenium complex [2-(4-butyl-1*H*-1,2,3-triazol-1-yl)-3-methylquinoxaline]bromotricarbonylrhenium(I) (22.0 mg, 35.6 µmol, 33% yield) was obtained as a red solid. 25 mg of an unknown product were isolated.

R_f = 0.39 (cyclohexane/ethyl acetate 1:2). ^{1}H NMR (400 MHz, $CDCl_3$, ppm) δ = 8.78 (dd, ^3J = 8.6 Hz, ^4J = 1.5 Hz, 1H, CH_{ar}), 8.59 (s, 1H, C$H_{triazole}$), 8.23 (dd, ^3J = 8.2 Hz, ^4J = 1.7 Hz, 1H, CH_{ar}), 8.11–8.00 (m, 2H, CH_{ar}), 3.28 (s, 3H, CH_3), 2.99–2.94 (m, 2H, CH_2), 1.88–1.79 (m, 2H, CH_2), 1.54–1.46 (m, 2H, CH_2), 1.02 (t, ^3J = 7.3 Hz, 3H, CH_2CH_3); ^{13}C NMR (100 MHz, $CDCl_3$ [77.0 ppm], ppm) δ = 195.9 (1C, *C*O), 192.9 (1C, *C*O), 186.8 (1C, *C*O), 152.8 (1C, C_q), 143.1 (1C, C_q), 142.9 (1C, C_q), 141.8 (1C, C_q), 138.4 (1C, C_q), 133.8 (1C, CH_{ar}), 132.6 (1C, CH_{ar}), 130.3 (1C, CH_{ar}), 129.7 (1C, CH_{ar}), 123.2 (1C, $CH_{triazole}$), 30.8 (1C, CH_2), 25.9 (1C, CH_3), 25.4 (1C, CH_2), 22.3 (1C, CH_2), 13.7 (1C, CH_3). MS (FAB, 3-NBA), m/z (%): 617 (1), 530 (17), 191 (17), 154 (18), 147 (27), 136 (19), 131 (17), 129 (15), 128 (17), 115 (21), 105 (18). IR (ATR, ṽ) = 3172 (w), 2951 (w), 2932 (m), 2921 (w), 2901 (w), 2856 (w), 2024 (vs), 1924 (vs), 1858 (vs), 1606 (w), 1570 (w), 1561 (w), 1537 (m), 1487 (m), 1462 (m), 1436 (m), 1426 (m), 1392 (w), 1375 (m), 1363 (s), 1326 (m), 1292 (m), 1265 (m), 1242 (m), 1204 (m), 1173 (m), 1133 (s), 1101 (w), 1067 (s), 1055 (m), 1034 (w), 1017 (m), 990 (s), 967 (m), 925 (w), 901 (m), 878 (m), 805 (m), 782 (m), 768 (vs), 731 (m), 704 (m), 688 (w), 639 (s), 626 (vs), 535 (m), 517 (m), 511 (m), 486 (vs), 470 (s), 459 (m) cm^{-1}. Crystals suitable for Single Crystal X-Ray Diffraction Analysis obtained via slow evaporation of a solution in DCM under ambient conditions. Crystal Data for $C_{18}H_{17}BrN_5O_3Re$ (M =617.47 g/mol): triclinic, space group P-1 (no. 2), a = 8.1070(3) Å, b = 9.9680(3) Å, c = 12.8159(4) Å, α = 105.330(3)°, β = 101.878(3)°, γ = 94.429(3)°, V = 967.87(6) Å3, Z = 2, T = 150 K, μ(GaKα) = 10.252 mm-1, Dcalc = 2.119 g/cm3, 10723 reflections measured (6.402° ≤ 2Θ ≤ 124.97°), 4524 unique (Rint = 0.0160, Rsigma = 0.0122) which were used in all calculations. The final R1 was 0.0224 (I > 2σ(I)) and wR2 was 0.0604 (all data). UV/VIS (acetonitrile, 18 µM solution), λ = 422 (0.06), 358 (0.19), 344 (0.17), 296 (0.14), 248 (0.44) nm.

Additional information on the chemical synthesis is available via Chemotion repository: https://doi.org/10.14272/reaction/SA-FUHFF-UHFFFADPSC-DDQBGZVUDR-UHFFFADPSC-NUHFF-MUHFF-NUHFF-ZZZ

Additional information on the analysis of the target compound is available via Chemotion repository:
https://doi.org/10.14272/DDQBGZVUDRTHAU-UHFFFAOYSA-M.1

[2-(4-Butyl-1*H*-1,2,3-triazol-1-yl)-3-phenylquinoxaline]bromotricarbonylrhenium(I) (**27d**)

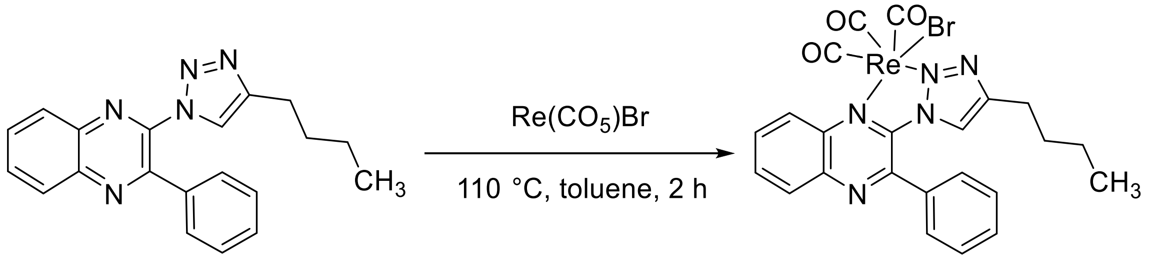

Name {P1|**27d**}: [2-(4-butyl-1*H*-1,2,3-triazol-1-yl)-3-phenylquinoxaline]bromotricarbonylrhenium(I); Formula: $C_{23}H_{19}BrN_5O_3Re$; Molecular Mass: 679.5397; Exact Mass: 679.0229; Smiles: CCCCc1nnn(c1)c1nc2ccccc2nc1c1ccccc1.[C-]#[O+].[C-]#[O+].[C-]#[O+].Br[Re]; InChIKey: BKFBOTQHKAJKOY-UHFFFAOYSA-M

The ligand 2-(4-butyl-1*H*-1,2,3-triazol-1-yl)-3-phenylquinoxaline (22.0 mg, 66.8 µmol, 1.00 equiv) was dissolved in dry toluene (1.00 mL) and heated to 110 °C under argon, then bromorhenium;carbon monoxide (29.8 mg, 73.5 µmol, 1.10 equiv) was added. Another 0.50 mL of toluene was added to rinse the walls of the flask. The solution was stirred at 110 °C under argon for 2 h and subsequently the red mixture was cooled to 25 °C; then the solvent was evaporated under reduced pressure. The obtained mixture was purified twice via flash chromatography (eluent *c*Hex/EtOAc 2:1) and the desired metal complex [2-(4-butyl-1*H*-1,2,3-triazol-1-yl)-3-phenylquinoxaline]bromotricarbonylrhenium(I) (15.0 mg, 22.1 µmol, 33% yield) was obtained as a red solid.

R_f = 0.39 (cyclohexane/ethyl acetate 2:1). ^{1}H NMR (400 MHz, $CDCl_3$, ppm) δ = 8.76 (d, ^{3}J = 10.1 Hz, 1H, CH_{ar}), 8.32 (dd, ^{3}J = 8.3 Hz, ^{4}J = 1.6 Hz, 1H, CH_{ar}), 8.15–8.11 (m, 1H, CH_{ar}), 8.07–8.03 (m, 1H, CH_{ar}), 7.74–7.71 (m, 5H, CH_{ar}), 7.06 (s, 1H, C$H_{triazole}$), 2.69 (t, ^{3}J = 8.1 Hz, 2H, CH_2), 1.59–1.52 (m, 2H, CH_2), 1.34–1.26 (m, 2H, CH_2), 0.91 (t, ^{3}J = 7.3 Hz, 3H, CH_3); ^{13}C NMR (ppm) δ = 195.8 (1C, *C*O), 192.9 (1C, *C*O), 187.0 (1C, *C*O), 151.3 (1C, C_q), 145.9 (1C, C_q), 142.3 (1C, C_q), 141.6 (1C, C_q), 138.8 (1C, C_q), 135.0 (1C, C_q), 134.3 (1C, CH_{ar}), 132.8 (1C, CH_{ar}), 131.6 (1C, CH_{ar}), 130.4 (1C, CH_{ar}), 130.0 (3C, CH_{ar}), 129.2 (2C, CH_{ar}), 123.5 (1C, $CH_{triazole}$), 30.2 (1C, CH_2), 25.0 (1C, CH_2), 21.9 (1C, CH_2), 13.6 (1C, CH_3). Assignment of the carbons between signals at 130.0 and 129.2 ambigous: 130.0 (2C, CH_{ar}), 129.2 (3C, CH_{ar}) is also a possible constellation. MS (FAB, 3-NBA), m/z (%): 681 $[M+2]^+$ (24), 680 $[M+1]^+$ (15), 679 $[M]^+$ (37), 662 (20), 600 (33), 598 (22), 205 (33), 155 (31), 154 (100), 147 (20), 139 (23), 138 (43), 137 (57), 136 (86), 115 (21), 107 (37), 105 (24), 97 (21), 95 (34), 91 (49), 89 (27). HRMS ($C_{23}H_{19}O_3N_5{}^{79}Br_1{}^{187}Re_1$): calcd 679.0223, found 679.0225. IR (ATR, ṽ) = 3160 (vw), 2956 (w), 2929 (w), 2864 (w), 2023 (vs), 1938 (s), 1902 (vs), 1568 (w), 1533 (w), 1483 (w), 1469 (w), 1441 (m), 1432 (m), 1395 (w), 1361 (m), 1339 (w), 1266 (w), 1217 (w), 1191 (w), 1139 (w), 1061 (w), 1045 (m), 1013 (w), 973 (w), 793 (w), 766 (m), 734 (w), 700 (m), 639 (m), 630 (m), 567 (w), 523 (w), 514 (w), 482 (m) cm^{-1}. Crystals suitable for Single Crystal X-Ray Diffraction Analysis obtained via slow evaporation of a solution in $CDCl_3$ under ambient conditions. Crystal Data for $C_{24}H_{20}BrCl_3N_5O_3Re$ (M =798.91 g/mol): monoclinic, space group C2/c (no. 15), a = 26.7745(6) Å, b = 17.4882(5) Å, c = 12.7656(3) Å, β = 112.170(2)°, V = 5535.4(3) Å3, Z = 8, T = 180 K, μ(GaKα) = 9.025 mm-1, Dcalc = 1.917 g/cm3, 17394 reflections measured (7.488° ≤ 2Θ ≤ 124.994°), 6505 unique (Rint = 0.0156, Rsigma = 0.0120) which were used in all

calculations. The final R1 was 0.0306 (I > 2σ(I)) and wR2 was 0.0780 (all data). UV/VIS (acetonitrile, 18 µM solution), λ = 432 (0.06), 362 (0.16), 254 (0.51) nm.

Additional information on the chemical synthesis is available via Chemotion repository: https://doi.org/10.14272/reaction/SA-FUHFF-UHFFFADPSC-BKFBOTQHKA-UHFFFADPSC-NUHFF-MUHFF-NUHFF-ZZZ

Additional information on the analysis of the target compound is available via Chemotion repository: https://doi.org/10.14272/BKFBOTQHKAJKOY-UHFFFAOYSA-M.1

[*N,N*-diethyl-2-(1-(quinoxalin-2-yl)-1*H*-1,2,3-triazol-4-yl)ethan-1-amine]bromotricarbonylrhenium(I) (**29**)

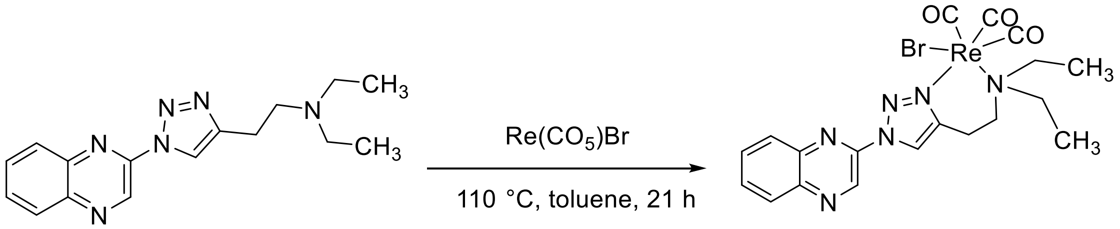

Name {P1|**29**}: [*N,N*-diethyl-2-(1-(quinoxalin-2-yl)-1*H*-1,2,3-triazol-4-yl)ethan-1-amine]bromotricarbonylrhenium(I); Formula: $C_{19}H_{20}BrN_6O_3Re$; Molecular Mass: 646.5115; Exact Mass: 646.0338; Smiles: CCN(CCc1nnn(c1)c1cnc2c(n1)cccc2)CC.[C-]#[O+].[C-]#[O+].[C-]#[O+].Br[Re]; InChIKey: LHPVGPJSNAKQBR-UHFFFAOYSA-M

The ligand *N,N*-diethyl-2-(1-(quinoxalin-2-yl)-1*H*-1,2,3-triazol-4-yl)ethan-1-amine (40.0 mg, 135 µmol, 1.00 equiv) was dissolved in dry toluene (1.50 mL) and heated to 110 °C under argon, then bromorhenium;carbon monoxide (60.0 mg, 148 µmol, 1.09 equiv) was added; another 0.50 mL of toluene was added to rinse the walls of the flask. The solution was stirred at 110 °C under argon for 6 h and subsequently the orange mixture was stirred for 16 h at 25 °C. Then the orange-brown solution was carefully pipetted off, the precipitated yellow solid was collected and dried under high vacuum. The rhenium complex [*N,N*-diethyl-2-(1-(quinoxalin-2-yl)-1*H*-1,2,3-triazol-4-yl)ethan-1-amine]bromotricarbonylrhenium(I) (84.0 mg, 130 µmol, 96% yield) was obtained as a yellow solid.

R_f = 0.35 (cyclohexane/ethyl acetate 1:2). ^{1}H NMR (400 MHz, $CDCl_3$, ppm) δ = 9.81 (s, 1H, NCH_{ar}), 8.67 (s, 1H, C$H_{triazole}$), 8.28–8.24 (m, 1H, CH_{ar}), 8.10–8.05 (m, 1H, CH_{ar}), 7.93–7.88 (m, 2H, CH_{ar}), 3.72–3.61 (m, 3H, CH_2), 3.58–3.49 (m, 1H, CH_2), 3.30–3.14 (m, 3H, CH_2), 3.00–2.94 (m, 1H, CH_2), 1.39 (t, ^3J = 7.2 Hz, 3H, CH_3), 1.22 (t, ^3J = 7.1 Hz, 3H, CH_3); ^{13}C NMR (100 MHz, $CDCl_3$ [77.0 ppm], ppm) δ = 196.8 (1C, *C*O), 191.8 (1C, *C*O), 177.7 (1C, *C*O), 145.3 (1C, C_q), 142.9 (1C, C_q), 141.4 (1C, C_q), 139.5 (1C, C_q), 137.3 (1C, NCH_{ar}), 132.0 (1C, CH_{ar}), 131.2 (1C, CH_{ar}), 129.9 (1C, CH_{ar}), 128.8 (1C, CH_{ar}), 119.5 (1C, $CH_{triazole}$), 55.7 (1C, CH_2), 53.5 (1C, CH_2), 52.6 (1C, CH_2), 21.0 (1C, CH_2), 10.6 (1C, CH_3), 9.1 (1C, CH_3). MS (FAB, 3-NBA), m/z (%): 646 [M]+ (4), 307 (36), 289 (16), 155 (35), 154 (100), 137 (70), 107 (20). HRMS (FAB, $C_{19}H_{20}O_3N_6{}^{79}Br_1{}^{187}Re_1$): calcd 646.0332, found 646.0331. IR (ATR, ṽ) = 3088 (w), 3002 (w), 2979 (w), 2946 (w), 2927 (w), 2878 (w), 2027 (vs), 1914 (vs), 1874 (vs), 1839 (vs), 1609 (w), 1561 (m), 1497 (s), 1472 (m), 1448 (s), 1432 (m), 1364 (m), 1347

(m), 1264 (m), 1248 (m), 1232 (m), 1207 (w), 1193 (m), 1170 (w), 1137 (m), 1123 (m), 1089 (m), 1075 (m), 1016 (s), 1007 (m), 950 (s), 915 (m), 871 (w), 864 (w), 822 (m), 792 (w), 772 (vs), 735 (s), 713 (m), 676 (w), 650 (s), 635 (m), 615 (w), 588 (m), 551 (w), 524 (s), 487 (s), 475 (m), 416 (s), 378 (m) cm^{-1}. Crystals suitable for Single Crystal X-Ray Diffraction Analysis obtained via slow evaporation of a solution in DCM under ambient conditions. Crystal Data for $C_{20.5}H_{21.5}BrCl_{4.5}N_6O_3Re$ (M =825.57 g/mol): monoclinic, space group C2/c (no. 15), a = 27.0130(7) Å, b = 16.1381(5) Å, c = 13.5743(3) Å, β = 106.059(2)°, V = 5686.6(3) Å3, Z = 8, T = 180 K, μ(Mo Kα) = 6.136 mm-1, Dcalc = 1.929 g/cm3, 28083 reflections measured (2.972° ≤ 2Θ ≤ 70.692°), 11833 unique (Rint = 0.0452, Rsigma = 0.0460) which were used in all calculations. The final R1 was 0.0776 (I > 2σ(I)) and wR2 was 0.2302 (all data). UV/VIS (acetonitrile, 18 μM solution), λ = 340 (0.20), 256 (0.46) nm.

Additional information on the chemical synthesis is available via Chemotion repository: https://doi.org/10.14272/reaction/SA-FUHFF-UHFFFADPSC-LHPVGPJSNA-UHFFFADPSC-NUHFF-MUHFF-NUHFF-ZZZ

Additional information on the analysis of the target compound is available via Chemotion repository:
https://doi.org/10.14272/LHPVGPJSNAKQBR-UHFFFAOYSA-M.1

[1-Butyl-4-(4-butyl-1*H*-1,2,3-triazol-1-yl)imidazo[1,2-*a*]quinoxaline]bromotricarbonylrhenium(I) (30)

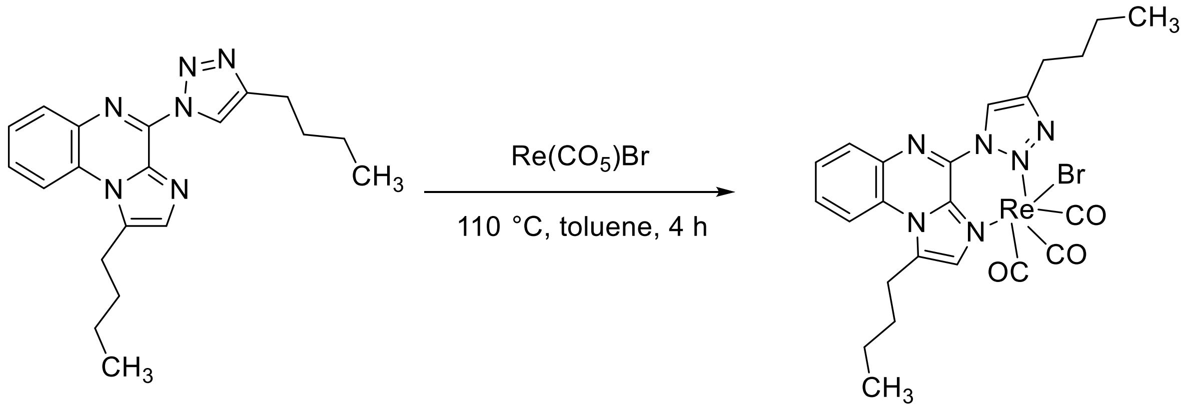

Name {P1|**30**}: [1-butyl-4-(4-butyl-1*H*-1,2,3-triazol-1-yl)imidazo[1,2-*a*]quinoxaline]bromotricarbonylrhenium(I); Formula: $C_{23}H_{24}BrN_6O_3Re$; Molecular Mass: 698.5861; Exact Mass: 698.0651; Smiles: CCCCc1nnn(c1)c1nc2ccccc2n2c1ncc2CCCC.[C-]#[O+].[C-]#[O+].[C-]#[O+].Br[Re]; InChIKey: IYHBIYUJPQGBCO-UHFFFAOYSA-M

In a two-necked flask, the ligand 1-butyl-4-(4-butyl-1*H*-1,2,3-triazol-1-yl)imidazo[1,2-a]quinoxaline (24.0 mg, 68.9 μmol, 1.00 equiv) was dissolved in dry toluene (1.50 mL) and heated to 110 °C under argon, then bromorhenium;carbon monoxide (34.0 mg, 83.7 μmol, 1.22 equiv) was added. Another 0.50 mL of toluene was added and the solution was stirred at 110 °C under argon for 4 h. Subsequently the red mixture was cooled to 25 °C and the solvent was evaporated under reduced pressure. The obtained crude product was purified twice via column chromatography (*c*Hex/ethyl

acetate, then DCM -> DCM/EtOAc 50:1) and the rhenium complex [1-butyl-4-(4-butyl-1*H*-1,2,3-triazol-1-yl)imidazo[1,2-*a*]quinoxaline]bromotricarbonylrhenium(I) (38.0 mg, 54.4 µmol, 79% yield) was obtained as an orange solid.

R_f = 0.61 (CH_2Cl_2). ^{1}H NMR (400 MHz, $CDCl_3$, ppm) δ = 8.96 (s, 1H, $CH_{triazole}$), 8.35 (dd, ^{3}J = 8.3 Hz, ^{4}J = 1.6 Hz, 1H, CH_{ar}), 8.16 (dd, ^{3}J = 7.9 Hz, ^{4}J = 1.8 Hz, 1H, CH_{ar}), 7.91 (s, 1H, $CH_{imidazole}$), 7.86–7.66 (m, 2H, CH_{ar}), 3.39–3.35 (m, 2H, C_qCH_2), 2.94–2.90 (m, 2H, C_qCH_2), 2.03–1.96 (m, 2H, CH_2CH_2), 1.86–1.78 (m, 2H, CH_2CH_2), 1.66 (h, ^{3}J = 7.3 Hz, 2H, CH_2CH_3), 1.51 (h, ^{3}J = 7.5 Hz, 2H, CH_2CH_3), 1.10 (t, ^{3}J = 7.3 Hz, 3H, CH_3), 1.02 (t, ^{3}J = 7.4 Hz, 3H, CH_3); ^{13}C NMR (100 MHz, $CDCl_3$ [77.0 ppm], ppm) δ = 195.7 (1C, *C*O), 193.8 (1C, *C*O), 191.6 (1C, *C*O), 150.7 (1C, C_q), 136.3 (1C, C_q), 136.2 (1C, CH_{ar}), 134.5 (1C, C_q), 134.4 (1C, C_q), 130.9 (1C, CH_{ar}), 130.6 (1C, CH_{ar}), 129.2 (1C, C_q), 128.6 (1C, CH_{ar}), 128.5 (1C, C_q), 121.5 (1C, $CH_{triazole}$), 115.9 (1C, $CH_{imidazole}$), 30.6 (1C, CH_2CH_2), 29.5 (1C, CH_2CH_2), 27.9 (1C, C_qCH_2), 25.2 (1C, C_qCH_2), 22.6 (1C, CH_2CH_3), 22.3 (1C, CH_2CH_3), 13.8 (1C, CH_3), 13.8 (1C, CH_3). MS (FAB, 3-NBA), m/z (%): 700 [M+2]+ (16), 698 [M]+ (28), 664 (22), 663 (57), 662 (32), 648 (16), 647 (33), 619 (20), 319 (18), 307 (20), 155 (33), 154 (100), 139 (21), 138 (38), 137 (59), 136 (70), 109 (16), 107 (25), 105 (17), 97 (20), 95 (25), 91 (28), 89 (17). HRMS (FAB, $C_{23}H_{24}O_3N_6{}^{79}Br_1{}^{187}Re_1$): calcd 698.0645, found 698.0643. IR (ATR, ṽ) = 3172 (w), 3119 (w), 2961 (w), 2931 (w), 2868 (w), 2031 (vs), 1921 (vs), 1866 (vs), 1594 (w), 1555 (w), 1506 (s), 1465 (s), 1443 (m), 1426 (m), 1395 (m), 1371 (m), 1364 (m), 1357 (m), 1323 (w), 1281 (w), 1252 (m), 1228 (m), 1183 (w), 1159 (w), 1132 (w), 1103 (w), 1064 (w), 1045 (vs), 1010 (w), 938 (m), 898 (w), 868 (w), 843 (w), 813 (w), 800 (w), 772 (vs), 732 (m), 722 (w), 705 (w), 664 (w), 642 (m), 632 (m), 623 (m), 596 (m), 534 (w), 521 (m), 476 (s), 459 (m), 388 (w) cm^{-1}. Crystals suitable for Single Crystal X-Ray Diffraction Analysis obtained via slow evaporation of a solution in acetonitrile under ambient conditions. Crystal Data for $C_{26.3}H_{29}BrN_{7.67}O_3Re$ (M =767.01 g/mol): triclinic, space group P-1 (no. 2), a = 12.6826(3) Å, b = 19.0014(5) Å, c = 21.4662(5) Å, α = 64.742(2)°, β = 74.174(2)°, γ = 71.344(2)°, V = 4376.3(2) Å3, Z = 6, T = 180 K, μ(Mo Kα) = 5.576 mm-1, Dcalc = 1.746 g/cm3, 53827 reflections measured (2.124° ≤ 2Θ ≤ 56°), 21092 unique (Rint = 0.1065, Rsigma = 0.0736) which were used in all calculations. The final R1 was 0.0665 (I > 2σ(I)) and wR2 was 0.1997 (all data). Asymmetric cell consists of three molecules of the complex and five molecules of acetonitrile. EA ($C_{23}H_{24}BrN_6O_3Re$): Calcd C 39.54; H 3.46; N 12.03. Found C 40.59; H 3.65; N 11.75. UV/VIS (acetonitrile), λ = 386 (0.16), 350 (0.17), 332 (0.18), 260 (0.42) nm.

Additional information on the chemical synthesis is available via Chemotion repository: https://doi.org/10.14272/reaction/SA-FUHFF-UHFFFADPSC-IYHBIYUJPQ-UHFFFADPSC-NUHFF-MUHFF-NUHFF-ZZZ

Additional information on the analysis of the target compound is available via Chemotion repository:
https://doi.org/10.14272/IYHBIYUJPQGBCO-UHFFFAOYSA-M.1

3. Absorption measurements

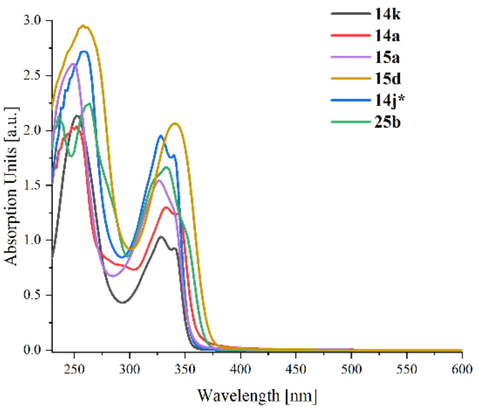

Figure S4: Qualitative UV–vis absorption spectra of the ligands.

4. Electrochemical measurements

The following cyclic voltammetry traces were recorded under the following conditions: 0.5 mM of the compound in MeCN solution with 0.1 M Bu_4NPF_6 under nitrogen at 25 °C, recorded at 0.1 V/s at a glassy carbon electrode and referenced to the saturated calomel electrode (SCE, 0.46 V vs. SCE [2]) using Fc/Fc^+ as an internal standard. For further information please see Section 1 : General Remarks.

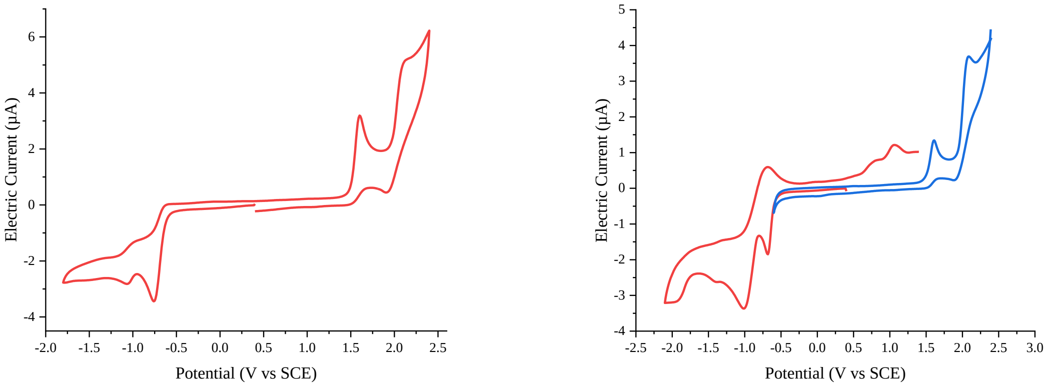

Figure S5: Cyclic voltammetry traces for **27a** (left) and **27b** (right) when scanning to more positive and negative potentials.

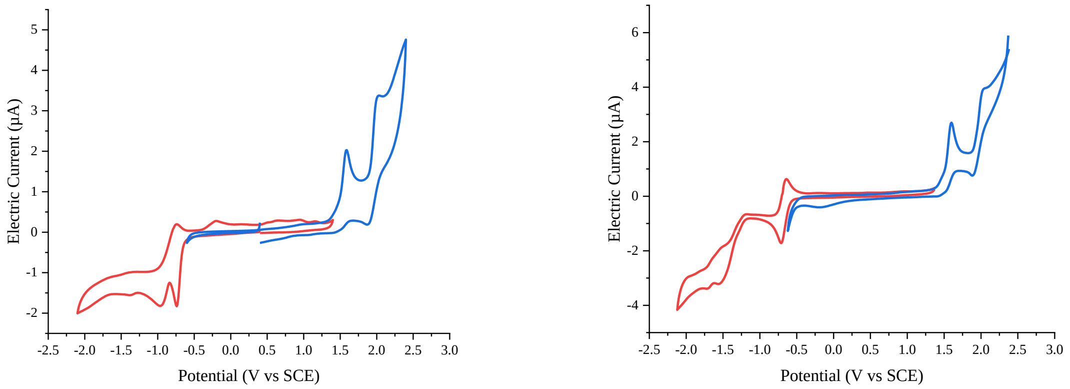

Figure S6: Cyclic voltammetry traces for **27c** (left) and **27d** (right) when scanning to more positive and negative potentials.

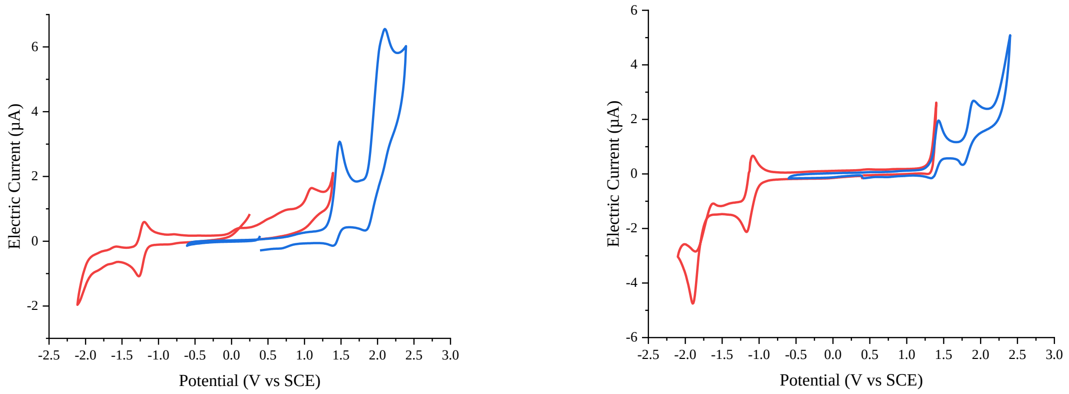

Figure S7: Cyclic voltammetry traces for **29** (left) and **30** (right) when scanning to more positive and negative potentials.

5. Crystallographic data

Table S7: Crystal data and structure refinement details for 25b, 27a-d, 29 and 30.

Compound	**25b**	**27a**	**27b**	**27c**
Empirical formula	$C_{20}H_{24}N_6$	$C_{17}H_{15}BrN_5O_3Re$	$C_{19}H_{11}BrN_5O_3Re$	$C_{18}H_{17}BrN_5O_3Re$
Formula weight	348.45	603.45	623.44	617.47
Temperature/K	150.0	180.0	180.0	150
Crystal system	triclinic	triclinic	monoclinic	triclinic
Space group	$P\bar{1}$	$P\bar{1}$	$P2_1/n$	$P\bar{1}$
a/Å	7.2494(4)	8.1736(3)	11.5453(4)	8.1070(3)
b/Å	9.0176(5)	9.8940(3)	14.0610(5)	9.9680(3)
c/Å	14.3850(8)	12.3886(4)	12.4364(4)	12.8159(4)
α/°	75.401(4)	68.789(3)	90	105.330(3)
β/°	85.805(4)	81.703(3)	110.258(3)	101.878(3)
γ/°	79.431(4)	85.226(3)	90	94.429(3)
Volume/Å^3	894.22(9)	923.69(6)	1894.02(12)	967.87(6)

Z	2	2	4	2
ρ_{calc}g/cm^3	1.294	2.170	2.186	2.119
μ/mm^{-1}	0.410	8.769	10.489	10.252
F(000)	372.0	572.0	1176.0	588.0
Radiation	GaKα (λ = 1.34143)	MoKα (λ = 0.71073)	GaKα (λ = 1.34143)	GaKα (λ = 1.34143)
2Θ range /°	5.53–125.0	3.55–60.0	8.57–125.0	6.40–125.0
Reflections collected	10270	12981	12569	10723
Independent reflections	4123 [R_{int} = 0.0447]	5361 [R_{int} = 0.0216]	4465 [R_{int} = 0.0138]	4524 [R_{int} = 0.0160]
Indep. refl. with I ≥ 2σ (I)	2974	5104	4122	4509
Data/restraints/parameters	4123/0/235	5361/0/245	4465/0/262	4524/0/321
Goodness-of-fit on F^2	1.379	1.058	1.148	1.105
Final R indexes [I ≥ 2σ (I)]	R_1 = 0.0944, wR_2 = 0.2907	R_1 = 0.0313, wR_2 = 0.0822	R_1 = 0.0251, wR_2 = 0.0622	R_1 = 0.0224, wR_2 = 0.0603
Final R indexes [all data]	R_1 = 0.1171, wR_2 = 0.3021	R_1 = 0.0332, wR_2 = 0.0833	R_1 = 0.0278, wR_2 = 0.0630	R_1 = 0.0225, wR_2 = 0.0604
Largest diff. peak/hole / e Å^{-3}	0.96/–0.88	1.68/–2.65	0.92/–0.98	0.81/–1.31
CCDC number	2129160	2129161	2129162	2129163

Table S7 (continued)

Compound	**27d**<br>**27d** · $CHCl_3$	**29**<br>**29** · 1.5 $CHCl_3$	**30**<br>**30** · 1.667 C_2H_3N
Empirical formula	$C_{24}H_{20}BrCl_3N_5O_3Re$	$C_{20.5}H_{21.5}BrCl_{4.5}N_6O_3Re$	$C_{26.333}H_{29}BrN_{7.667}O_3Re$
Formula weight	798.91	825.57	767.01
Temperature/K	180	180	180
Crystal system	monoclinic	monoclinic	triclinic
Space group	*C*2/*c*	*C*2/*c*	$P\bar{1}$
a/Å	26.7745(6)	27.0130(7)	12.6826(3)
b/Å	17.4882(5)	16.1381(5)	19.0014(5)
c/Å	12.7656(3)	13.5743(3)	21.4662(5)
α/°	90	90	64.742(2)
β/°	112.170(2)	106.059(2)	74.174(2)
γ/°	90	90	71.344(2)
Volume/Å^3	5535.4(3)	5686.6(3)	4376.3(2)
Z	8	8	6
ρ_{calc}g/cm^3	1.917	1.929	1.746
μ/mm^{-1}	9.025	6.136	5.576
F(000)	3072.0	3176.0	2248.0
Radiation	GaKα (λ = 1.34143)	Mo Kα (λ = 0.71073)	Mo Kα (λ = 0.71073)
2Θ range /°	7.49–125.0	2.97–70.7	2.12–56.0
Reflections collected	17394	28083	53827
Independent reflections	6505 [R_{int} = 0.0156]	11833 [R_{int} = 0.0452]	21092 [R_{int} = 0.1065]
Indep. refl. with I ≥ 2σ (I)	6401	9120	16718
Data/restraints/parameters	6505/0/332	11833/3/303	21092/0/1065
Goodness-of-fit on F^2	1.149	1.048	1.030
Final R indexes [I ≥ 2σ (I)]	R_1 = 0.0306,<br>wR_2 = 0.0775	R_1 = 0.0776,<br>wR_2 = 0.2157	R_1 = 0.0665,<br>wR_2 = 0.1857
Final R indexes [all data]	R_1 = 0.0312,<br>wR_2 = 0.0780	R_1 = 0.0953,<br>wR_2 = 0.2302	R_1 = 0.0835,<br>wR_2 = 0.1997
Largest diff. peak/hole / e Å^{-3}	1.07/–1.23	4.26/–7.08	4.51/–3.39
CCDC number	2129164	2129165	2129166

6. References

1. Swinehart, D. F. *J. Chem. Educ.* **1962,** *39*, 333. doi:10.1021/ed039p333
2. Bertrand, H. C.; Clède, S.; Guillot, R.; Lambert, F.; Policar, C. *Inorg. Chem.* **2014,** *53* (12), 6204–6223. doi:10.1021/ic5007007
3. Dolomanov, O. V.; Bourhis, L. J.; Gildea, R. J.; Howard, J. A. K.; Puschmann, H. *J Appl Crystallogr* **2009,** *42*, 339–341. doi:10.1107/S0021889808042726
4. Sheldrick, G. M. *Acta Crystallogr., Sect. A: Found. Adv.* **2015,** *71* (Pt 1), 3–8. doi:10.1107/S2053273314026370
5. Sheldrick, G. M. *Acta Crystallogr., Sect. C: Struct. Chem.* **2015,** *71* (Pt 1), 3–8. doi:10.1107/S2053229614024218

6. Chattopadhyay, B.; Vera, C. I. R.; Chuprakov, S.; Gevorgyan, V. *Org. Lett.* **2010,** *12*, 2166–2169. doi:10.1021/ol100745d
7. Roy, S.; Khatua, H.; Das, S. K.; Chattopadhyay, B. *Angew. Chem., Int. Ed.* **2019,** *58*, 11439–11443. doi:10.1002/anie.201904702
8. Dhameliya, T. M.; Chourasiya, S. S.; Mishra, E.; Jadhavar, P. S.; Bharatam, P. V.; Chakraborti, A. K. *J. Org. Chem.* **2017,** *82*, 10077–10091. doi:10.1021/acs.joc.7b01548
9. Dahn, H.; Leresche, J.-P.; Schlunke, H.-P. *Helv. Chim. Acta* **1966,** *49*, 26–30. doi:10.1002/hlca.660490106
10. Tian, M.; Liu, S.; Bu, X.; Yu, J.; Yang, X. *Chemistry (Weinheim an der Bergstrasse, Germany)* **2020,** *26*, 369–373. doi:10.1002/chem.201903523
11. Murthy, S. N.; Madhav, B.; Nageswar, Y. V. D. *Helv. Chim. Acta* **2010,** *93*, 1216–1220. doi:10.1002/hlca.200900358
12. Benali, B.; Lazar, Z.; Boucetta, A.; El Assyry, A.; Lakhrissi, B.; Massoui, M.; Jermoumi, C.; Negrier, P.; Leger, J. M.; Mondieig, D. *Spectrosc. Lett*. **2008,** *41*, 64–71. doi:10.1080/00387010801943525
13. Wang, H.; Wen, K.; Le Wang; Xiang, Y.; Xu, X.; Shen, Y.; Sun, Z. *Molecules (Basel, Switzerland)* **2012,** *17*, 4533–4544. doi:10.3390/molecules17044533
14. Xia, Q.-H.; Hu, W.; Li, C.; Wu, J.-F.; Yang, L.; Han, X.-M.; Shen, Y.-M.; Li, Z.-Y.; Li, X. *Eur. J. Med. Chem.* **2016,** *124*, 311–325. doi:10.1016/j.ejmech.2016.08.010
15. Li, Z.; Wang, X.; Xia, S.; Jin, J. *Org. Lett.* **2019,** *21*, 4259–4265. doi:10.1021/acs.orglett.9b01439
16. Wei, Z.; Qi, S.; Xu, Y.; Liu, H.; Wu, J.; Li, H.; Xia, C.; Duan, G. *Adv. Synth. Catal.* **2019,** *361*, 5490–5498. doi:10.1002/adsc.201900885
17. Raghunadh, A.; Tadiparthi, K.; Meruva, S. B.; Murthy, V. N.; Rao, L. V.; Kumar, U. K. S. *SynOpen* **2020,** *04*, 55–61. doi:10.1055/s-0040-1707203
18. Wagle, S.; Adhikari, A. V.; Kumari, N. S. *Eur. J. Med. Chem.* **2009,** *44*, 1135–1143. doi:10.1016/j.ejmech.2008.06.006
19. Wentrup, C.; Thétaz, C.; Tagliaferri, E.; Lindner, H. J.; Kitschke, B.; Winter, H.-W.; Reisenauer, H. P. *Angew. Chem.* **1980,** *92*, 556–557. doi:10.1002/ange.19800920714
20. Lippmann, E.; Tober, E. *J. Prakt. Chem.* **1982,** *324* (2), 329–334. doi:10.1002/prac.19823240217
21. Holzschneider, K.; Tong, M. L.; Mohr, F.; Kirsch, S. F. *Chemistry (Weinheim an der Bergstrasse, Germany)* **2019,** *25*, 11725–11733. doi:10.1002/chem.201902131
22. Muelbaier, M.; Ramsden, N.; Thomson, D. Preparation of tetrazoloquinoxaline derivatives as tankyrase inhibitors, Sep 19, 2013.
23. Dreikorn, B. A.; Thibault, T. D. 4,5-Dihydrotetrazolo(1,5-a)quinoxalines, Jun 10, 1975.
24. Rauws, T. R.; Biancalani, C.; Schutter, J. W. de; Maes, B. U. *Tetrahedron* **2010,** *66*, 6958–6964. doi:10.1016/j.tet.2010.06.038
25. Falck, J. R.; He, A.; Fukui, H.; Tsutsui, H.; Radha, A. *Angew. Chem., Int. Ed.* **2007,** *46*, 4527–4529. doi:10.1002/anie.200700321
26. Alumasa, J. N.; Manzanillo, P. S.; Peterson, N. D.; Lundrigan, T.; Baughn, A. D.; Cox, J. S.; Keiler, K. C. *ACS Infect. Dis.* **2017,** *3*, 634–644. doi:10.1021/acsinfecdis.7b00028
27. Kawski, A.; Nowaczyk, K.; Kuklinski, B. *Z. Naturforsch., A: Phys. Sci.* **1991,** *46*, 700–702. doi:10.1515/zna-1991-0808

28. Didenko, A. V.; Vorobiev, M. V.; Sevenard, D. V.; Sosnovskikh, V. Y. *Chem Heterocycl Comp* **2015,** *51*, 259–268. doi:10.1007/s10593-015-1694-5
29. Krasavin, M.; Shkavrov, S.; Parchinsky, V.; Bukhryakov, K. *J. Org. Chem.* **2009,** *74*, 2627–2629. doi:10.1021/jo900050k
30. Adam, M. S. S.; Kindermann, M. K.; Köckerling, M.; Heinicke, J. W. *Eur. J. Org. Chem.* **2009,** *2009* (27), 4655–4665. doi:10.1002/ejoc.200900698
31. Sun, D.; Watson, W. H. *J. Org. Chem.* **1997,** *62*, 4082–4084. doi:10.1021/jo970197m
32. Deleuze-Masquefa, C.; Moarbess, G.; Bonnet, P.-A.; Pinguet, F.; Bazarbachi, A.; Bressolle, F. Synthesis of imidazo[l,2-a]quinoxalines for treating cancers.
