# Supplementary material for: Scope of tetrazolo[1,5-a]quinoxalines in CuAAC reactions for the synthesis of triazoloquinoxalines, imidazoloquinoxalines, and rhenium complexes thereof
Source: Beilstein J Org Chem. 2022 Aug 24;18:1088–99. doi: 10.3762/bjoc.18.111 (PMC9443424; doi:10.3762/bjoc.18.111)

BEILSTEIN JOURNAL OF ORGANIC CHEMISTRY

Supporting Information

for

Scope of tetrazolo[1,5-*a*]quinoxalines in CuAAC reactions for the synthesis of triazoloquinoxalines, imidazoloquinoxalines, and rhenium complexes thereof

Laura Holzhauer, Chloé Liagre, Olaf Fuhr, Nicole Jung and Stefan Bräse

Beilstein J. Org. Chem. **2022,** *18,* 1088–1099. doi:10.3762/bjoc.18.111

NMR spectra

License and Terms: This is a supporting information file under the terms of the Creative Commons Attribution License (https://creativecommons.org/licenses/by/4.0). Please note that the reuse, redistribution and reproduction in particular requires that the author(s) and source are credited and that individual graphics may be subject to special legal provisions.
The license is subject to the *Beilstein Journal of Organic Chemistry* terms and conditions: (https://www.beilstein-journals.org/bjoc/terms)

[**S2a**] 1*H*-Quinoxalin-2-one

CHMO:0000593 | ^{1}H nuclear magnetic resonance spectroscopy (^{1}H NMR)

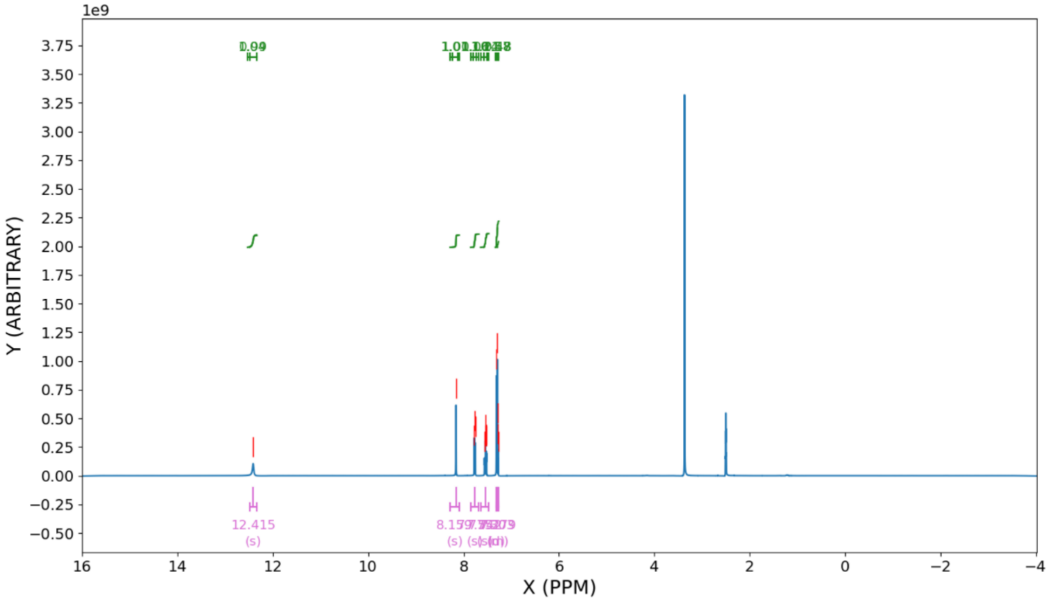

CHMO:0000595 | ^{13}C nuclear magnetic resonance spectroscopy (^{13}C NMR)

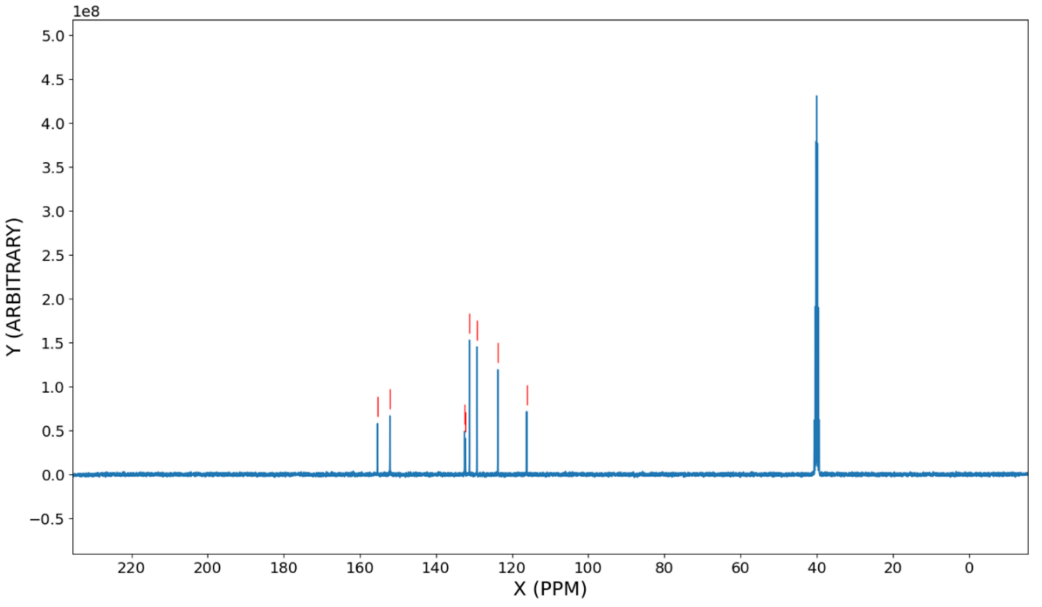

[**S2b**] 3-Methyl-1*H*-quinoxalin-2-one

CHMO:0000593 | ^{1}H nuclear magnetic resonance spectroscopy (^{1}H NMR)

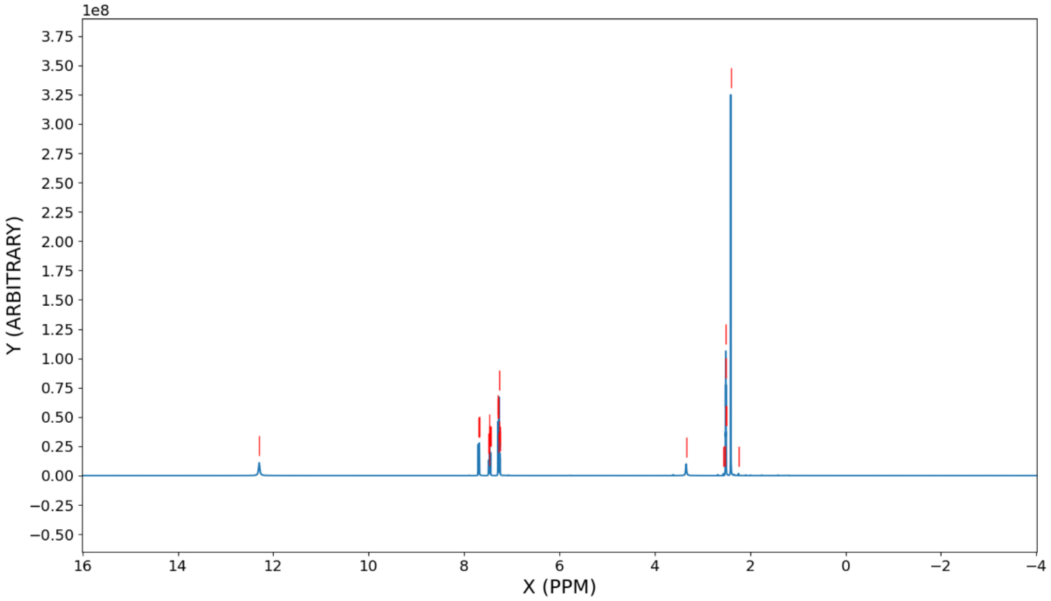

CHMO:0000595 | ^{13}C nuclear magnetic resonance spectroscopy (^{13}C NMR)

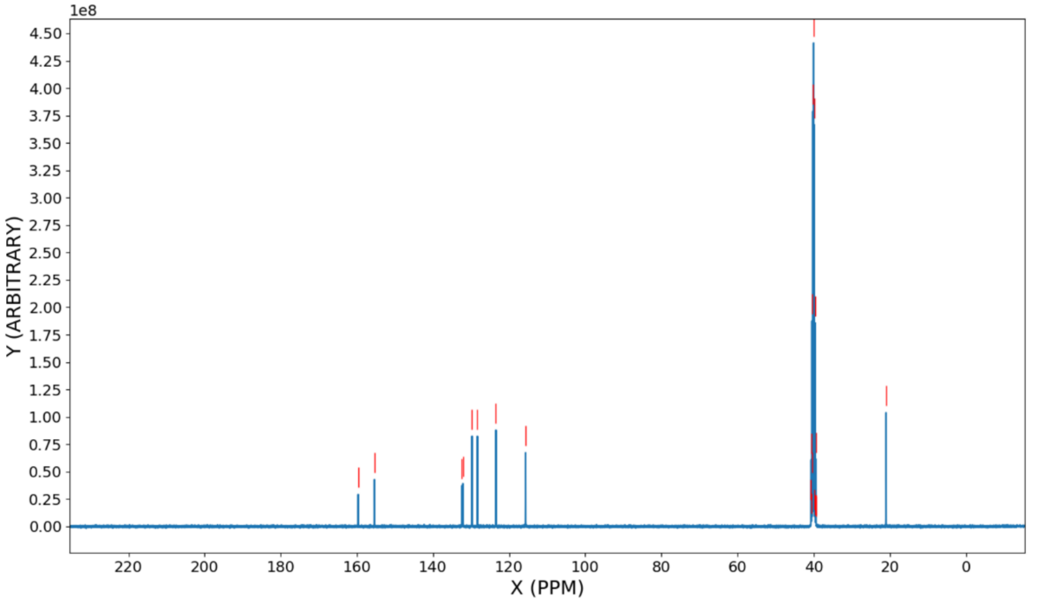

[**S2c**] 3-Propan-2-yl-1*H*-quinoxalin-2-one

CHMO:0000593 | ^{1}H nuclear magnetic resonance spectroscopy (^{1}H NMR)

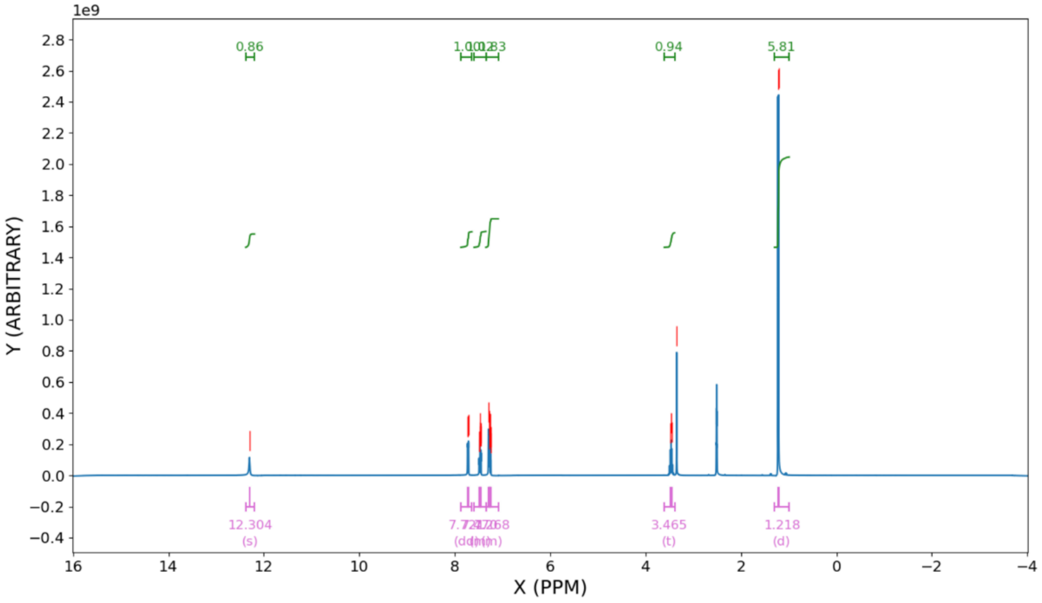

CHMO:0000595 | ^{13}C nuclear magnetic resonance spectroscopy (^{13}C NMR)

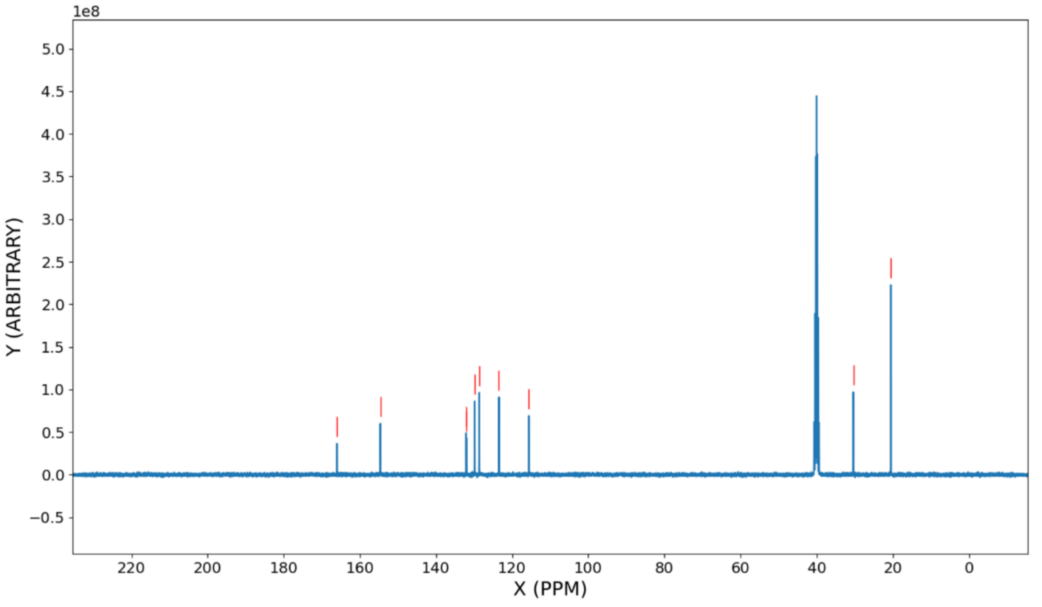

[**S2d**] 3-(Trifluoromethyl)-1*H*-quinoxalin-2-one

CHMO:0000593 | ^{1}H nuclear magnetic resonance spectroscopy (^{1}H NMR)

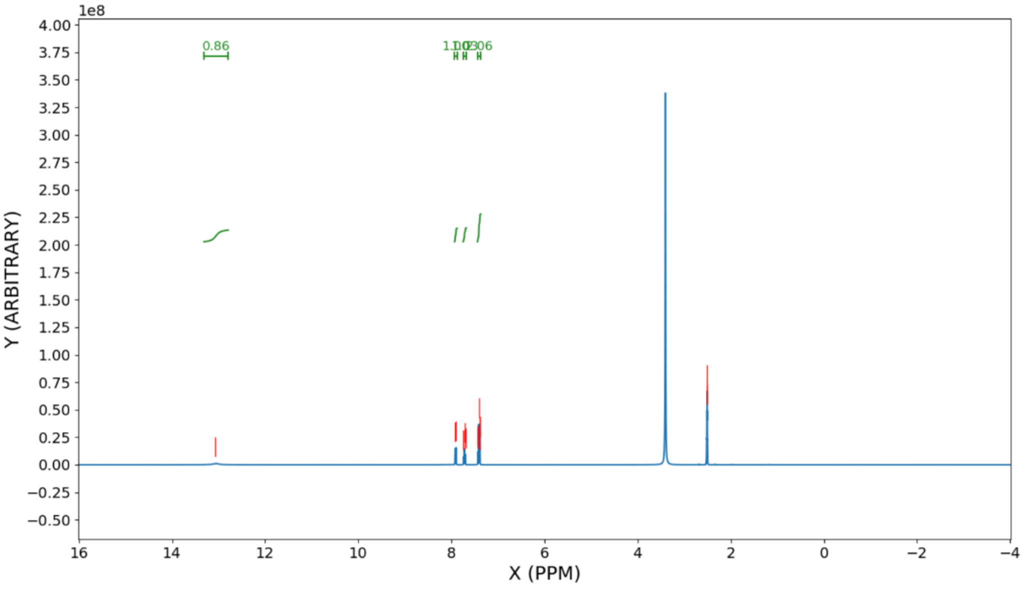

CHMO:0000595 | ^{13}C nuclear magnetic resonance spectroscopy (^{13}C NMR)

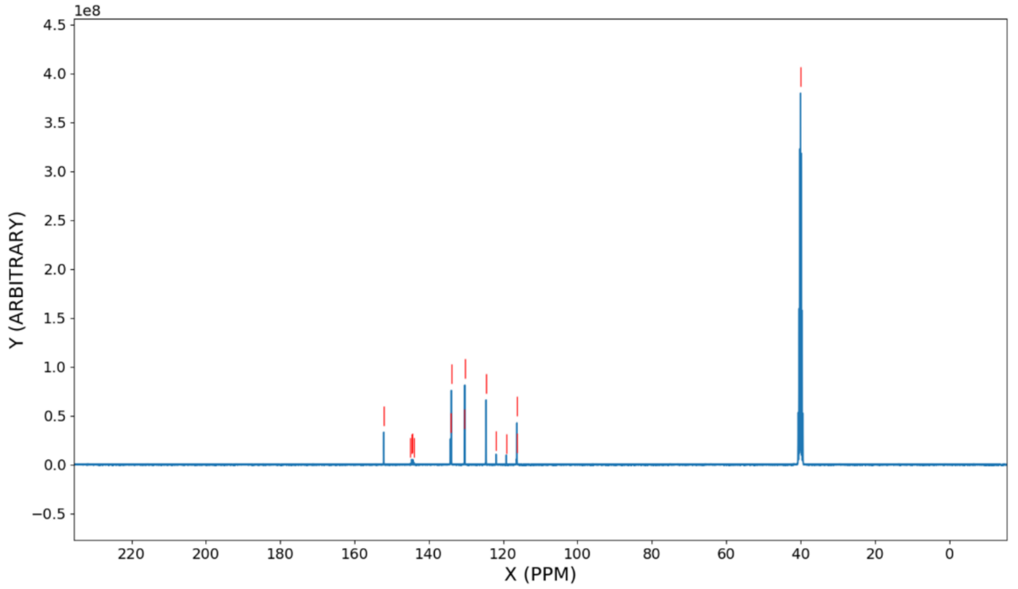

CHMO:0000597 | ^{19}F nuclear magnetic resonance spectroscopy (^{19}F NMR)

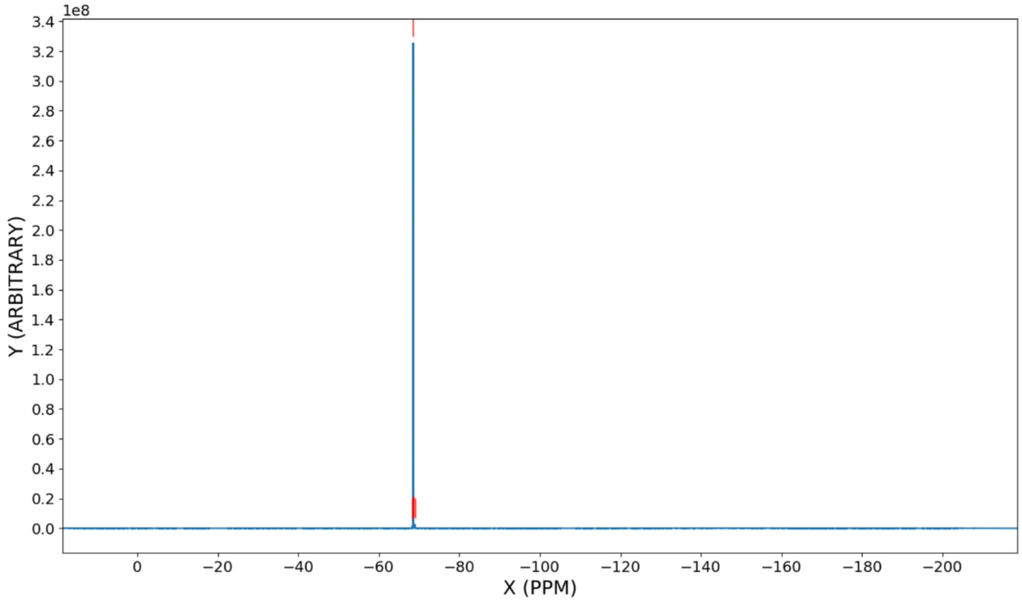

[**S2e**] 3-Phenyl-1*H*-quinoxalin-2-one

CHMO:0000593 | ^{1}H nuclear magnetic resonance spectroscopy (^{1}H NMR)

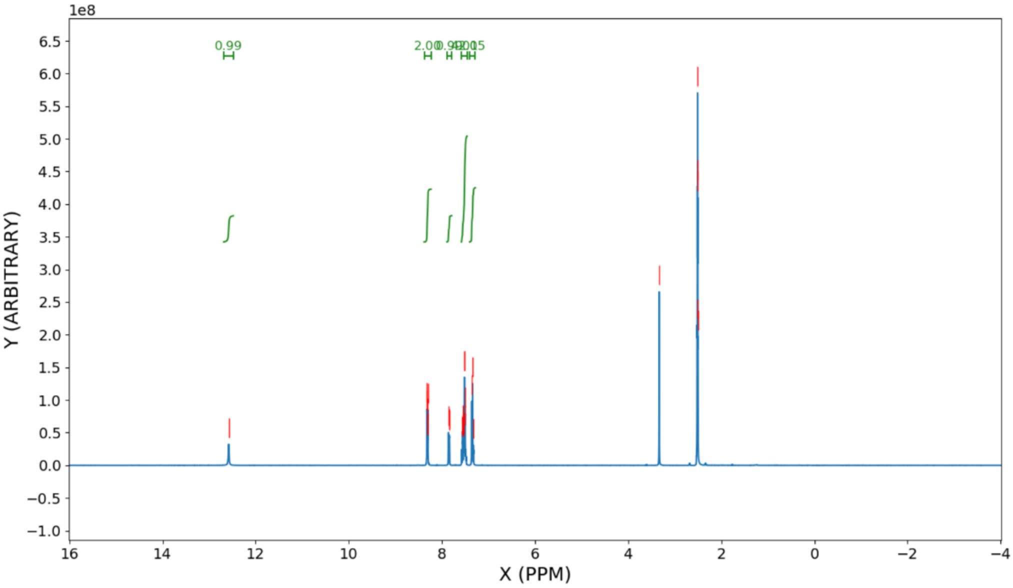

CHMO:0000595 | ^{13}C nuclear magnetic resonance spectroscopy (^{13}C NMR)

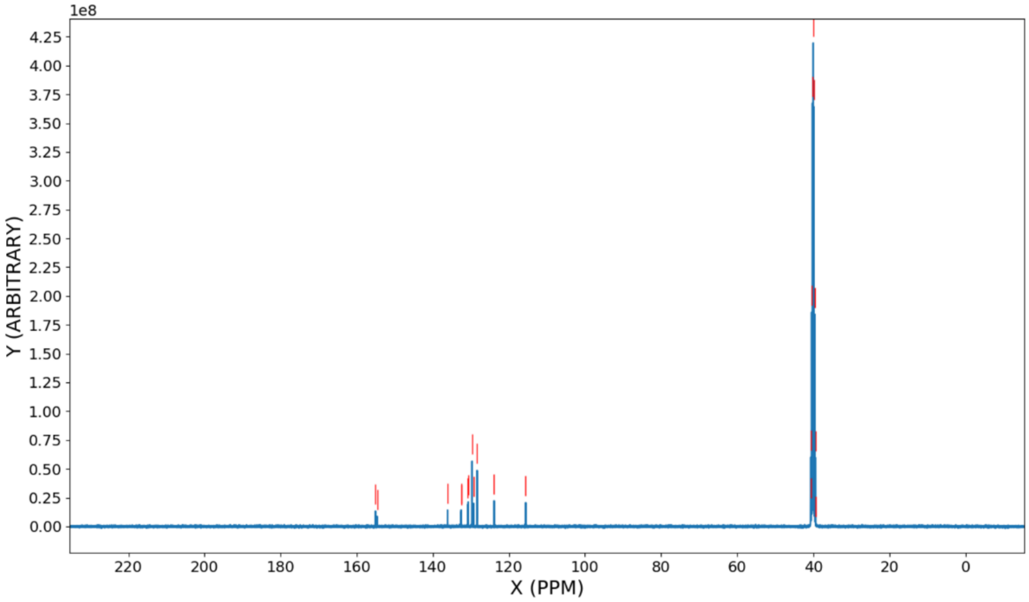

[**S1**] 1,4-Dihydroquinoxaline-2,3-dione

CHMO:0000593 | ^{1}H nuclear magnetic resonance spectroscopy (^{1}H NMR)

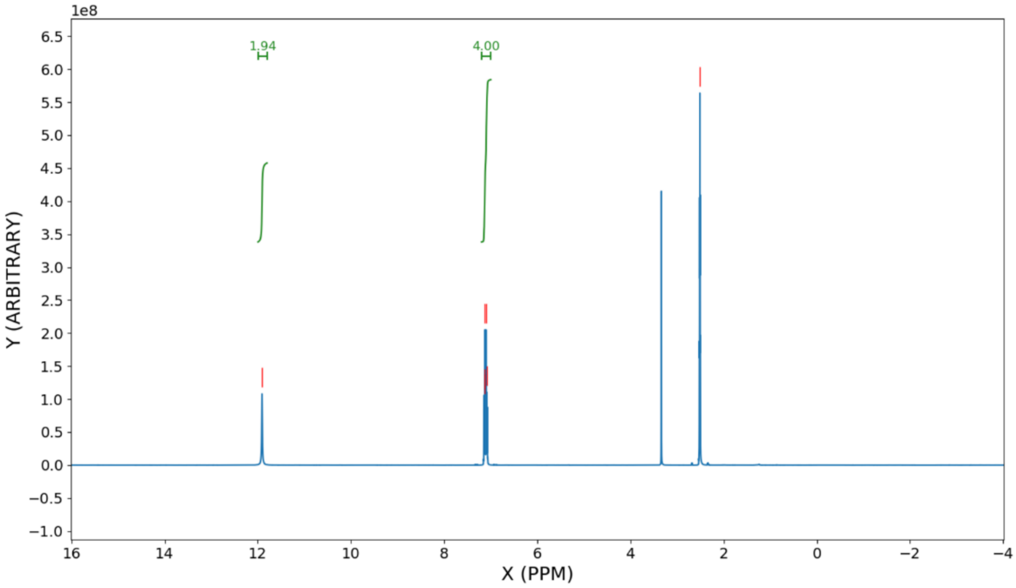

CHMO:0000595 | ^{13}C nuclear magnetic resonance spectroscopy (^{13}C NMR)

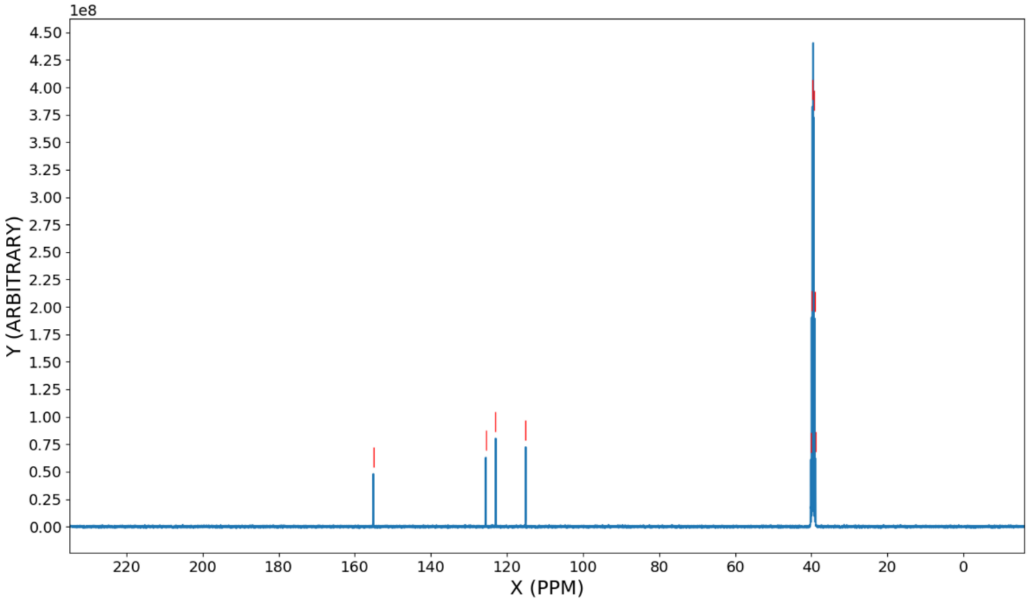

[**10a**] 2-Chloroquinoxaline

CHMO:0000593 | ^{1}H nuclear magnetic resonance spectroscopy (^{1}H NMR)

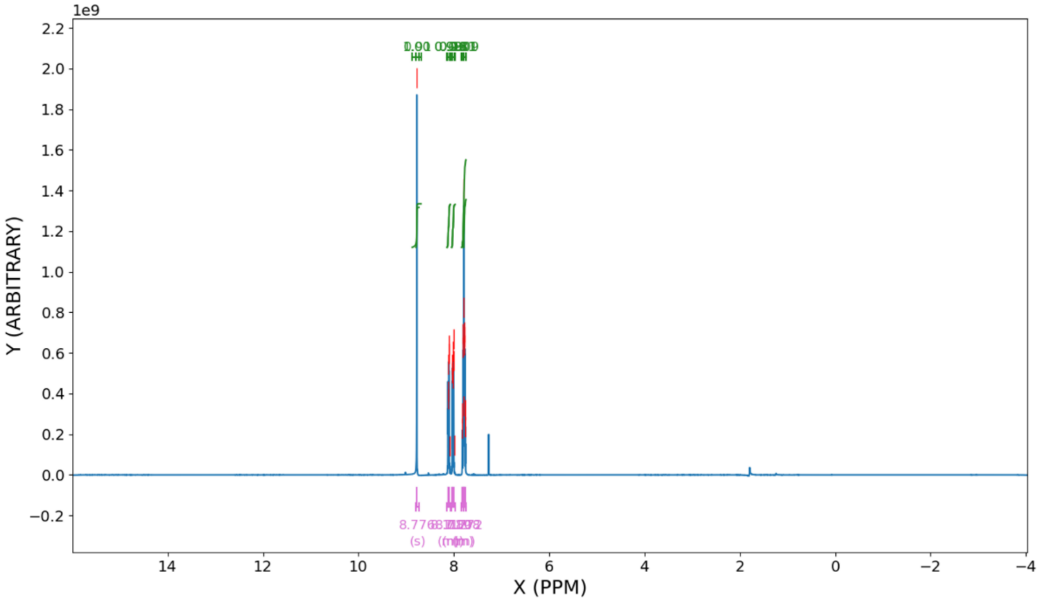

CHMO:0000595 | ^{13}C nuclear magnetic resonance spectroscopy (^{13}C NMR)

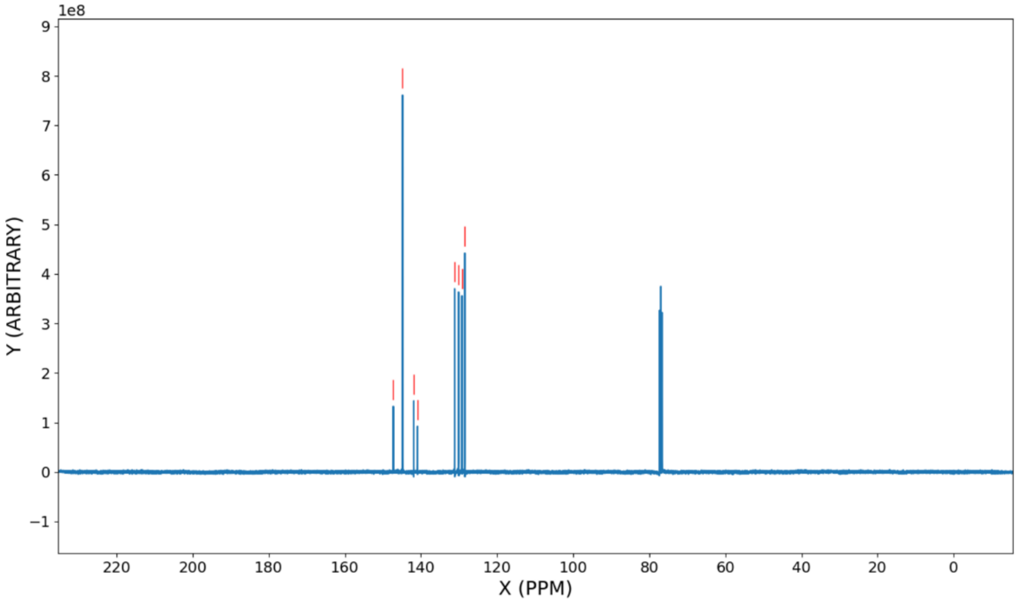

[**10b**] 2-Chloro-3-methylquinoxaline

CHMO:0000593 | ^{1}H nuclear magnetic resonance spectroscopy (^{1}H NMR)

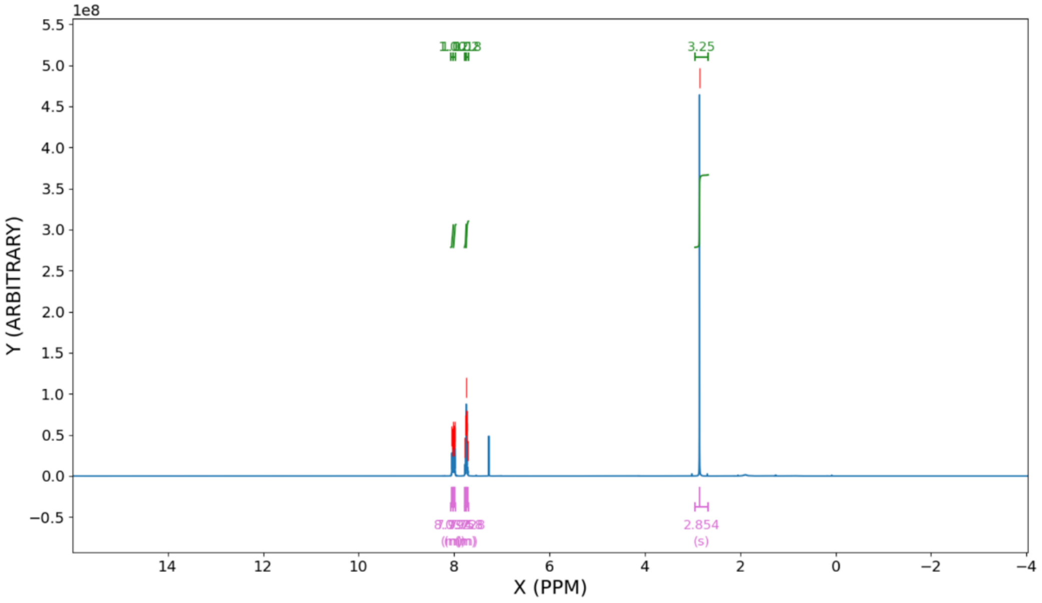

CHMO:0000595 | ^{13}C nuclear magnetic resonance spectroscopy (^{13}C NMR)

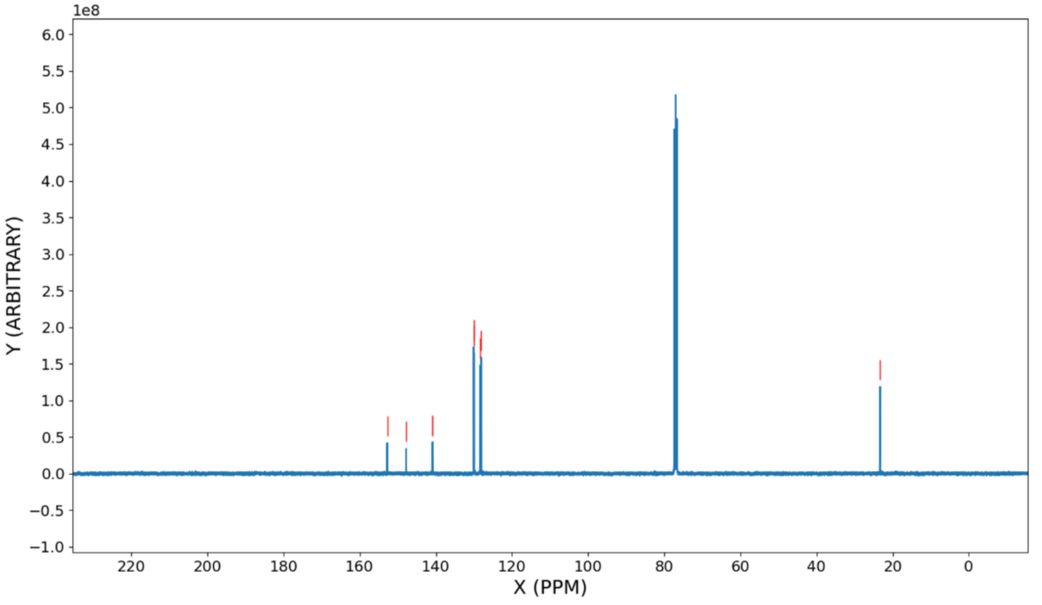

[**10c**] 2-Chloro-3-propan-2-ylquinoxaline

CHMO:0000593 | ^{1}H nuclear magnetic resonance spectroscopy (^{1}H NMR)

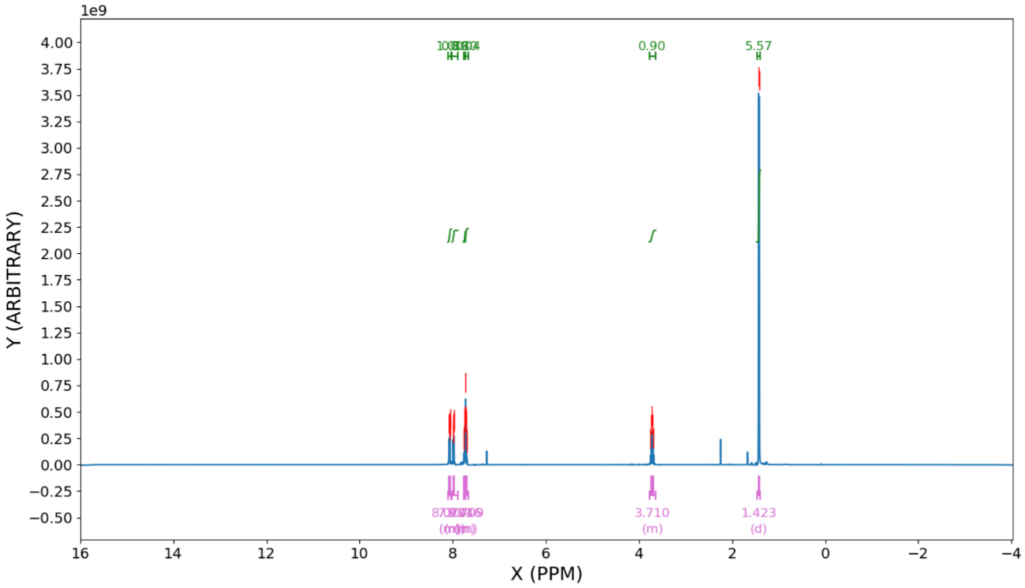

CHMO:0000595 | ^{13}C nuclear magnetic resonance spectroscopy (^{13}C NMR)

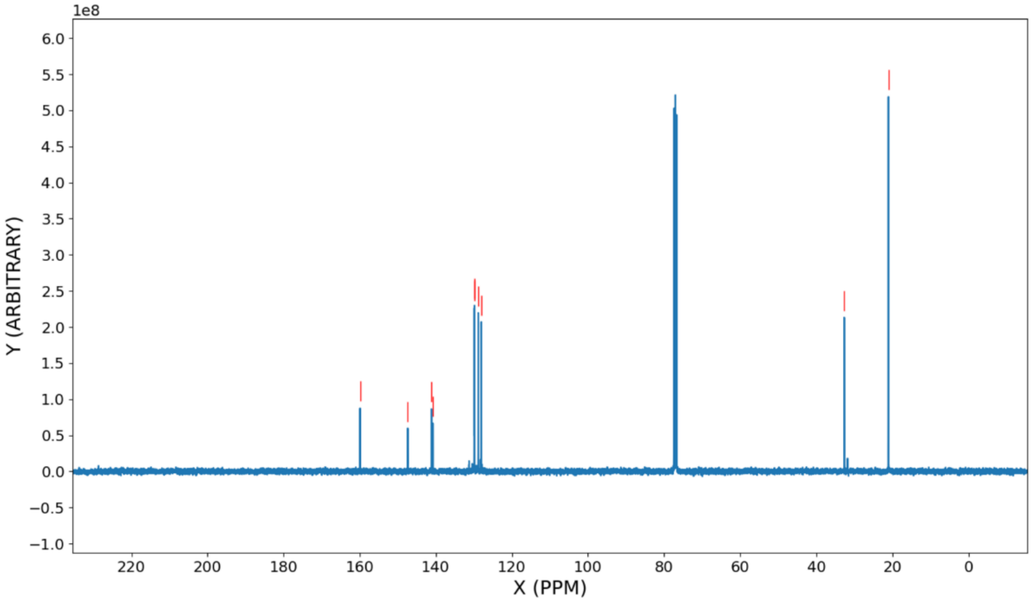

[**10d**] 2-Chloro-3-(trifluoromethyl)quinoxaline

CHMO:0000593 | ^{1}H nuclear magnetic resonance spectroscopy (^{1}H NMR)

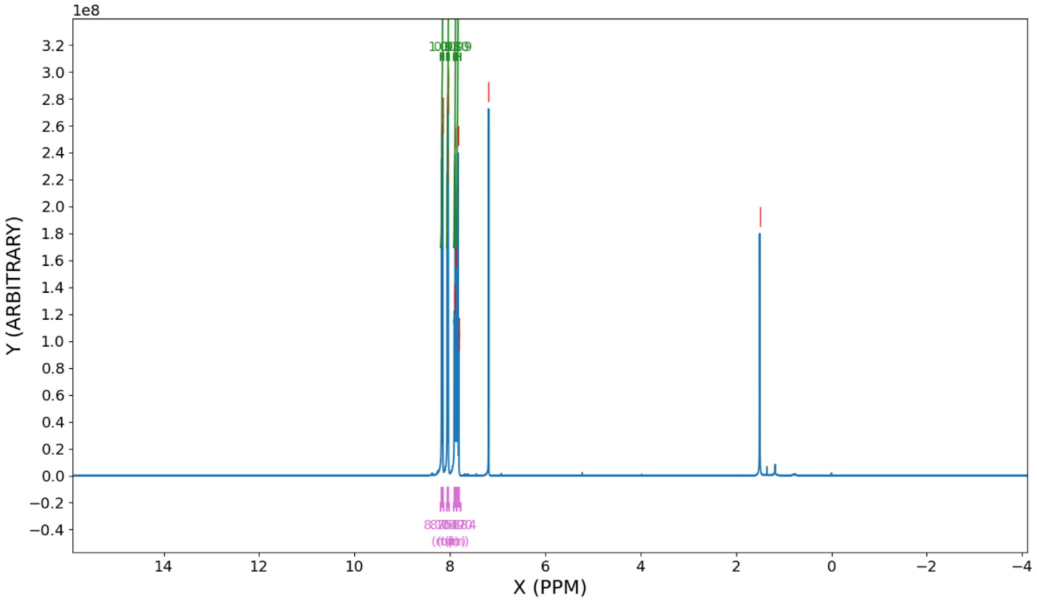

CHMO:0000595 | ^{13}C nuclear magnetic resonance spectroscopy (^{13}C NMR)

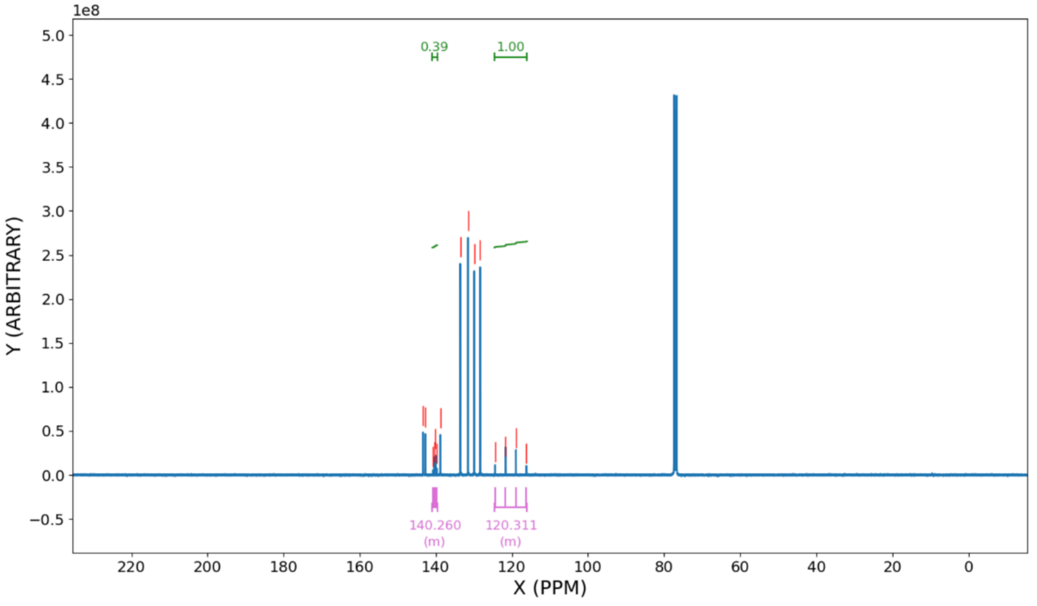

CHMO:0000597 | ^{19}F nuclear magnetic resonance spectroscopy (^{19}F NMR)

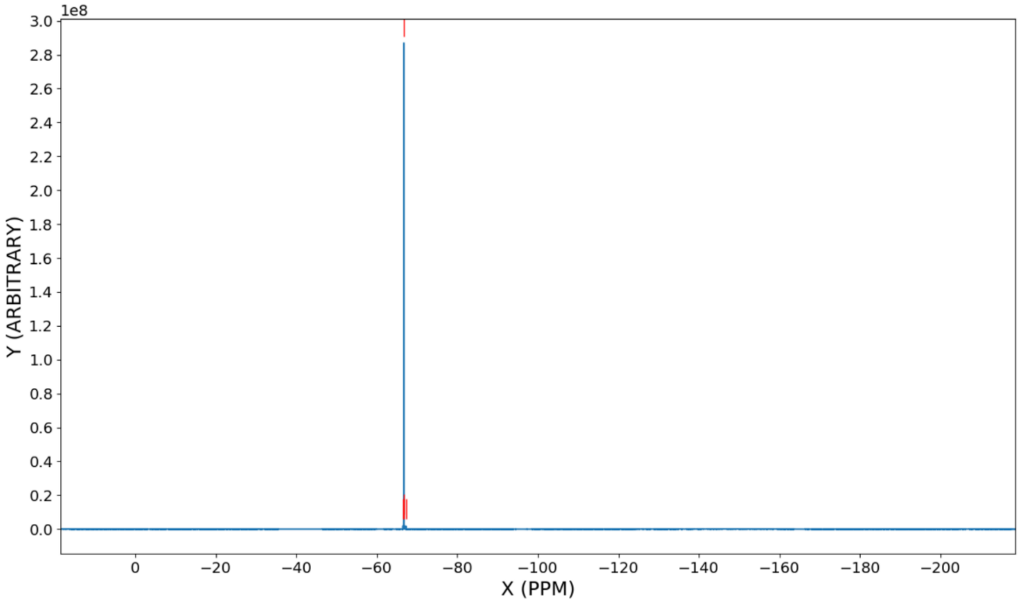

[**10e**] 2-Chloro-3-phenylquinoxaline

CHMO:0000595 | ^{1}H nuclear magnetic resonance spectroscopy (^{1}H NMR)

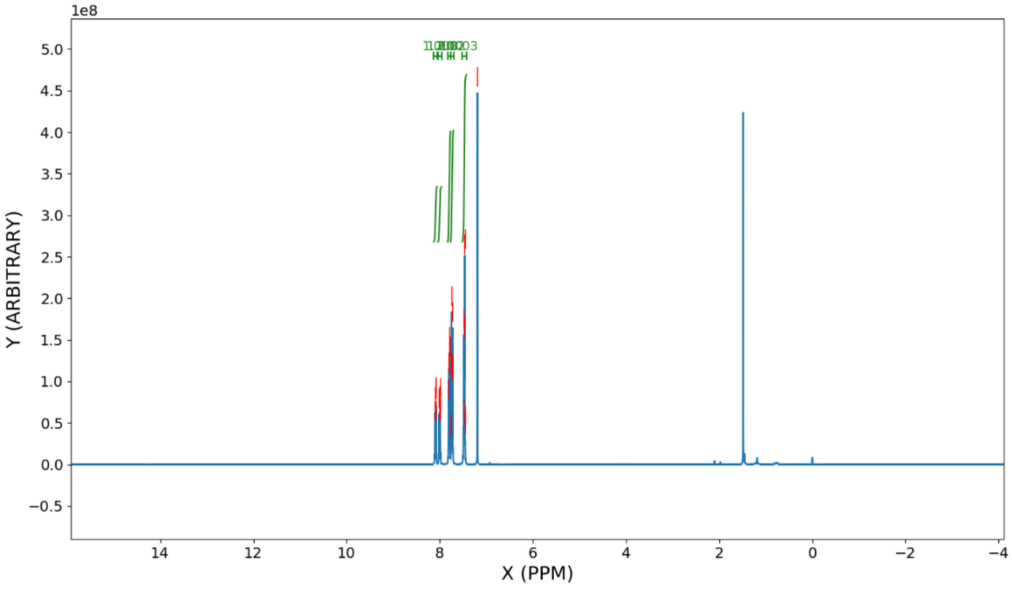

CHMO:0000595 | ^{13}C nuclear magnetic resonance spectroscopy (^{13}C NMR)

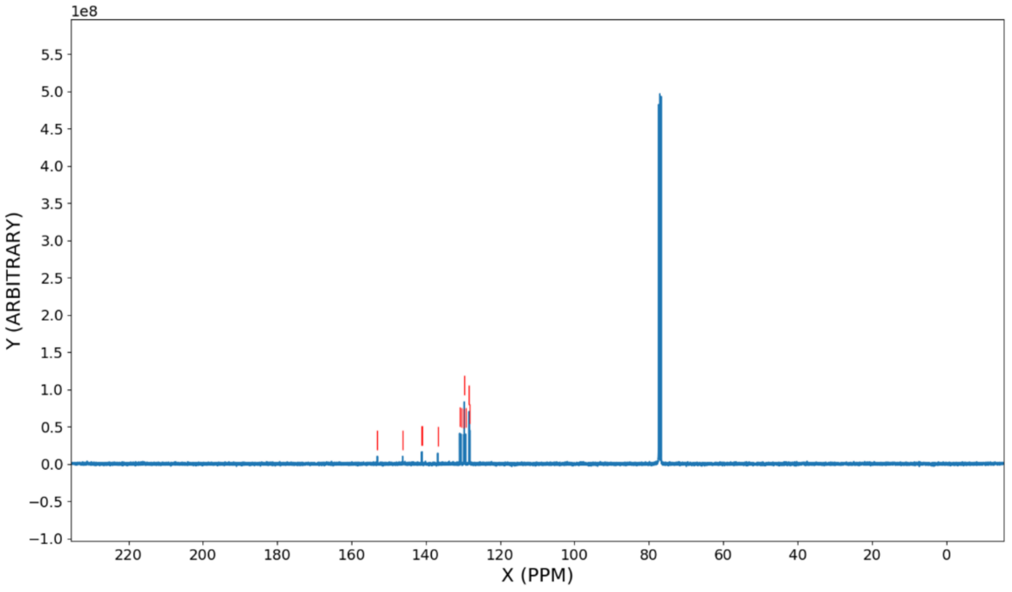

[**10f**] 2,3-Dichloroquinoxaline

CHMO:0000593 | ^{1}H nuclear magnetic resonance spectroscopy (^{1}H NMR)

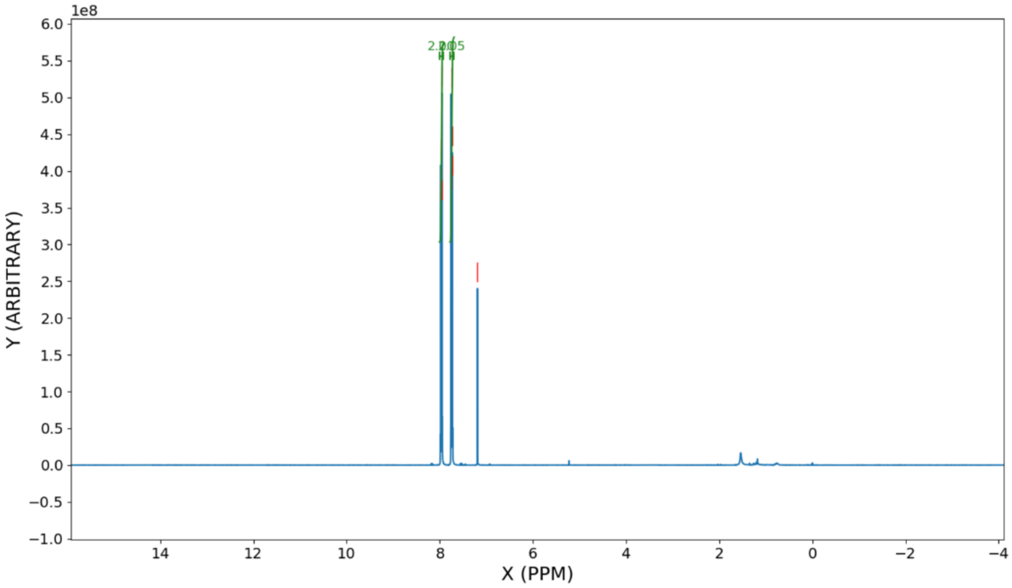

CHMO:0000595 | ^{13}C nuclear magnetic resonance spectroscopy (^{13}C NMR)

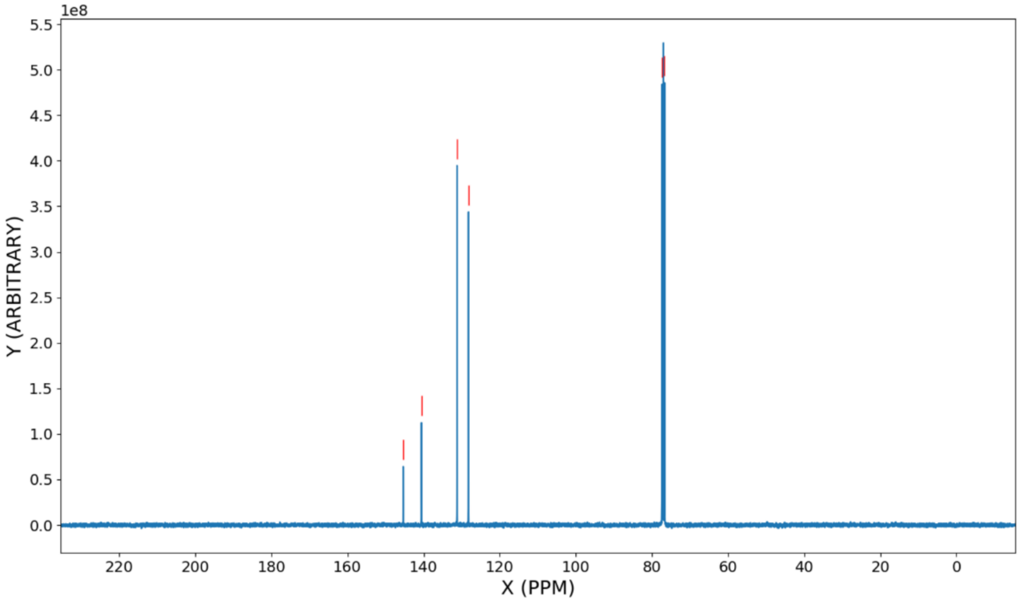

[**11a**] Tetrazolo[1,5-*a*]quinoxaline

CHMO:0000593 | ^{1}H nuclear magnetic resonance spectroscopy (^{1}H NMR)

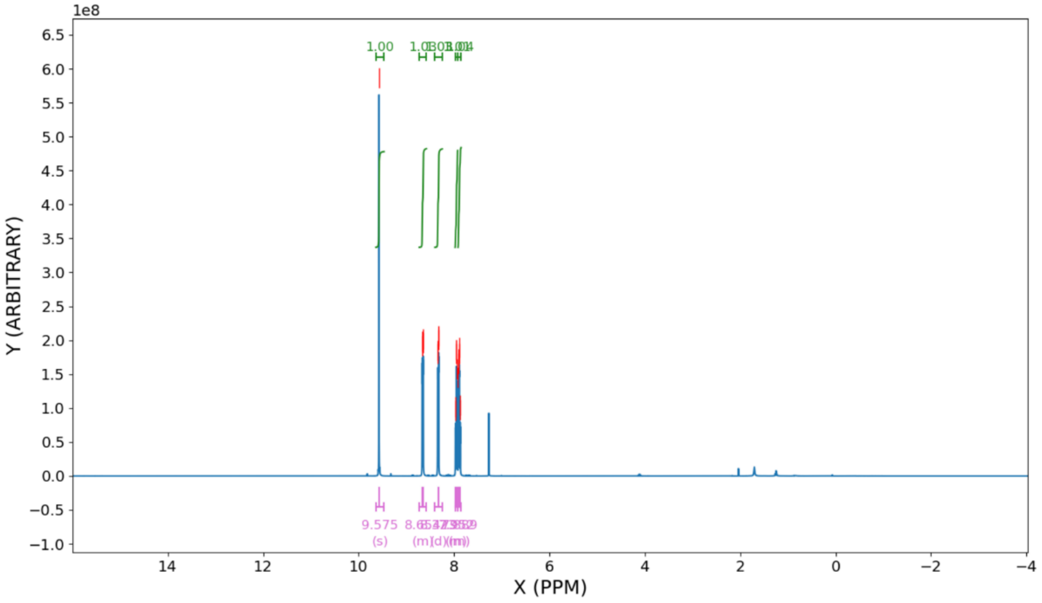

CHMO:0000595 | ^{13}C nuclear magnetic resonance spectroscopy (^{13}C NMR)

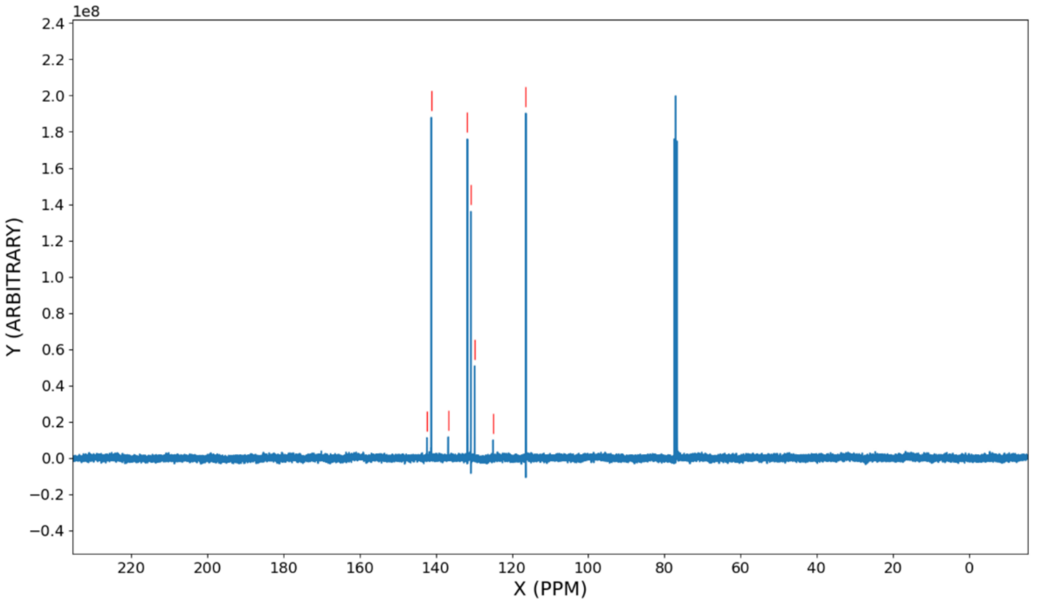

[**11b**] 4-Methyltetrazolo[1,5-*a*]quinoxaline

CHMO:0000593 | ^{1}H nuclear magnetic resonance spectroscopy (^{1}H NMR)

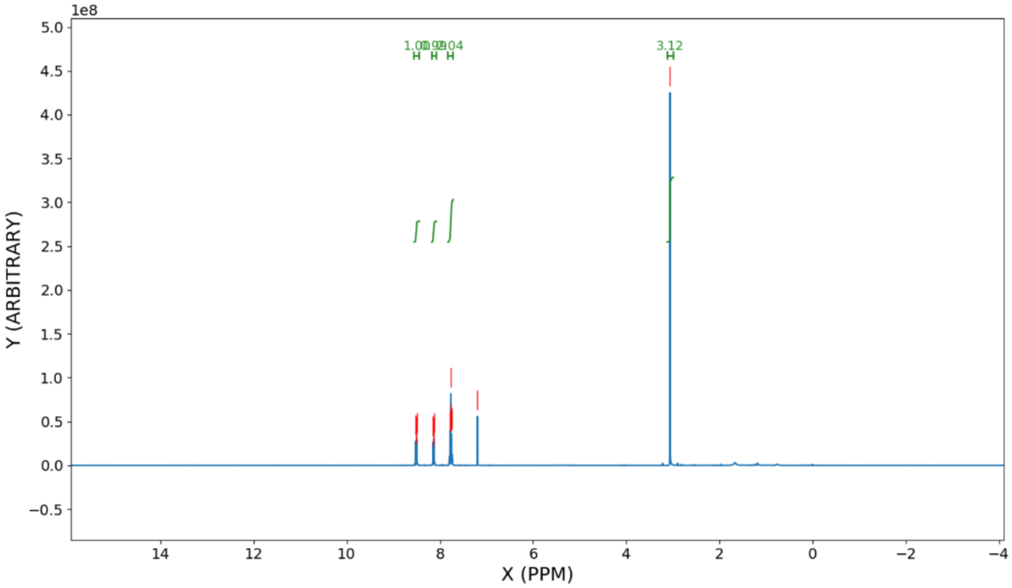

CHMO:0000595 | ^{13}C nuclear magnetic resonance spectroscopy (^{13}C NMR)

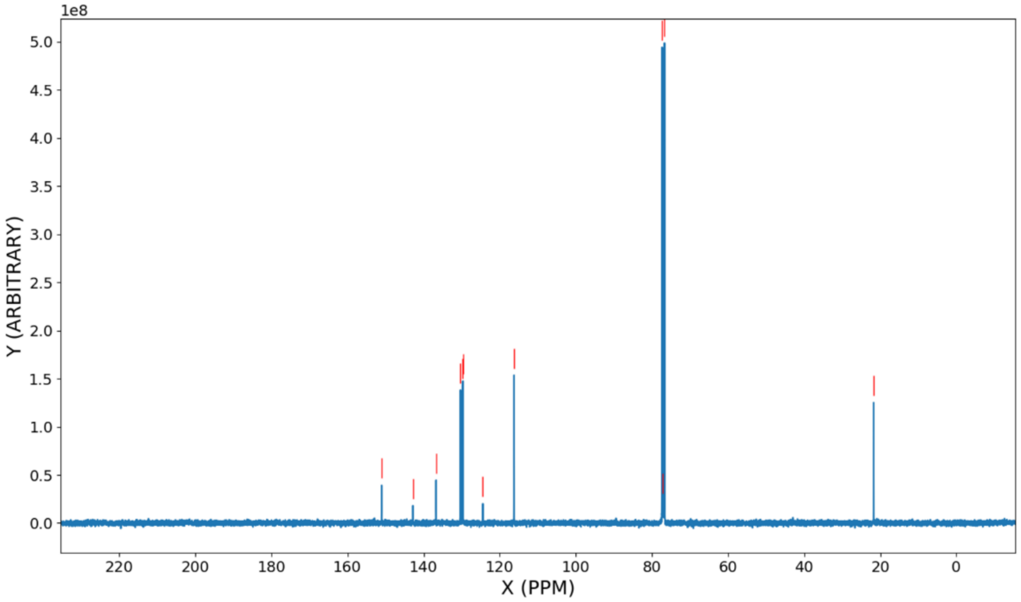

[**11c**] 4-Isopropyltetrazolo[1,5-*a*]quinoxaline

CHMO:0000593 | ^{1}H nuclear magnetic resonance spectroscopy (^{1}H NMR)

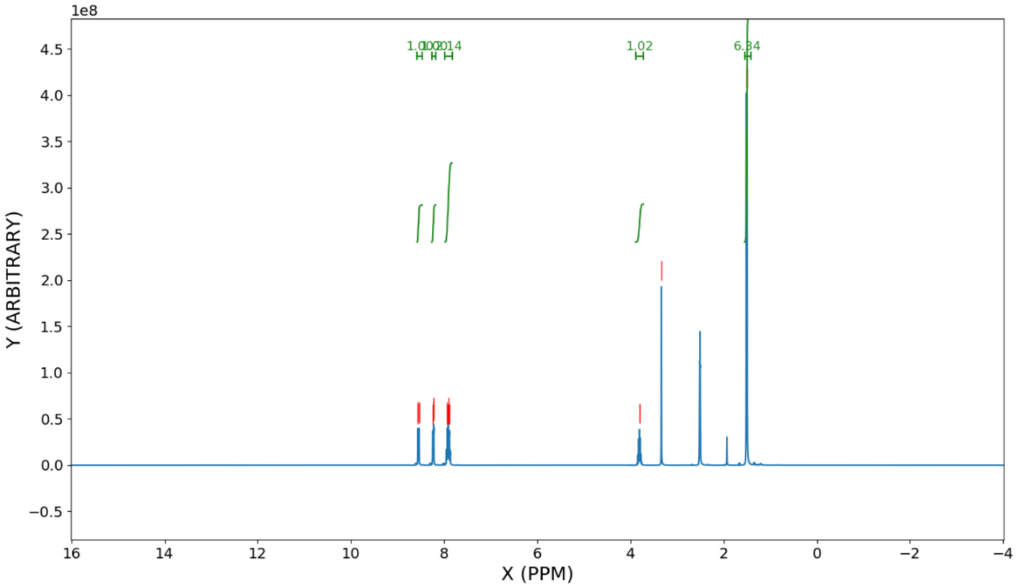

CHMO:0000595 | ^{13}C nuclear magnetic resonance spectroscopy (^{13}C NMR)

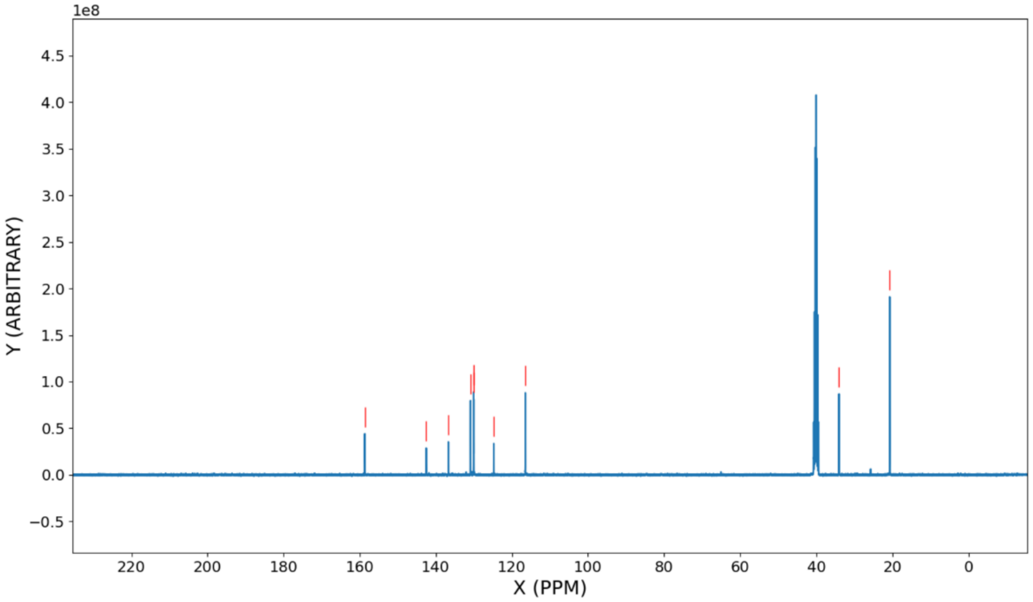

[**11d**] 4-(Trifluoromethyl)tetrazolo[1,5-*a*]quinoxaline

CHMO:0000593 | ^{1}H nuclear magnetic resonance spectroscopy (^{1}H NMR)

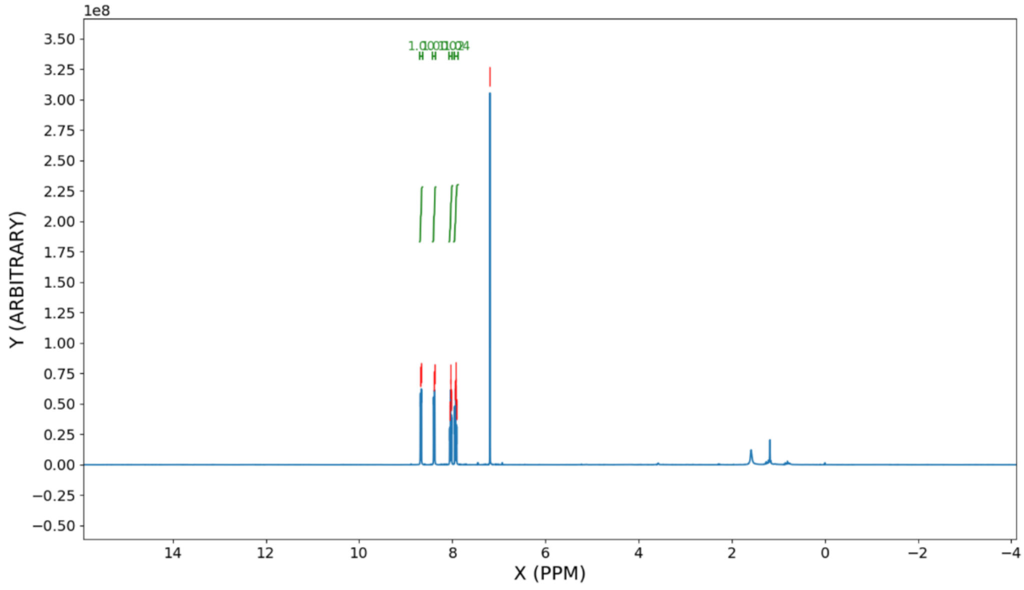

CHMO:0000595 | ^{13}C nuclear magnetic resonance spectroscopy (^{13}C NMR)

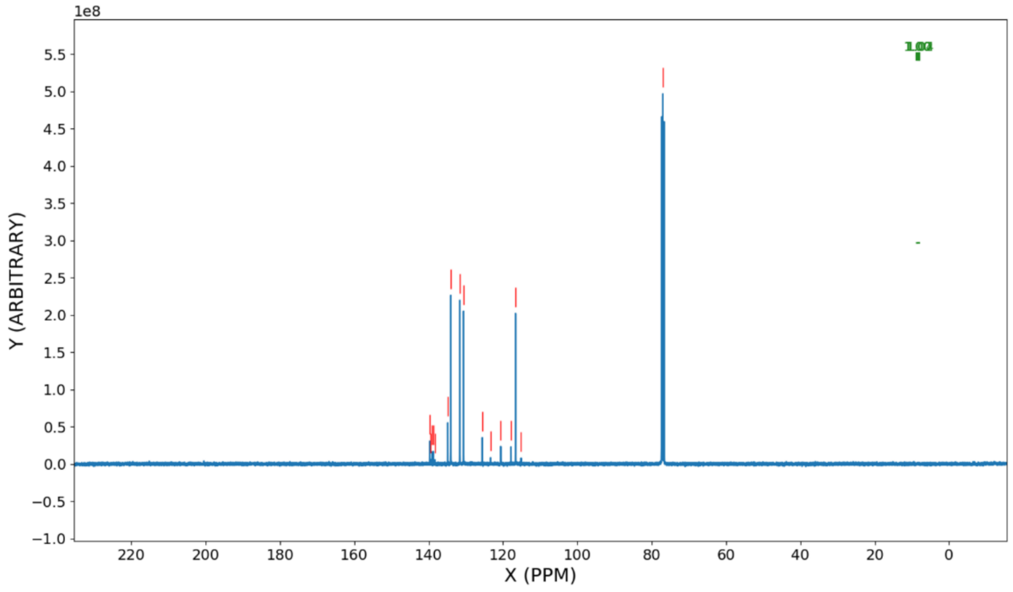

CHMO:0000597 | ^{19}F nuclear magnetic resonance spectroscopy (^{19}F NMR)

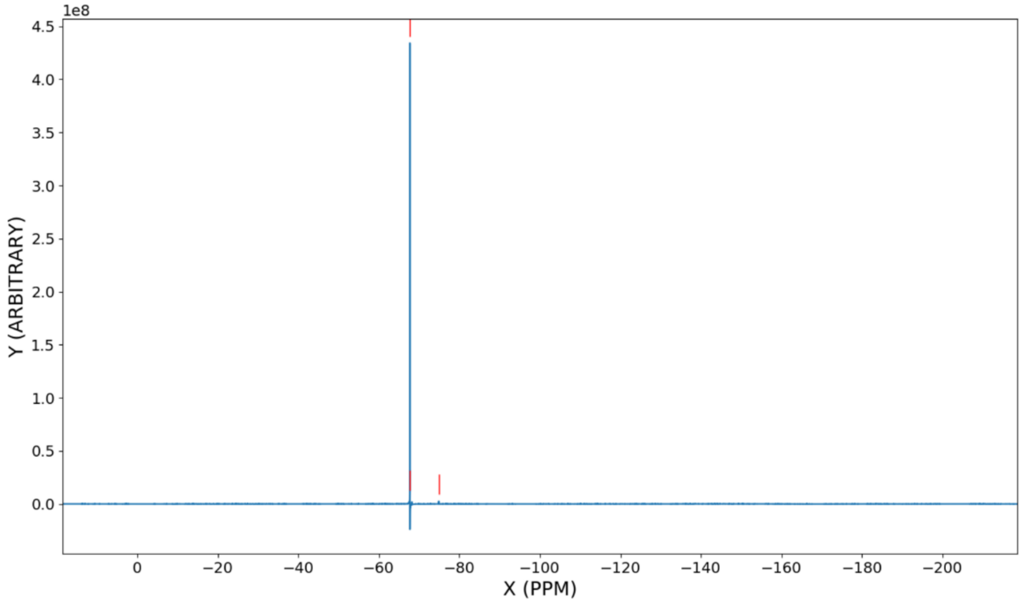

[**11e**] 4-Phenyltetrazolo[1,5-*a*]quinoxaline

CHMO:0000593 | ^{1}H nuclear magnetic resonance spectroscopy (^{1}H NMR)

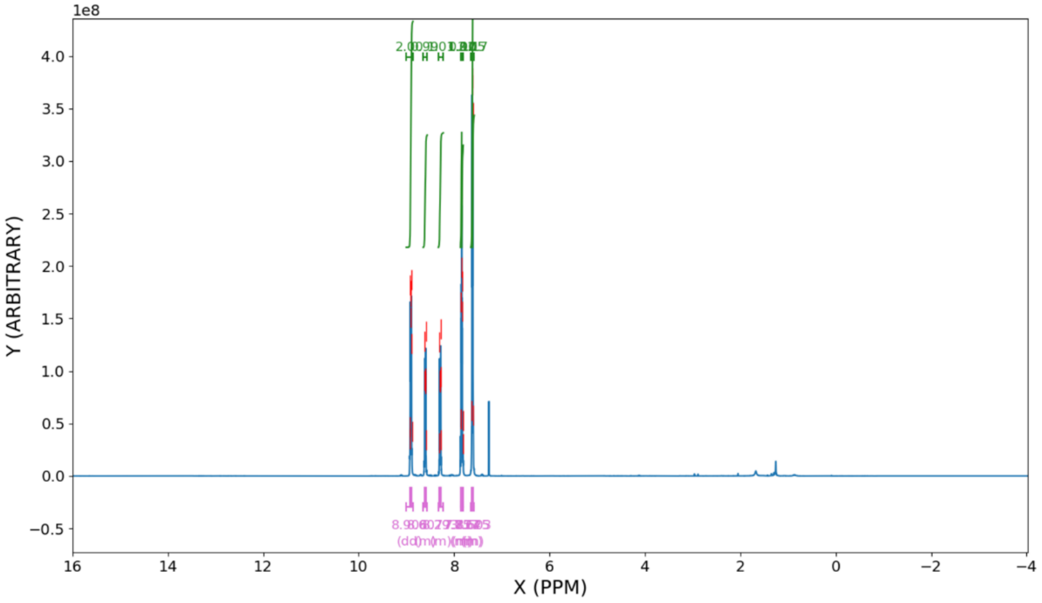

CHMO:0000595 | ^{13}C nuclear magnetic resonance spectroscopy (^{13}C NMR)

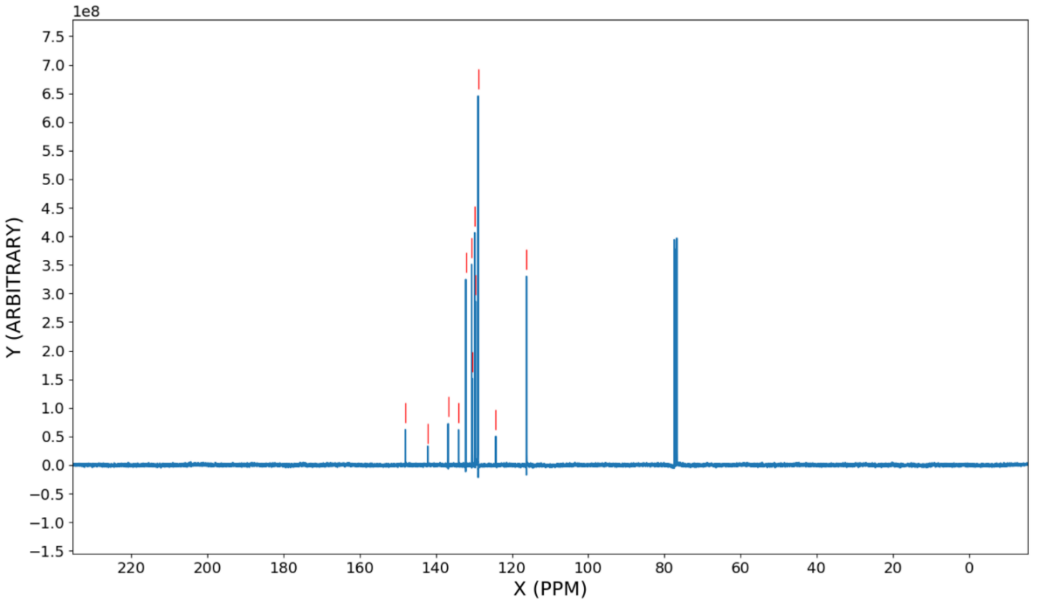

[**11f**] 4-Chlorotetrazolo[1,5-*a*]quinoxaline

CHMO:0000593 | ^{1}H nuclear magnetic resonance spectroscopy (^{1}H NMR)

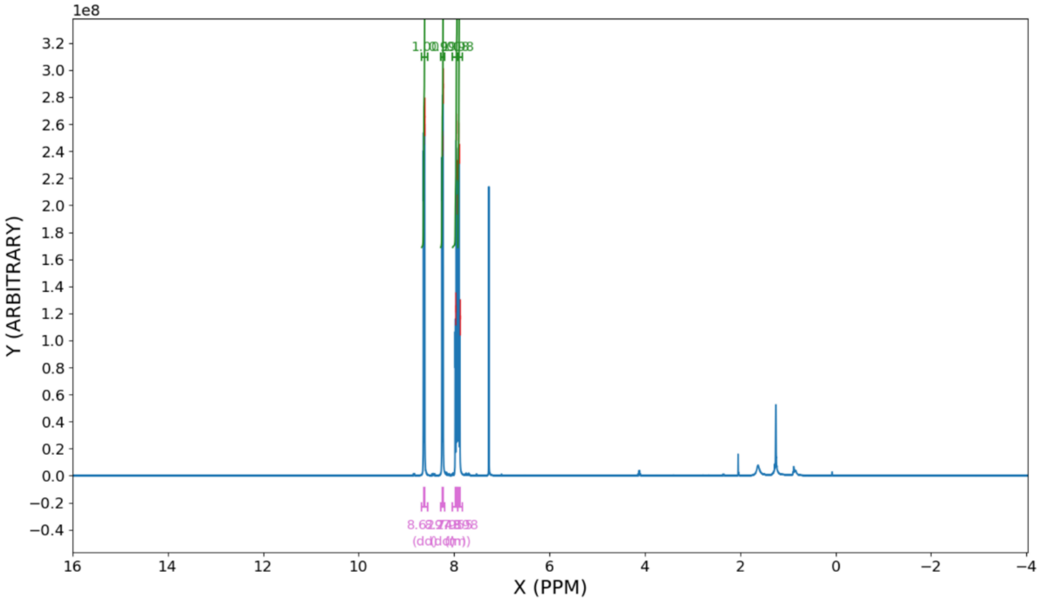

CHMO:0000595 | ^{13}C nuclear magnetic resonance spectroscopy (^{13}C NMR)

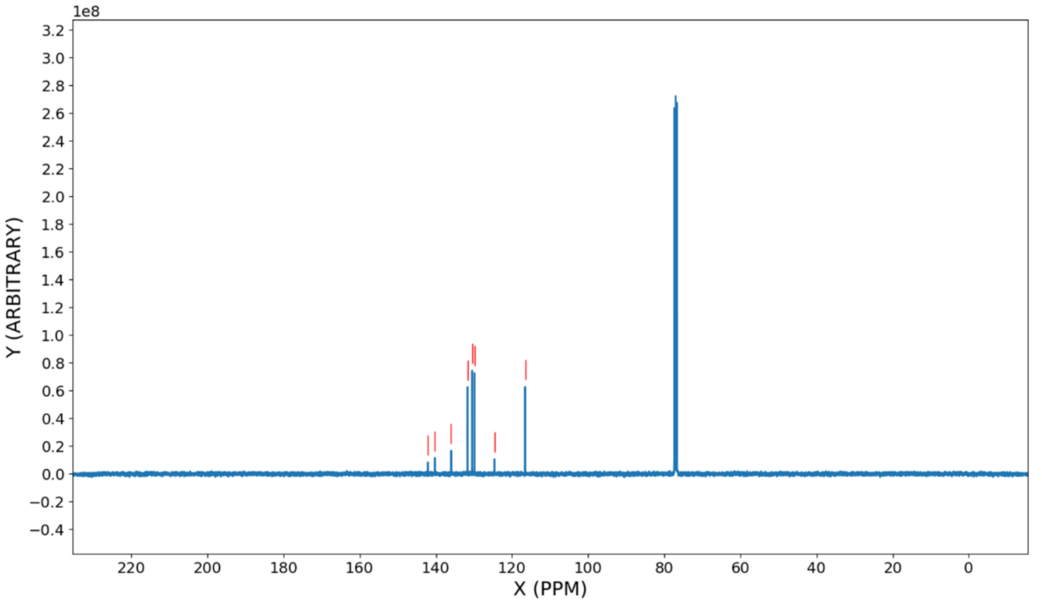

[**11g**] 4-Methoxytetrazolo[1,5-*a*]quinoxaline

CHMO:0000593 | ^{1}H nuclear magnetic resonance spectroscopy (^{1}H NMR)

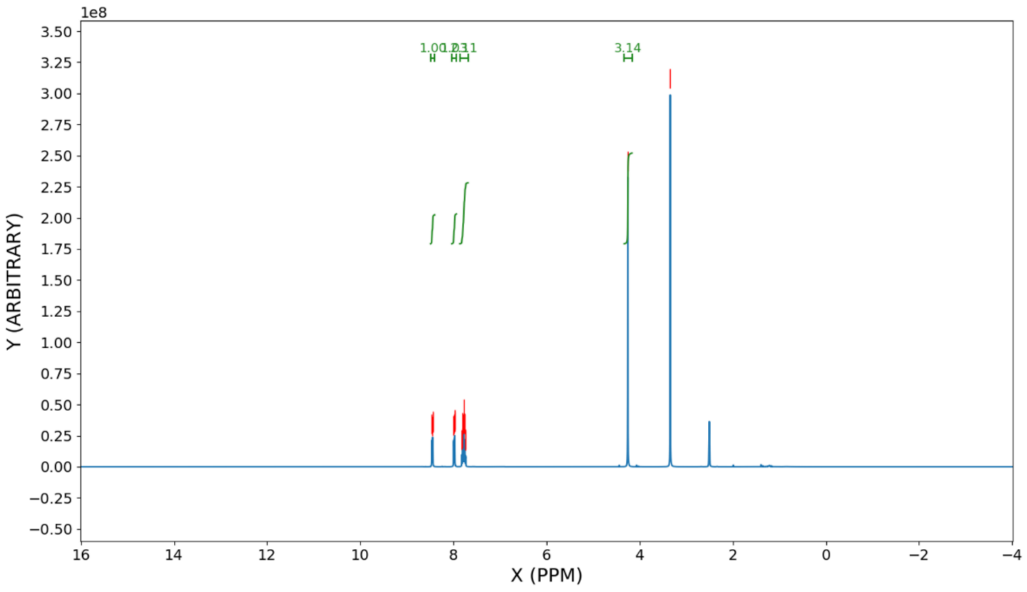

CHMO:0000595 | ^{13}C nuclear magnetic resonance spectroscopy (^{13}C NMR)

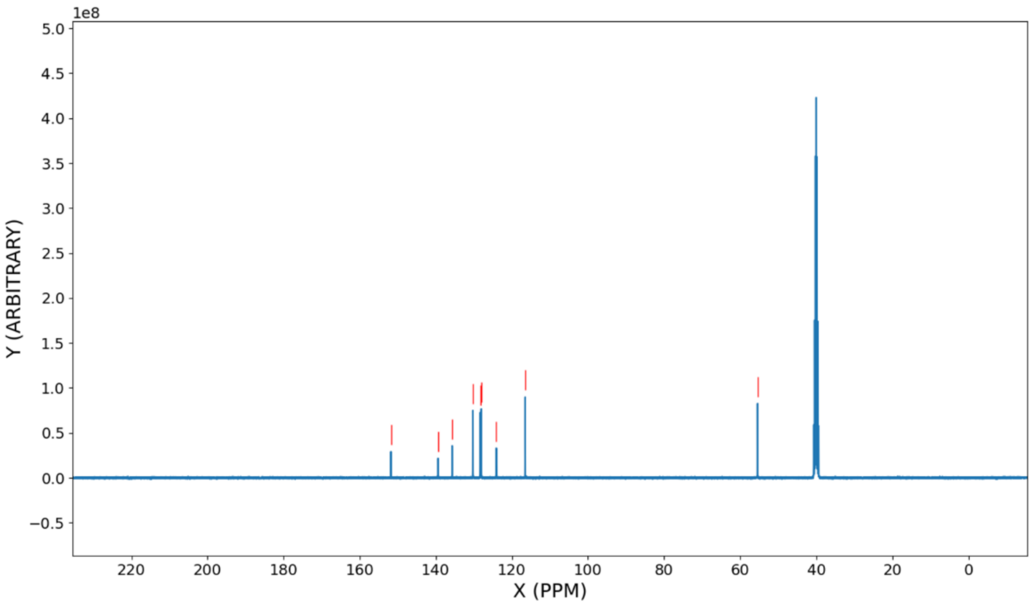

[**11h**] 5*H*-Tetrazolo[1,5-*a*]quinoxalin-4-one

CHMO:0000593 | ^{1}H nuclear magnetic resonance spectroscopy (^{1}H NMR)

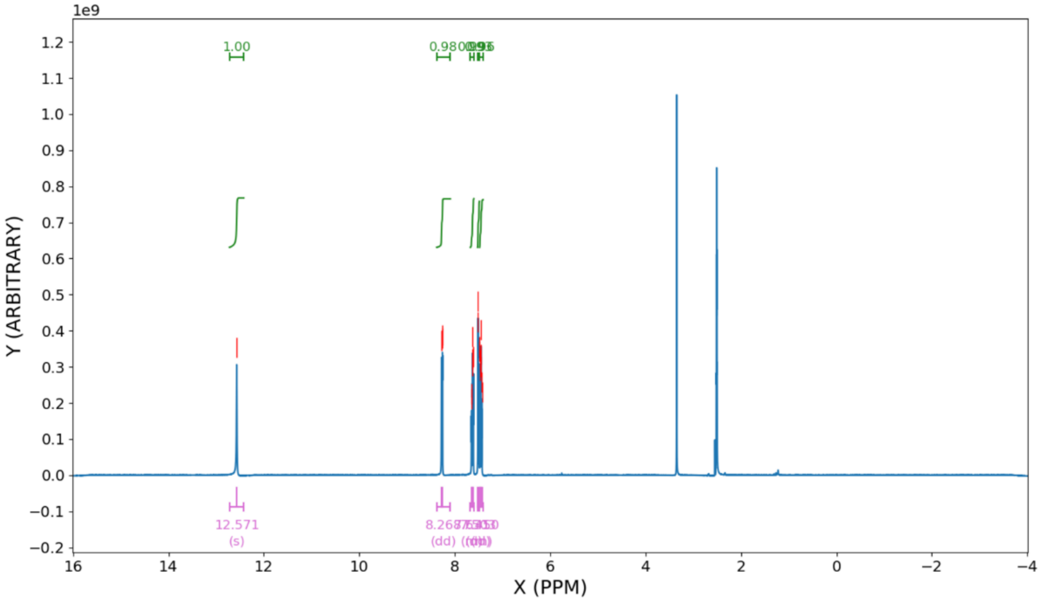

CHMO:0000595 | ^{13}C nuclear magnetic resonance spectroscopy (^{13}C NMR)

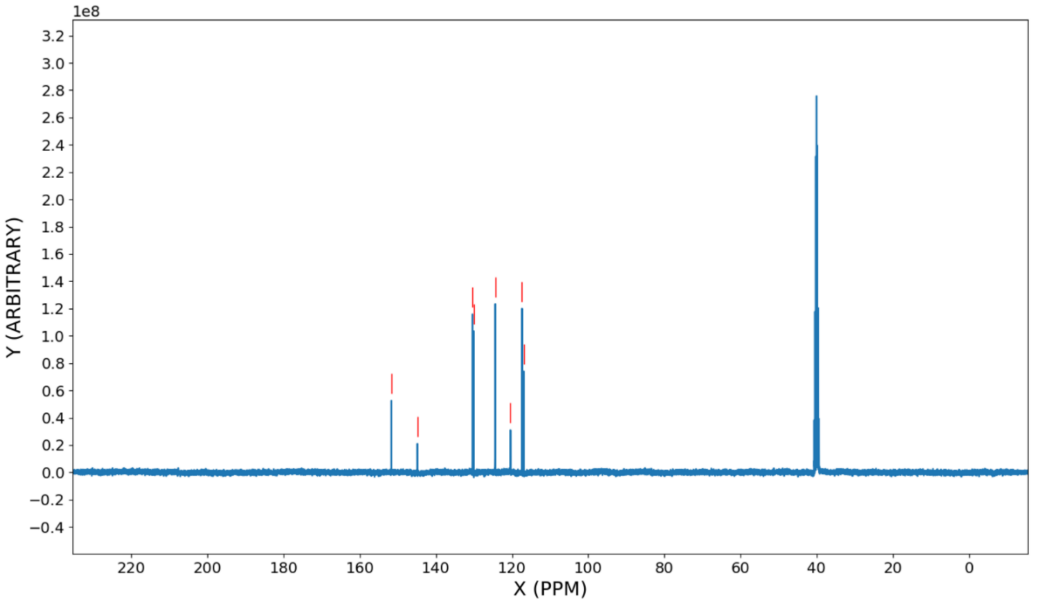

[**11i**] *N*,*N*-Dimethyltetrazolo[1,5-*a*]quinoxalin-4-amine

CHMO:0000593 | ^{1}H nuclear magnetic resonance spectroscopy (^{1}H NMR)

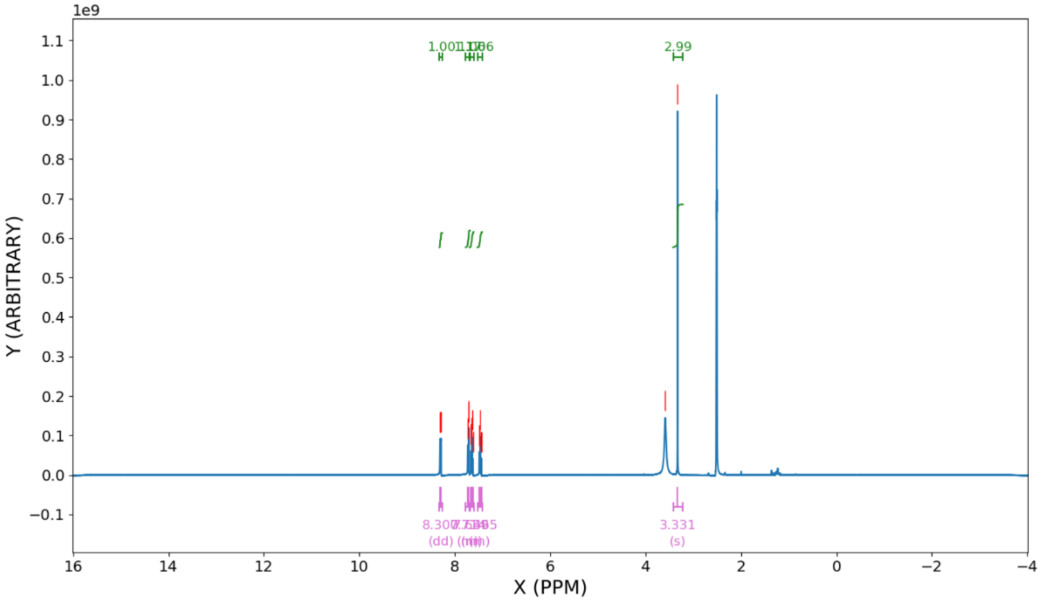

CHMO:0000595 | ^{13}C nuclear magnetic resonance spectroscopy (^{13}C NMR)

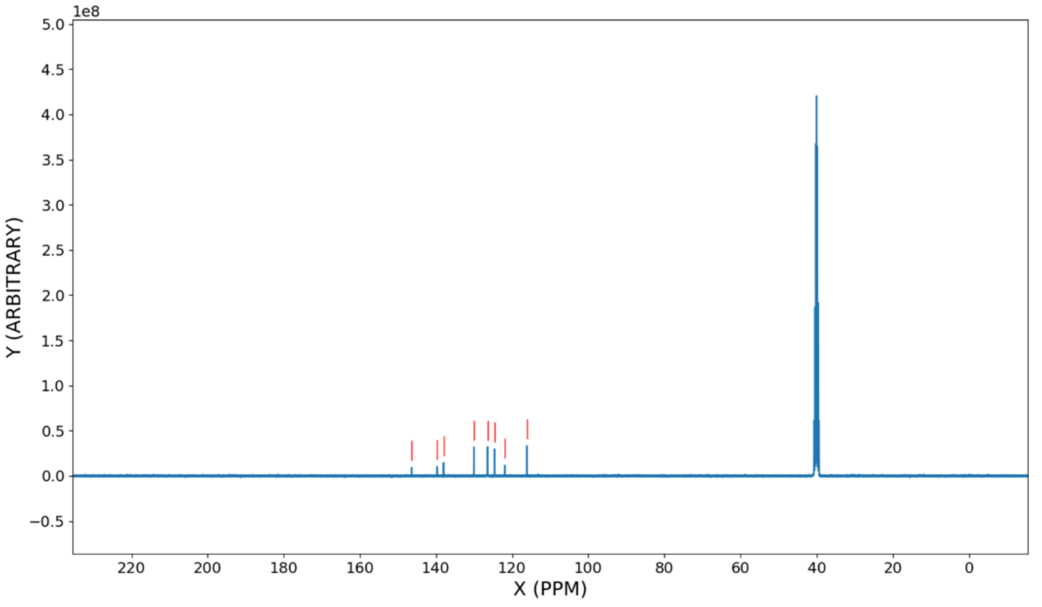

[**11j**] 1-[4-(Tetrazolo[1,5-*a*]quinoxalin-4-ylamino)phenyl]ethanone

CHMO:0000593 | ^{1}H nuclear magnetic resonance spectroscopy (^{1}H NMR)

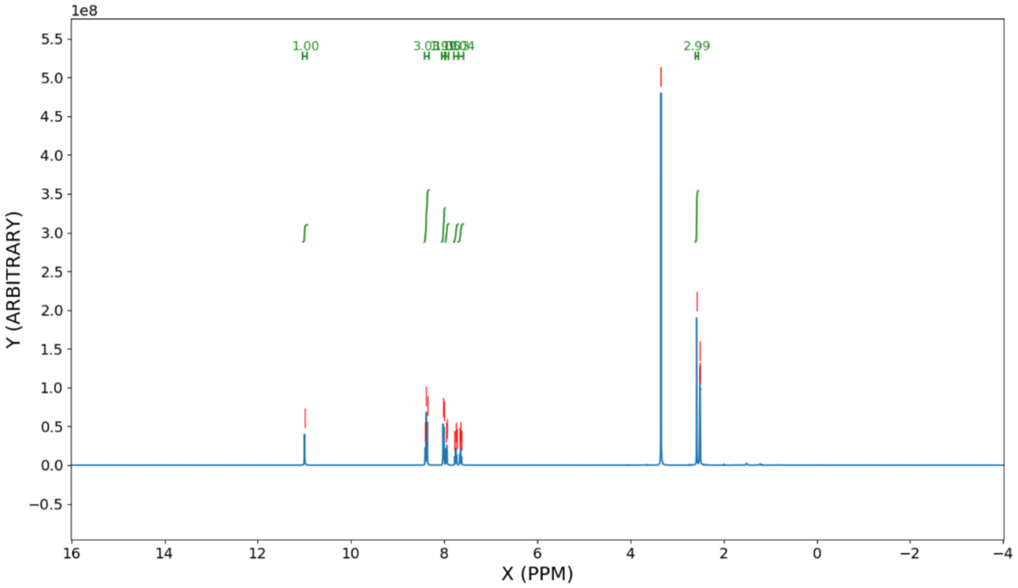

CHMO:0000595 | ^{13}C nuclear magnetic resonance spectroscopy (^{13}C NMR)

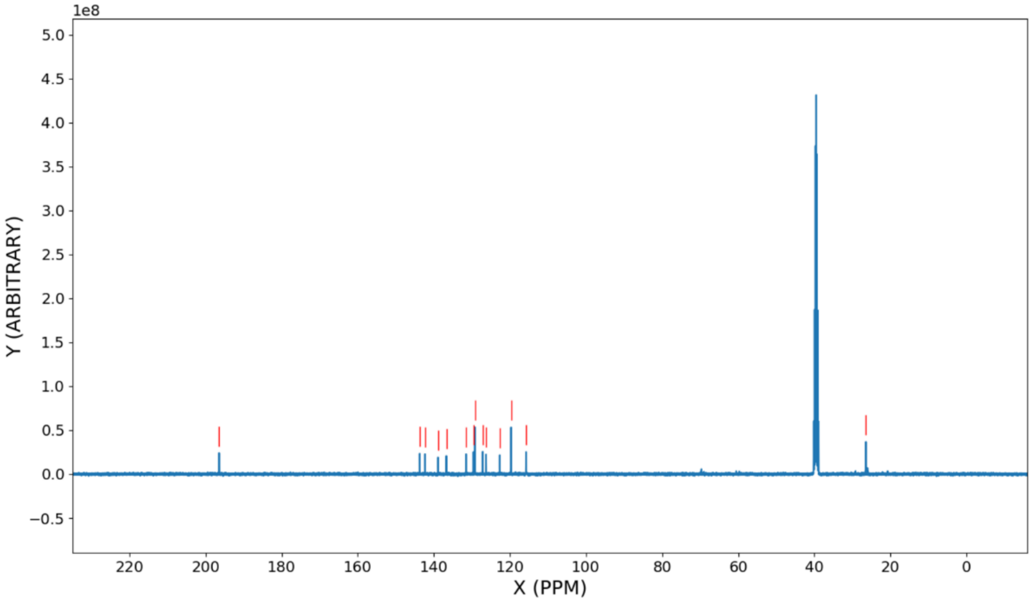

[**11k**] 4-((3,3,4,4,5,5,6,6,7,7,8,8,9,9,10,10,10-Heptadecafluorodecyl)oxy)tetrazolo[1,5-*a*]quinoxaline

CHMO:0000593 | ^{1}H nuclear magnetic resonance spectroscopy (^{1}H NMR)

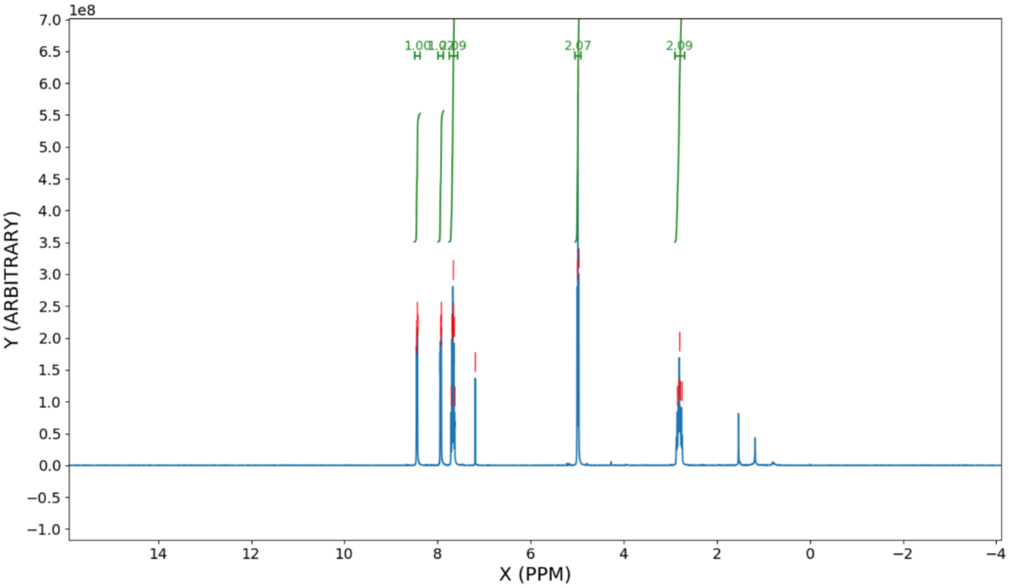

CHMO:0000595 | ^{13}C nuclear magnetic resonance spectroscopy (^{13}C NMR)

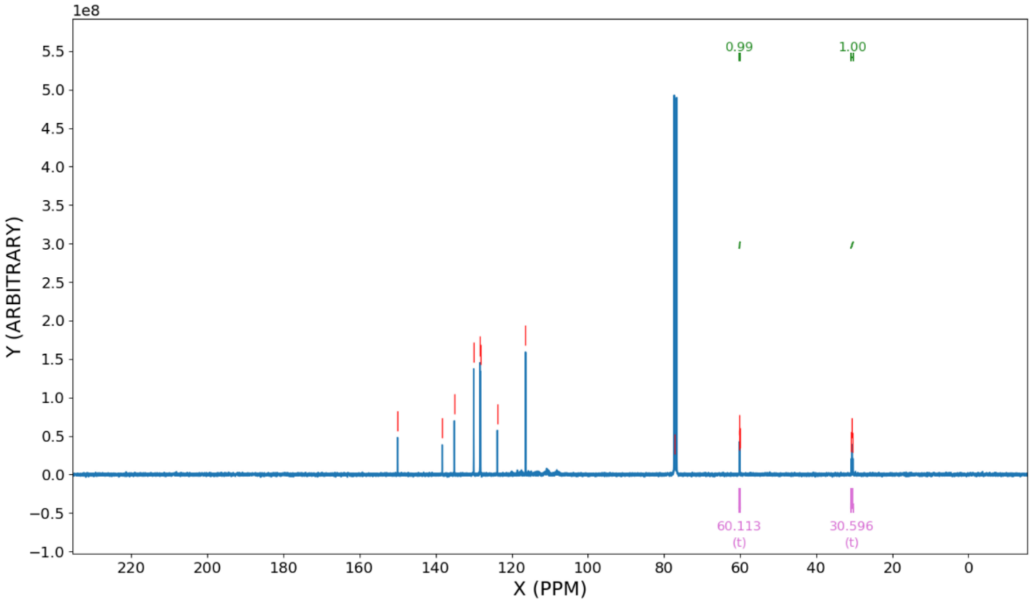

CHMO:0000597 | ^{19}F nuclear magnetic resonance spectroscopy (^{19}F NMR)

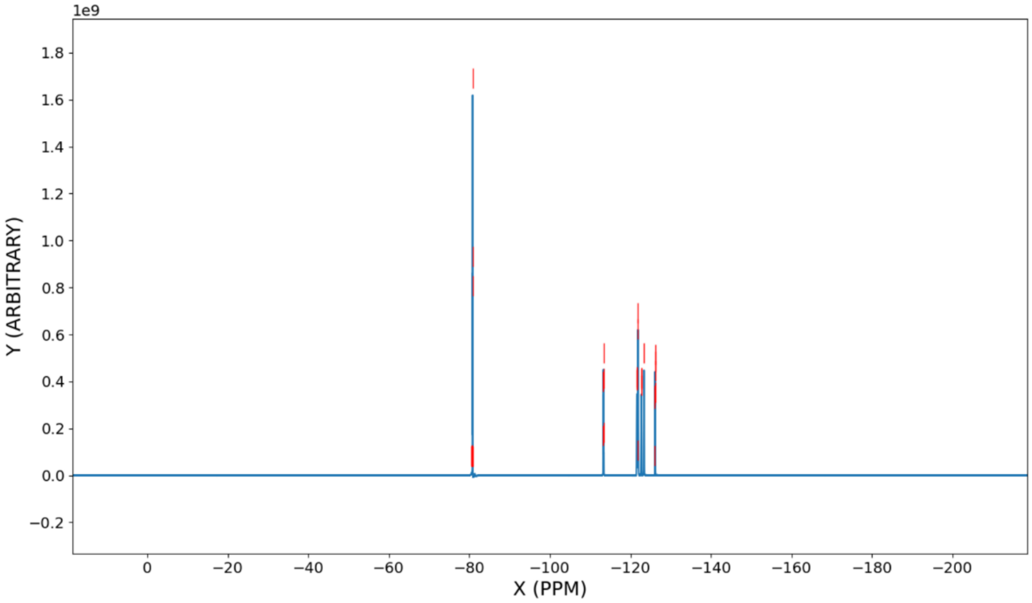

[**11l**] 4-((Trimethylsilyl)ethynyl)tetrazolo[1,5-*a*]quinoxaline

CHMO:0000593 | ^{1}H nuclear magnetic resonance spectroscopy (^{1}H NMR)

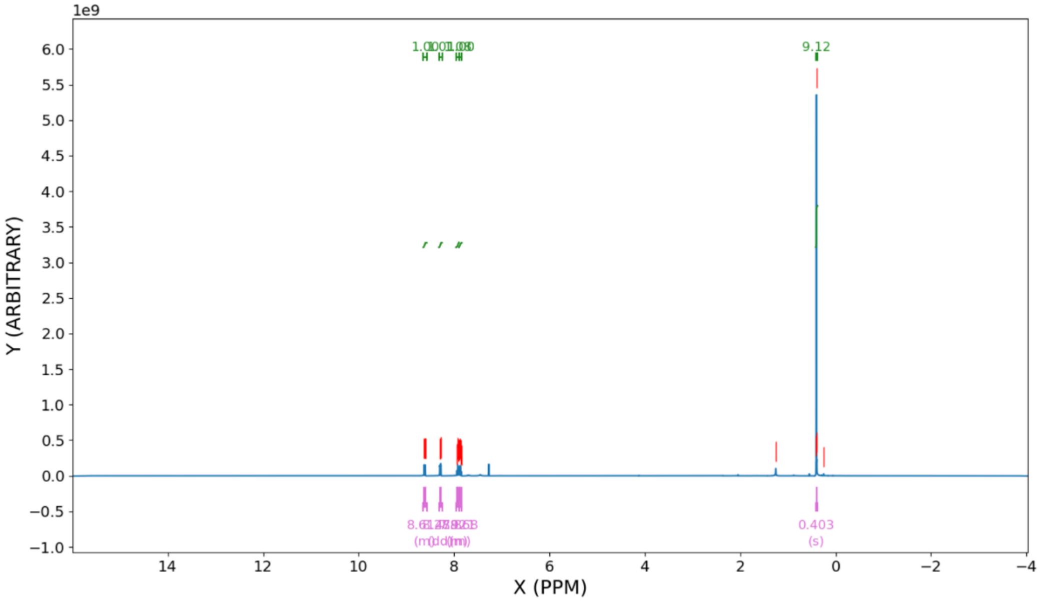

CHMO:0000595 | ^{13}C nuclear magnetic resonance spectroscopy (^{13}C NMR)

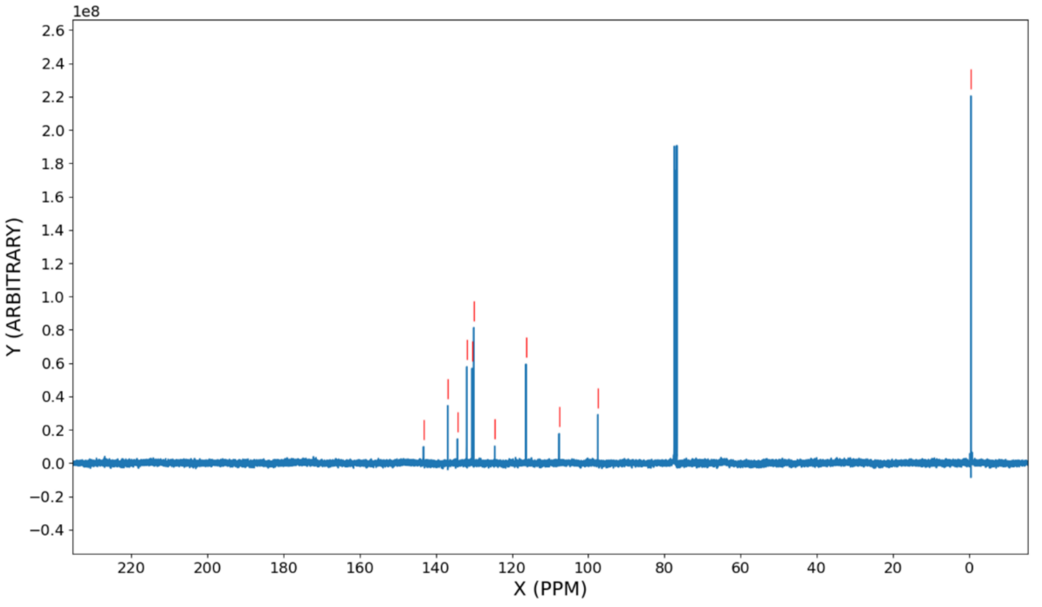

[**12**] (3-Chloroquinoxalin-2-yl)hydrazine

CHMO:0000593 | ^{1}H nuclear magnetic resonance spectroscopy (^{1}H NMR)

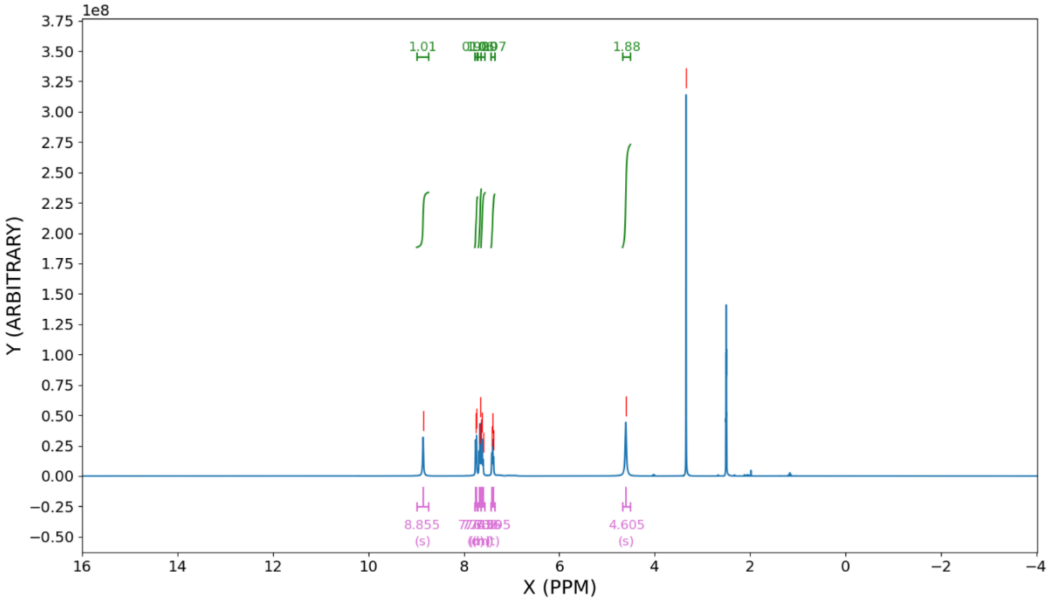

CHMO:0000595 | ^{13}C nuclear magnetic resonance spectroscopy (^{13}C NMR)

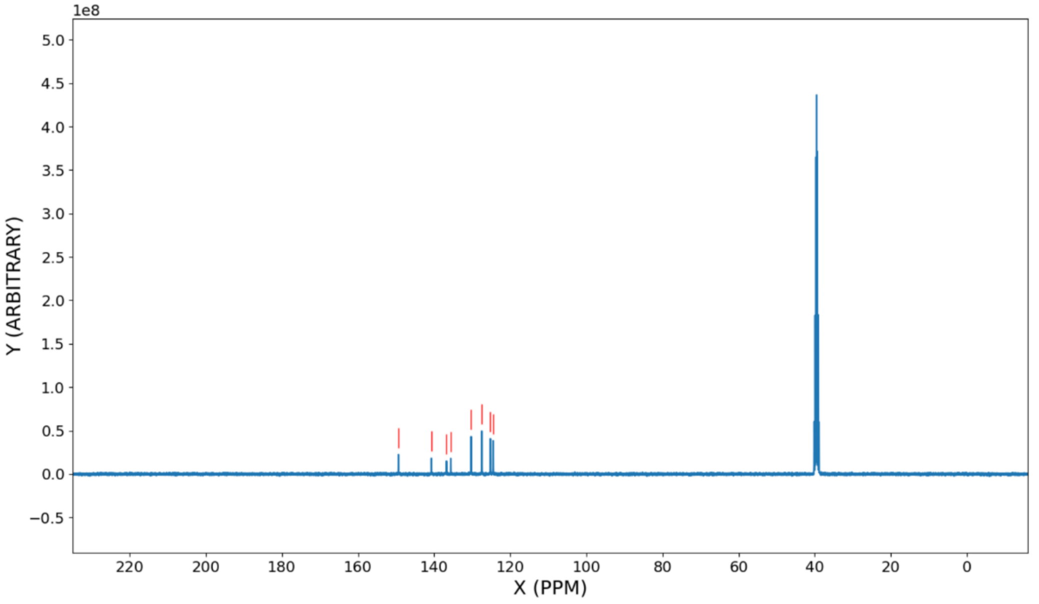

[**S3**] 4,5-Dihydrotetrazolo[1,5-*a*]quinoxaline

CHMO:0000593 | ^{1}H nuclear magnetic resonance spectroscopy (^{1}H NMR)

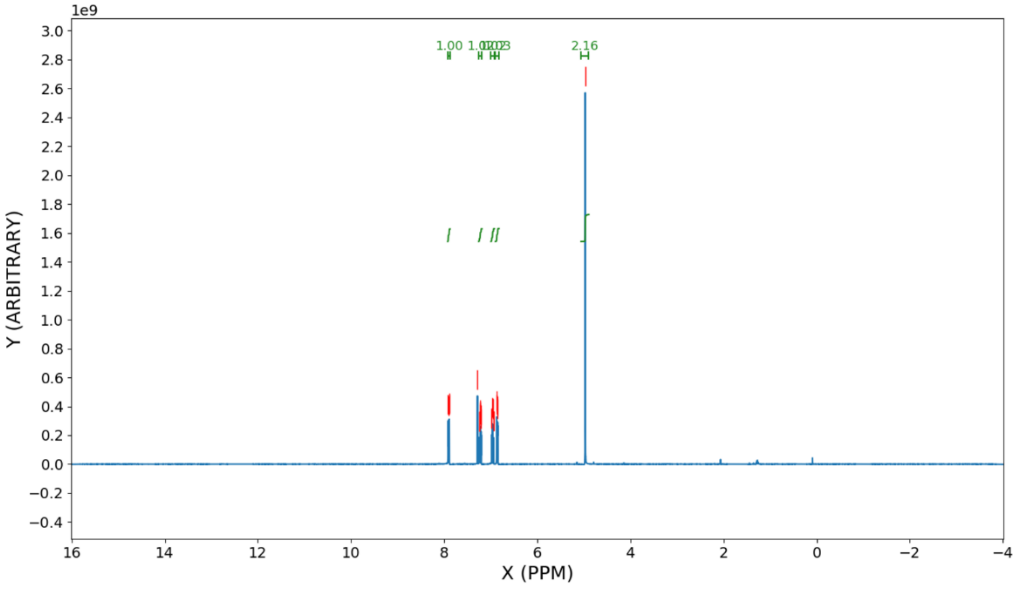

CHMO:0000595 | ^{13}C nuclear magnetic resonance spectroscopy (^{13}C NMR)

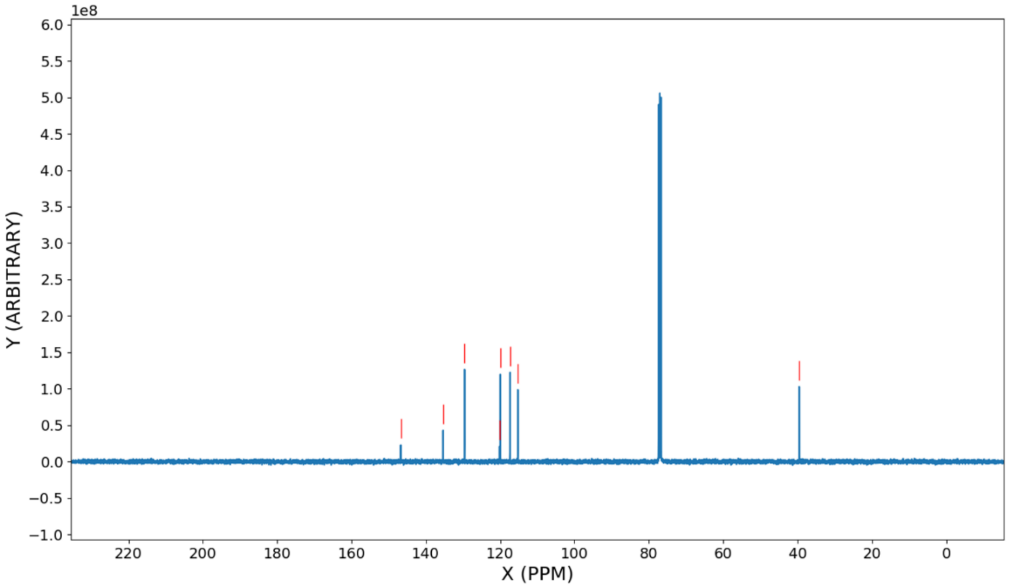

[**S4**] 5-Methyl-4*H*-tetrazolo[1,5-*a*]quinoxaline

CHMO:0000593 | ^{1}H nuclear magnetic resonance spectroscopy (^{1}H NMR)

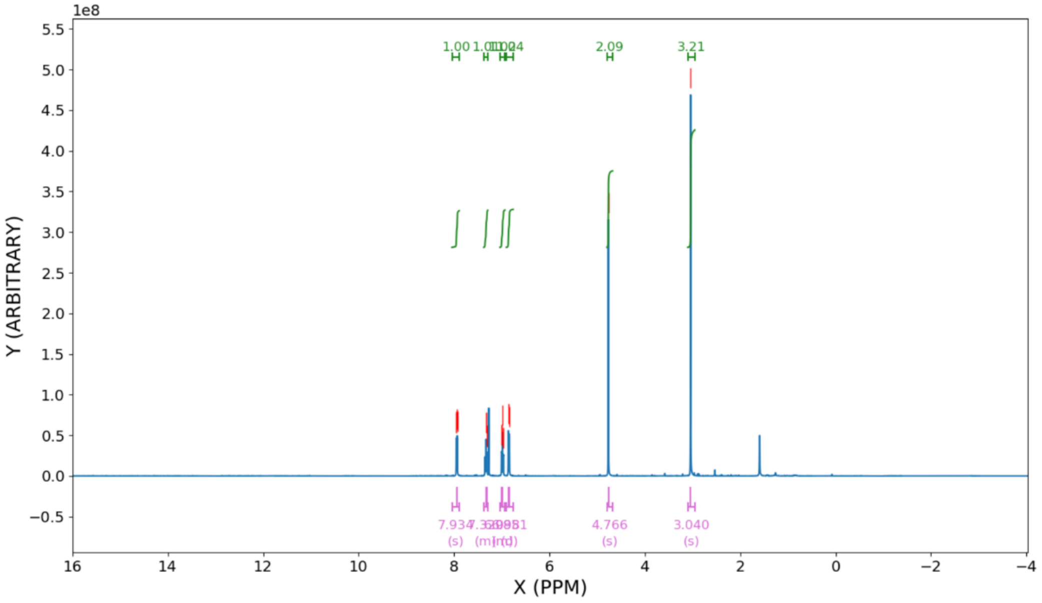

CHMO:0000595 | ^{13}C nuclear magnetic resonance spectroscopy (^{13}C NMR)

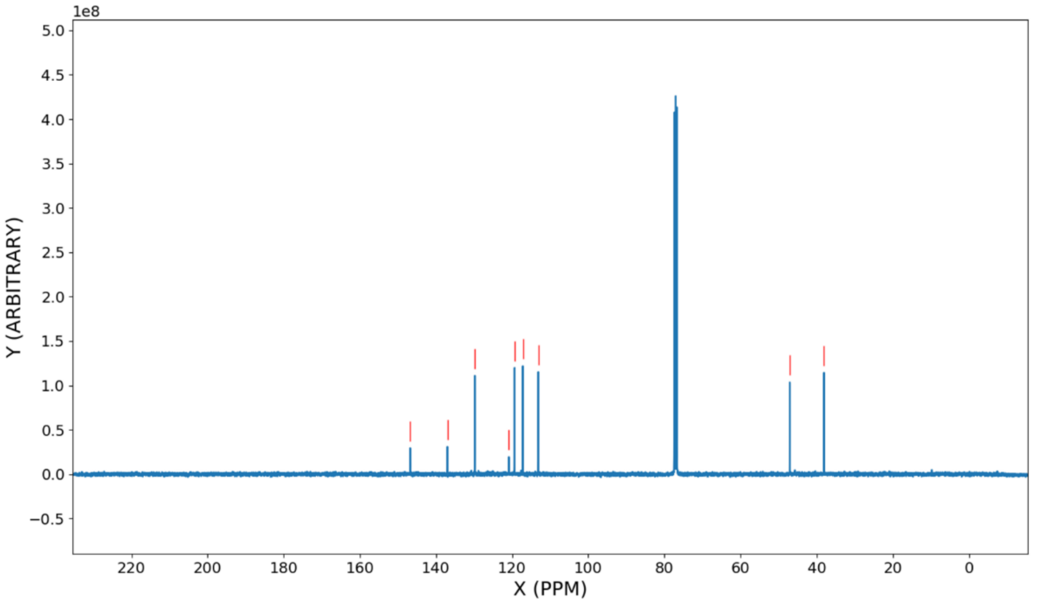

[**14a**] 2-(4-Phenyl-1*H*-1,2,3-triazol-1-yl)quinoxaline

CHMO:0000593 | ^{1}H nuclear magnetic resonance spectroscopy (^{1}H NMR)

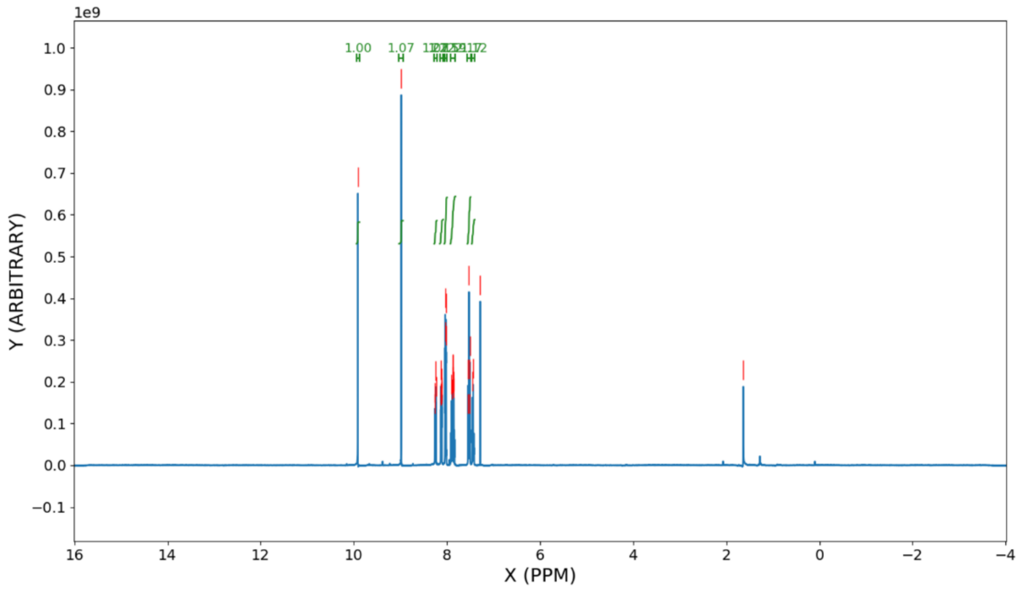

CHMO:0000595 | ^{13}C nuclear magnetic resonance spectroscopy (^{13}C NMR)

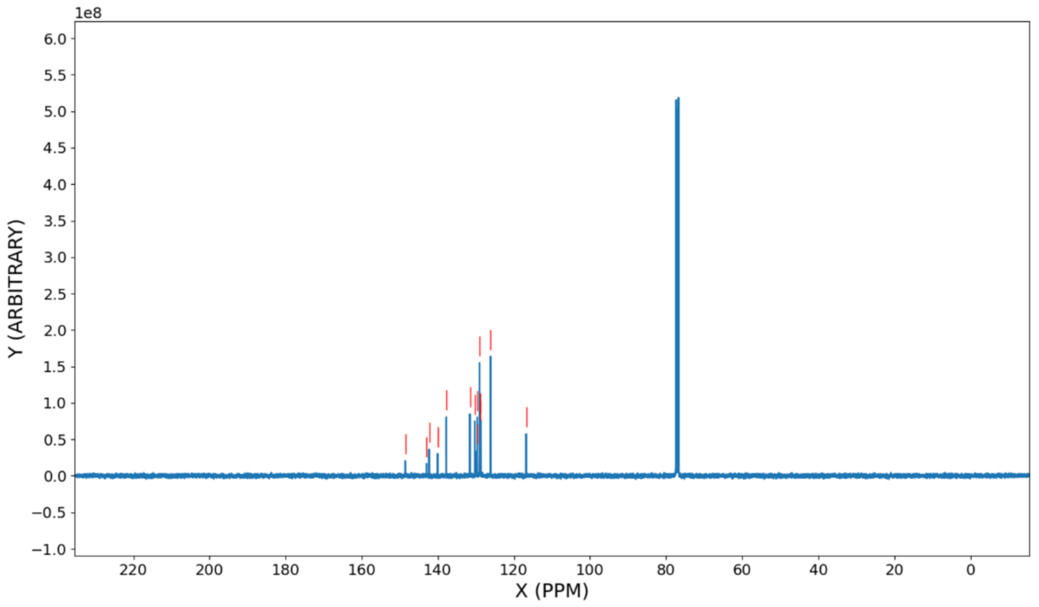

[**14b**] 2-(4-(4-Methoxyphenyl)-1*H*-1,2,3-triazol-1-yl)quinoxaline

CHMO:0000593 | ^{1}H nuclear magnetic resonance spectroscopy (^{1}H NMR)

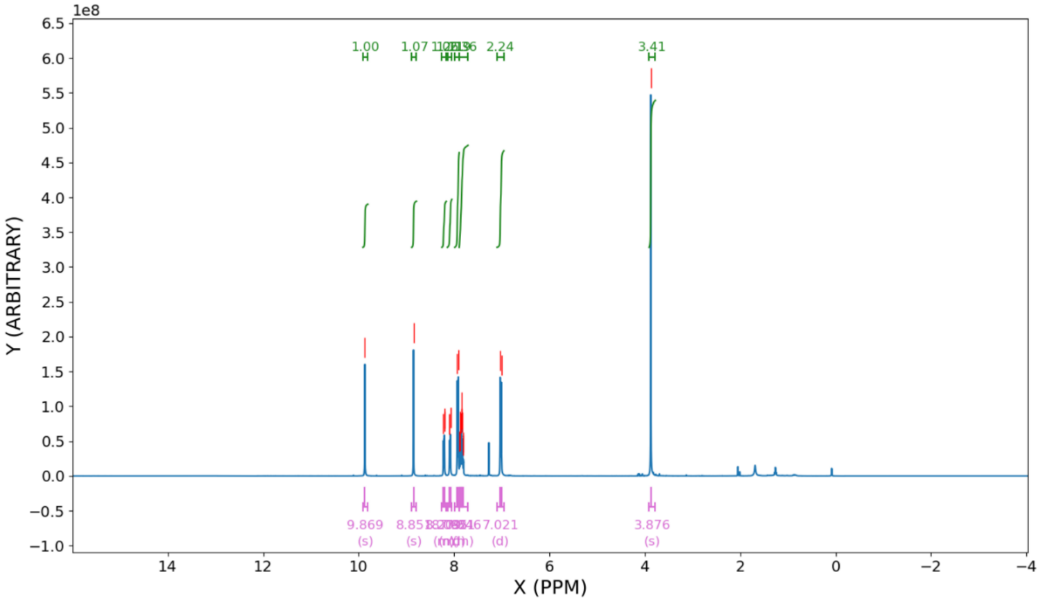

CHMO:0000595 | ^{13}C nuclear magnetic resonance spectroscopy (^{13}C NMR)

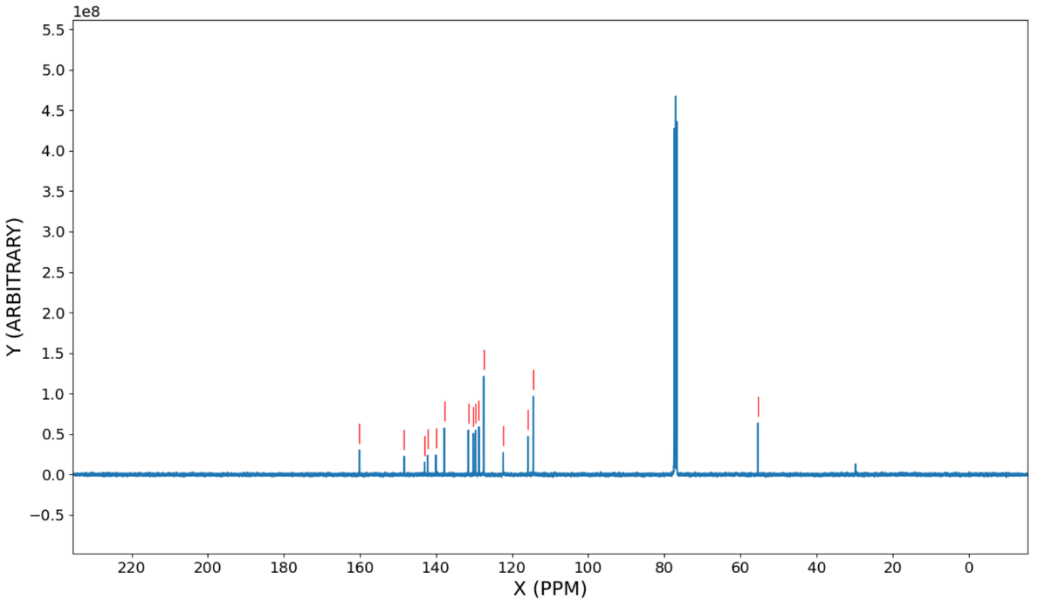

[**14c**] 4-(1-(Quinoxalin-2-yl)-1*H*-1,2,3-triazol-4-yl)benzenaminium chloride

CHMO:0000593 | ^{1}H nuclear magnetic resonance spectroscopy (^{1}H NMR)

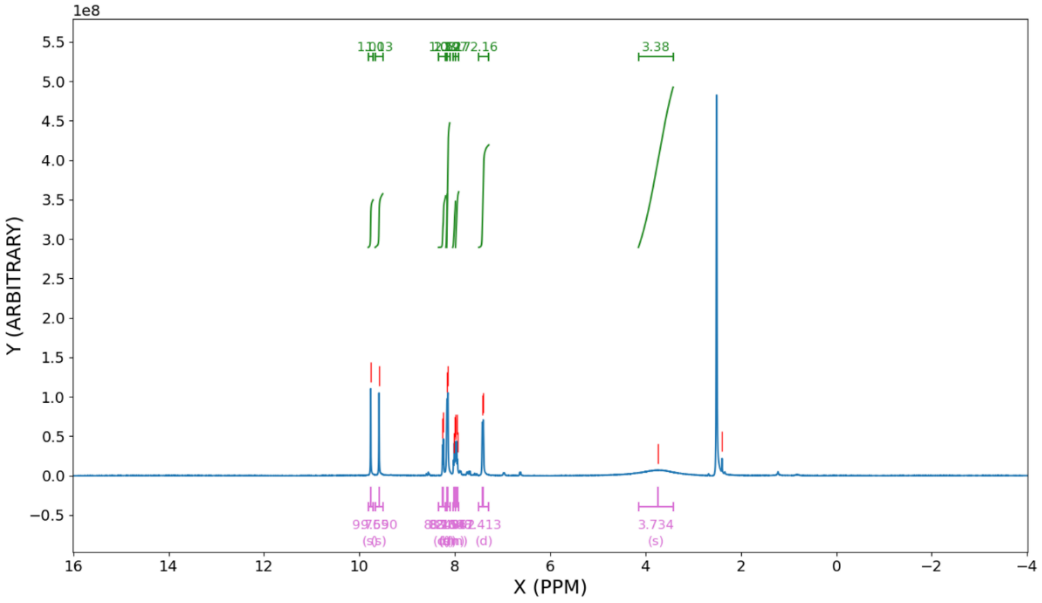

CHMO:0000595 | ^{13}C nuclear magnetic resonance spectroscopy (^{13}C NMR)

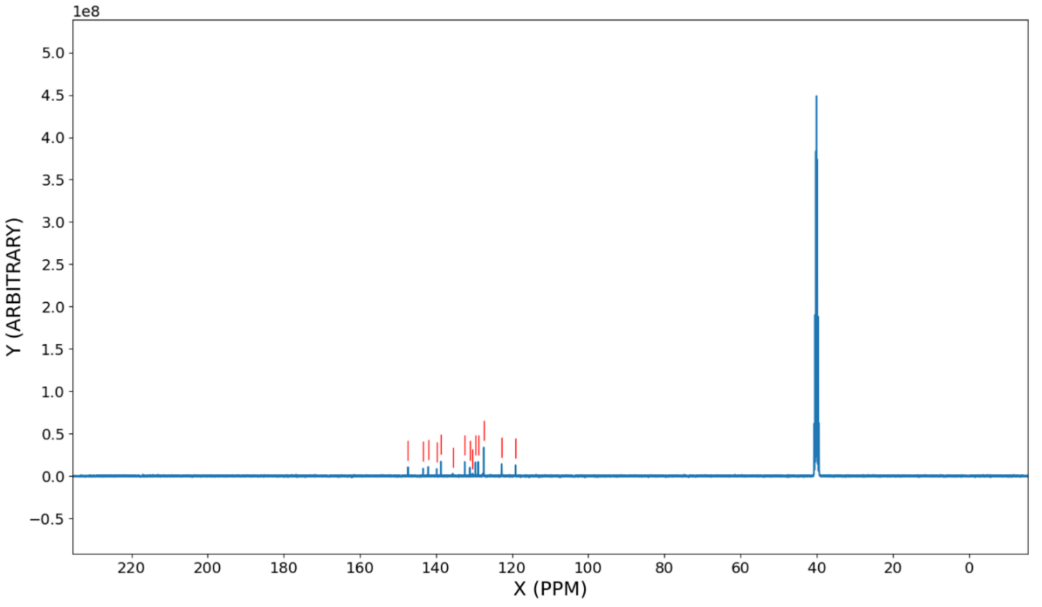

[**14d**] *N*,*N*-Dimethyl-4-(1-(quinoxalin-2-yl)-1*H*-1,2,3-triazol-4-yl)aniline

CHMO:0000593 | ^{1}H nuclear magnetic resonance spectroscopy (^{1}H NMR)

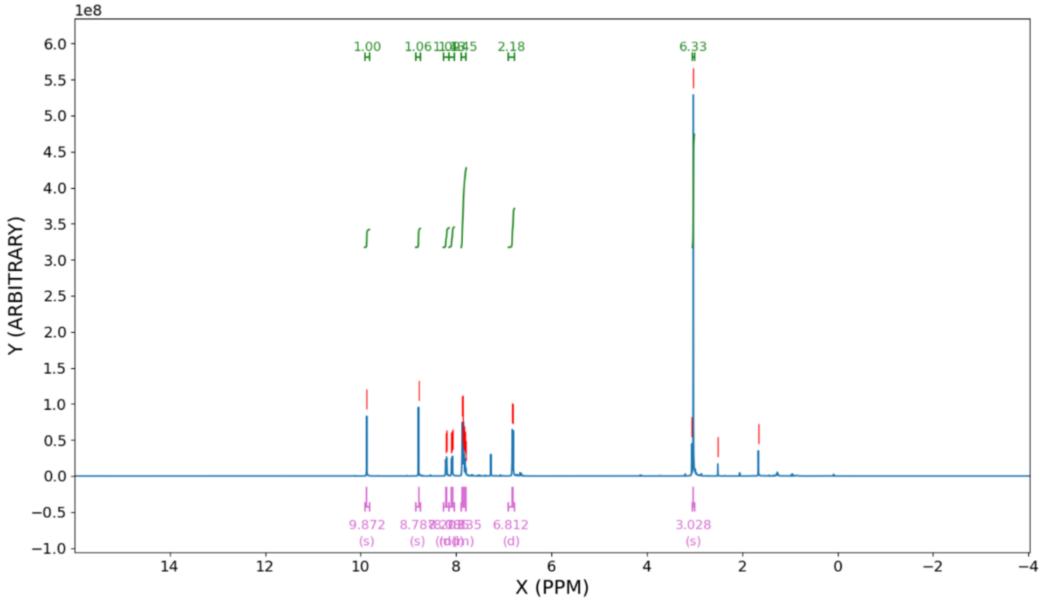

CHMO:0000595 | ^{13}C nuclear magnetic resonance spectroscopy (^{13}C NMR)

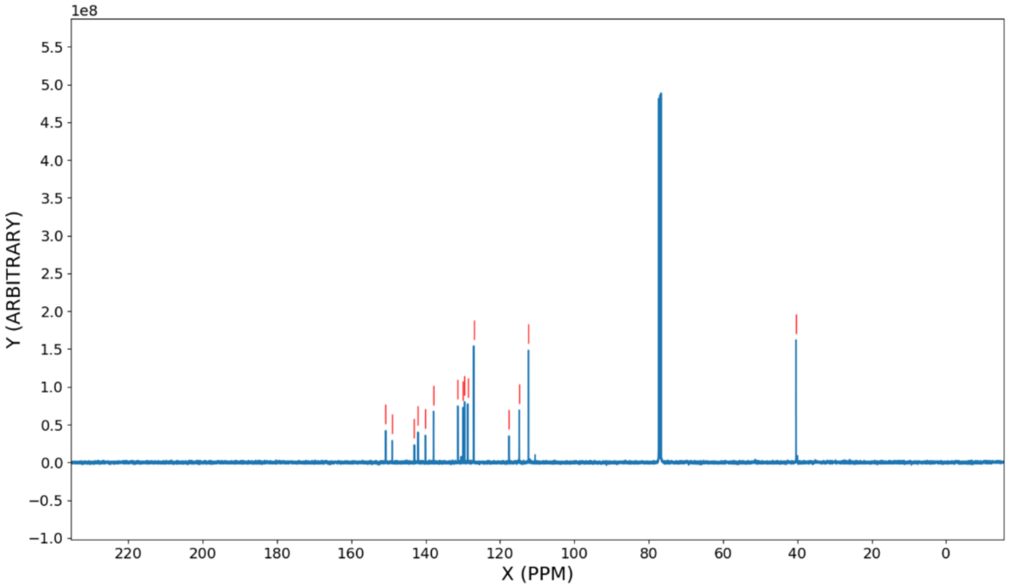

[**14e**] Methyl 4-(1-(quinoxalin-2-yl)-1*H*-1,2,3-triazol-4-yl)benzoate

CHMO:0000593 | ^{1}H nuclear magnetic resonance spectroscopy (^{1}H NMR)

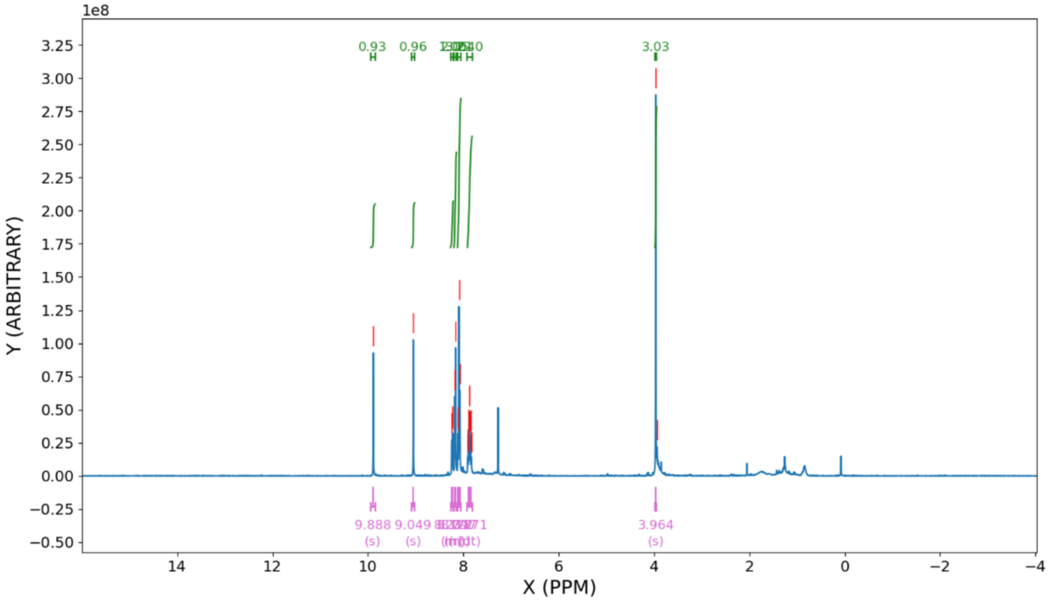

CHMO:0000595 | ^{13}C nuclear magnetic resonance spectroscopy (^{13}C NMR)

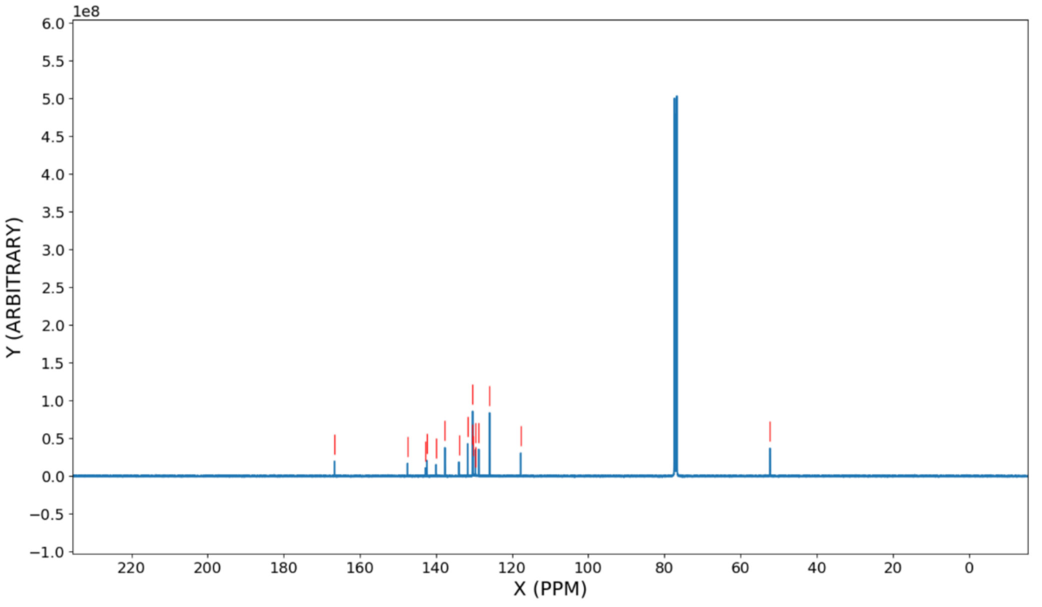

[**S10**] Quinoxalin-2-ylamine

CHMO:0000593 | ^{1}H nuclear magnetic resonance spectroscopy (^{1}H NMR)

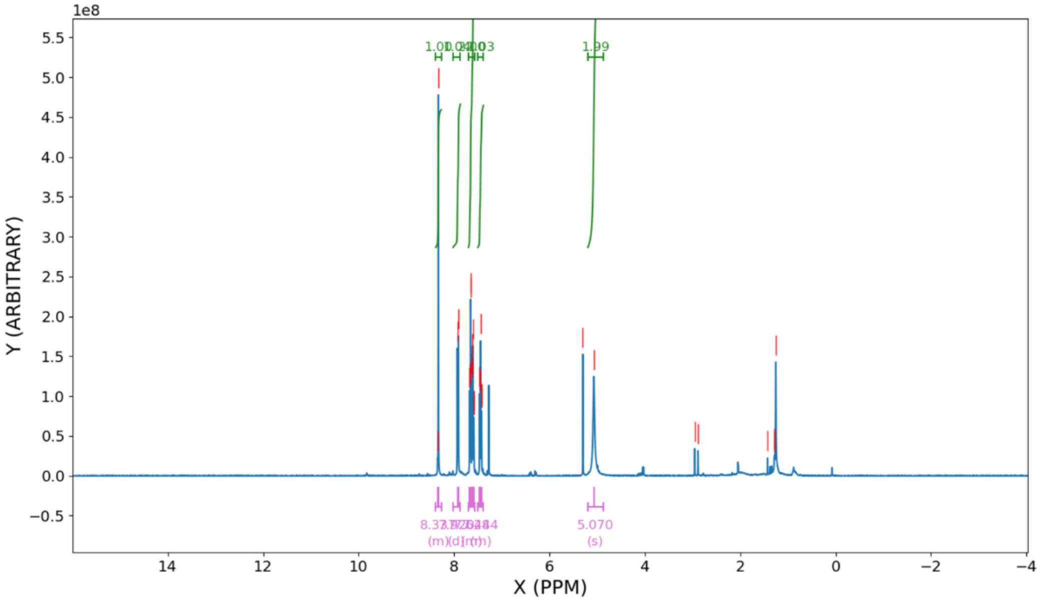

[**14f**] 2-(4-(4-Ethynylphenyl)-1*H*-1,2,3-triazol-1-yl)quinoxaline

CHMO:0000593 | ^{1}H nuclear magnetic resonance spectroscopy (^{1}H NMR)

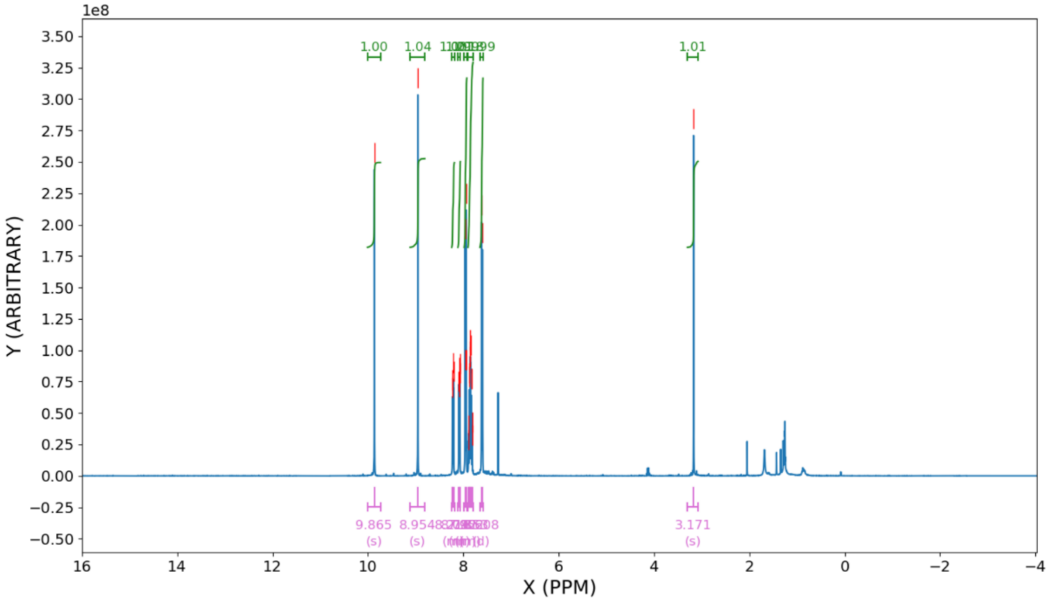

CHMO:0000595 | ^{13}C nuclear magnetic resonance spectroscopy (^{13}C NMR)

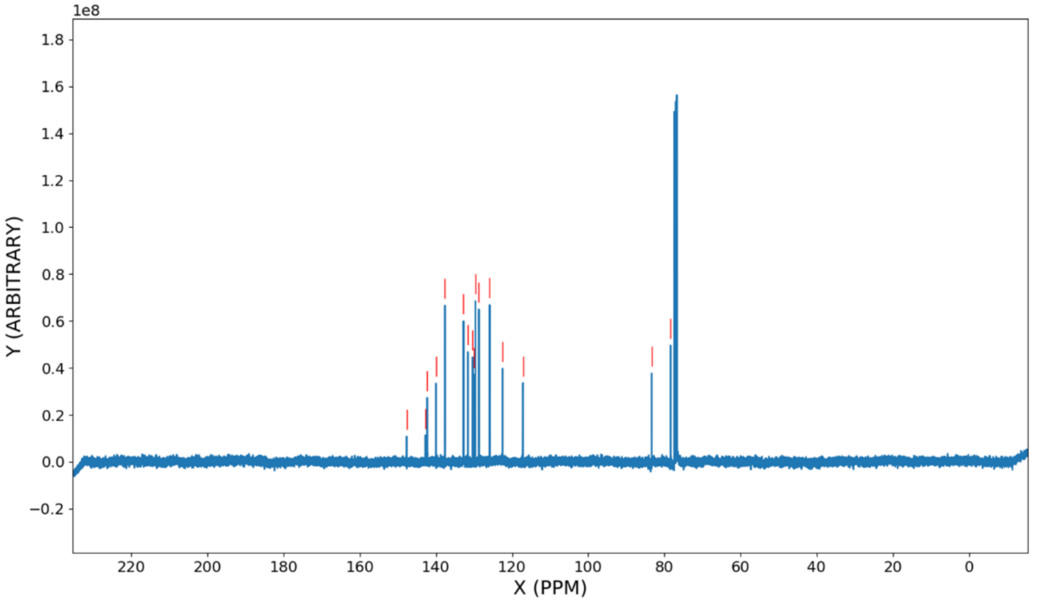

[**14g**] 2-(4-(3-Methoxyphenyl)-1*H*-1,2,3-triazol-1-yl)quinoxaline

CHMO:0000593 | ^{1}H nuclear magnetic resonance spectroscopy (^{1}H NMR)

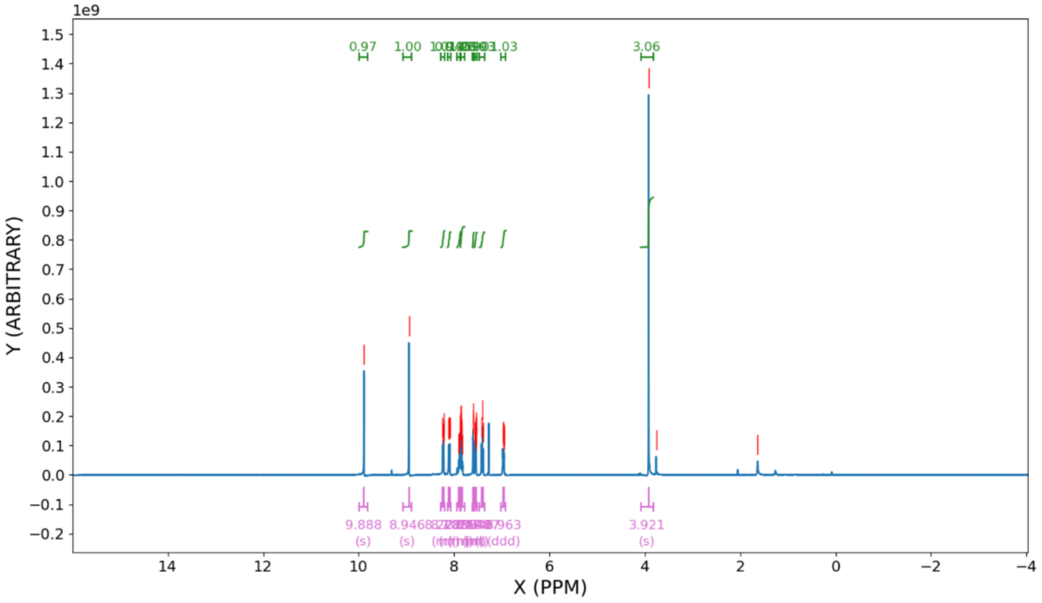

CHMO:0000595 | ^{13}C nuclear magnetic resonance spectroscopy (^{13}C NMR)

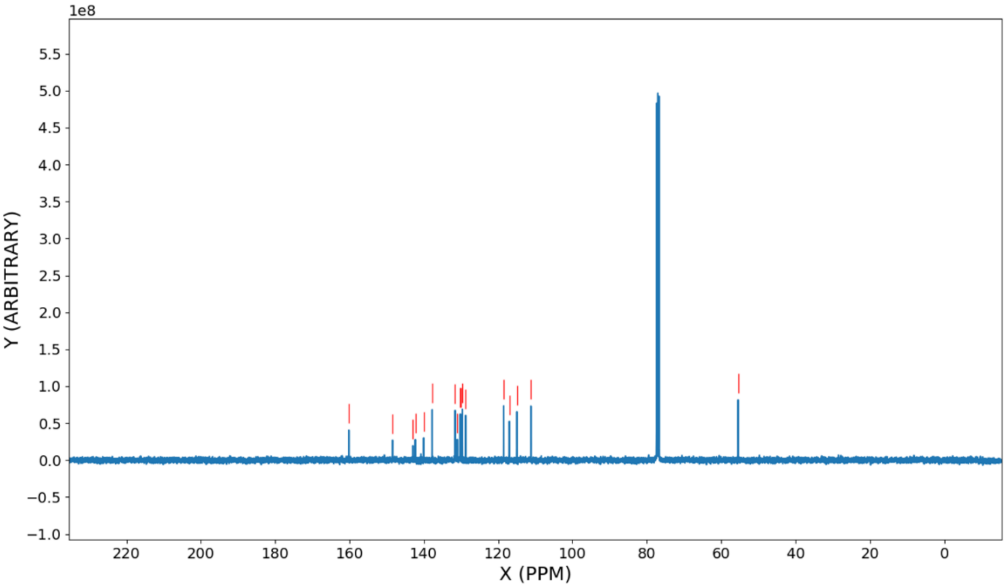

[**14h**] 3-(1-(Quinoxalin-2-yl)-1*H*-1,2,3-triazol-4-yl)benzenaminium

CHMO:0000593 | ^{1}H nuclear magnetic resonance spectroscopy (^{1}H NMR)

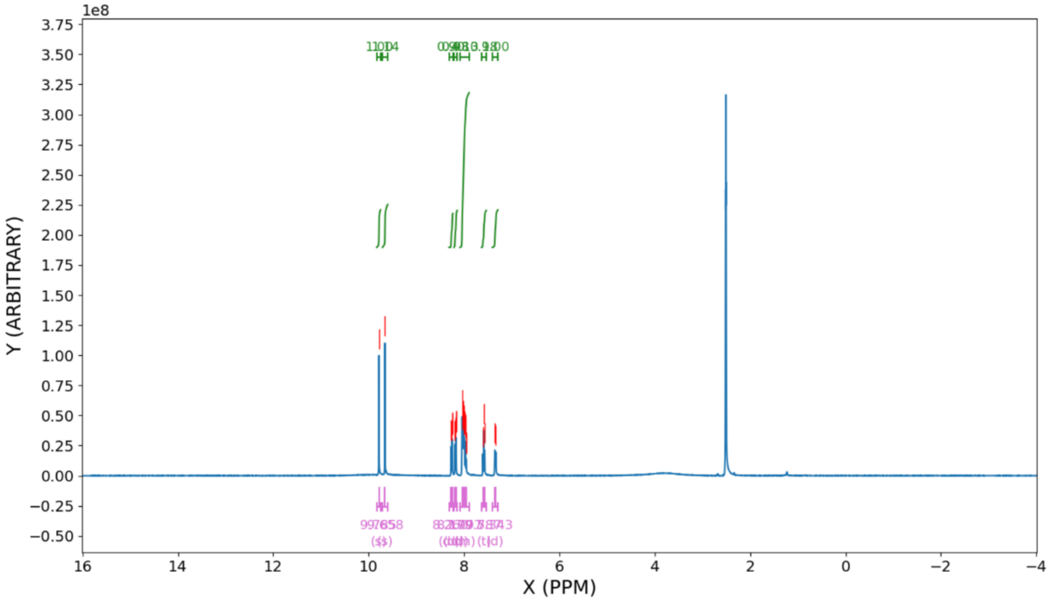

CHMO:0000595 | ^{13}C nuclear magnetic resonance spectroscopy (^{13}C NMR)

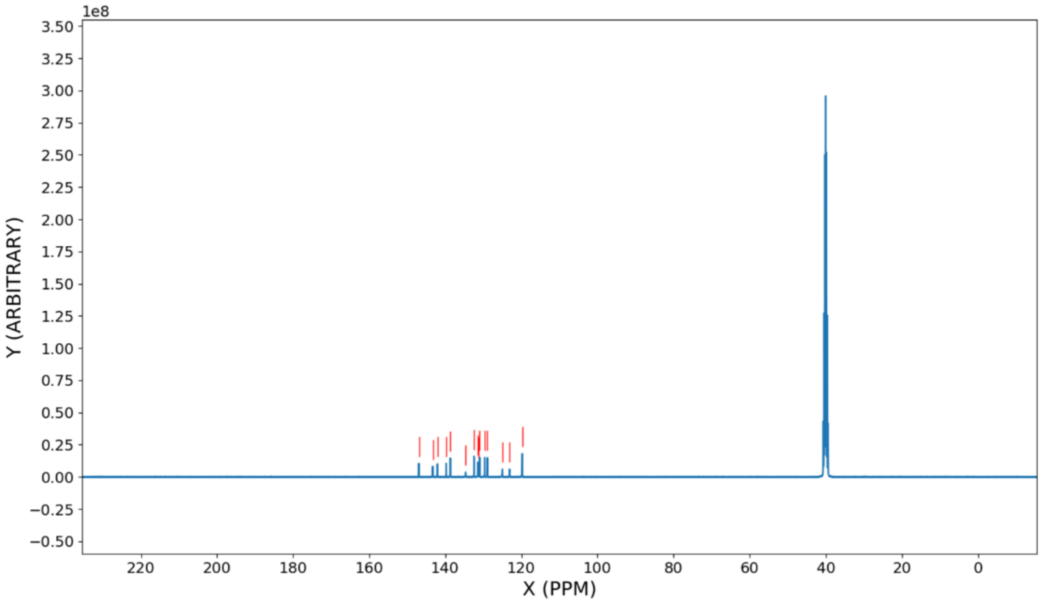

[**14i**] 2-((1-(Quinoxalin-2-yl)-1*H*-1,2,3-triazol-4-yl)methyl)isoindoline-1,3-dione

CHMO:0000593 | ^{1}H nuclear magnetic resonance spectroscopy (^{1}H NMR)

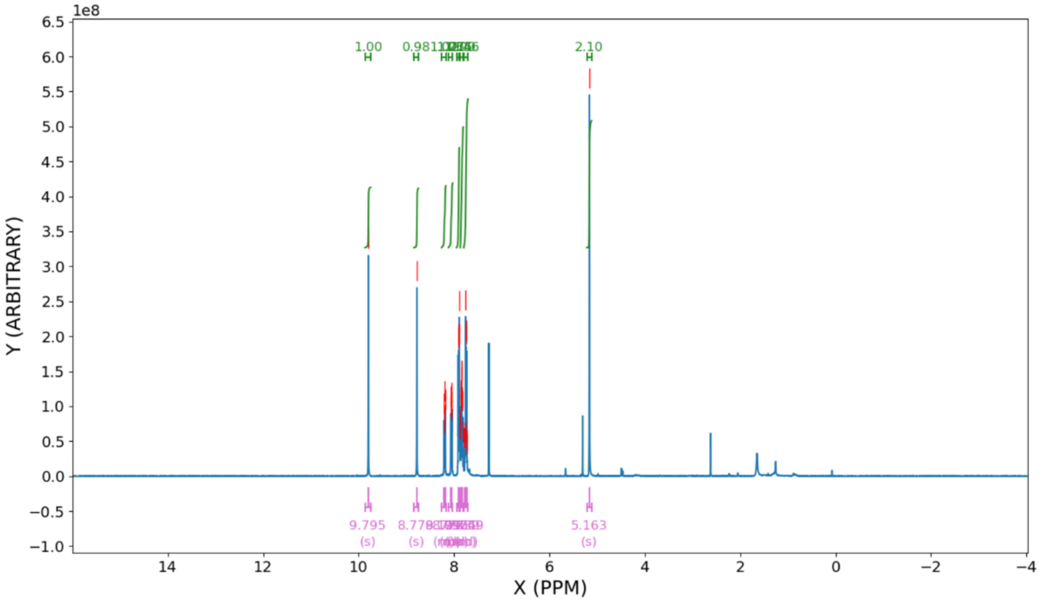

CHMO:0000595 | ^{13}C nuclear magnetic resonance spectroscopy (^{13}C NMR)

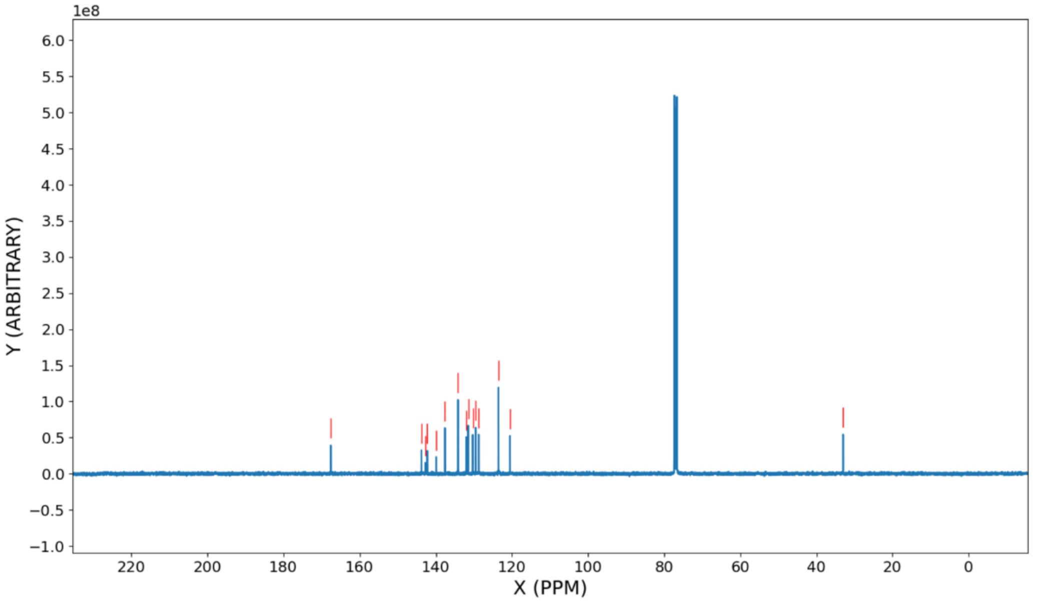

[**4j**] But-3-ynyl 4-methylbenzenesulfonate

CHMO:0000593 | ^{1}H nuclear magnetic resonance spectroscopy (^{1}H NMR)

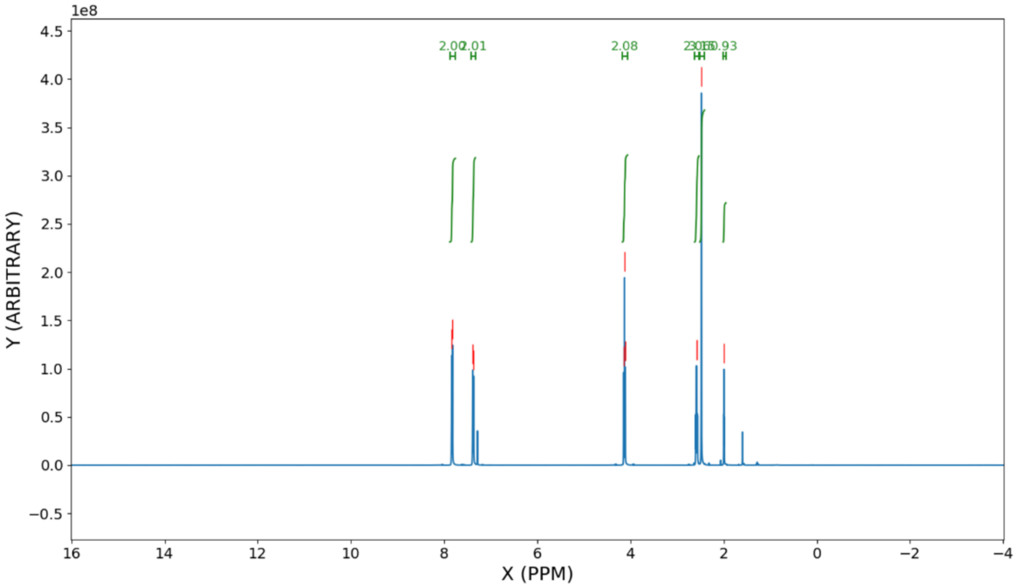

CHMO:0000595 | ^{13}C nuclear magnetic resonance spectroscopy (^{13}C NMR)

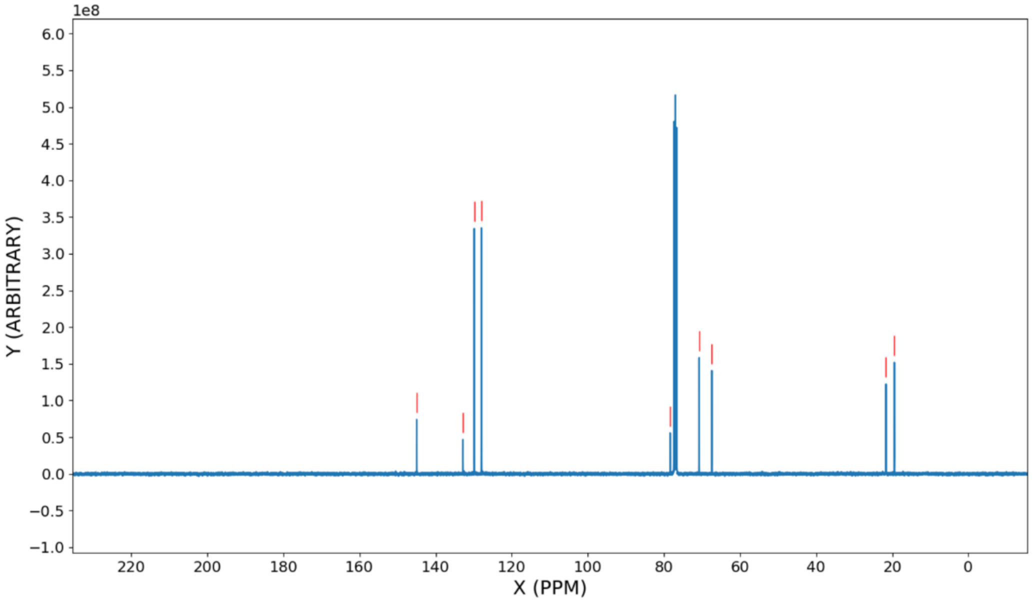

[**14j**] 2-(1-(Quinoxalin-2-yl)-1*H*-1,2,3-triazol-4-yl)ethyl 4-methylbenzenesulfonate

CHMO:0000593 | ^{1}H nuclear magnetic resonance spectroscopy (^{1}H NMR)

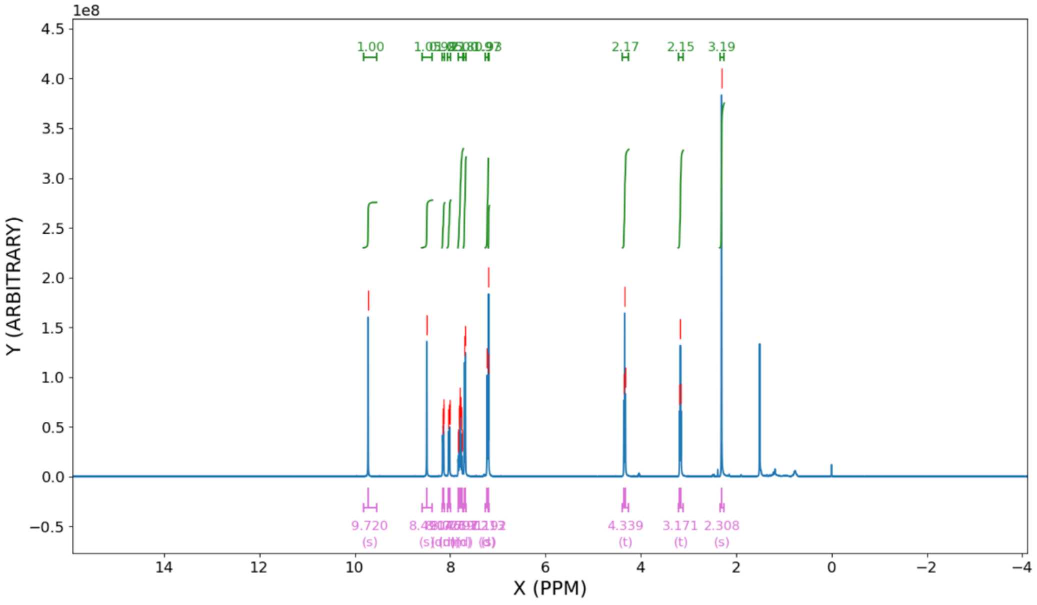

CHMO:0000595 | ^{13}C nuclear magnetic resonance spectroscopy (^{13}C NMR)

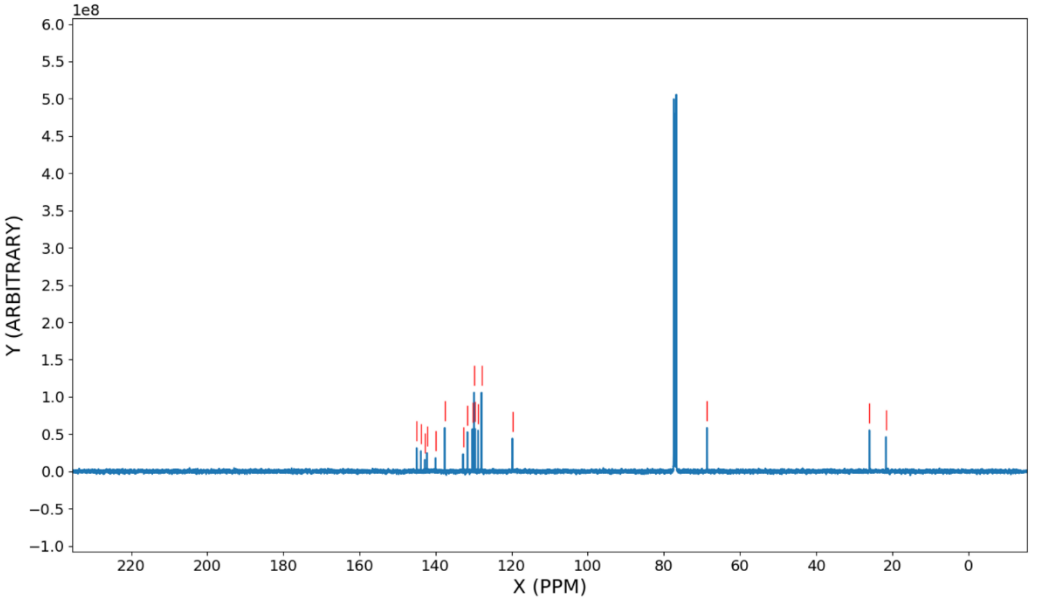

[**14j***] *N*,*N*-Diethyl-2-(1-(quinoxalin-2-yl)-1*H*-1,2,3-triazol-4-yl)ethan-1-amine

CHMO:0000593 | ^{1}H nuclear magnetic resonance spectroscopy (^{1}H NMR)

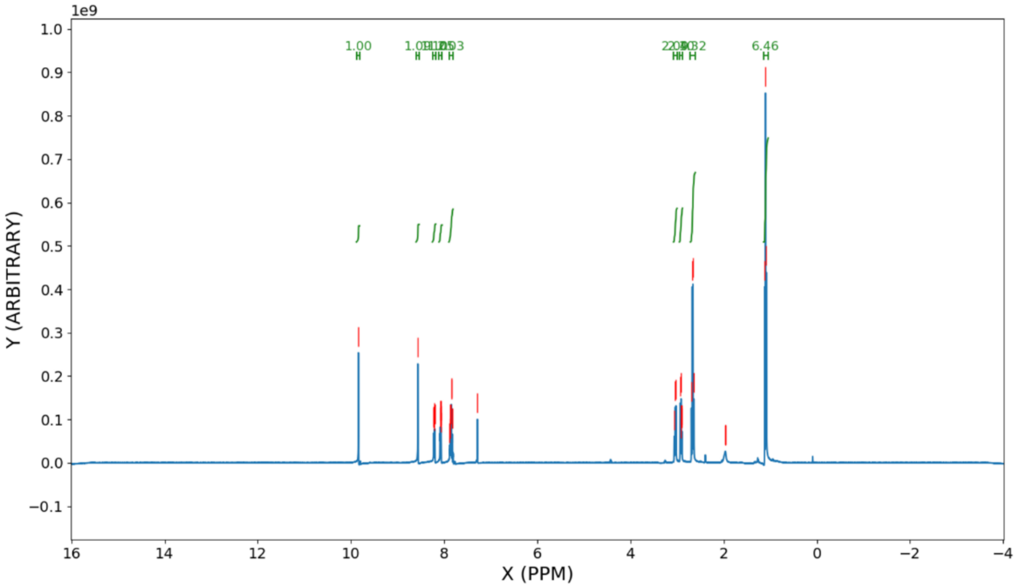

CHMO:0000595 | ^{13}C nuclear magnetic resonance spectroscopy (^{13}C NMR)

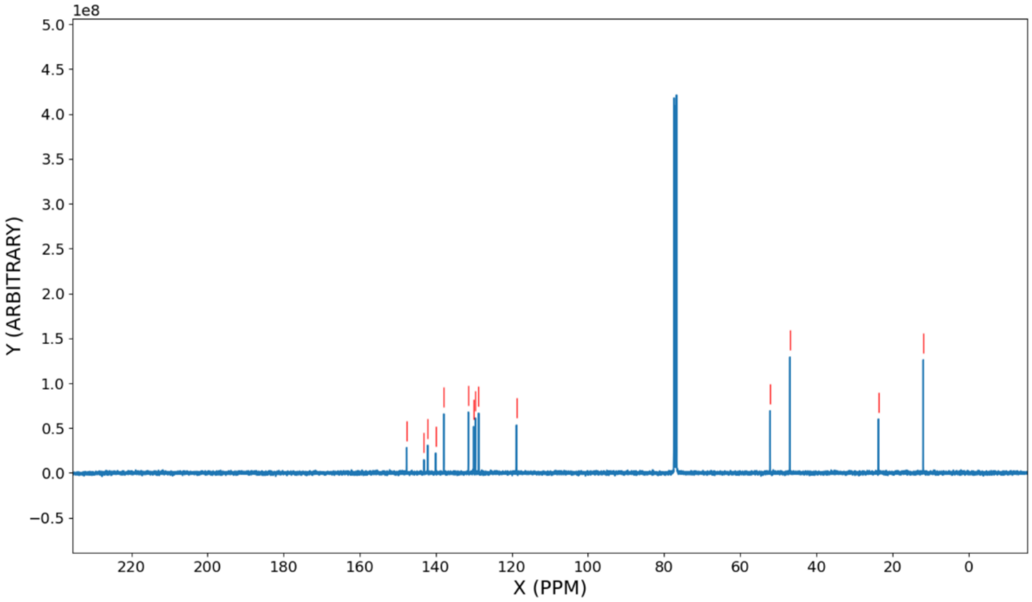

[**14k**] 2-(4-Butyl-1*H*-1,2,3-triazol-1-yl)quinoxaline

CHMO:0000593 | ^{1}H nuclear magnetic resonance spectroscopy (^{1}H NMR)

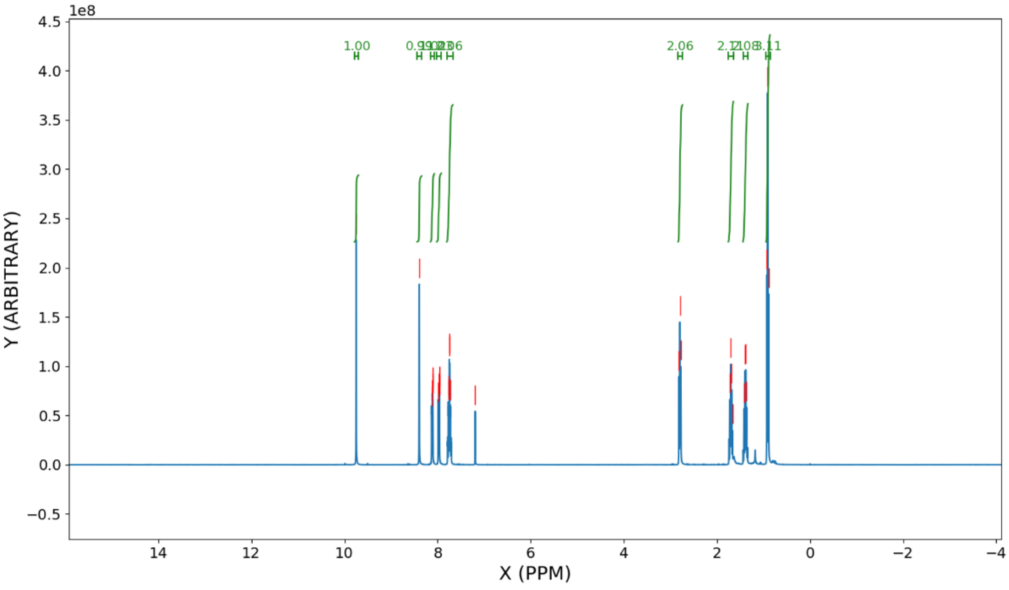

CHMO:0000595 | ^{13}C nuclear magnetic resonance spectroscopy (^{13}C NMR)

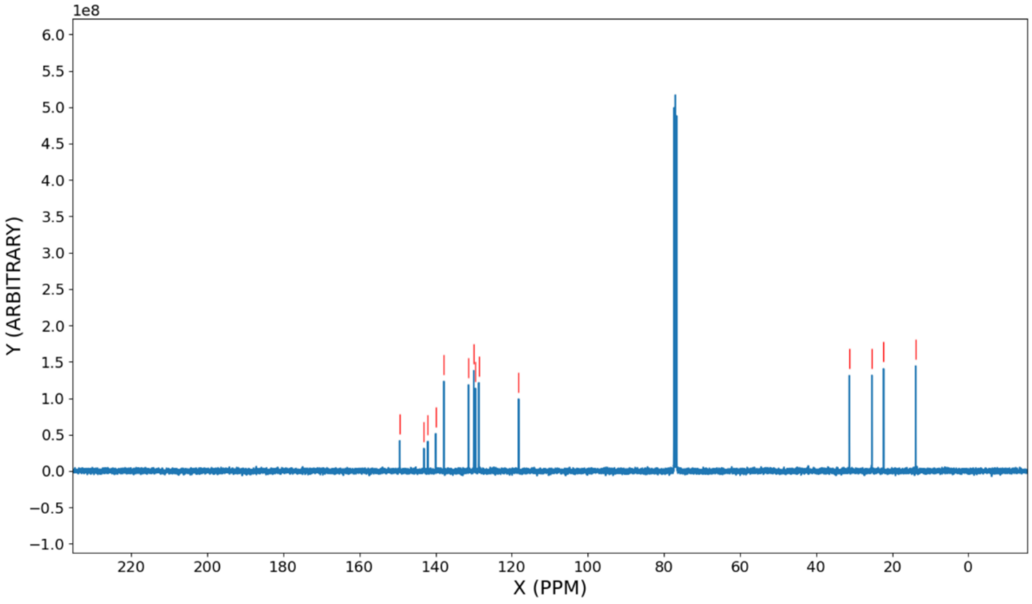

[**14l**] (1-(Quinoxalin-2-yl)-1*H*-1,2,3-triazol-4-yl)methyl acetate

CHMO:0000593 | ^{1}H nuclear magnetic resonance spectroscopy (^{1}H NMR)

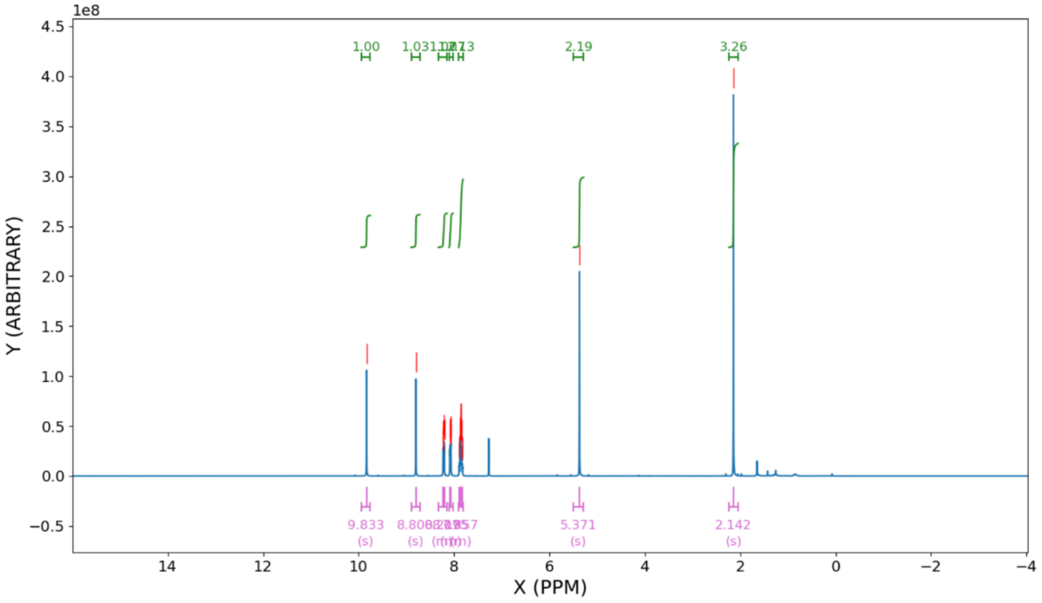

CHMO:0000595 | ^{13}C nuclear magnetic resonance spectroscopy (^{13}C NMR)

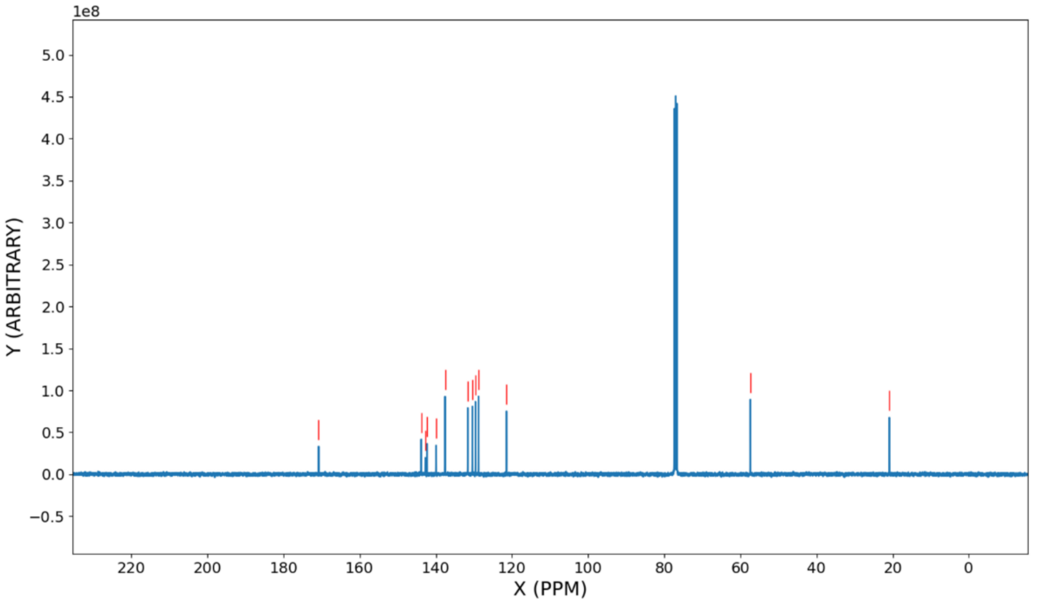

[**14m**] (1-(Quinoxalin-2-yl)-1*H*-1,2,3-triazol-4-yl)methyl acrylate

CHMO:0000593 | ^{1}H nuclear magnetic resonance spectroscopy (^{1}H NMR)

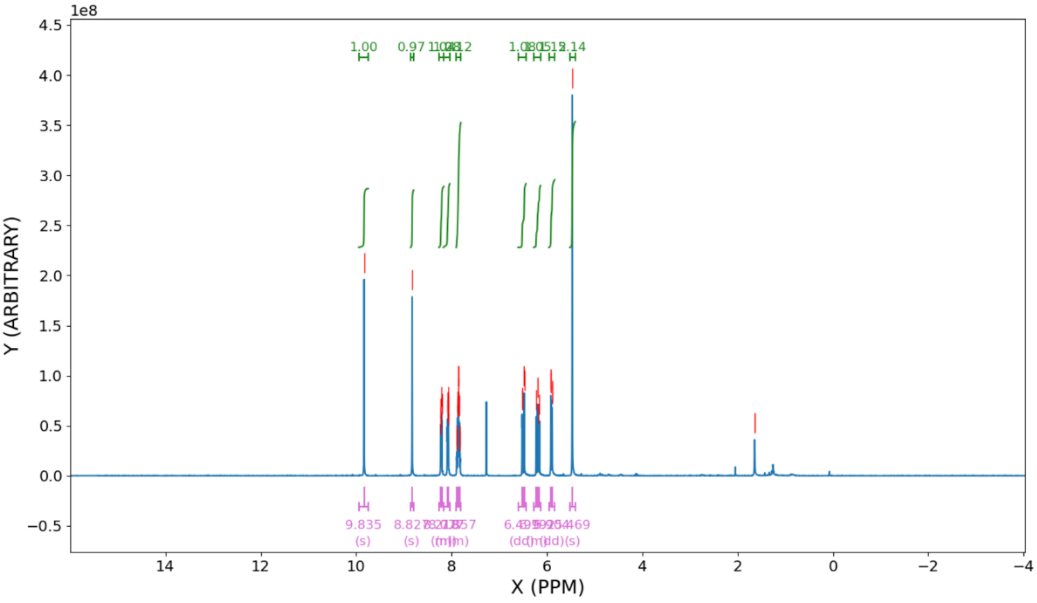

CHMO:0000595 | ^{13}C nuclear magnetic resonance spectroscopy (^{13}C NMR)

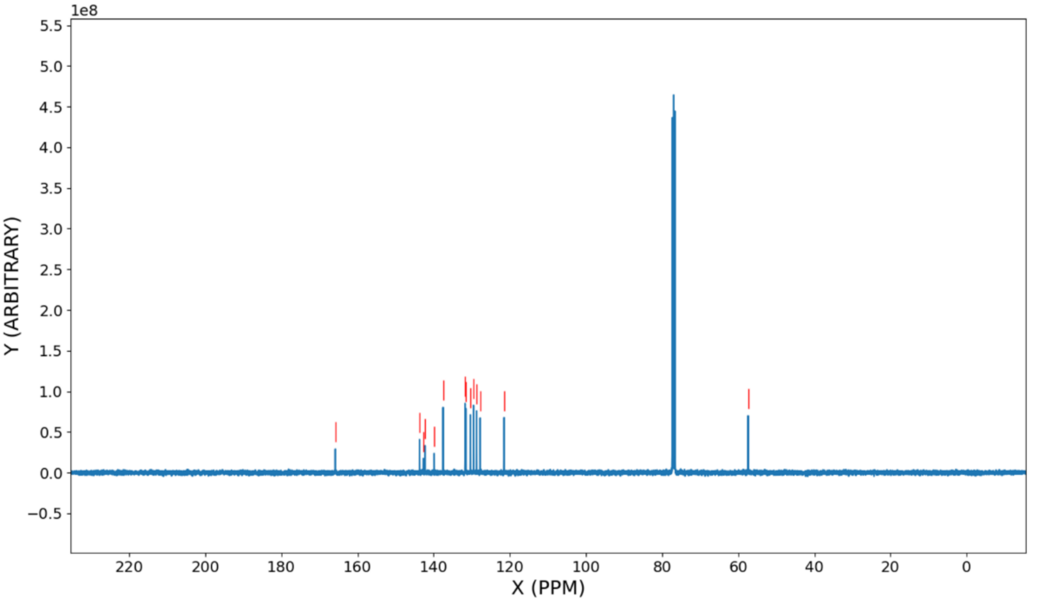

[**14n**] *N*-(Prop-2-yn-1-yl)-*N*-((1-(quinoxalin-2-yl)-1*H*-1,2,3-triazol-4-yl)methyl)prop-2-yn-1-amine

CHMO:0000593 | ^{1}H nuclear magnetic resonance spectroscopy (^{1}H NMR)

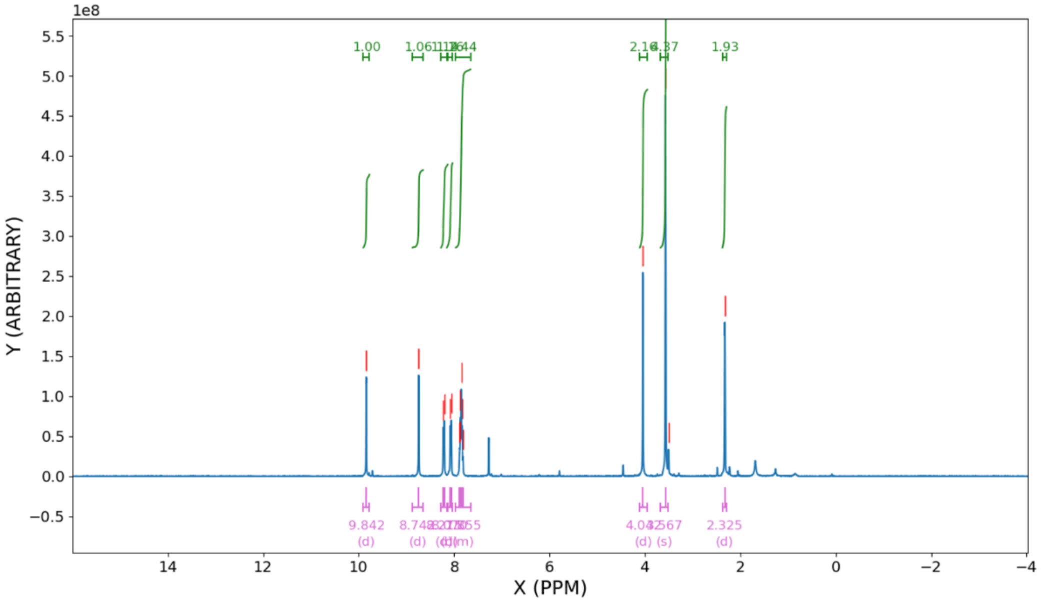

CHMO:0000595 | ^{13}C nuclear magnetic resonance spectroscopy (^{13}C NMR)

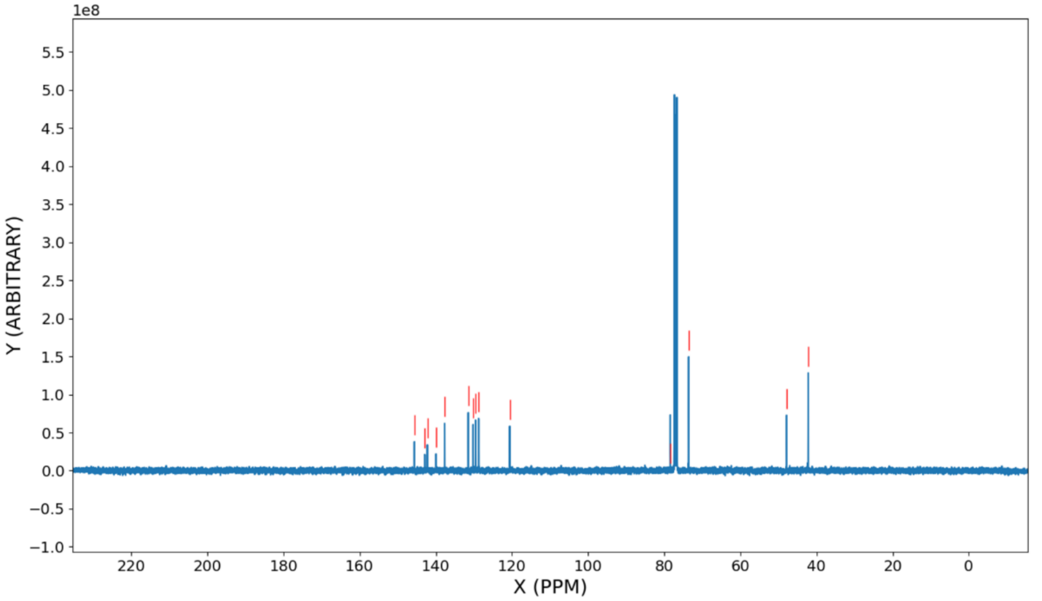

[**14o**] (1-(Quinoxalin-2-yl)-1*H*-1,2,3-triazol-4-yl)methanol

CHMO:0000593 | ^{1}H nuclear magnetic resonance spectroscopy (^{1}H NMR)

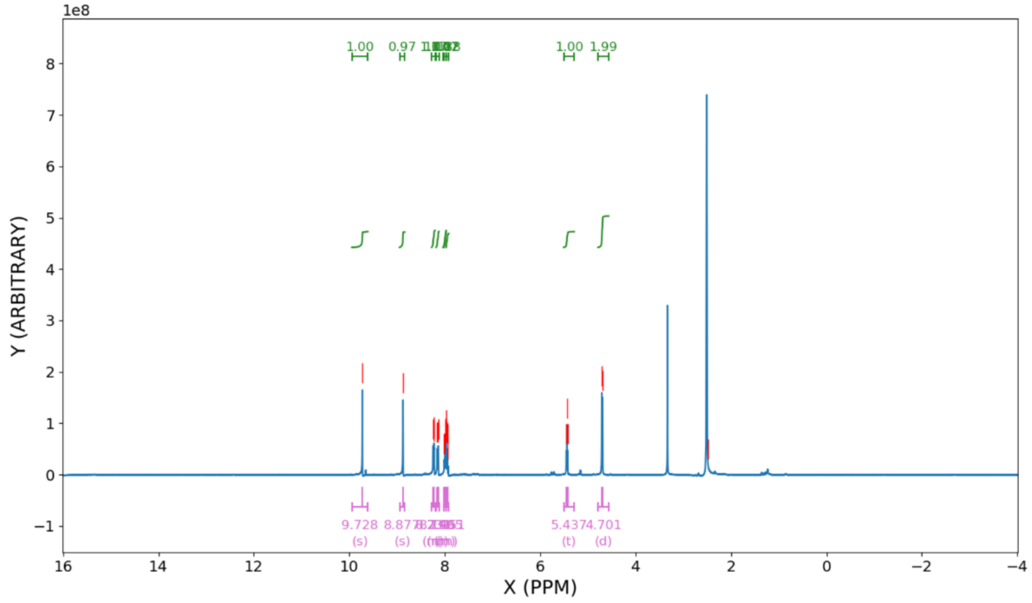

CHMO:0000595 | ^{13}C nuclear magnetic resonance spectroscopy (^{13}C NMR)

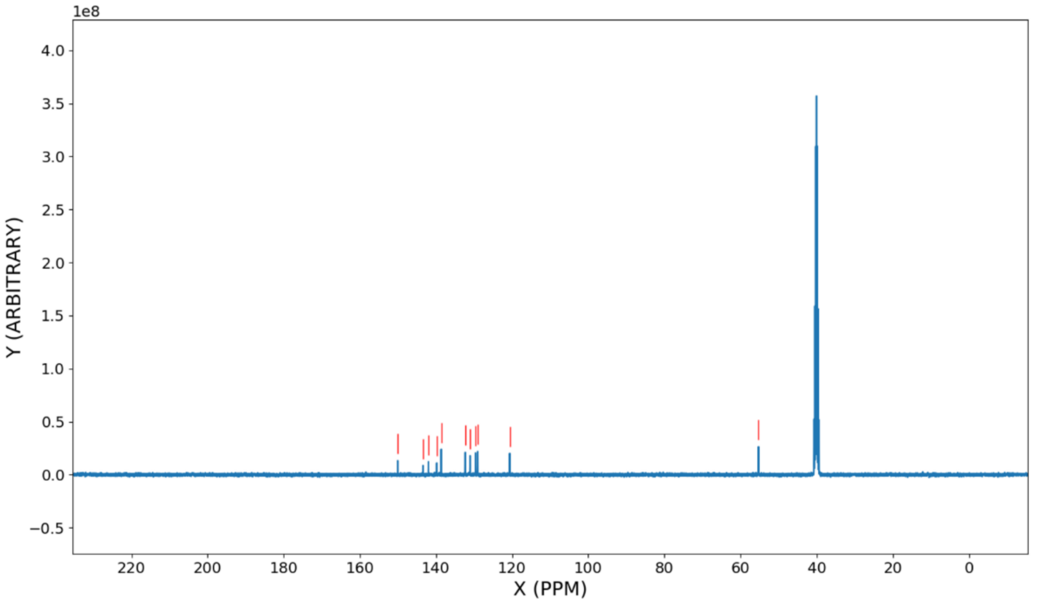

[**4p**] 4-Ethynylbenzoic acid

CHMO:0000593 | ^{1}H nuclear magnetic resonance spectroscopy (^{1}H NMR)

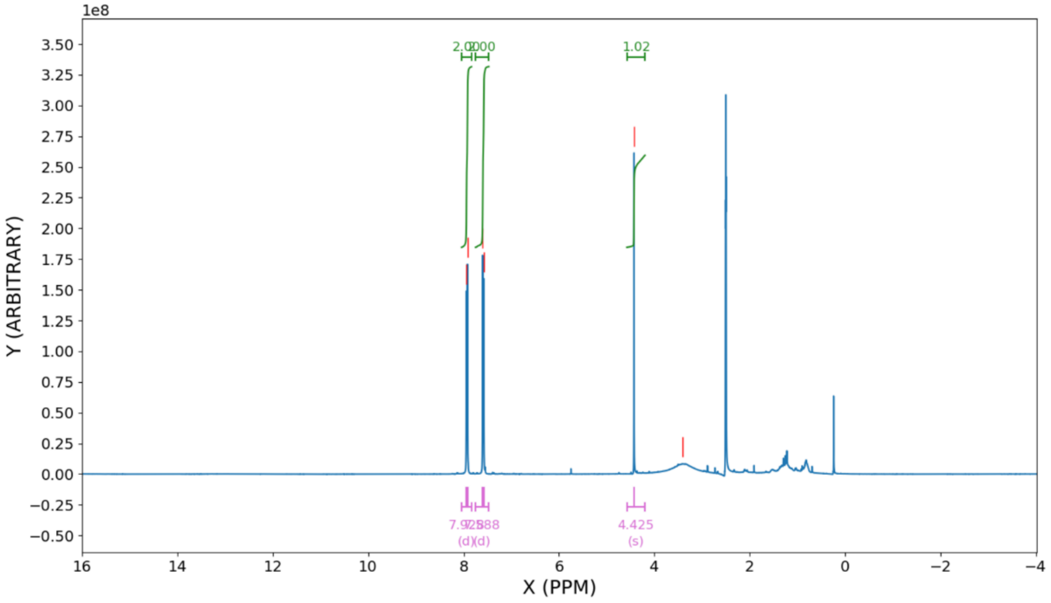

CHMO:0000595 | ^{13}C nuclear magnetic resonance spectroscopy (^{13}C NMR)

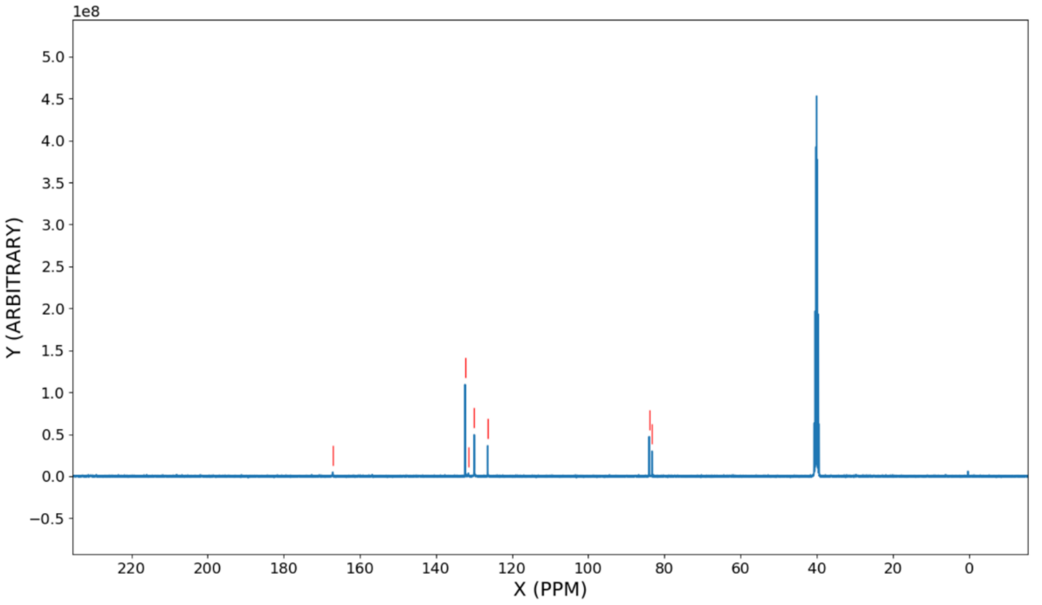

[**15a**] 2-(4-Butyl-1*H*-1,2,3-triazol-1-yl)-3-methylquinoxaline

CHMO:0000593 | ^{1}H nuclear magnetic resonance spectroscopy (^{1}H NMR)

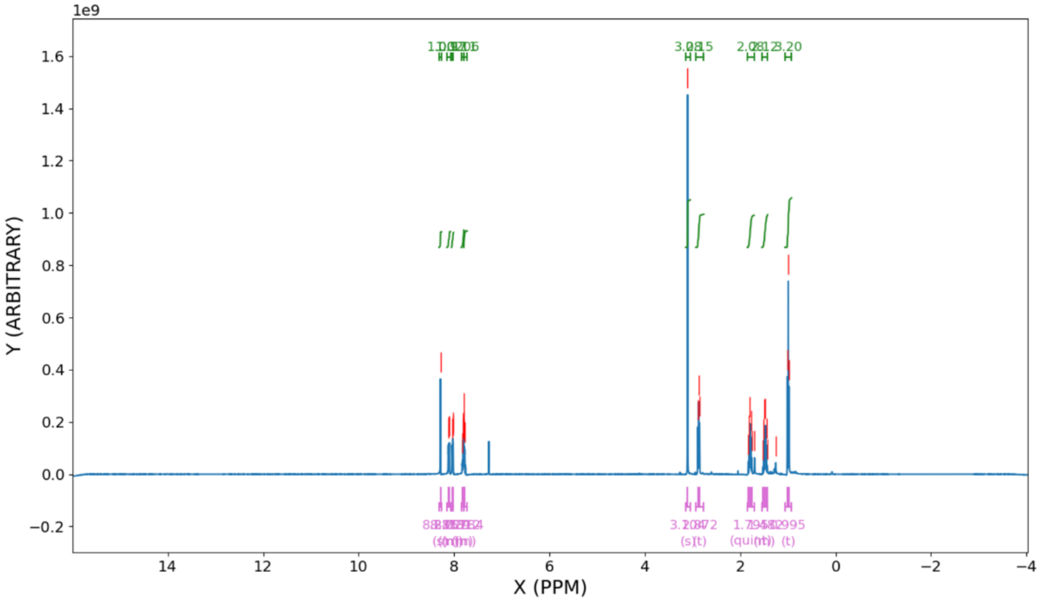

CHMO:0000595 | ^{13}C nuclear magnetic resonance spectroscopy (^{13}C NMR)

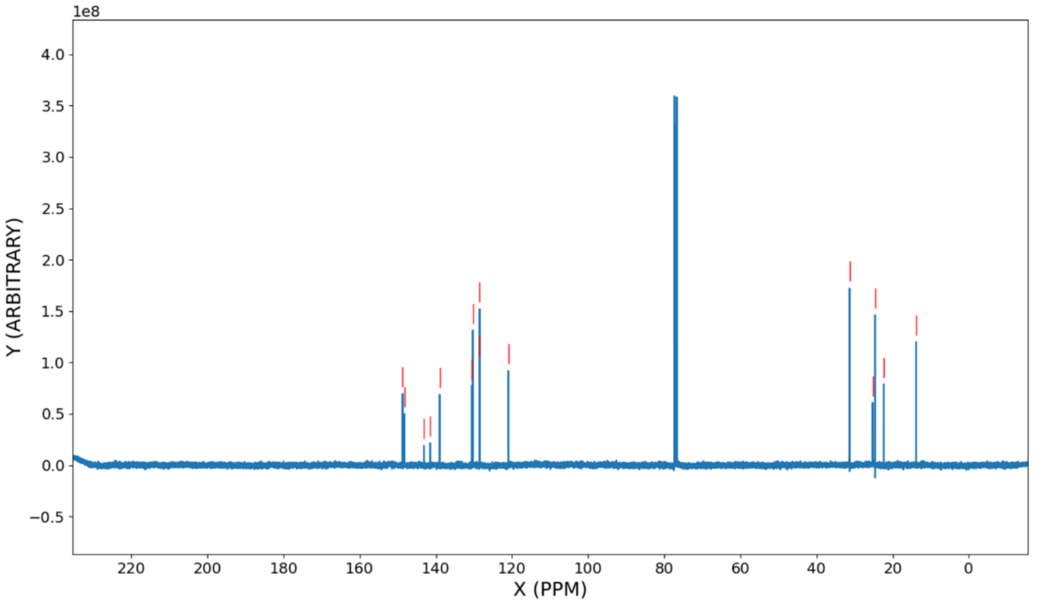

[**17a**] 3-Methylquinoxalin-2-amine

CHMO:0000593 | ^{1}H nuclear magnetic resonance spectroscopy (^{1}H NMR)

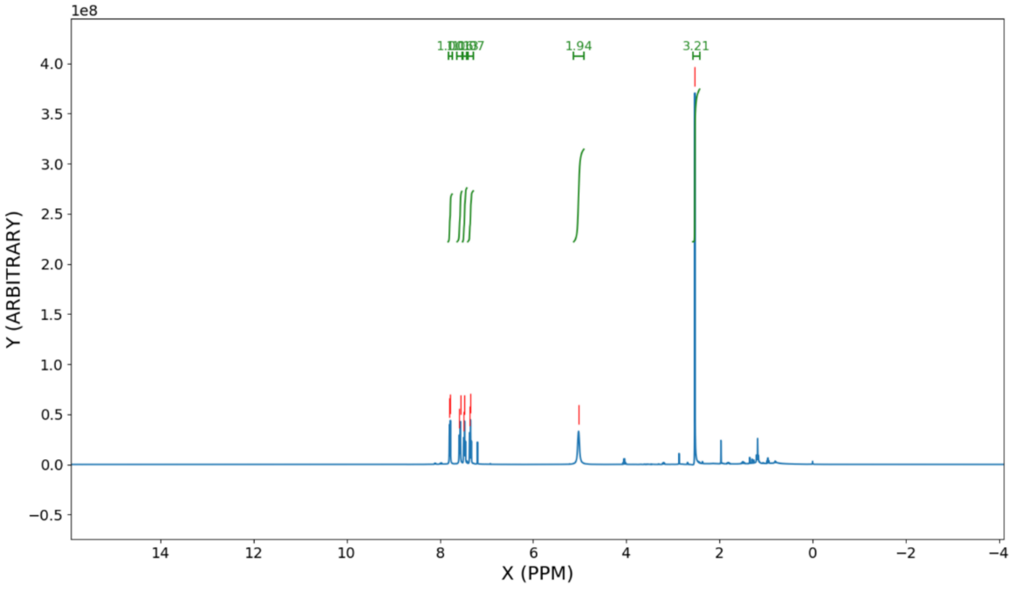

CHMO:0000595 | ^{13}C nuclear magnetic resonance spectroscopy (^{13}C NMR)

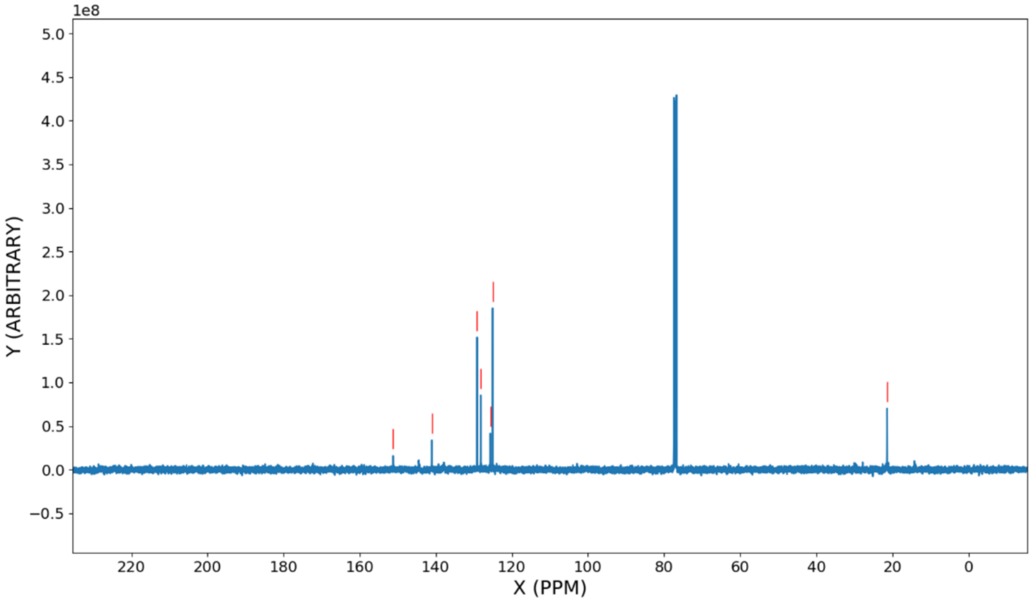

[**15b**] 2-(4-Butyl-1*H*-1,2,3-triazol-1-yl)-3-isopropylquinoxaline

CHMO:0000593 | ^{1}H nuclear magnetic resonance spectroscopy (^{1}H NMR)

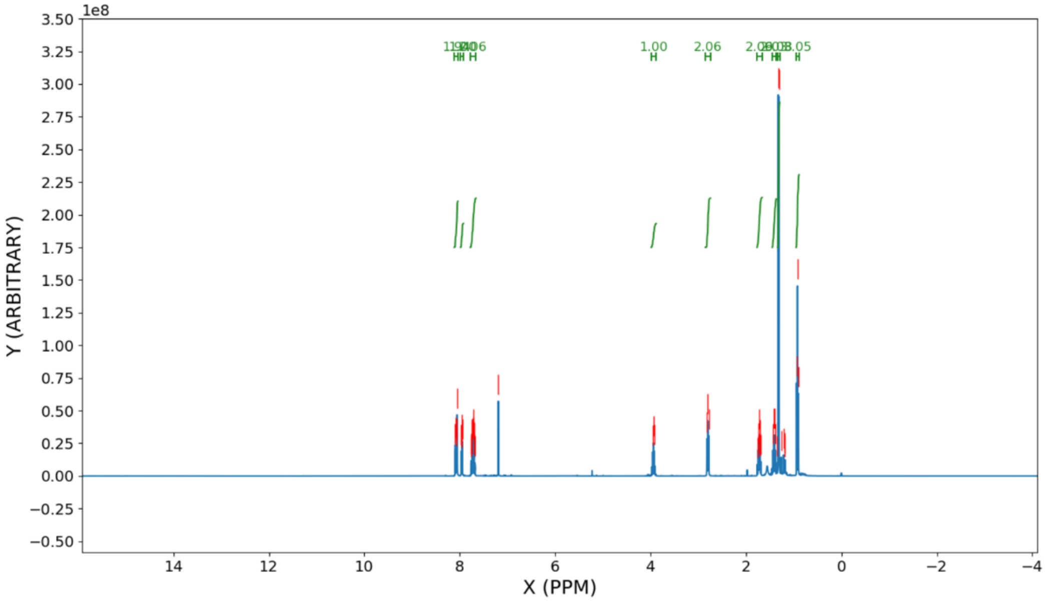

CHMO:0000595 | ^{13}C nuclear magnetic resonance spectroscopy (^{13}C NMR)

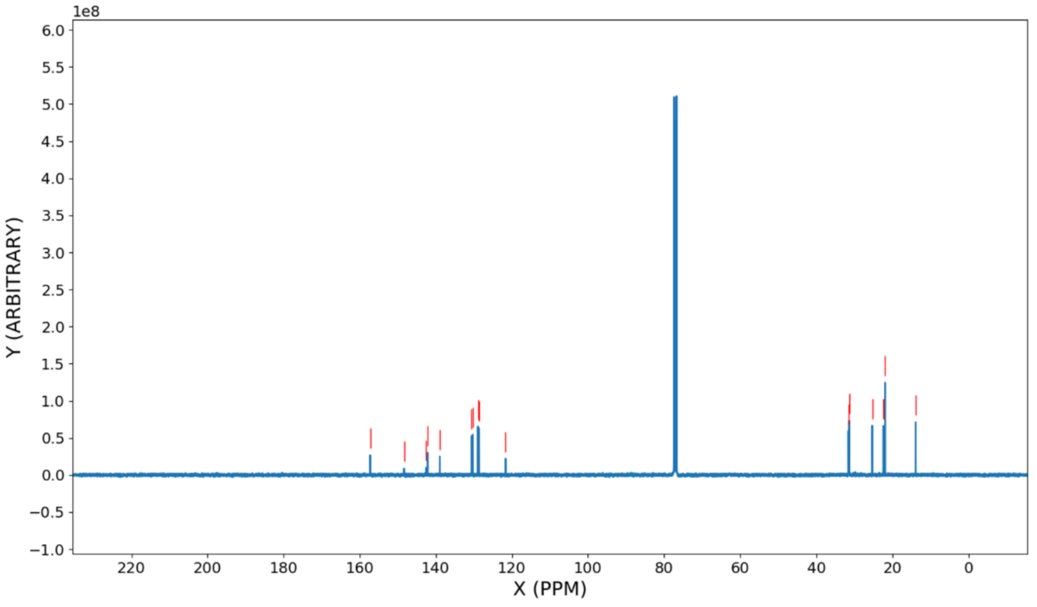

[**16b**] 1-Butyl-4-isopropylimidazo[1,2-*a*]quinoxaline

CHMO:0000593 | ^{1}H nuclear magnetic resonance spectroscopy (^{1}H NMR)

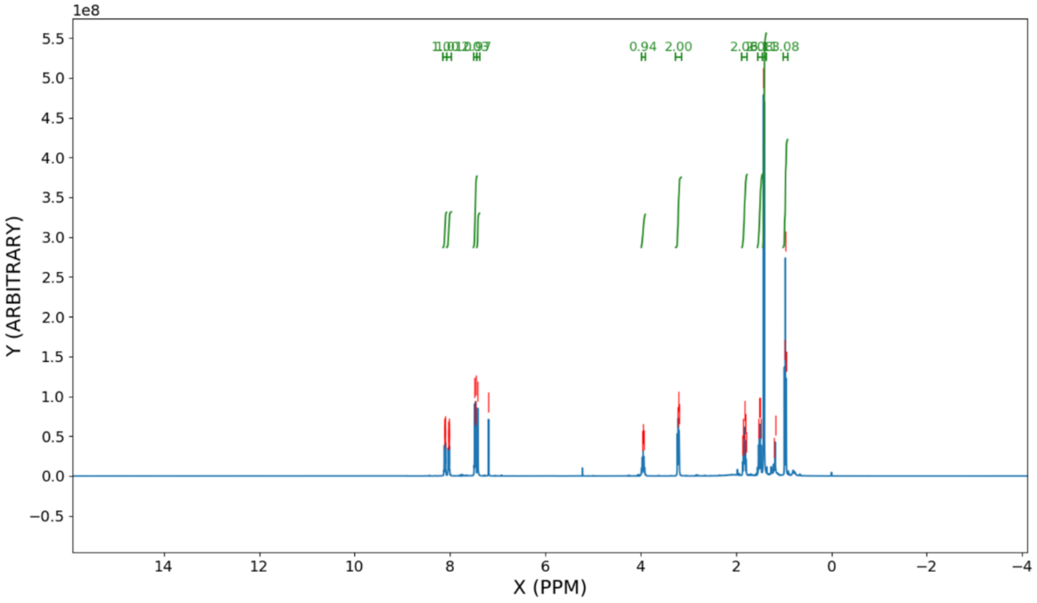

CHMO:0000595 | ^{13}C nuclear magnetic resonance spectroscopy (^{13}C NMR)

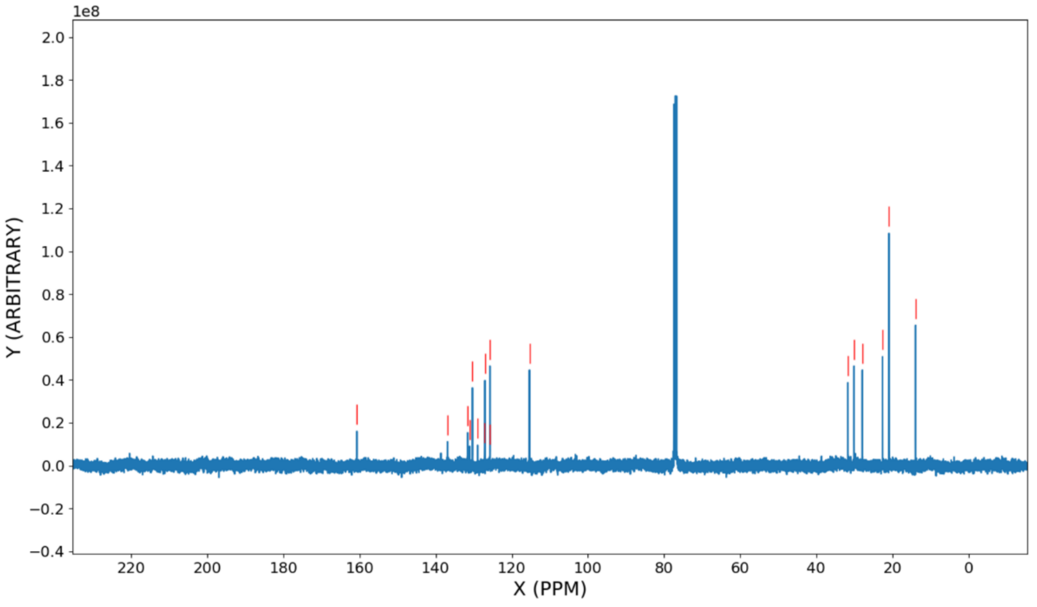

[**17b**] 3-Propan-2-ylquinoxalin-2-amine

CHMO:0000593 | ^{1}H nuclear magnetic resonance spectroscopy (^{1}H NMR)

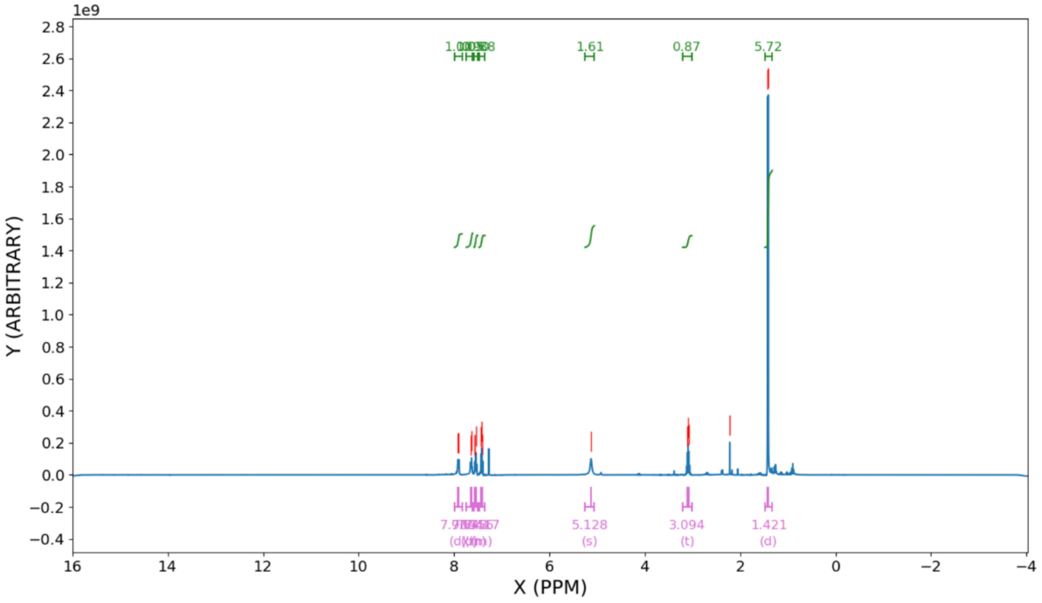

CHMO:0000595 | ^{13}C nuclear magnetic resonance spectroscopy (^{13}C NMR)

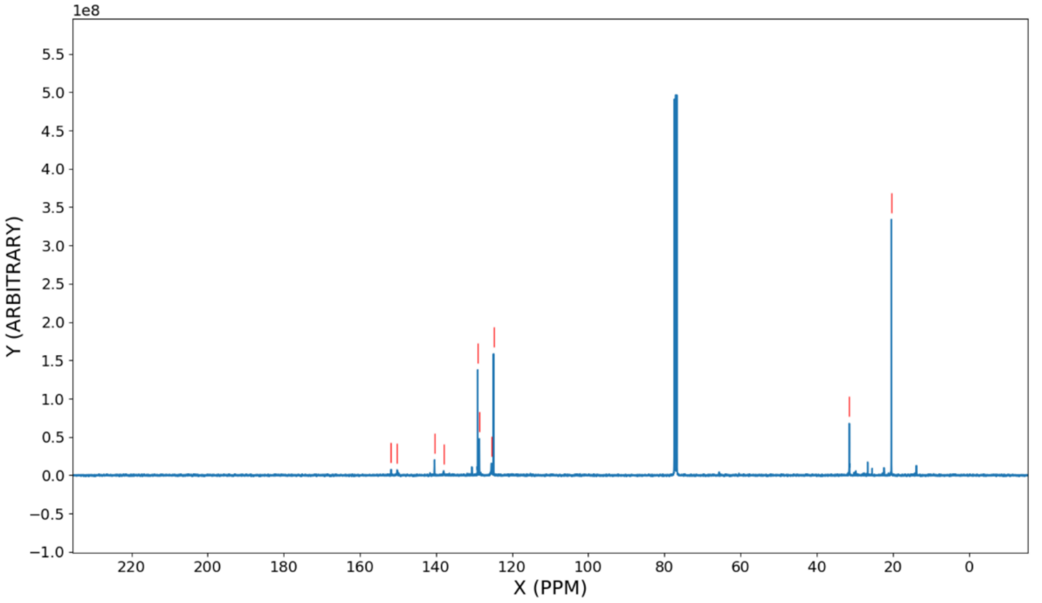

[**16c**] 1-Butyl-4-(trifluoromethyl)imidazo[1,2-*a*]quinoxaline

CHMO:0000593 | ^{1}H nuclear magnetic resonance spectroscopy (^{1}H NMR)

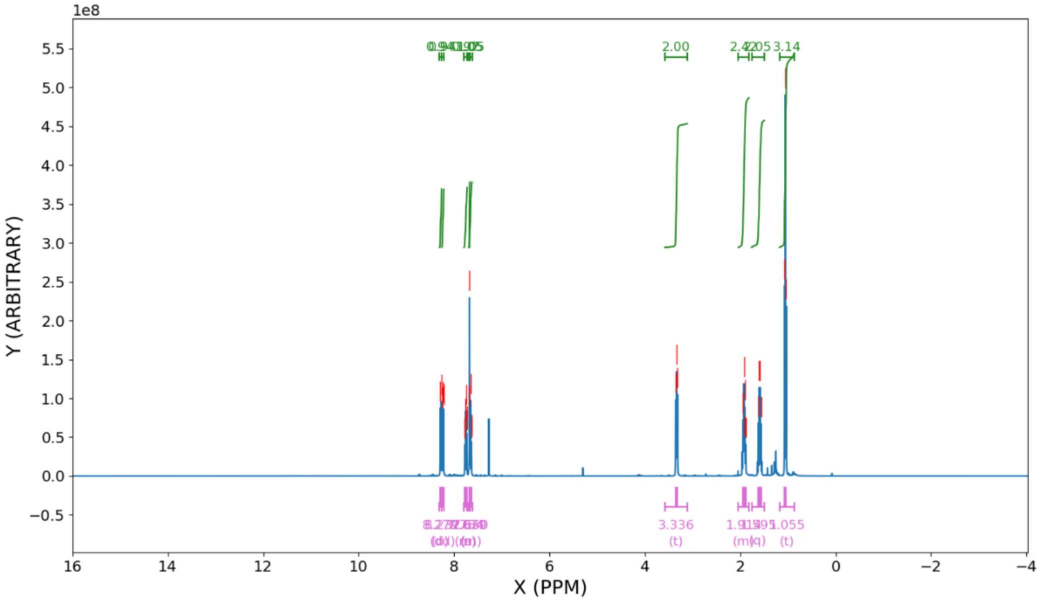

CHMO:0000595 | ^{13}C nuclear magnetic resonance spectroscopy (^{13}C NMR)

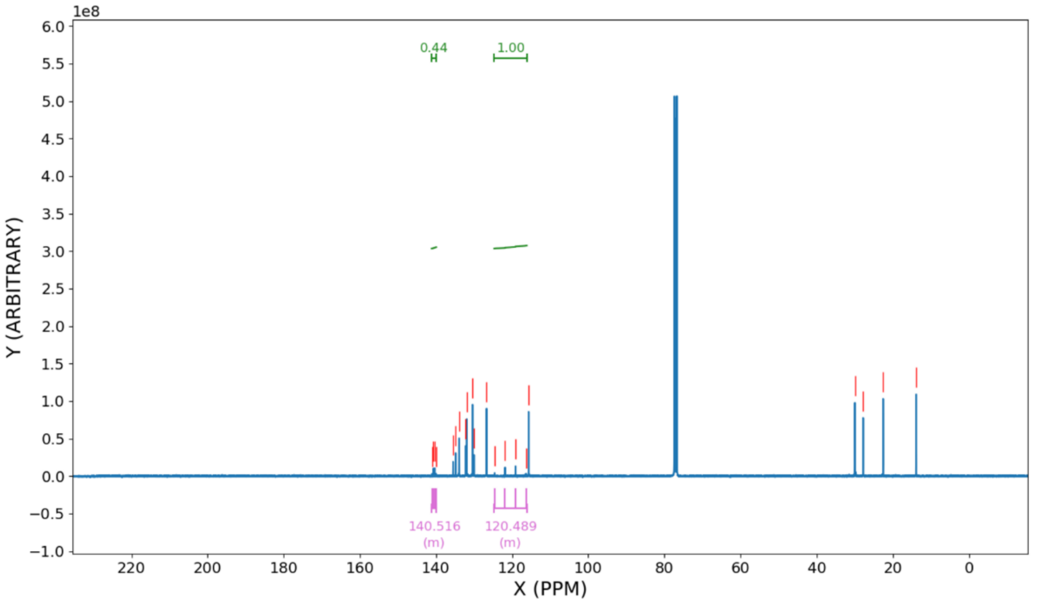

CHMO:0000597 | ^{19}F nuclear magnetic resonance spectroscopy (^{19}F NMR)

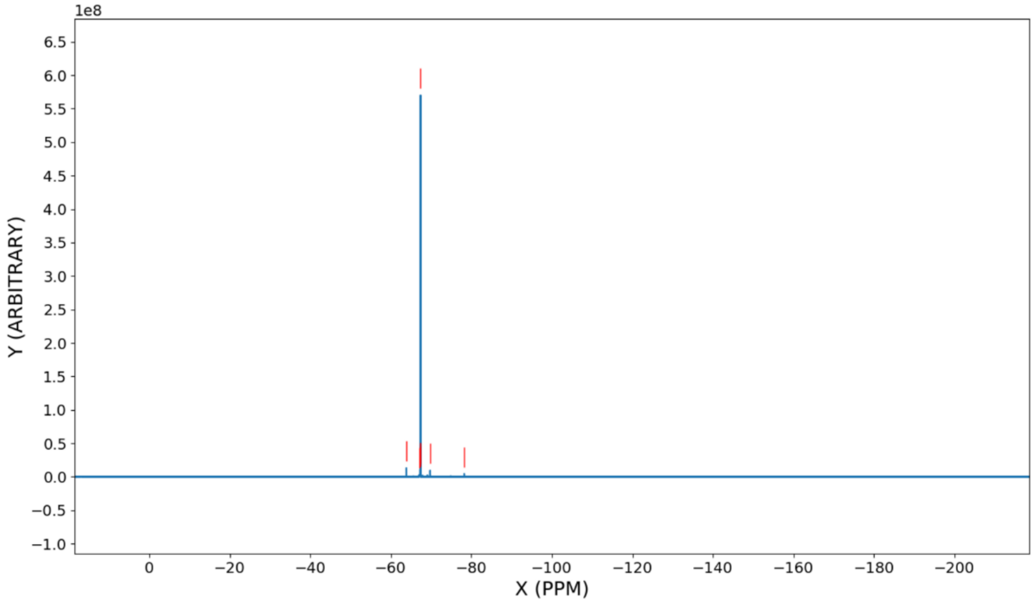

[**17c**] 3-(Trifluoromethyl)quinoxalin-2-amine

CHMO:0000593 | ^{1}H nuclear magnetic resonance spectroscopy (^{1}H NMR)

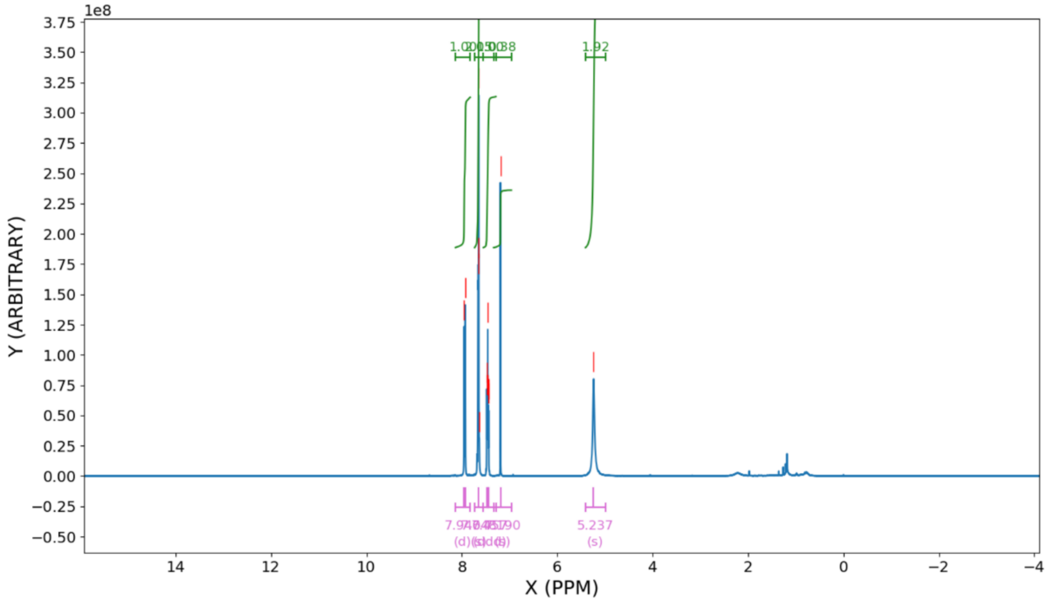

CHMO:0000595 | ^{13}C nuclear magnetic resonance spectroscopy (^{13}C NMR)

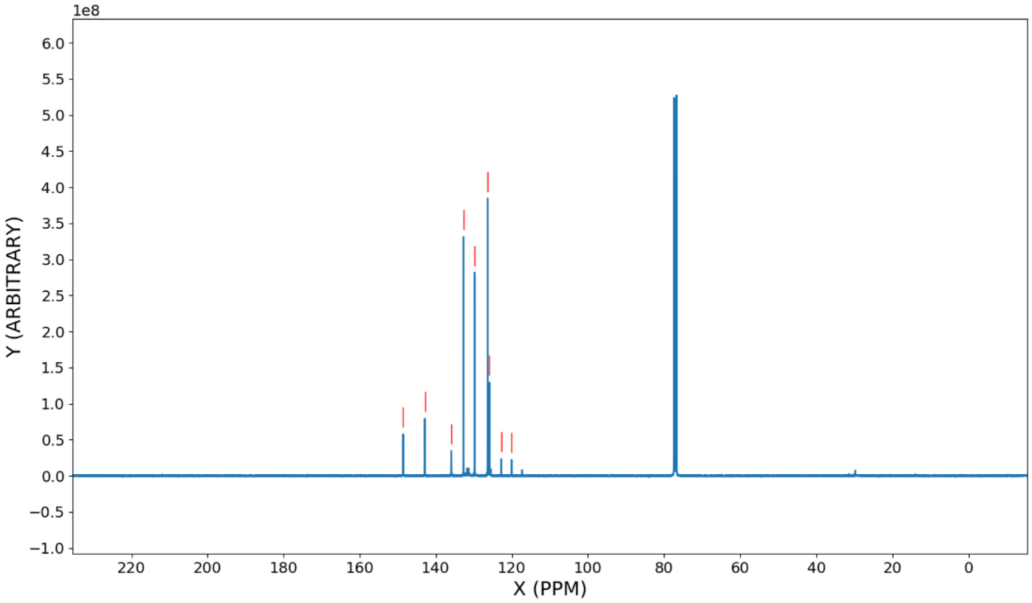

CHMO:0000597 | ^{19}F nuclear magnetic resonance spectroscopy (^{19}F NMR)

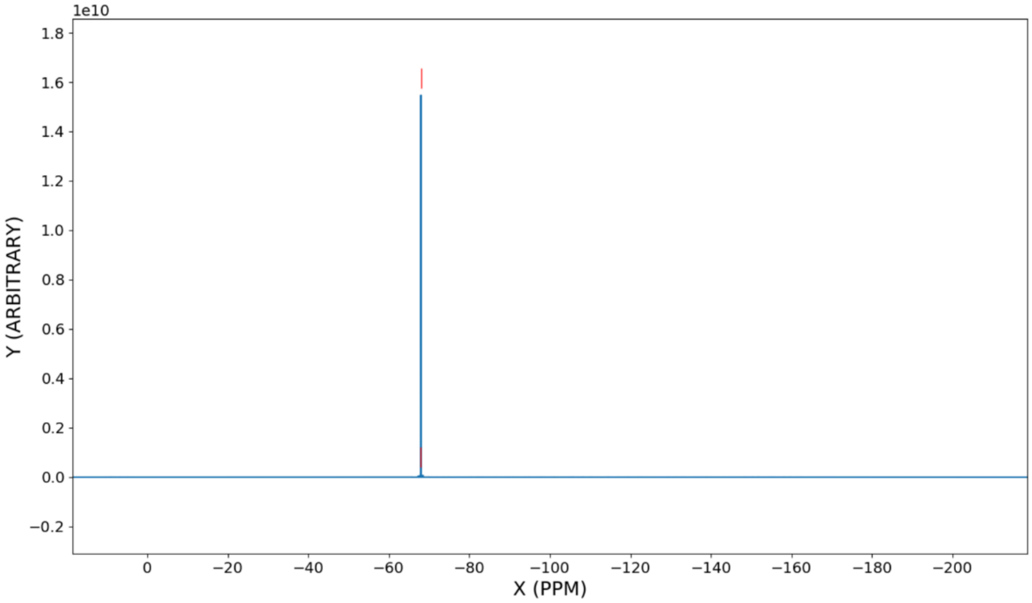

[**15d**] 2-(4-Butyl-1*H*-1,2,3-triazol-1-yl)-3-phenylquinoxaline

CHMO:0000593 | ^{1}H nuclear magnetic resonance spectroscopy (^{1}H NMR)

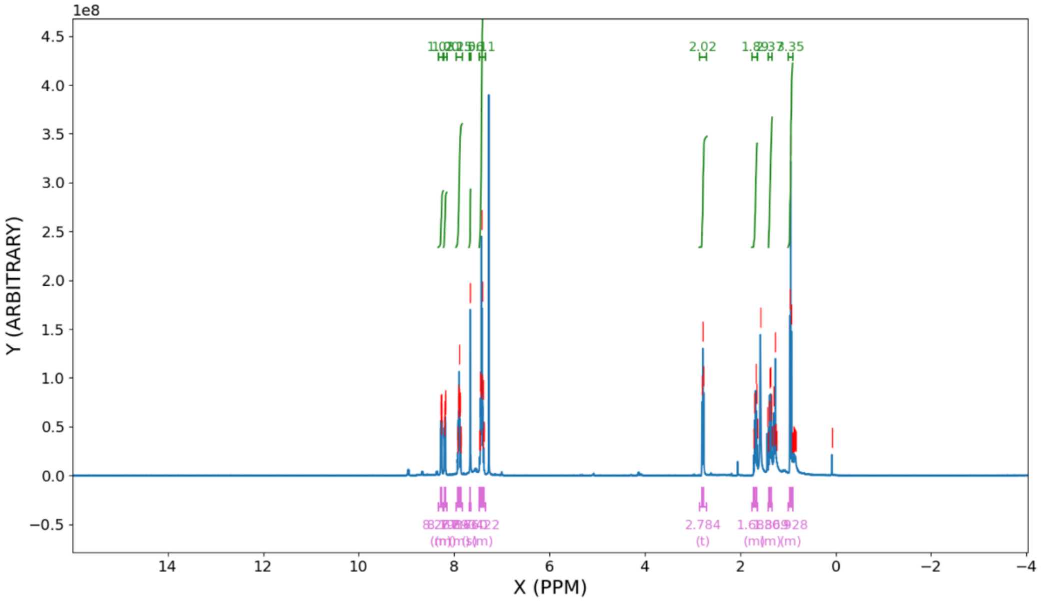

CHMO:0000595 | ^{13}C nuclear magnetic resonance spectroscopy (^{13}C NMR)

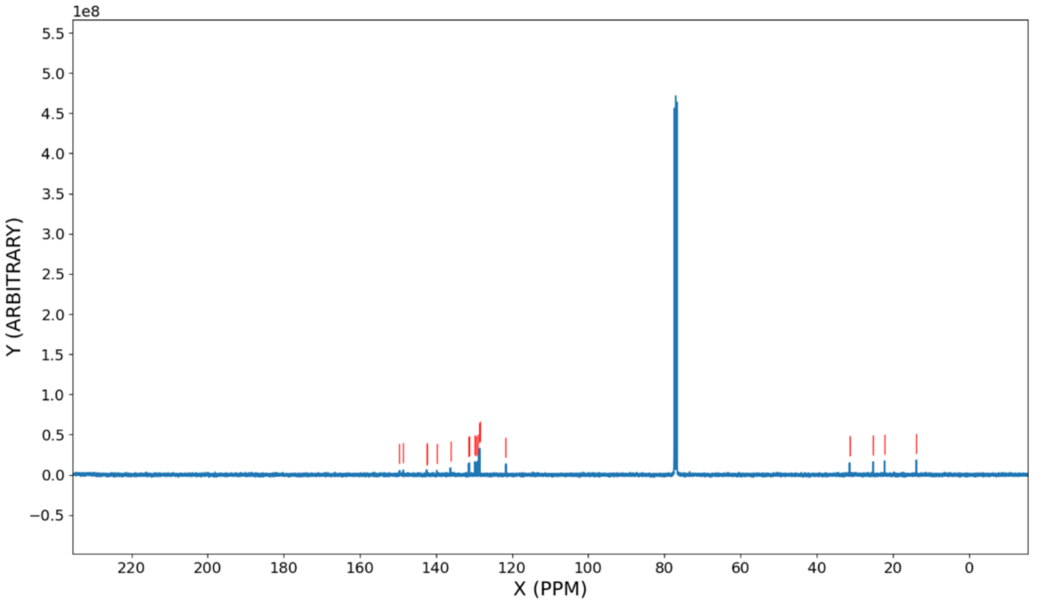

[**17d**] 3-Phenylquinoxalin-2-amine

CHMO:0000593 | ^{1}H nuclear magnetic resonance spectroscopy (^{1}H NMR)

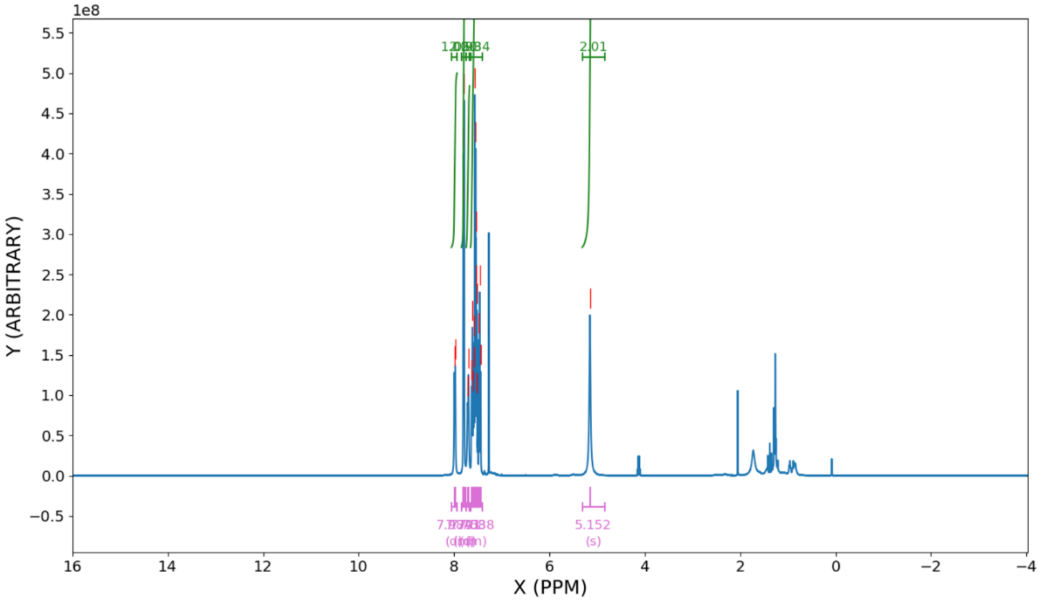

CHMO:0000595 | ^{13}C nuclear magnetic resonance spectroscopy (^{13}C NMR)

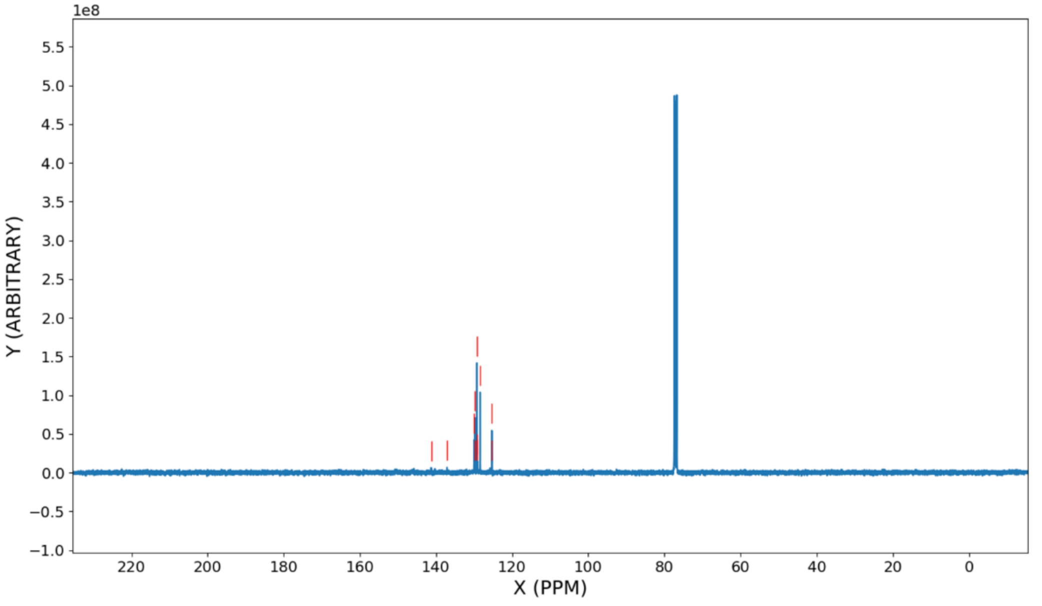

[**16e**] 1-Butyl-4-chloroimidazo[1,2-*a*]quinoxaline

CHMO:0000593 | ^{1}H nuclear magnetic resonance spectroscopy (^{1}H NMR)

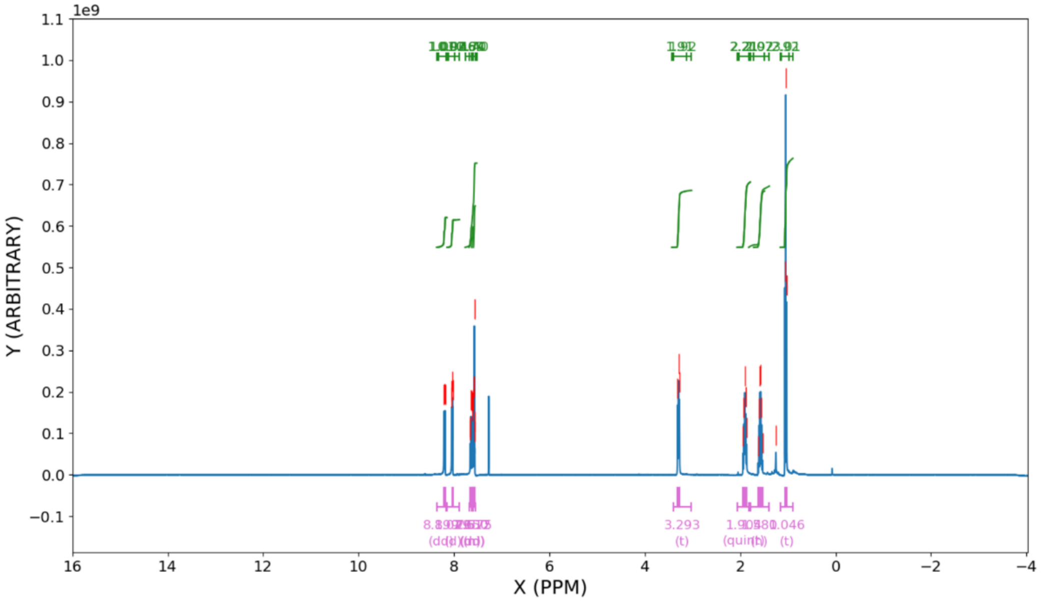

CHMO:0000595 | ^{13}C nuclear magnetic resonance spectroscopy (^{1}3C NMR)

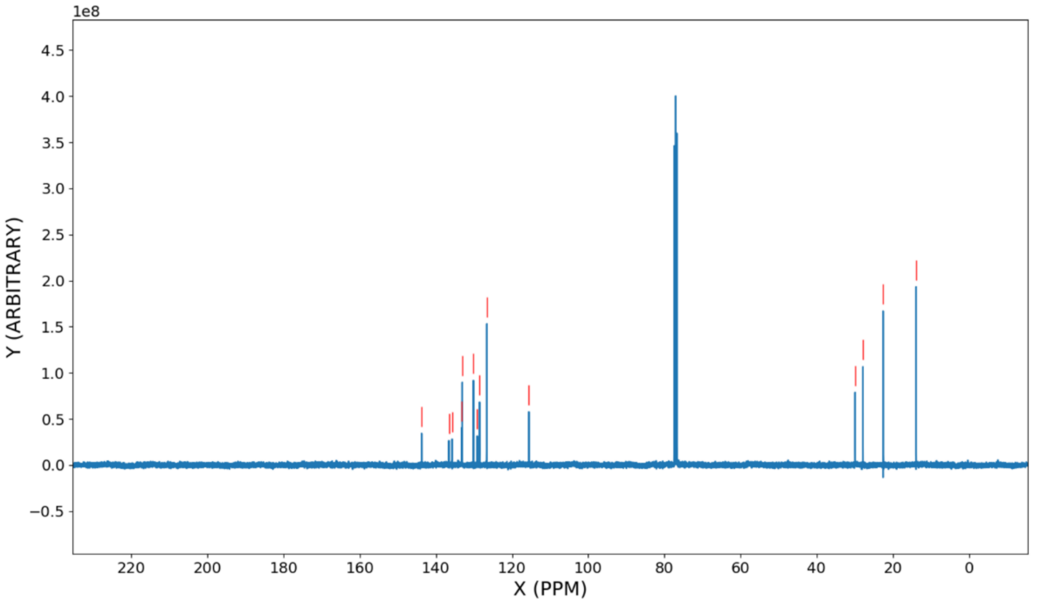

[**17e**] 3-Chloroquinoxalin-2-amine

CHMO:0000593 | ^{1}H nuclear magnetic resonance spectroscopy (^{1}H NMR)

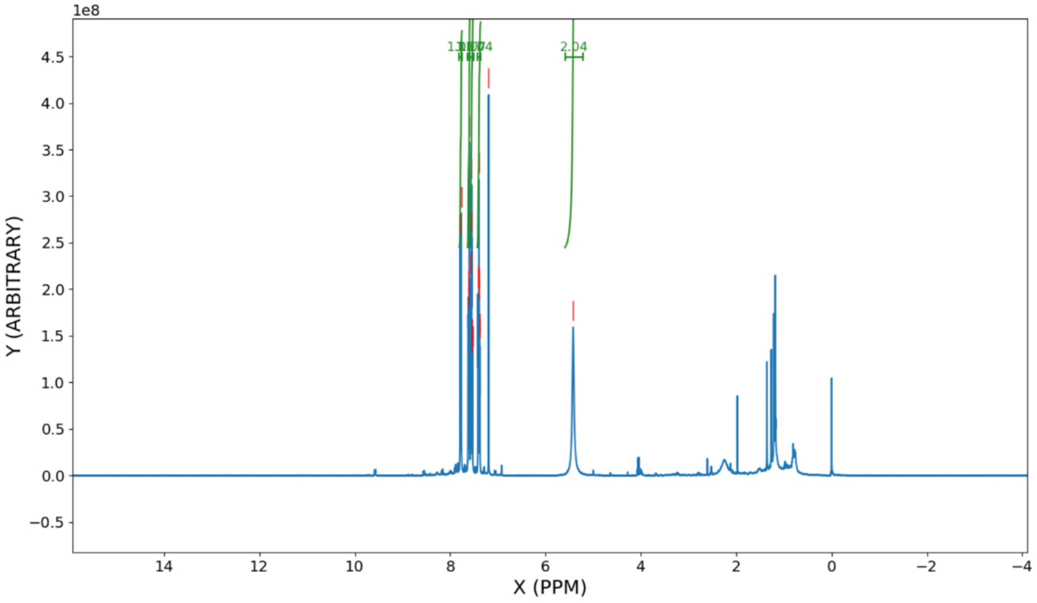

[**15f**] 2-(4-Butyl-1*H*-1,2,3-triazol-1-yl)-3-methoxyquinoxaline

CHMO:0000593 | ^{1}H nuclear magnetic resonance spectroscopy (^{1}H NMR)

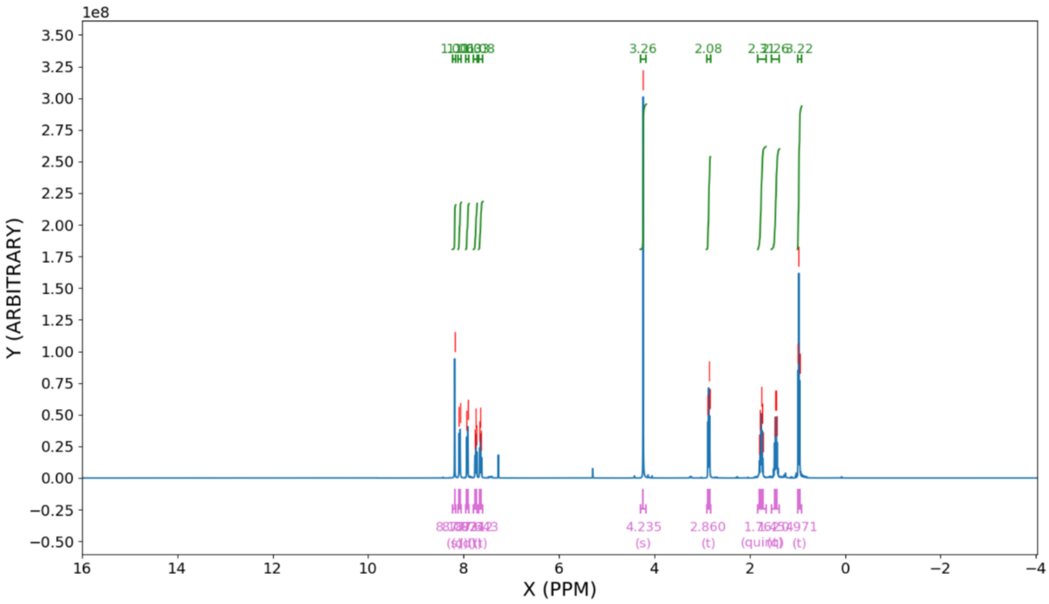

CHMO:0000595 | ^{13}C nuclear magnetic resonance spectroscopy (^{13}C NMR)

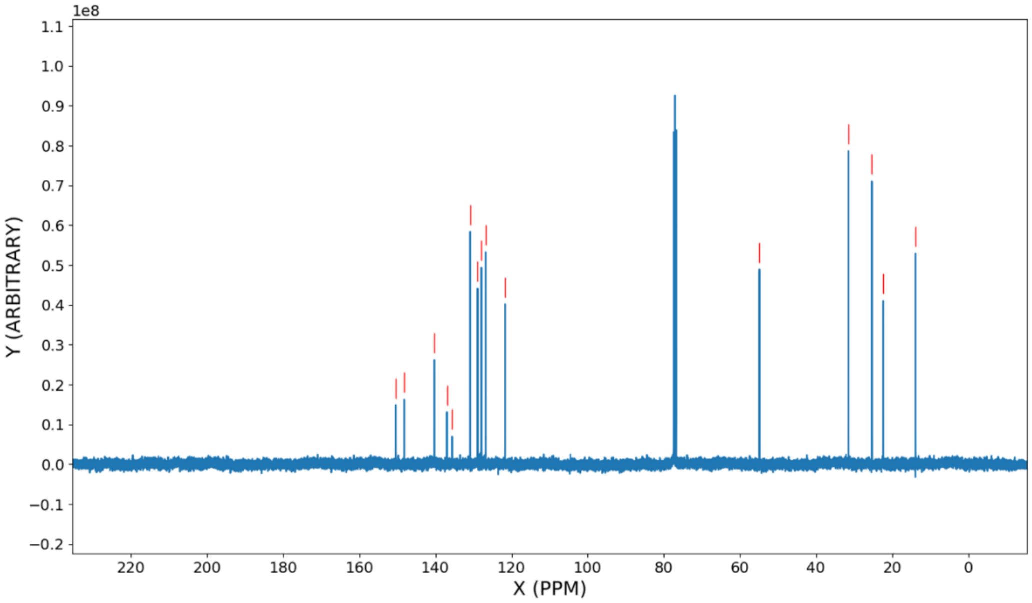

[**15g**] 1-(4-((3-(4-Butyl-1*H*-1,2,3-triazol-1-yl)quinoxalin-2-yl)amino)phenyl)ethan-1-one

CHMO:0000593 | *1*H nuclear magnetic resonance spectroscopy (*1*H NMR)

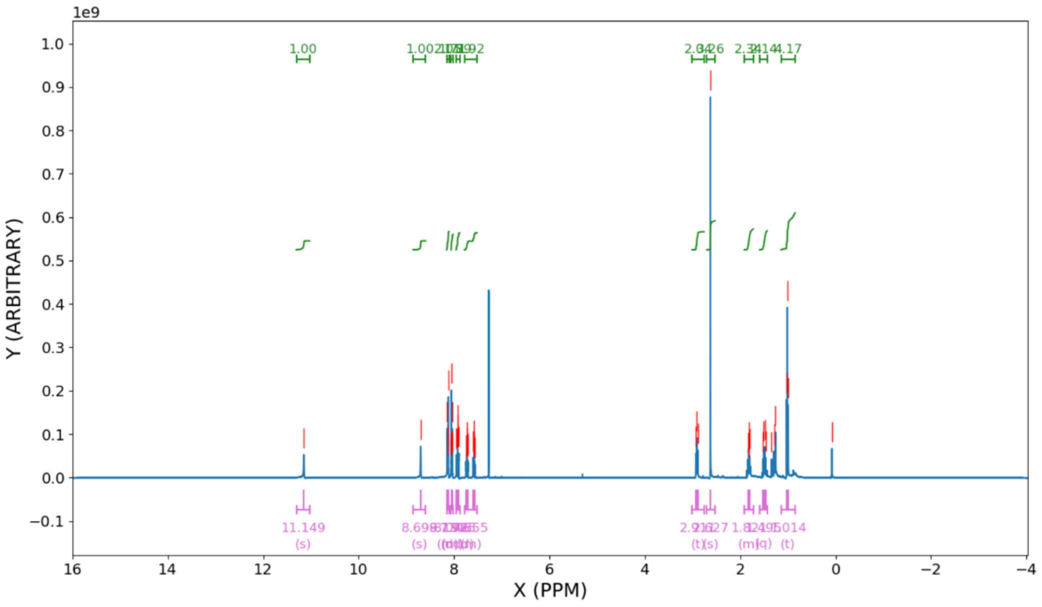

CHMO:0000595 | *13*C nuclear magnetic resonance spectroscopy (*13*C NMR)

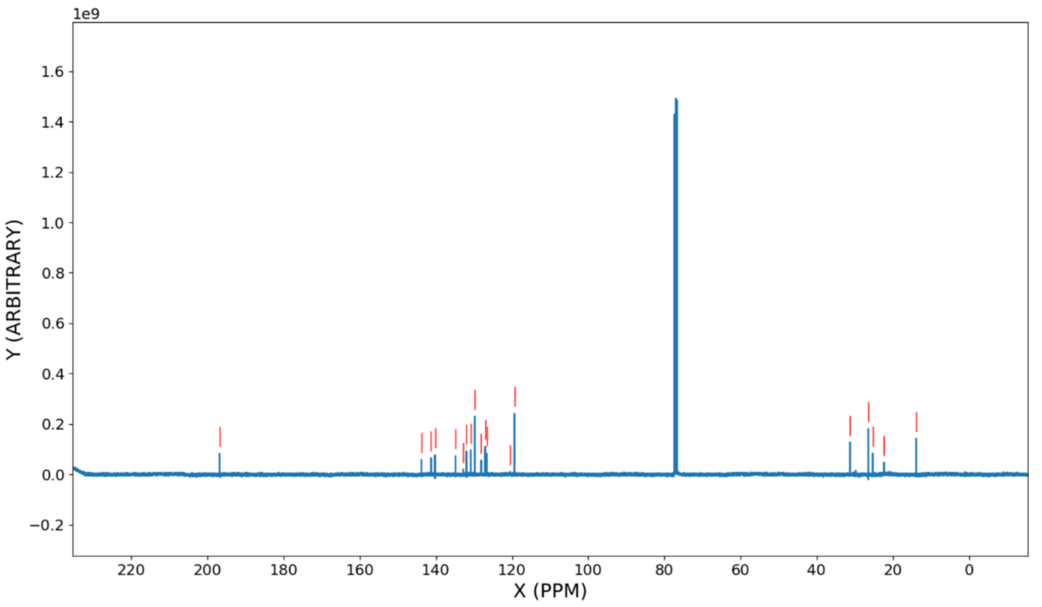

[**17g**] 1-(4-((3-Aminoquinoxalin-2-yl)amino)phenyl)ethan-1-one

CHMO:0000593 | ^{1}H nuclear magnetic resonance spectroscopy (^{1}H NMR)

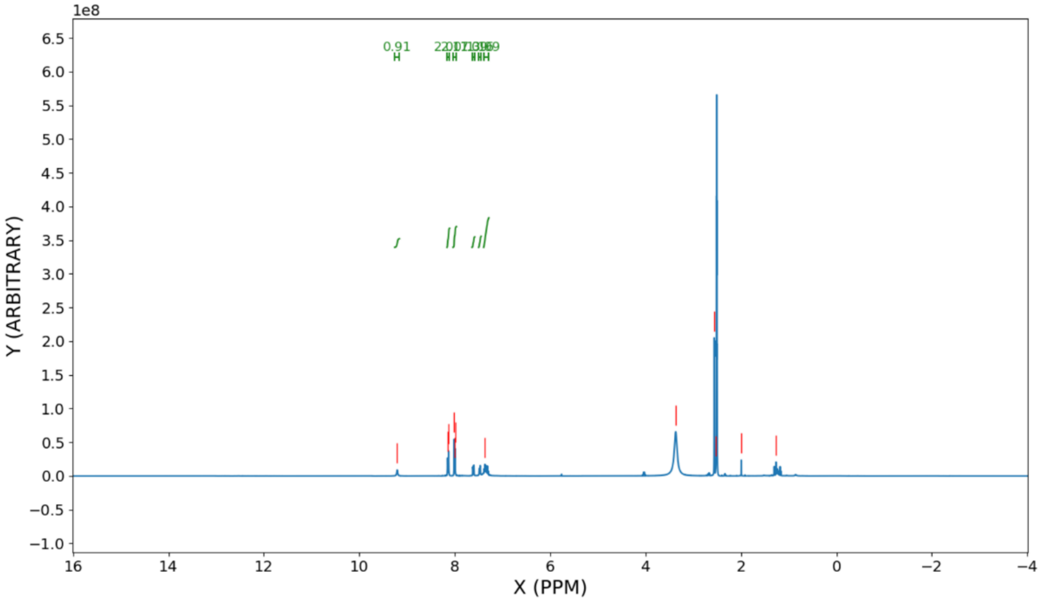

CHMO:0000595 | ^{13}C nuclear magnetic resonance spectroscopy (^{13}C NMR)

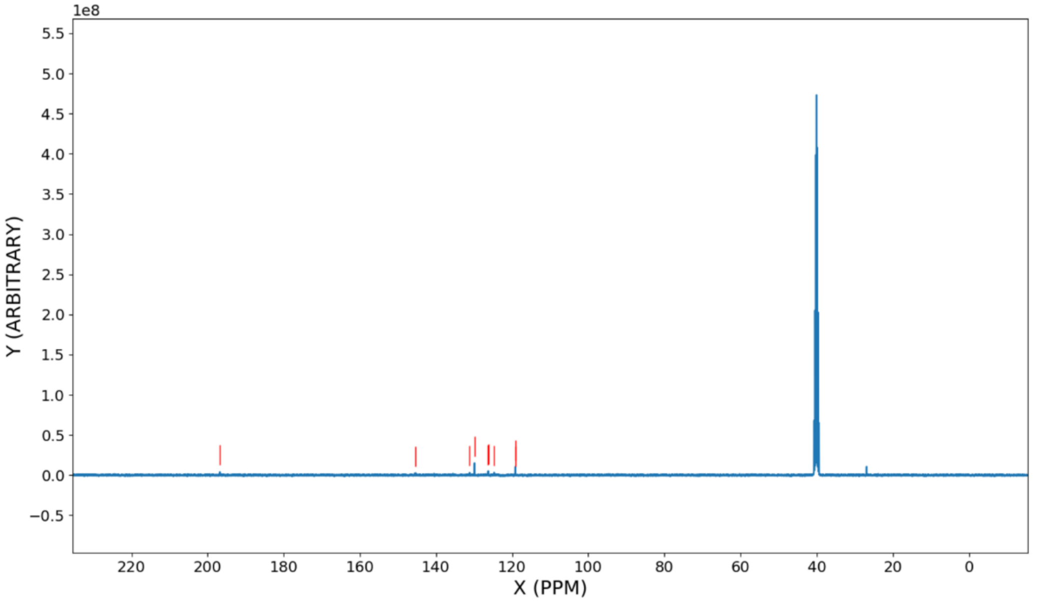

[**15h**] 2-(4-Butyl-1*H*-1,2,3-triazol-1-yl)-3-((3,3,4,4,5,5,6,6,7,7,8,8,9,9,10,10,10-heptadecafluorodecyl)oxy)quinoxaline

CHMO:0000593 | ^{1}H nuclear magnetic resonance spectroscopy (^{1}H NMR)

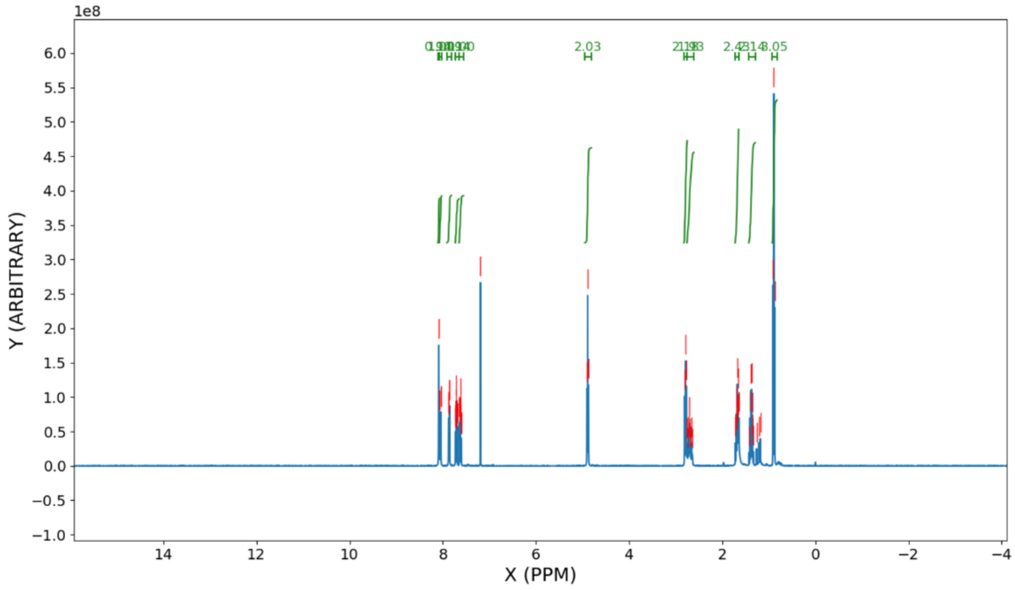

CHMO:0000595 | ^{13}C nuclear magnetic resonance spectroscopy (^{13}C NMR)

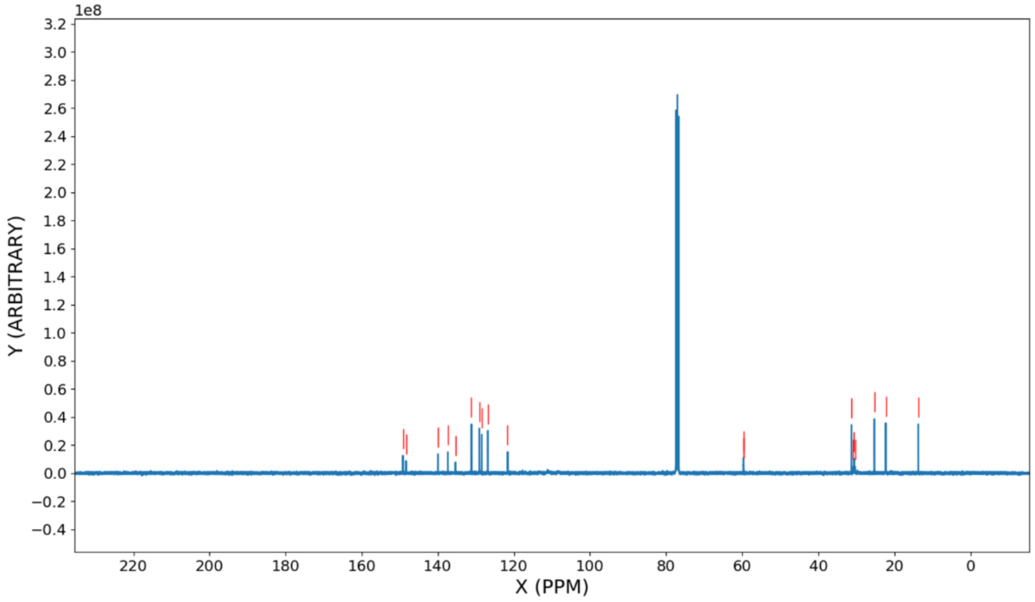

CHMO:0000597 | ^{19}F nuclear magnetic resonance spectroscopy (^{19}F NMR)

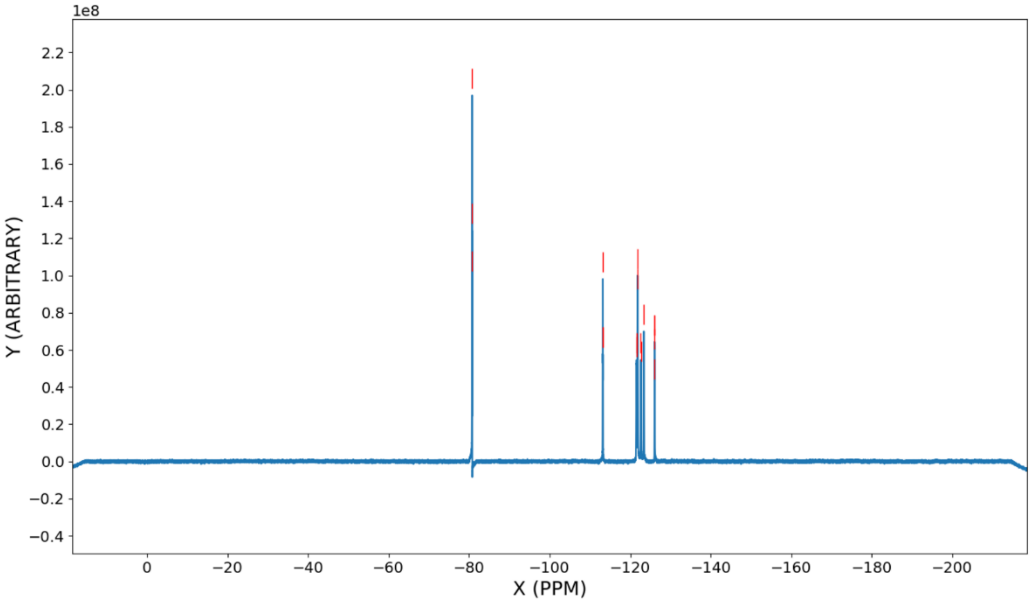

[**16h**] 1-Butyl-4-((3,3,4,4,5,5,6,6,7,7,8,8,9,9,10,10,10-heptadecafluorodecyl)oxy)imidazo[1,2-*a*]quinoxaline

CHMO:0000593 | ^{1}H nuclear magnetic resonance spectroscopy (^{1}H NMR)

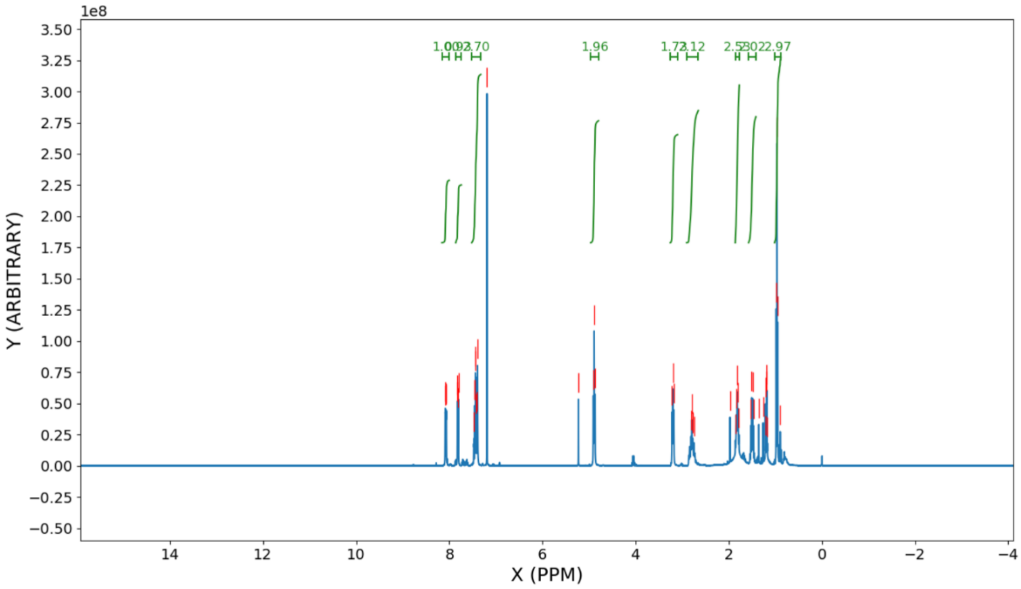

CHMO:0000595 | ^{13}C nuclear magnetic resonance spectroscopy (^{13}C NMR)

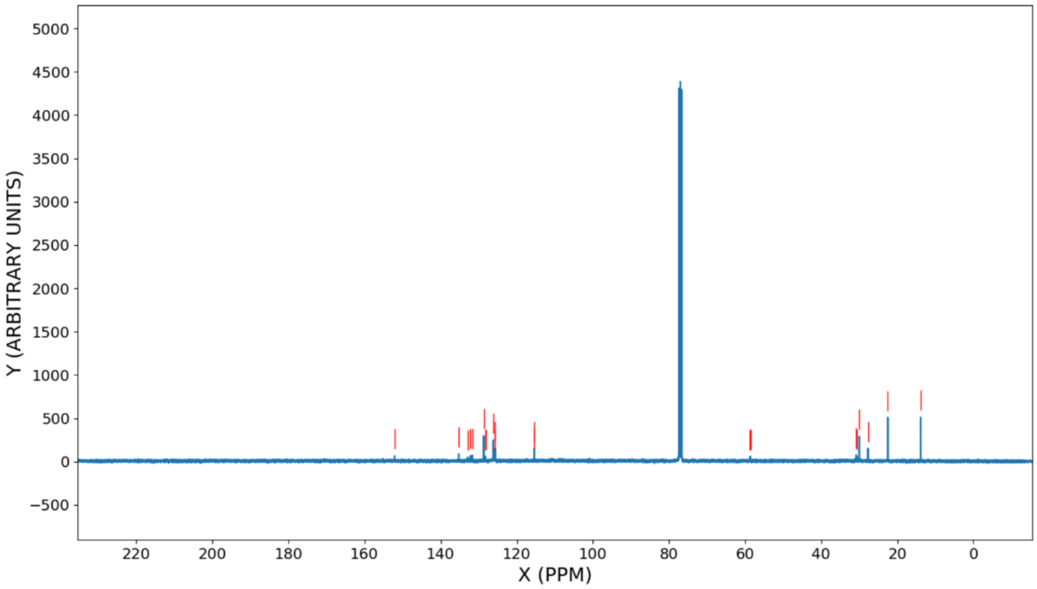

CHMO:0000597 | ^{19}F nuclear magnetic resonance spectroscopy (^{19}F NMR)

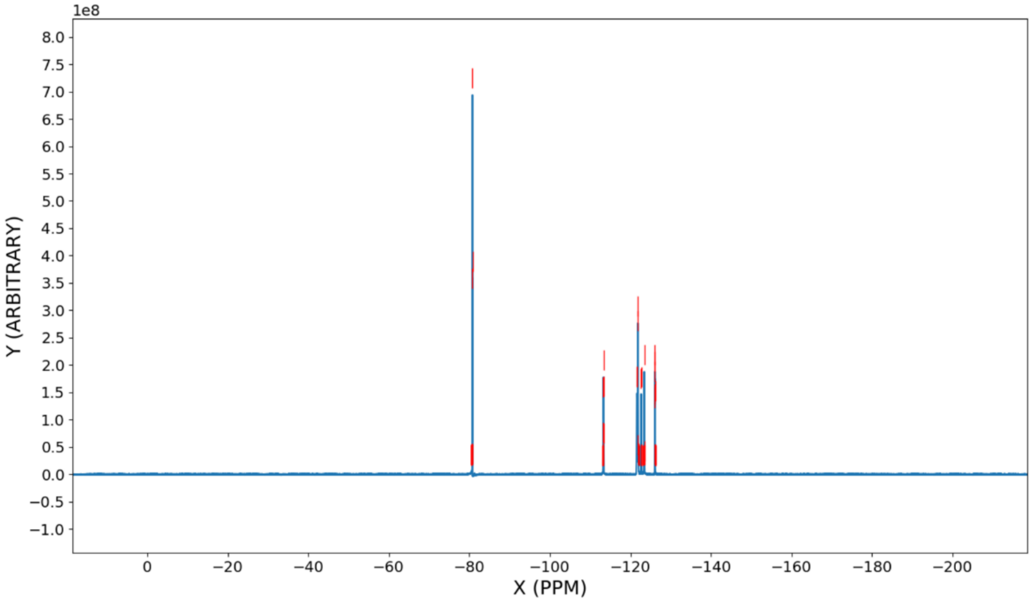

[**17h**] 3-((3,3,4,4,5,5,6,6,7,7,8,8,9,9,10,10,10-Heptadecafluorodecyl)oxy)quinoxalin-2-amine

CHMO:0000593 | ^{1}H nuclear magnetic resonance spectroscopy (^{1}H NMR)

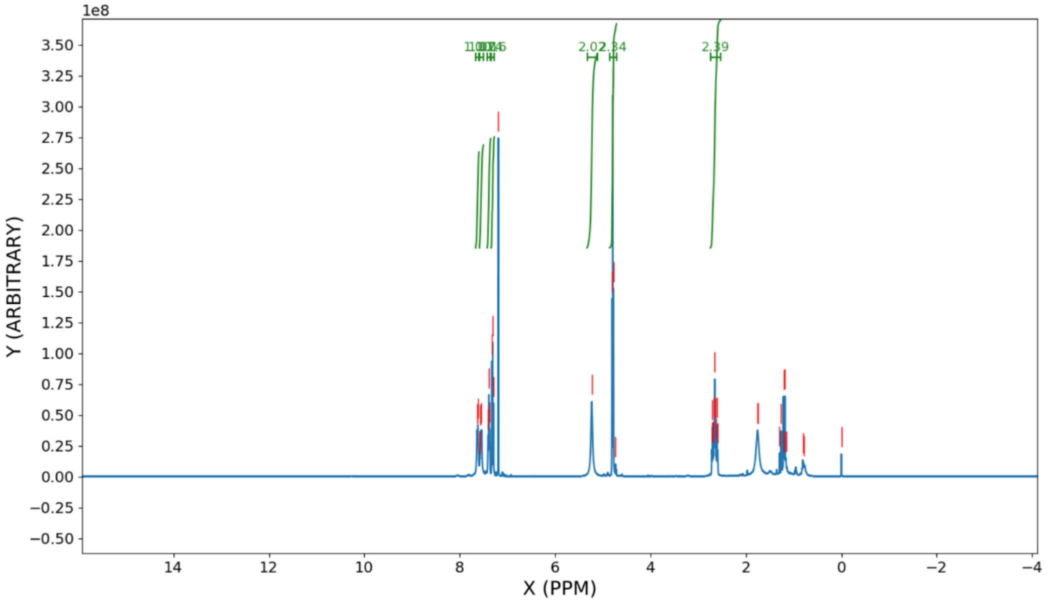

CHMO:0000595 | ^{13}C nuclear magnetic resonance spectroscopy (^{13}C NMR)

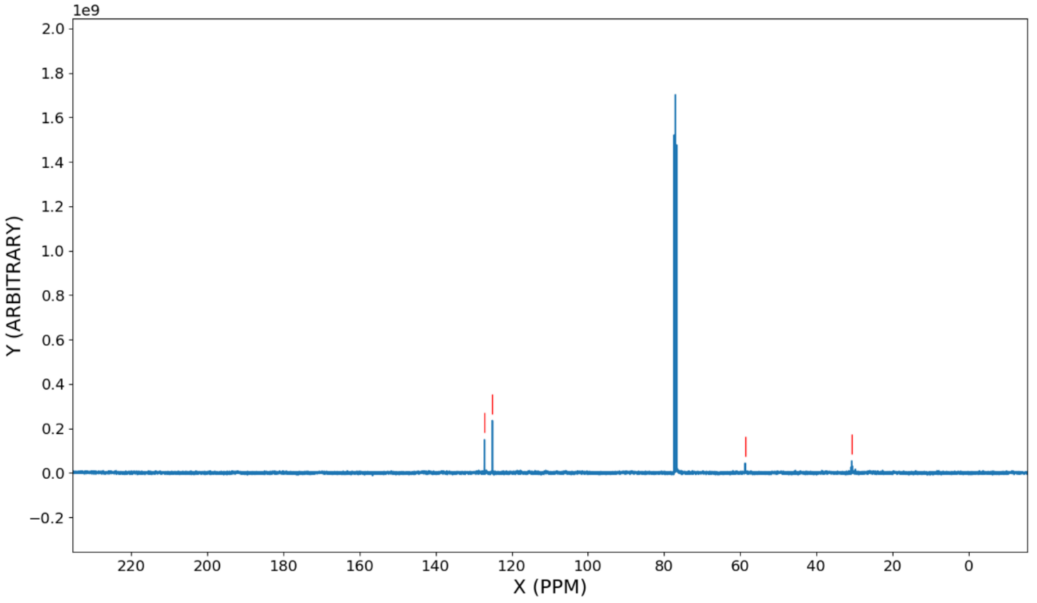

CHMO:0000597 | ^{19}F nuclear magnetic resonance spectroscopy (^{19}F NMR)

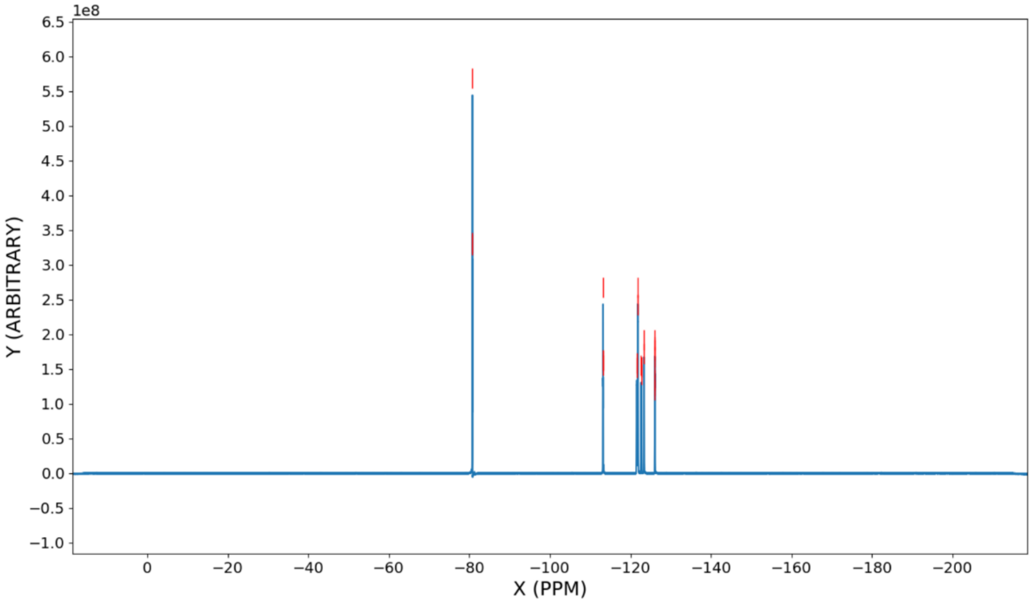

[**S9**] 1-Phenyl-4-(trifluoromethyl)imidazo[1,2-*a*]quinoxaline

CHMO:0000593 | ^{1}H nuclear magnetic resonance spectroscopy (^{1}H NMR)

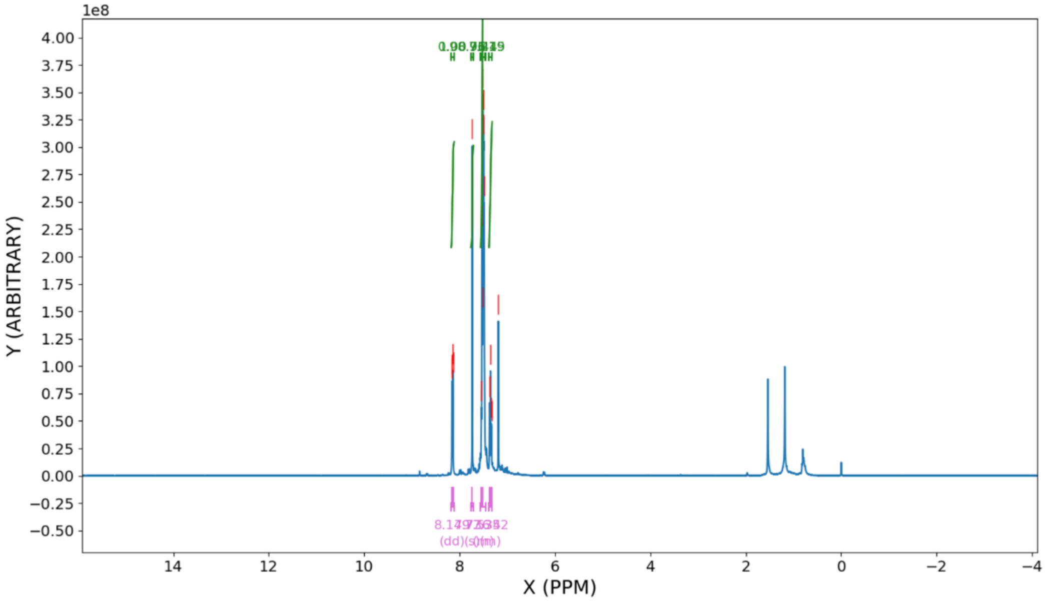

CHMO:0000595 | ^{13}C nuclear magnetic resonance spectroscopy (^{13}C NMR)

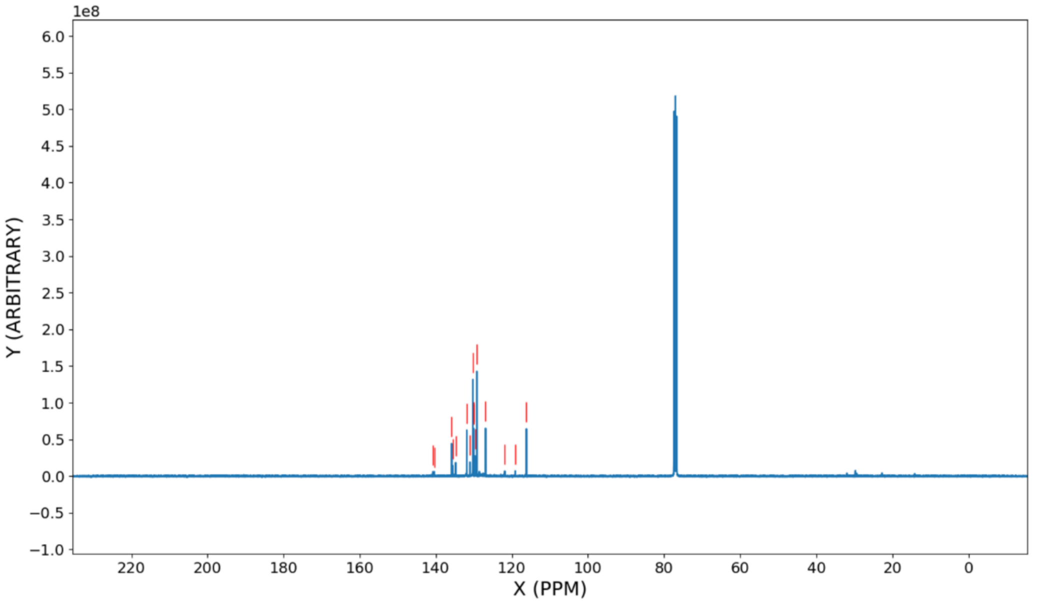

[**24**] Bis(tetrazolo)[1,5-*a*:5',1'-*c*]quinoxaline

CHMO:0000593 | ^{1}H nuclear magnetic resonance spectroscopy (^{1}H NMR)

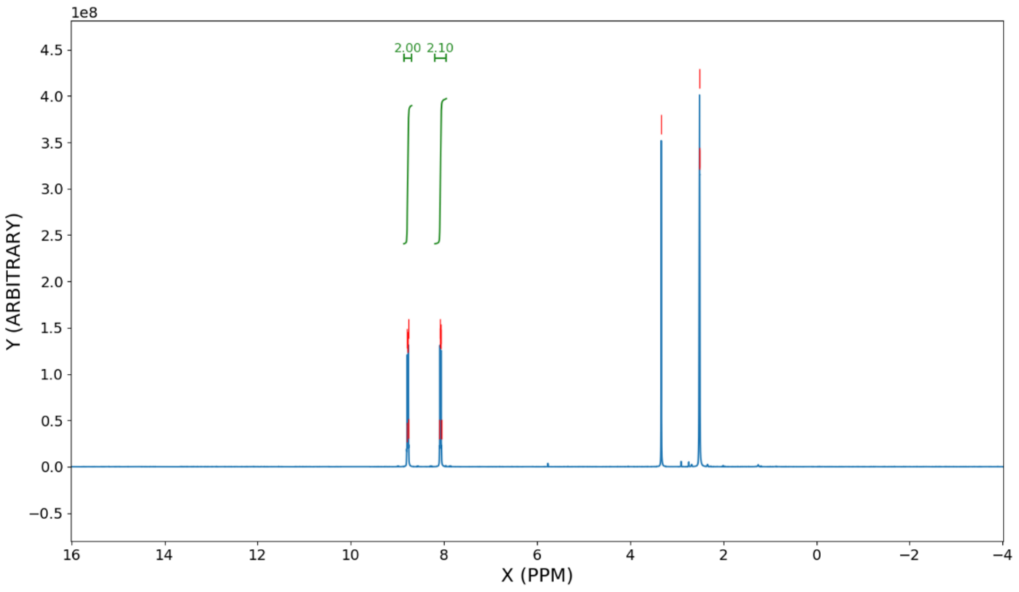

CHMO:0000595 | ^{13}C nuclear magnetic resonance spectroscopy (^{13}C NMR)

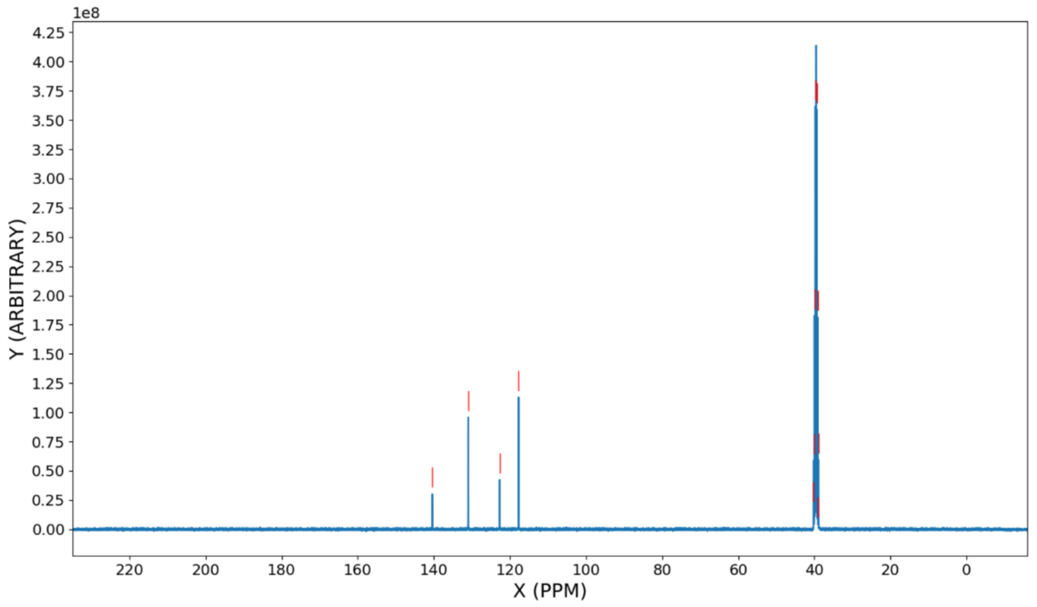

[**25a**] 1-Phenyl-4-(4-phenyl-1*H*-1,2,3-triazol-1-yl)imidazo[1,2-*a*]quinoxaline

CHMO:0000593 | ^{1}H nuclear magnetic resonance spectroscopy (^{1}H NMR)

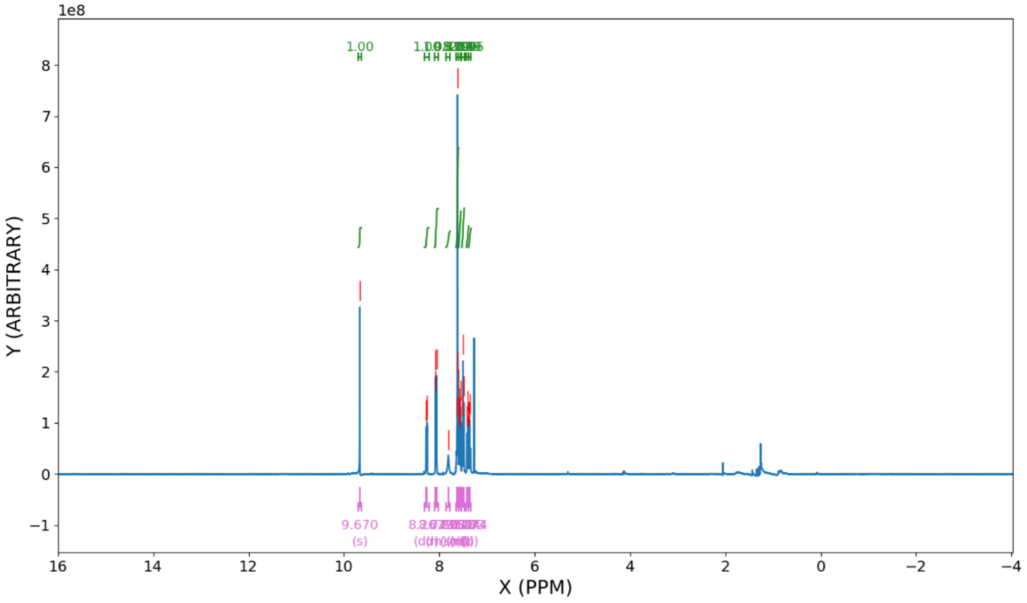

CHMO:0000595 | ^{13}C nuclear magnetic resonance spectroscopy (^{13}C NMR)

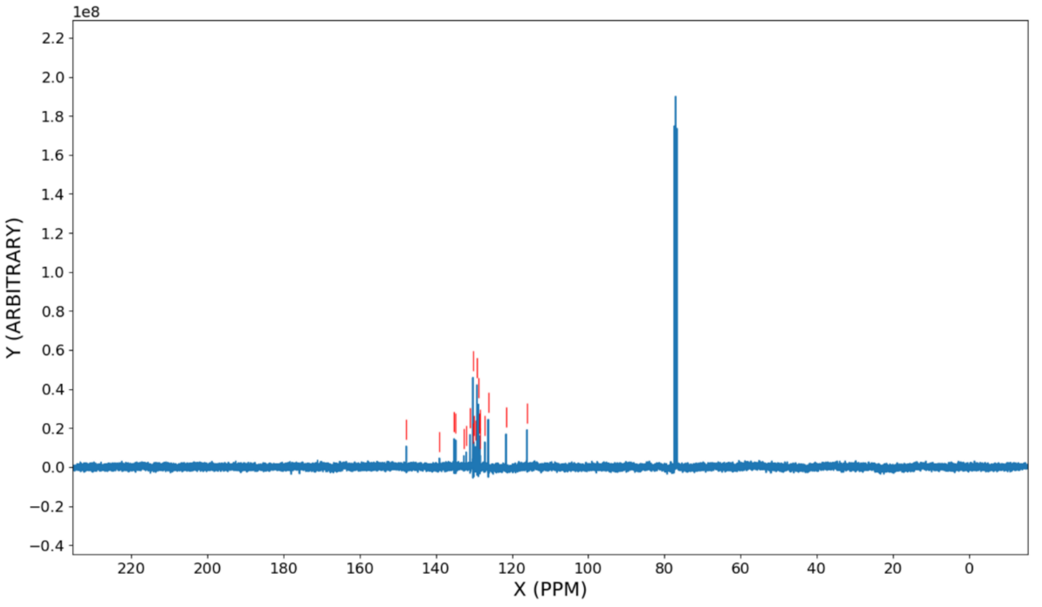

[**S5a**] 1-Phenylimidazo[1,2-*a*]quinoxalin-4-amine

CHMO:0000593 | ^{1}H nuclear magnetic resonance spectroscopy (^{1}H NMR)

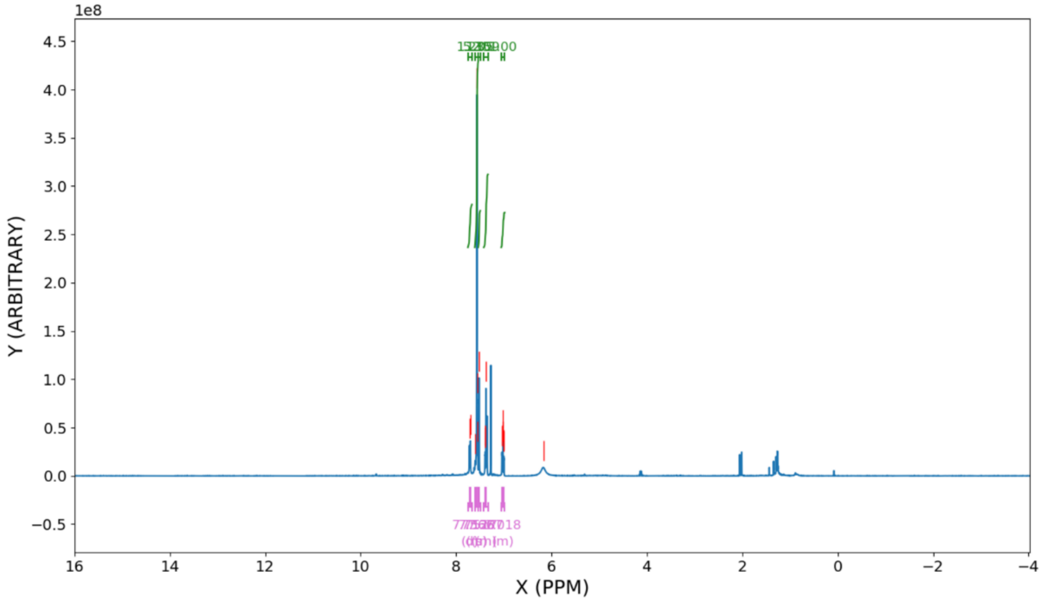

CHMO:0000595 | ^{13}C nuclear magnetic resonance spectroscopy (^{13}C NMR)

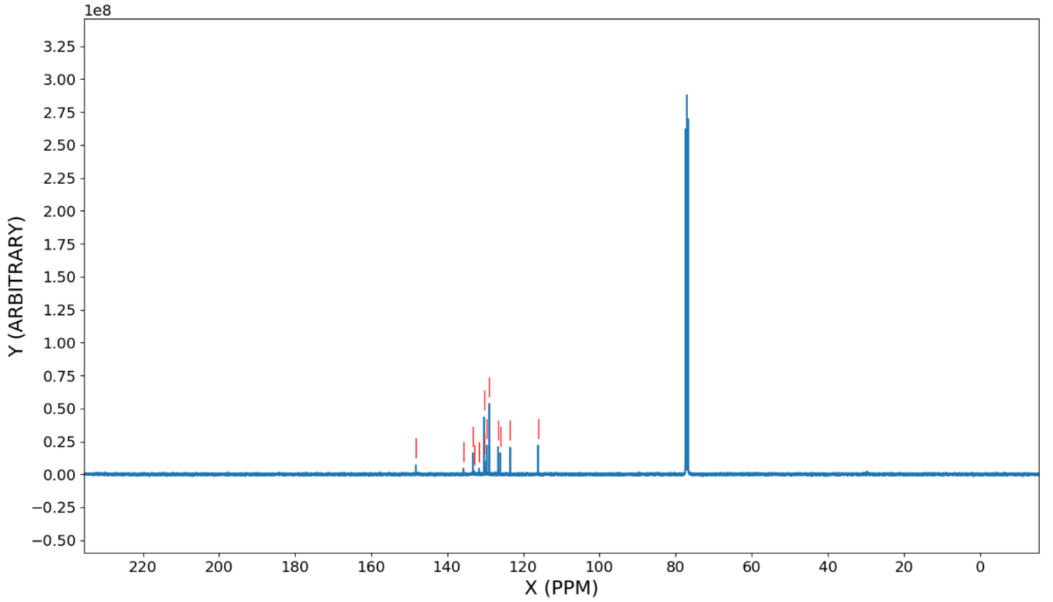

[**S6a**] 3-(4-Phenyl-1*H*-1,2,3-triazol-1-yl)quinoxalin-2-amine

CHMO:0000593 | ^{1}H nuclear magnetic resonance spectroscopy (^{1}H NMR)

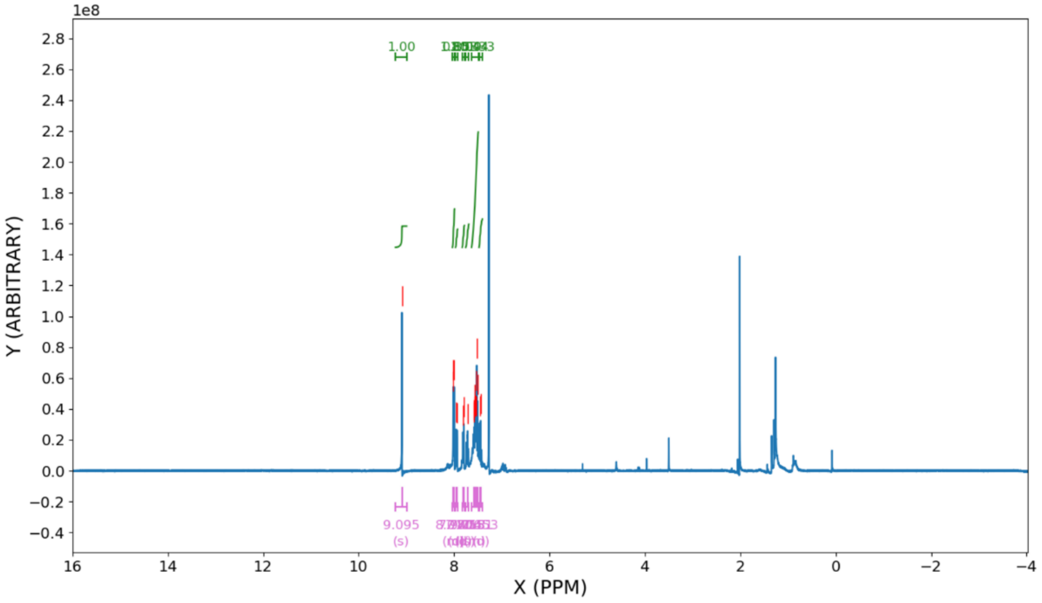

CHMO:0000595 | ^{13}C nuclear magnetic resonance spectroscopy (^{13}C NMR)

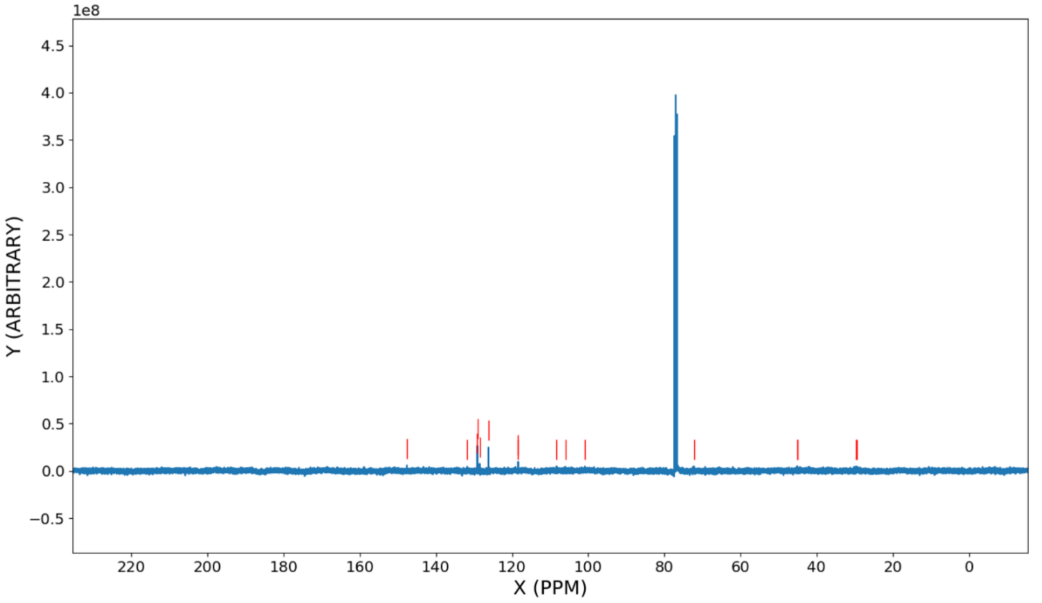

[**25b**] 1-Butyl-4-(4-butyl-1*H*-1,2,3-triazol-1-yl)imidazo[1,2-*a*]quinoxaline

CHMO:0000593 | ^{1}H nuclear magnetic resonance spectroscopy (^{1}H NMR)

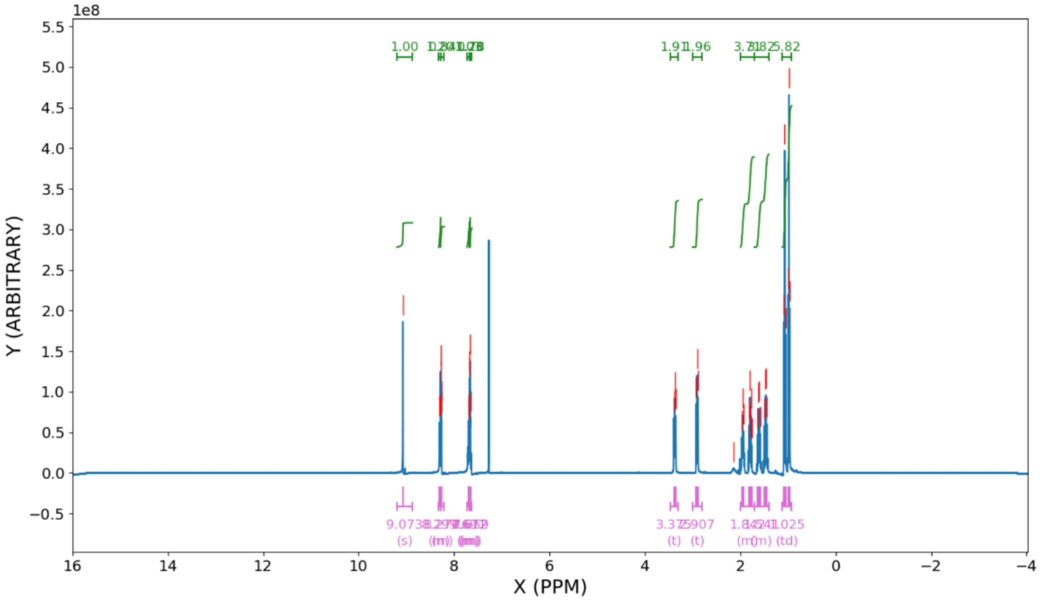

CHMO:0000595 | ^{13}C nuclear magnetic resonance spectroscopy (^{13}C NMR)

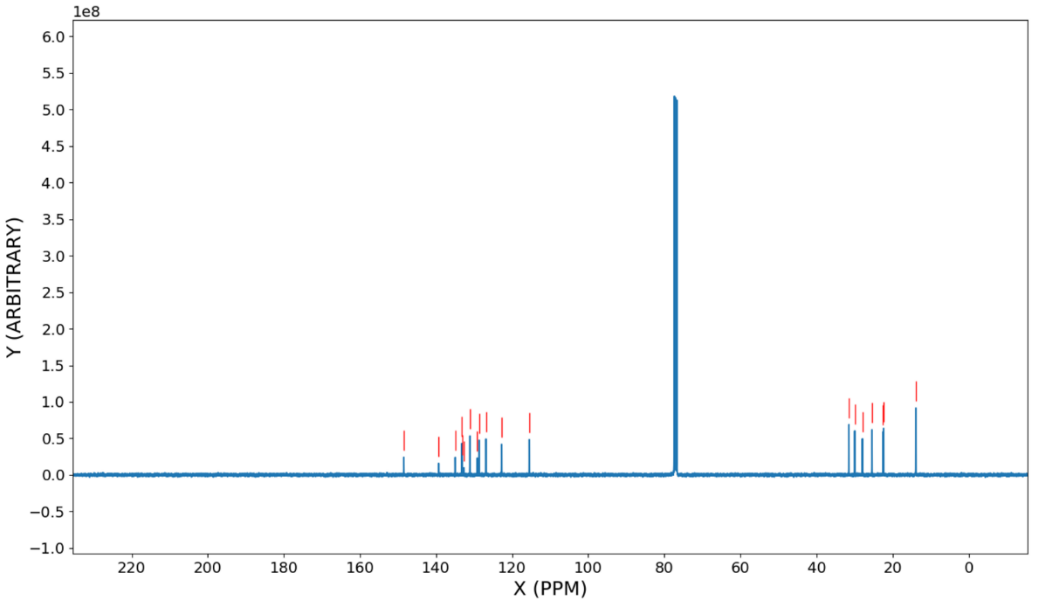

[**S5b**] 1-Butylimidazo[1,2-*a*]quinoxalin-4-amine

CHMO:0000593 | ^{1}H nuclear magnetic resonance spectroscopy (^{1}H NMR)

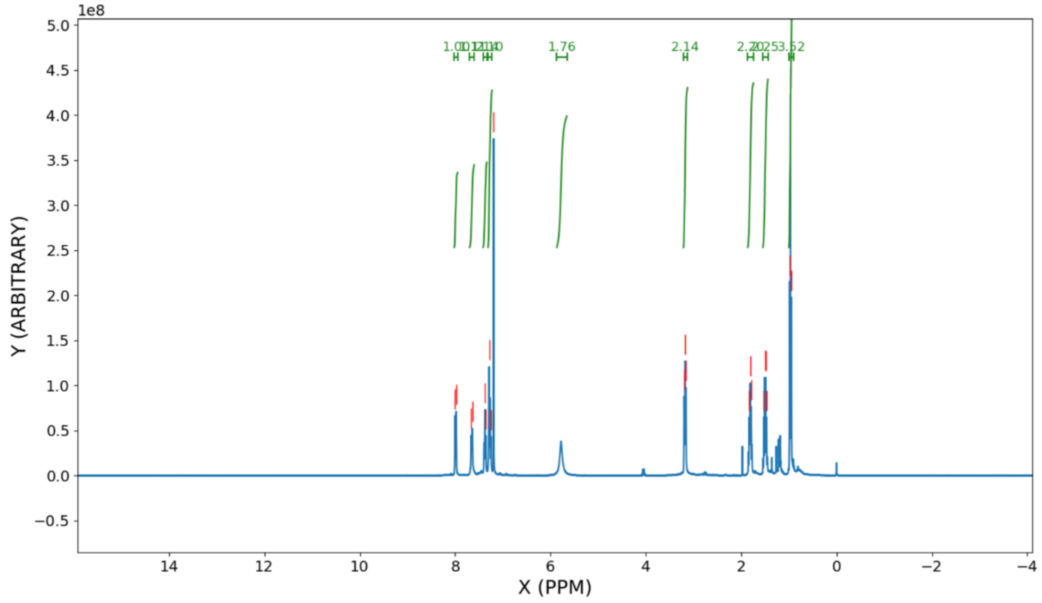

CHMO:0000595 | ^{13}C nuclear magnetic resonance spectroscopy (^{13}C NMR)

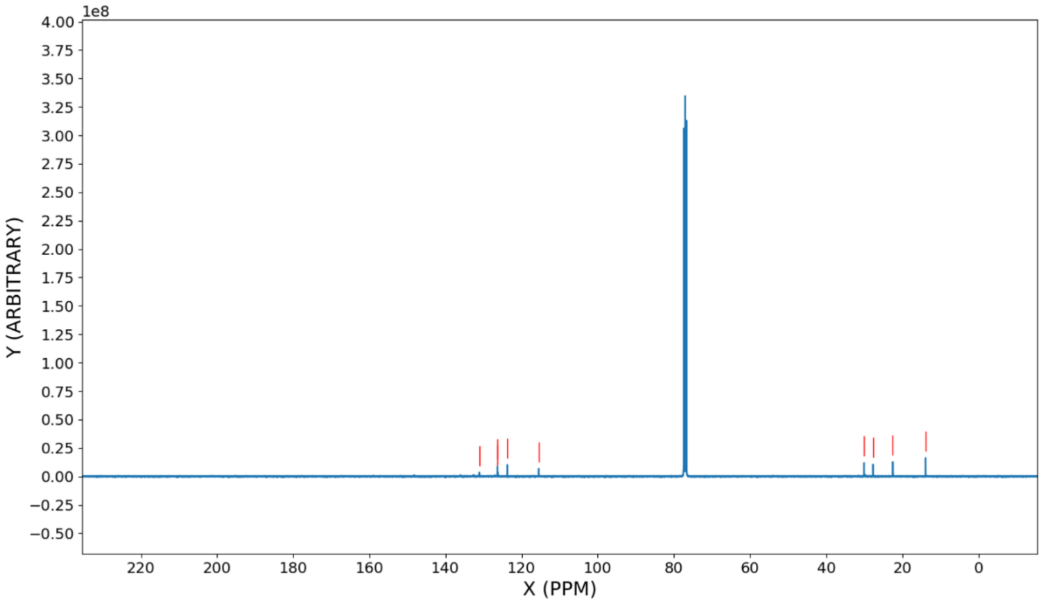

[**S7b**] 2,3-Bis(4-butyl-1*H*-1,2,3-triazol-1-yl)quinoxaline

CHMO:0000593 | ^{1}H nuclear magnetic resonance spectroscopy (^{1}H NMR)

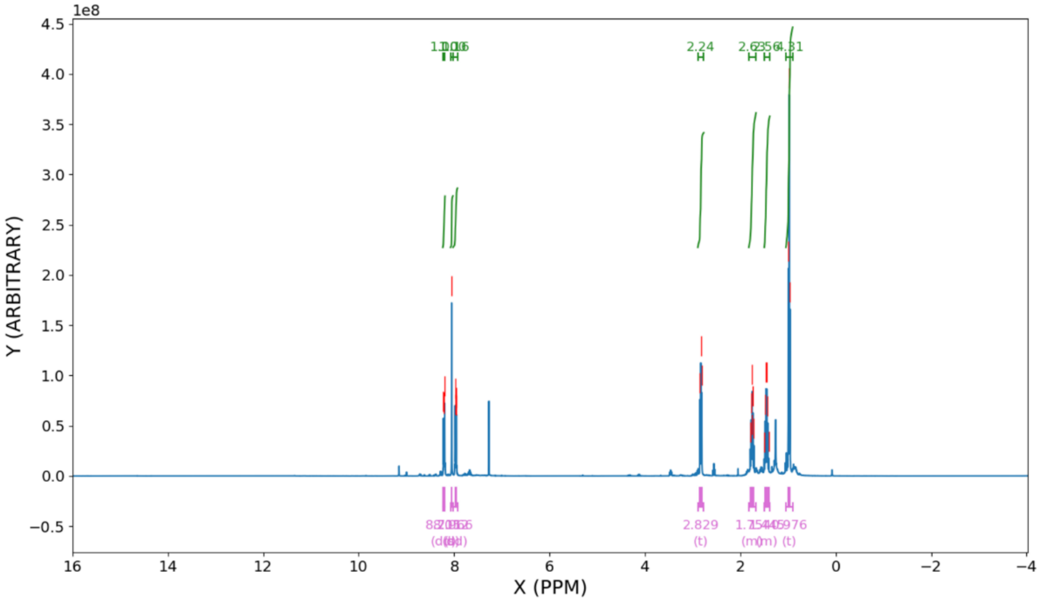

CHMO:0000595 | ^{13}C nuclear magnetic resonance spectroscopy (^{13}C NMR)

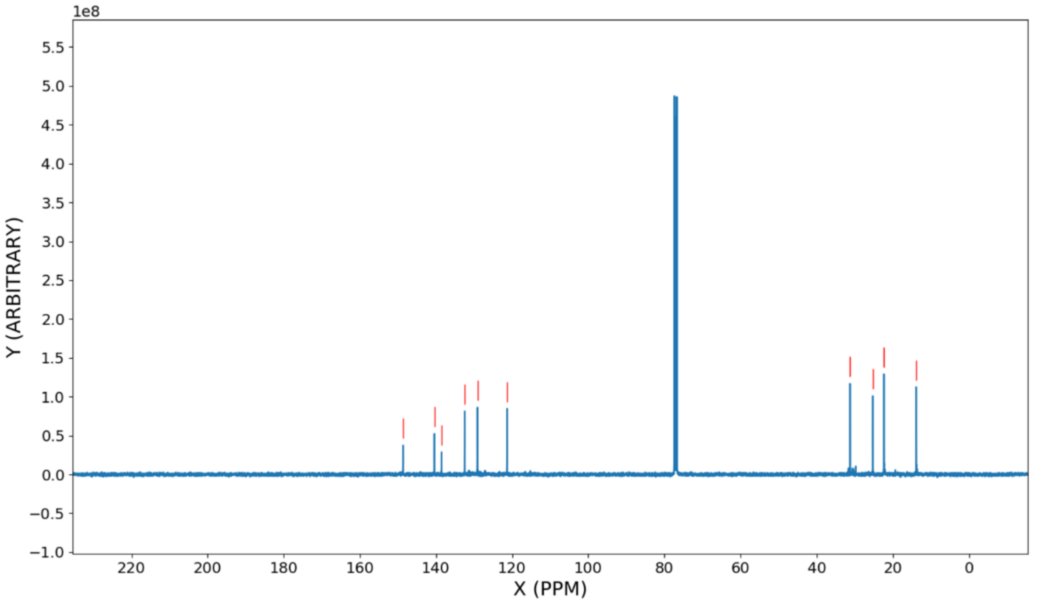

[**S6b**] 3-(4-Butyl-1*H*-1,2,3-triazol-1-yl)quinoxalin-2-amine

CHMO:0000593 | ^{1}H nuclear magnetic resonance spectroscopy (^{1}H NMR)

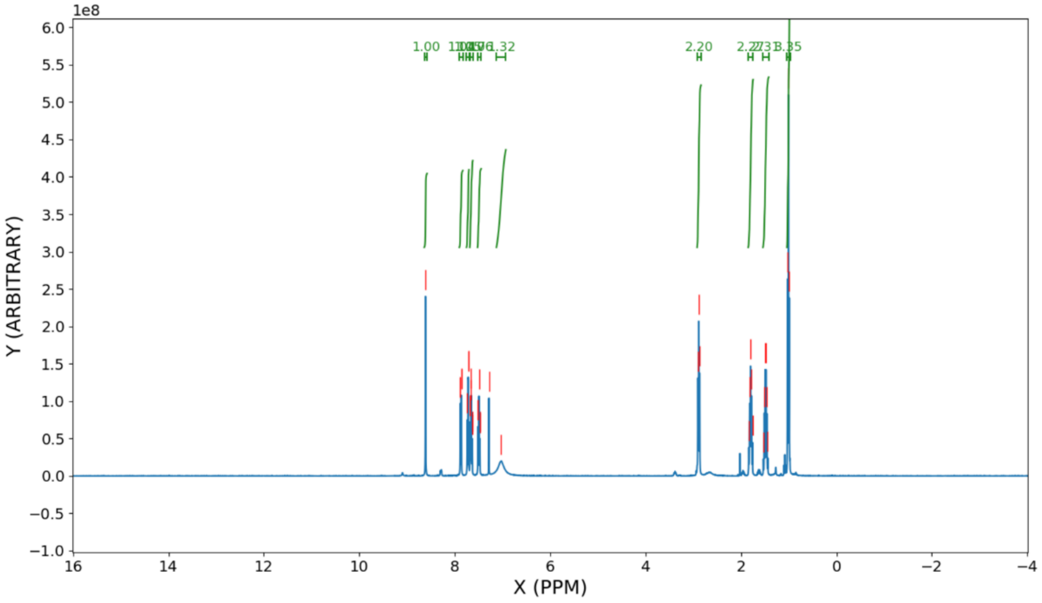

CHMO:0000595 | ^{13}C nuclear magnetic resonance spectroscopy (^{13}C NMR)

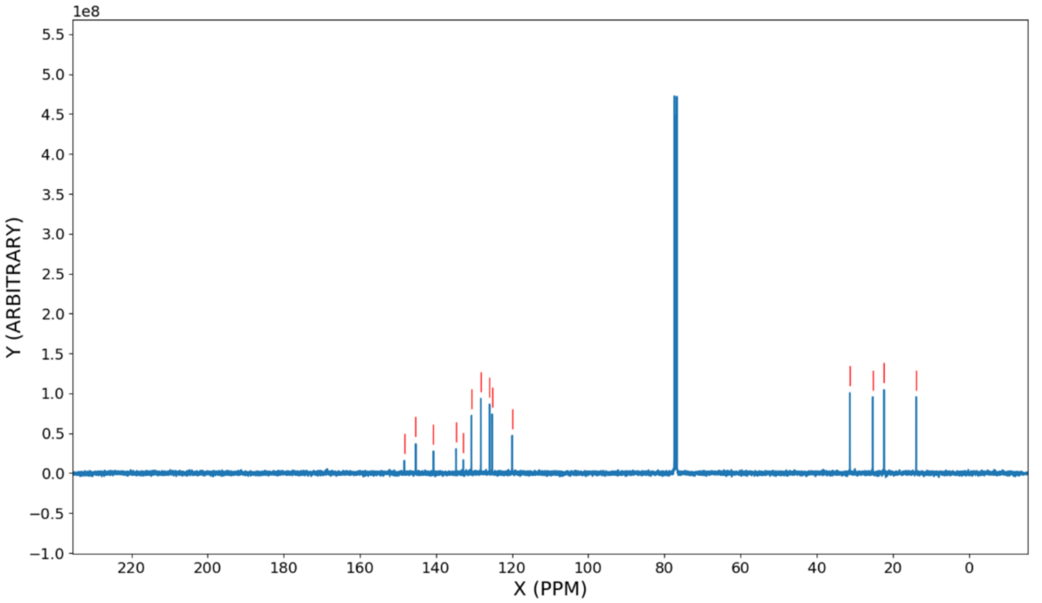

[**27a**] 2-(4-Butyl-1*H*-1,2,3-triazol-1-yl)quinoxalinetricarbonylrhenium(I)-bromide

CHMO:0000593 | ^{1}H nuclear magnetic resonance spectroscopy (^{1}H NMR)

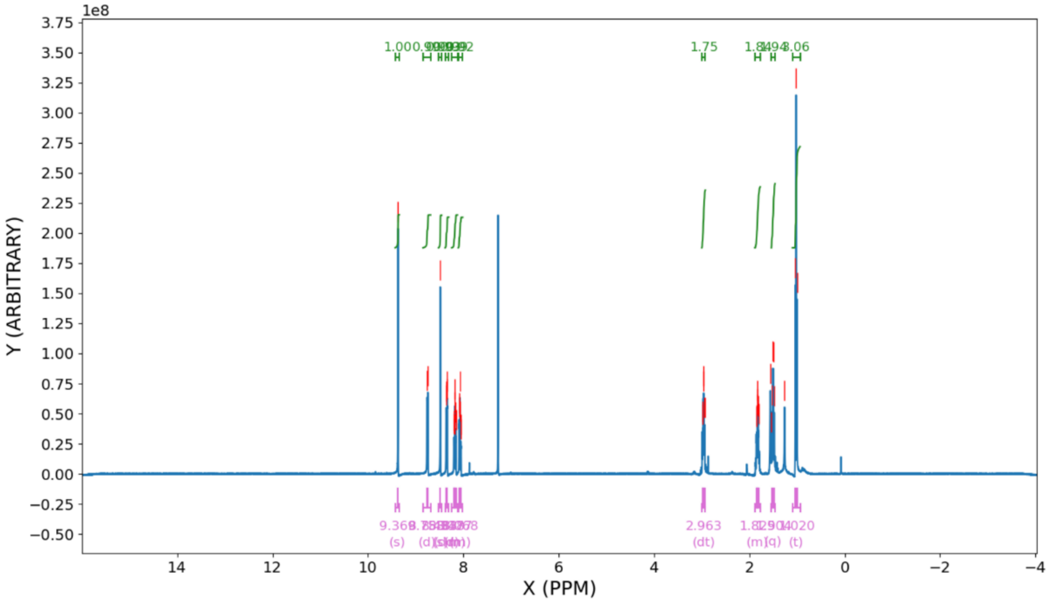

CHMO:0000595 | ^{13}C nuclear magnetic resonance spectroscopy (^{13}C NMR)

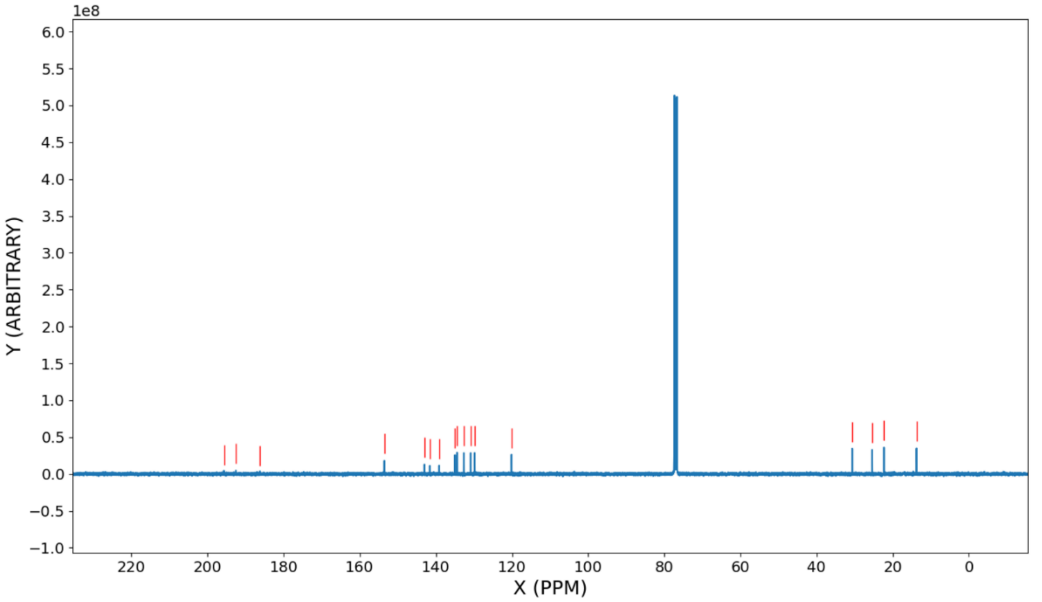

[**27b**] [(2-(4-Phenyl-1*H*-1,2,3-triazol-1-yl)quinoxaline)]bromotricarbonylrhenium(I)

CHMO:0000593 | ^{1}H nuclear magnetic resonance spectroscopy (^{1}H NMR)

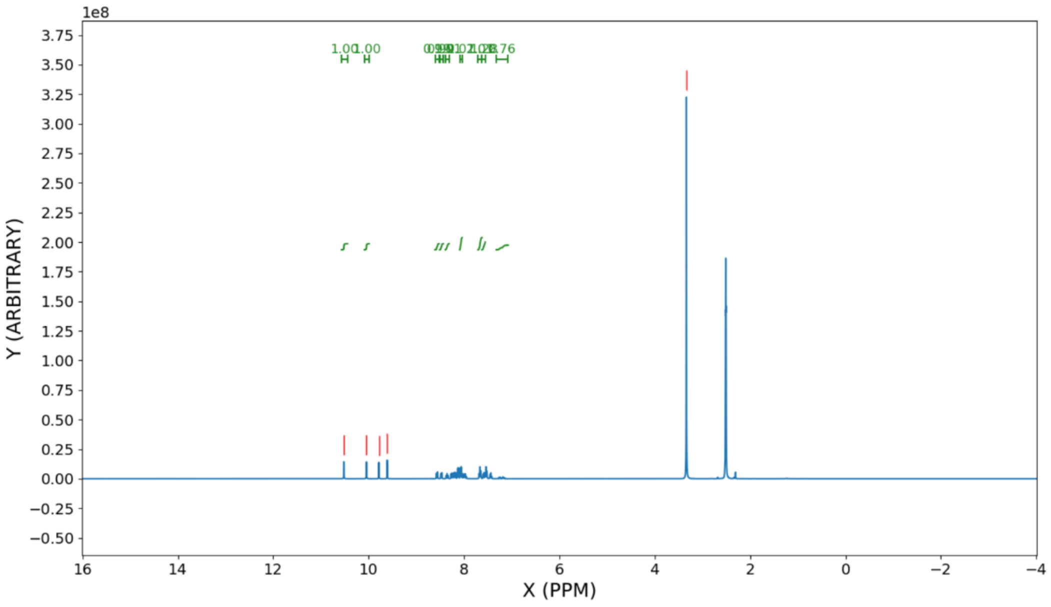

CHMO:0000595 | ^{13}C nuclear magnetic resonance spectroscopy (^{13}C NMR)

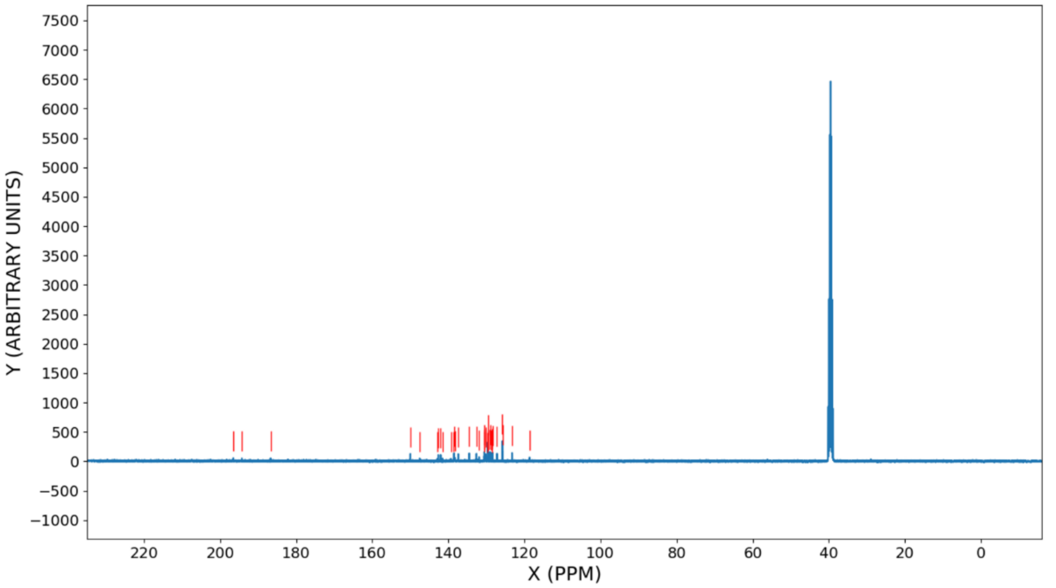

[**27c**] [2-(4-Butyl-1*H*-1,2,3-triazol-1-yl)-3-methylquinoxaline]bromotricarbonylrhenium(I)

CHMO:0000593 | ^{1}H nuclear magnetic resonance spectroscopy (^{1}H NMR)

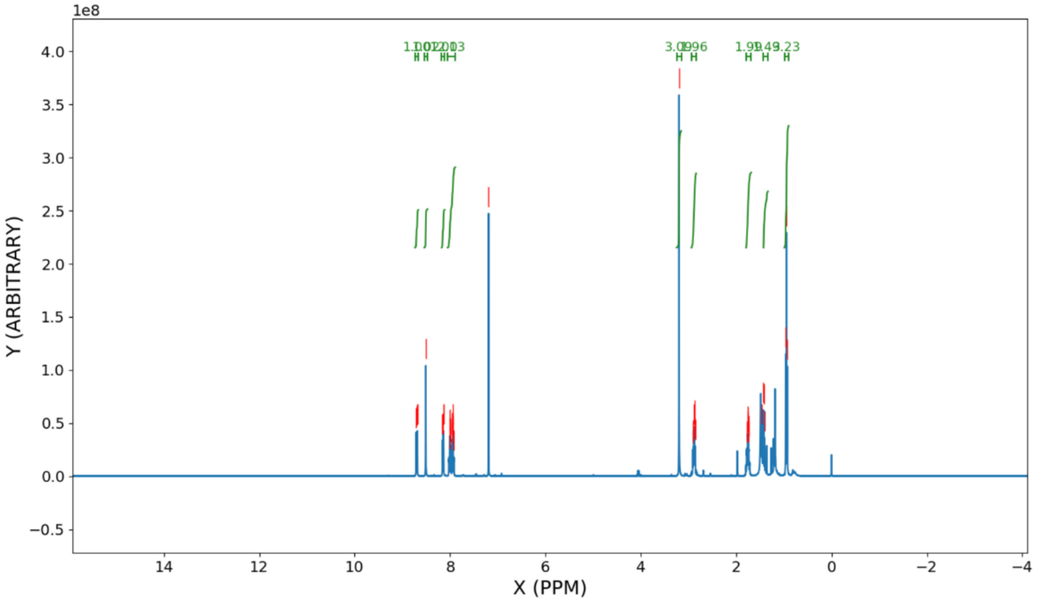

CHMO:0000595 | ^{13}C nuclear magnetic resonance spectroscopy (^{13}C NMR)

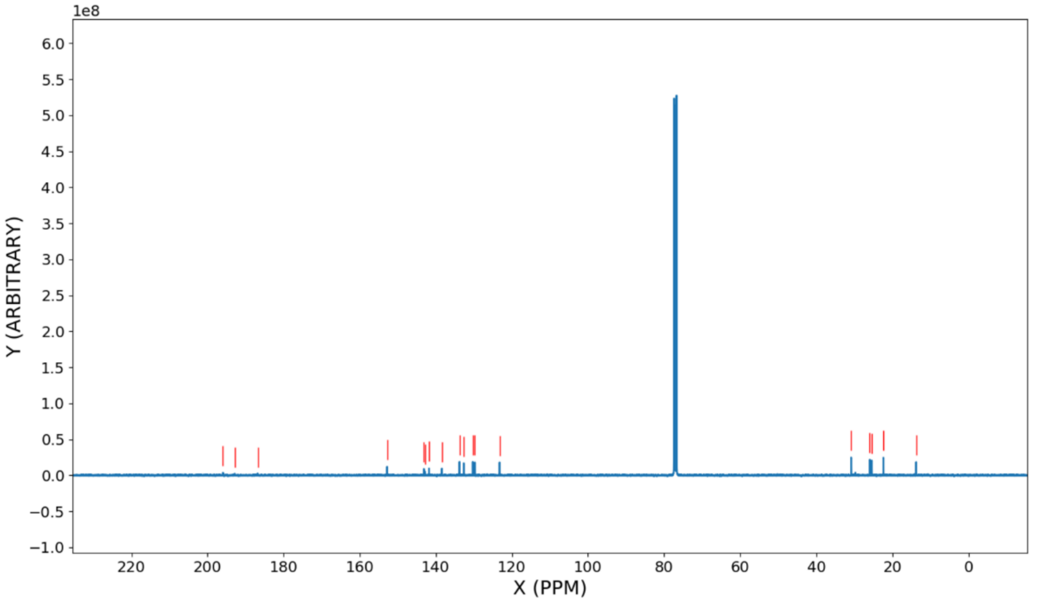

[**27d**] [2-(4-Butyl-1*H*-1,2,3-triazol-1-yl)-3-phenylquinoxaline]bromotricarbonylrhenium(I)

CHMO:0000593 | ^{1}H nuclear magnetic resonance spectroscopy (^{1}H NMR)

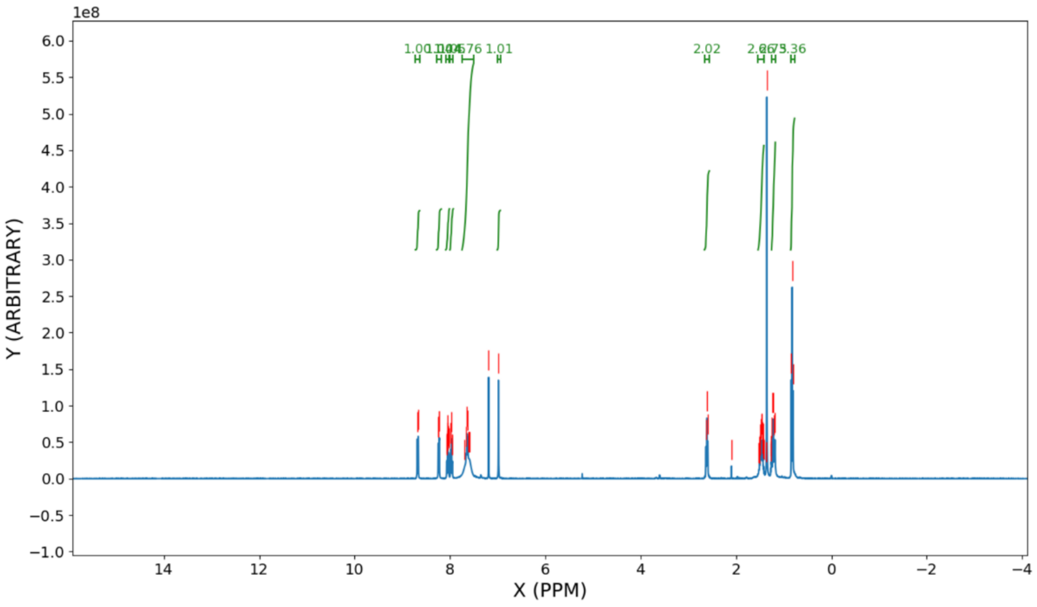

CHMO:0000595 | ^{13}C nuclear magnetic resonance spectroscopy (^{13}C NMR)

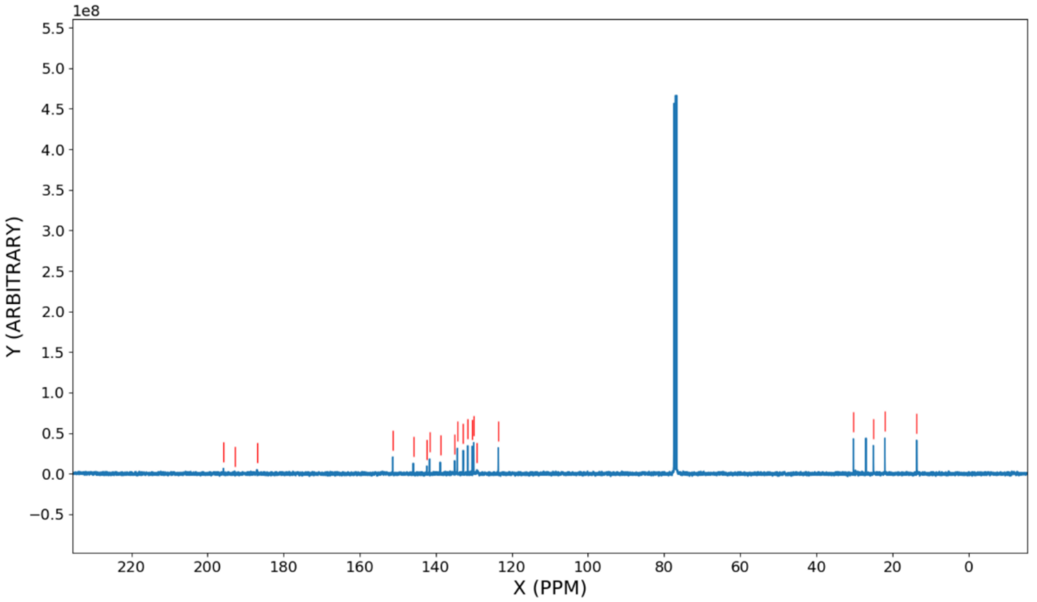

[**29**] [*N*,*N*-Diethyl-2-(1-(quinoxalin-2-yl)-1*H*-1,2,3-triazol-4-yl)ethan-1-amine]bromotricarbonylrhenium(I)

CHMO:0000593 | ^{1}H nuclear magnetic resonance spectroscopy (^{1}H NMR)

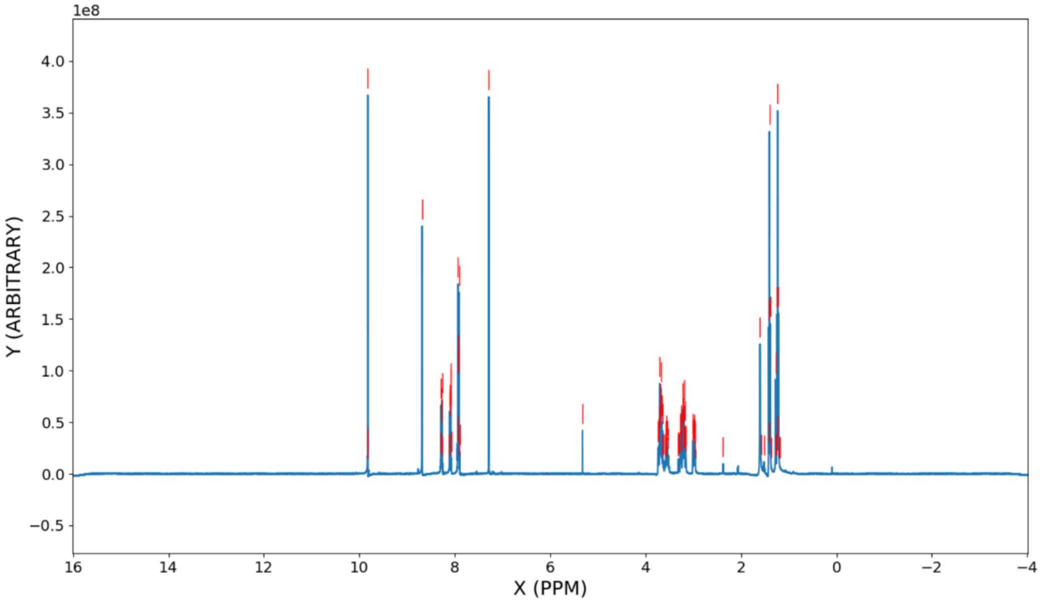

CHMO:0000595 | ^{13}C nuclear magnetic resonance spectroscopy (^{13}C NMR)

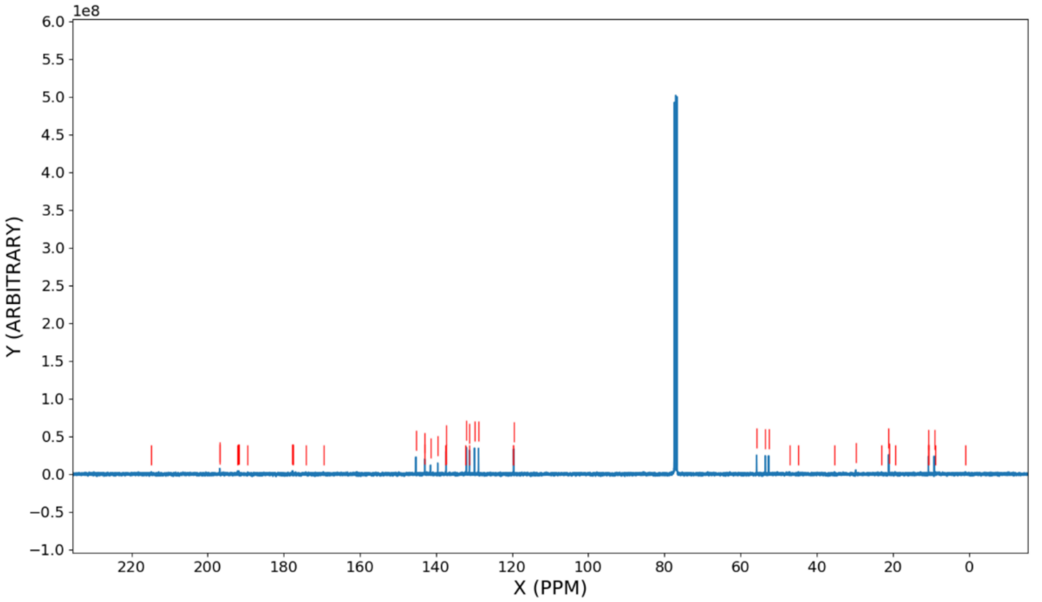

[**30**] [1-Butyl-4-(4-butyl-1*H*-1,2,3-triazol-1-yl)imidazo[1,2-*a*]quinoxaline]bromotricarbonylrhenium(I)

CHMO:0000593 | ^{1}H nuclear magnetic resonance spectroscopy (^{1}H NMR)

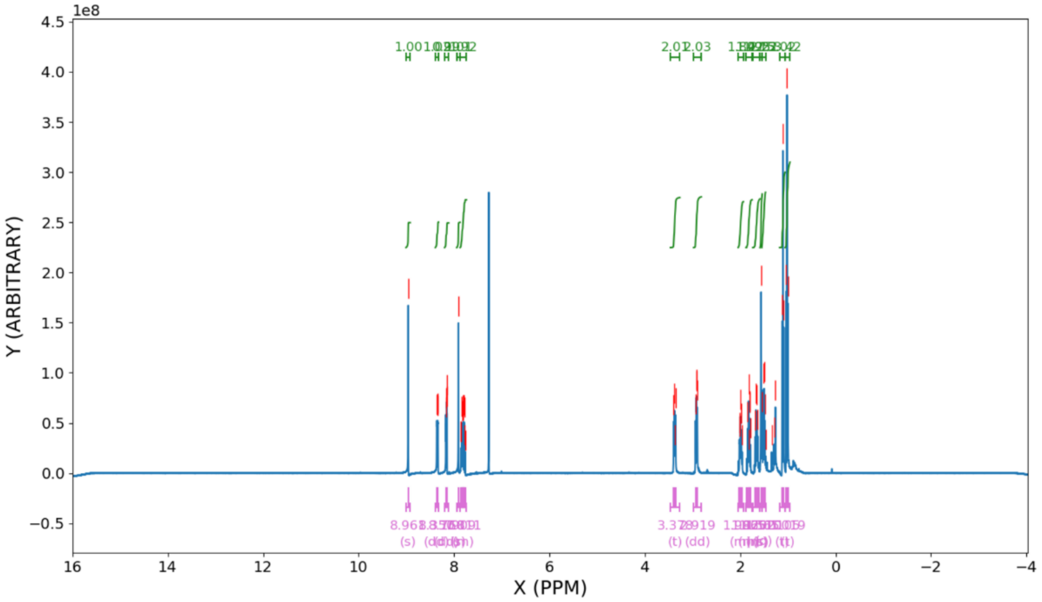

CHMO:0000595 | ^{13}C nuclear magnetic resonance spectroscopy (^{13}C NMR)

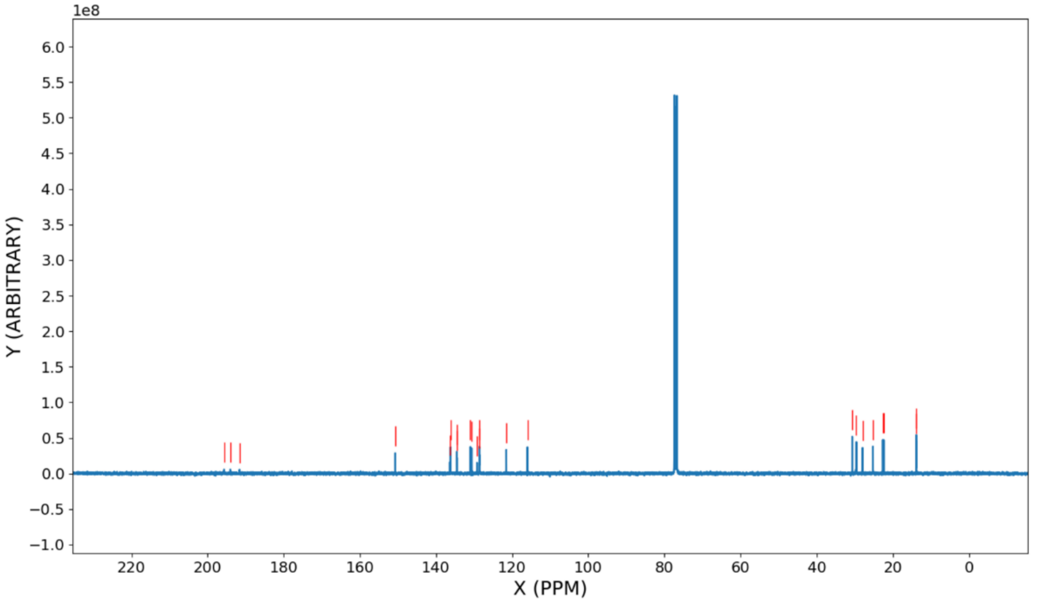

Supplement: File 2 — NMR spectra. [file Beilstein_J_Org_Chem-18-1088-s002.pdf]
